# Iron‐Catalyzed Borrowing Hydrogen *C*‐Alkylation of Oxindoles with Alcohols

**DOI:** 10.1002/cssc.201900799

**Published:** 2019-05-07

**Authors:** Mubarak B. Dambatta, Kurt Polidano, Alexander D. Northey, Jonathan M. J. Williams, Louis C. Morrill

**Affiliations:** ^1^ Cardiff Catalysis Institute School of Chemistry Cardiff University Main Building, Park Place Cardiff CF10 3AT UK; ^2^ Department of Chemistry University of Bath Claverton Down Bath BA2 7AY UK

**Keywords:** alcohols, alkylation, borrowing hydrogen, iron catalysis, oxindoles

## Abstract

A general and efficient iron‐catalyzed *C*‐alkylation of oxindoles has been developed. This borrowing hydrogen approach employing a (cyclopentadienone)iron carbonyl complex (2 mol %) exhibited a broad reaction scope, allowing benzylic and simple primary and secondary aliphatic alcohols to be employed as alkylating agents. A variety of oxindoles underwent selective mono‐*C*3‐alkylation in good‐to‐excellent isolated yields (28 examples, 50–92 % yield, 79 % average yield).

The oxindole framework is present in a diverse array of naturally occurring compounds.^1^ Furthermore, oxindoles that are mono‐ or disubstituted at the *C*3 position are commonly employed in drug discovery programs,^2^ with examples including the development of HIV‐1 non‐nucleoside reverse transcriptase inhibitors, spirocyclic compounds with anti‐cancer and anti‐inflammatory properties, and antagonists of progesterone and 5‐hydroxytryptamine_7_ (5‐HT_7_) receptors (Scheme 1 A). The traditional method for alkylation of unprotected oxindoles employs toxic alkyl halides and exhibits poor selectivity (mono‐ vs. dialkylation, *C*‐ vs. *N*‐alkylation) alongside the generation of stoichiometric quantities of undesired byproducts.^3^ An alternative approach employs the borrowing hydrogen (BH) principle, also known as hydrogen autotransfer, which allows bench‐stable and inexpensive alcohols to be used as alkylating agents, generating water as the sole byproduct.^4^ Recent progress in this area has provided alternatives to commonly employed precious‐metal catalysts through the development of catalysts based on earth‐abundant first‐row transition metals.^5^

**Scheme 1:**
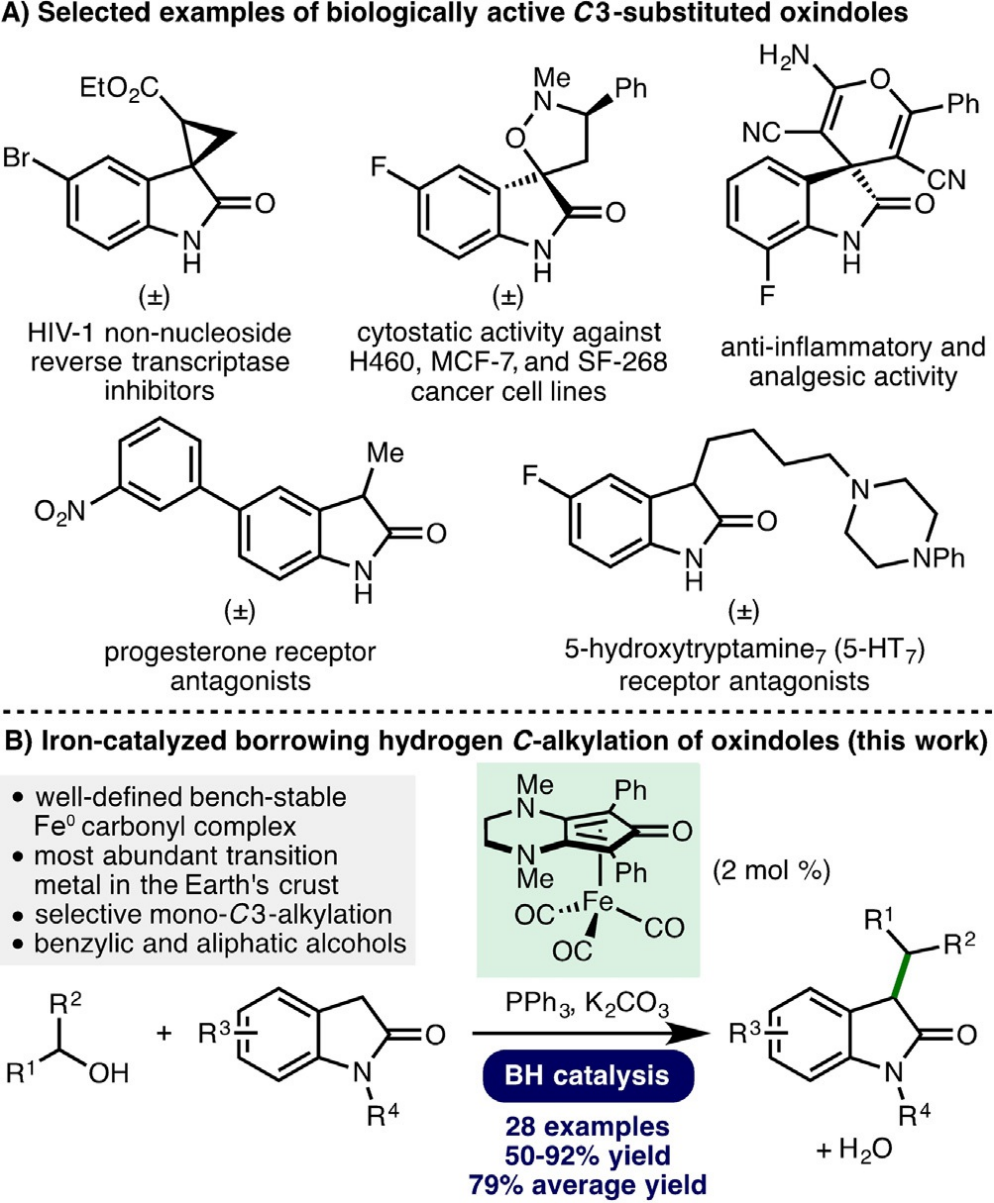
Oxindole importance and project overview.

The BH alkylation of oxindoles with alcohols, which selectively produces mono‐*C*3‐alkylation products, has been reported through heterogeneous catalysis^6^ and by employing homogeneous precious‐metal catalyst systems based on ruthenium and iridium.^7^ However, with respect to earth‐abundant first‐row transition‐metal catalysis, only sporadic examples appear in the literature, in each case forming only a minor component of a broader study.^8^ As such, the development of a general catalytic BH *C*‐alkylation of oxindoles with well‐defined complexes based on earth‐abundant first‐row transition metals is required and would represent a valuable addition to the synthetic toolbox. To this end, herein we report the use of a bench‐stable (cyclopentadieneone)iron(0) carbonyl complex (2 mol %) for the selective mono‐*C*3‐alkylation of various oxindoles with both benzylic and simple primary and secondary aliphatic alcohols as alkylating agents (Scheme 1 B).^9^

To commence our studies, we selected the *C*3‐benzylation of oxindole **2** with benzyl alcohol **1** (1.2 equiv.) as a model system (Table 1). After extensive optimization,^10^ it was found that a BH system composed of the bench‐stable (cyclopentadieneone)iron(0) carbonyl complex **3** (2 mol %),^11^ triphenylphosphine (4 mol %) to form the active catalyst, and K_2_CO_3_ (0.5 equiv.) as base in xylenes ([**2**]=0.5 m) at 150 °C for 24 h enabled the efficient *C*‐benzylation of **2**, giving **4** in 97 % yield based on ^1^H NMR spectroscopy and 90 % isolated yield (entry 1).^12^ Importantly, only 1.2 equiv. of the alkylating agent and substoichiometric quantities of base were required for complete conversion, giving a high‐atom‐economy process.^13^ No alkylation occurred in the absence of iron precatalyst **3** (entry 2), and only 26 % conversion was observed in the absence of K_2_CO_3_ (entry 3). The PPh_3_‐bound [Fe] precatalyst **5** could be employed, accessing **4** in 95 % yield based on ^1^H NMR spectroscopy (entry 4), verifying it as a plausible catalytic intermediate (see also Scheme 3). Interestingly, from the iron complexes employed in this study, it was found that the (cyclopentadienone)iron carbonyl precatalysts **3** and **5**, which contain a more electron‐rich cyclopentadienone framework, were uniquely effective for the desired transformation, whereas the use of alternative iron precatalysts **6**–**10** resulted in low‐to‐negligible formation of alkylated oxindole **4** (entries 5–9).^14^ The reaction could be performed in the absence of PPh_3_, albeit in a slightly diminished yield, indicating thermal activation of the precatalyst occurred at 150 °C (entry 10).^11^ Substituting triphenylphopshine for trimethylamine *N*‐oxide (4 mol %)^15^ also had a slightly negative impact on the reaction (entry 11). Employing Cs_2_CO_3_ as base resulted in lower conversion to **4** (entry 12). Lowering the quantity of K_2_CO_3_ (entry 13) highlighted that catalytic quantities of base (10 mol %) can be employed, accessing **4** in 88 % yield based on ^1^H NMR spectroscopy. Employing toluene as solvent (entry 14), increasing the reaction concentration (entry 15), lowering the reaction temperature (entry 16), reducing the reaction time (entry 17), or reducing the catalyst loading (entry 18) all lowered the efficiency of the iron‐catalyzed mono‐*C*3‐benzylation of **2**.

**Table 1:** Optimization of the Fe‐catalyzed oxindole *C*‐benzylation.^[a]^ 
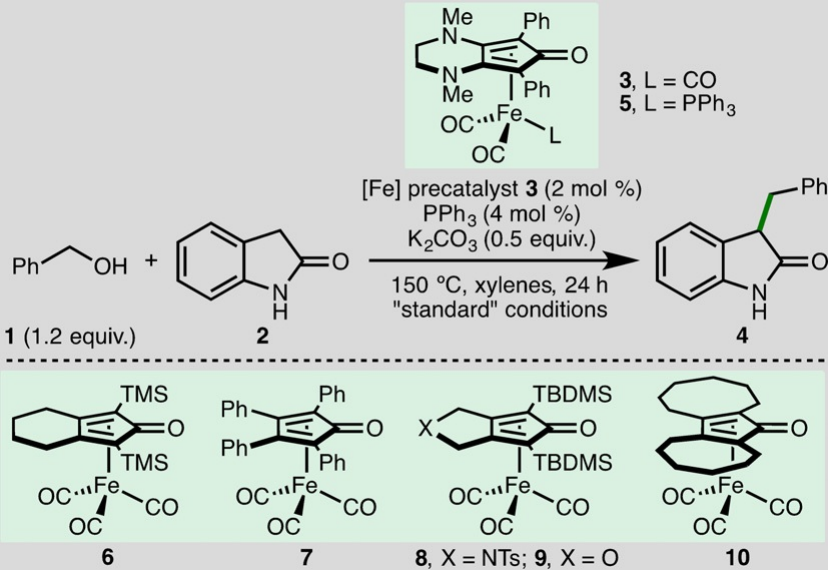

Entry	Variation from “standard” conditions	Yield^[b]^ [%]
1	none	97 (90)
2	no [Fe] precatalyst **3**	<2
3	no K_2_CO_3_	26
4^[c]^	**5** (2 mol %) instead of **3**	95
5	**6** (2 mol %) instead of **3**	18
6	**7** (2 mol %) instead of **3**	5
7	**8** (2 mol %) instead of **3**	5
8	**9** (2 mol %) instead of **3**	5
9	**10** (2 mol %) instead of **3**	5
10	no PPh_3_ activator	90
11	Me_3_NO (4 mol %) instead of PPh_3_	92
12	Cs_2_CO_3_ (0.5 equiv.) instead of K_2_CO_3_	85
13	K_2_CO_3_ (0.1 equiv.)	88
14	toluene instead of xylenes	91
15	[**2**]=1 m	93
16	130 °C	86
17	reaction time=6 h	92
18^[d]^	[Fe] precatalyst **3** (1 mol %)	73

[a] Reactions performed with oxindole **2** (1 mmol) and bench‐grade xylenes. [**2**]=0.5 m. [b] Yield after 24 h as determined by ^1^H NMR spectroscopy of the crude reaction mixture with 1,3,5‐trimethylbenzene as the internal standard. Isolated yield given in parentheses. [c] No PPh_3_. [d] 2 mol % of PPh_3_.

The full scope of the Fe‐catalyzed BH *C*3‐alkylation of oxindoles was explored, starting with the *C*‐alkylation of oxindole **2** (Scheme 2 A, B).^16^ Under the optimized reaction conditions (Table 1, entry 1) a variety of substituted benzylic alcohols could be employed as alkylating agents, giving the corresponding mono‐*C*3‐alkylated oxindoles in excellent isolated yields (products **4** and **11**–**24**, 52–91 % yield). With regard to the alcohol, sterically encumbered aryl units such as *o*‐tolyl and 1‐naphthyl were tolerated in addition to electron‐donating (4‐OMe, 4‐OBn) and electron‐withdrawing (4‐CF_3_, 4‐CN) substituents. The catalytic system exhibited chemoselectivity, tolerating the reducible nitrile and alkene moieties present within products **19** and **20**. 4‐Iodobenzyl alcohol was employed as the alkylating agent, incorporating an additional functional handle into oxindole **21** for subsequent elaboration through established cross‐coupling methods.^17^ Furan‐2‐ylmethanol and thiophene‐2‐ylmethanol were both compatible with this methodology, incorporating an additional heterocycle into products **23** and **24**, which were isolated in 77 and 84 % yield, respectively. We were pleased to discover that less activated simple aliphatic alcohols could also be employed as alkylating agents in this process (products **25**–**31**, 53–84 % yield). In each case, the alcohol was used as solvent to obtain high isolated yields of the mono‐*C*3‐alkylated oxindoles. Under otherwise identical reaction conditions, decan‐1‐ol, butan‐1‐ol, ethanol, and methanol were all successfully utilized as alkylating agents. 1,4‐Butanediol was also employed as the alkylating agent, accessing the mono‐*C*3‐alkylated oxindole **29** in 53 % isolated yield, with no dialkylation products observed. Remarkably, it was found that the unactivated secondary alcohols propan‐2‐ol and butan‐2‐ol were also tolerated, giving alkylated oxindoles **30** and **31** in excellent isolated yields. This is a rare example of secondary alcohol compatibility as alkylating agents in BH catalysis employing earth‐abundant first‐row transition‐metal catalysts.9e, 9f, 9m, 18 Unfortunately, despite examining a range of alternative reaction conditions, benzylic alcohols containing nitro or ketone functional groups, allylic alcohols, propargylic alcohols, and bulkier secondary alcohols (e.g., 1‐phenylethan‐1‐ol) were found to be incompatible with this *C*‐alkylation procedure.

**Scheme 2:**
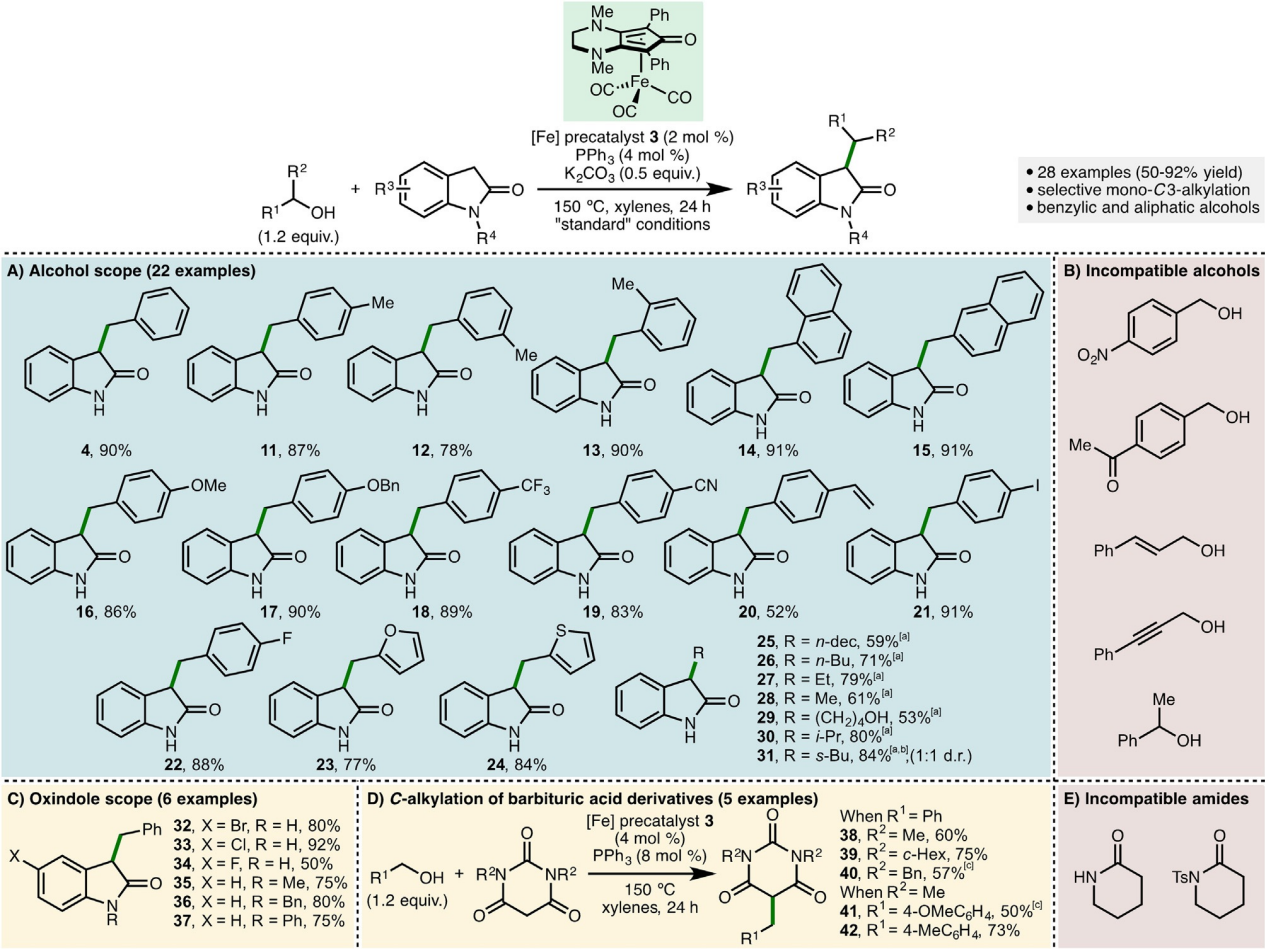
Scope of the Fe‐catalyzed *C*‐alkylation of oxindoles. Reactions performed with oxindole starting material (1 mmol) and bench‐grade xylenes. All yields are isolated yields after chromatographic purification. Reagents and conditions: [a] alcohol used as solvent; [b] [Fe] precatalyst **3** (4 mol %), PPh_3_ (8 mol %); [c] K_2_CO_3_ (0.5 equiv.).

Next, we explored the scope of the reaction with respect to variation within the oxindole component (Scheme 2 C). By employing the optimized reaction conditions (Table 1, entry 1) a variety of substituted oxindoles underwent efficient and selective mono‐*C*3‐alkylation with benzyl alcohol (products **32**–**37**, 50–92 % yield). Oxindoles containing halogen substitution at the 5‐position (5‐Br, 5‐Cl, and 5‐F) in addition to *N*‐methyl, *N*‐benzyl, and *N*‐phenyl substitution were all well tolerated. Barbituric acids are a class of activated amides that have been shown to participate as competent nucleophiles in homogeneous BH alkylation processes employing precious‐metal catalysts.^19^ By using the [Fe] precatalyst **3** (4 mol %), it was found that a selection of *N*‐alkyl barbituric acid derivatives underwent efficient *C*5‐monoalkylation, giving products **38**–**42** in 50–75 % isolated yield (Scheme 2 D). This iron‐catalyzed process is the first example of a BH alkylation of barbituric acid derivatives employing an earth‐abundant transition‐metal catalyst. Unfortunately, piperdin‐2‐one and 1‐tosylpiperdin‐2‐one were found to be incompatible with this protocol, with complex reaction mixtures obtained across a range of reaction conditions explored.

To obtain insights into the reaction mechanism, the α,β‐unsaturated amide **43** was synthesized and subjected to the “standard” *C*‐alkylation reaction conditions, which produced **4** in 71 % yield based on ^1^H NMR spectroscopy, indicating that **43** is a plausible reaction intermediate (Scheme 3 A). In line with this observation and previous related investigations,^11^ a plausible reaction mechanism begins with CO decoordination of the [Fe] precatalyst **3** by PPh_3_ to form the active iron complex, which abstracts hydrogen from benzyl alcohol in the presence of base to form the required transient reactive benzaldehyde intermediate (Scheme 3 B). Subsequent nucleophilic attack of oxindole **2** generates the β‐hydroxy amide **44**, which undergoes rapid base‐catalyzed E1cB dehydration to form the α,β‐unsaturated amide **43**. Finally, reduction of **43** by the iron‐hydrogen complex gives the *C*3‐alkylated product **4** with regeneration of the active iron complex.

**Scheme 3:**
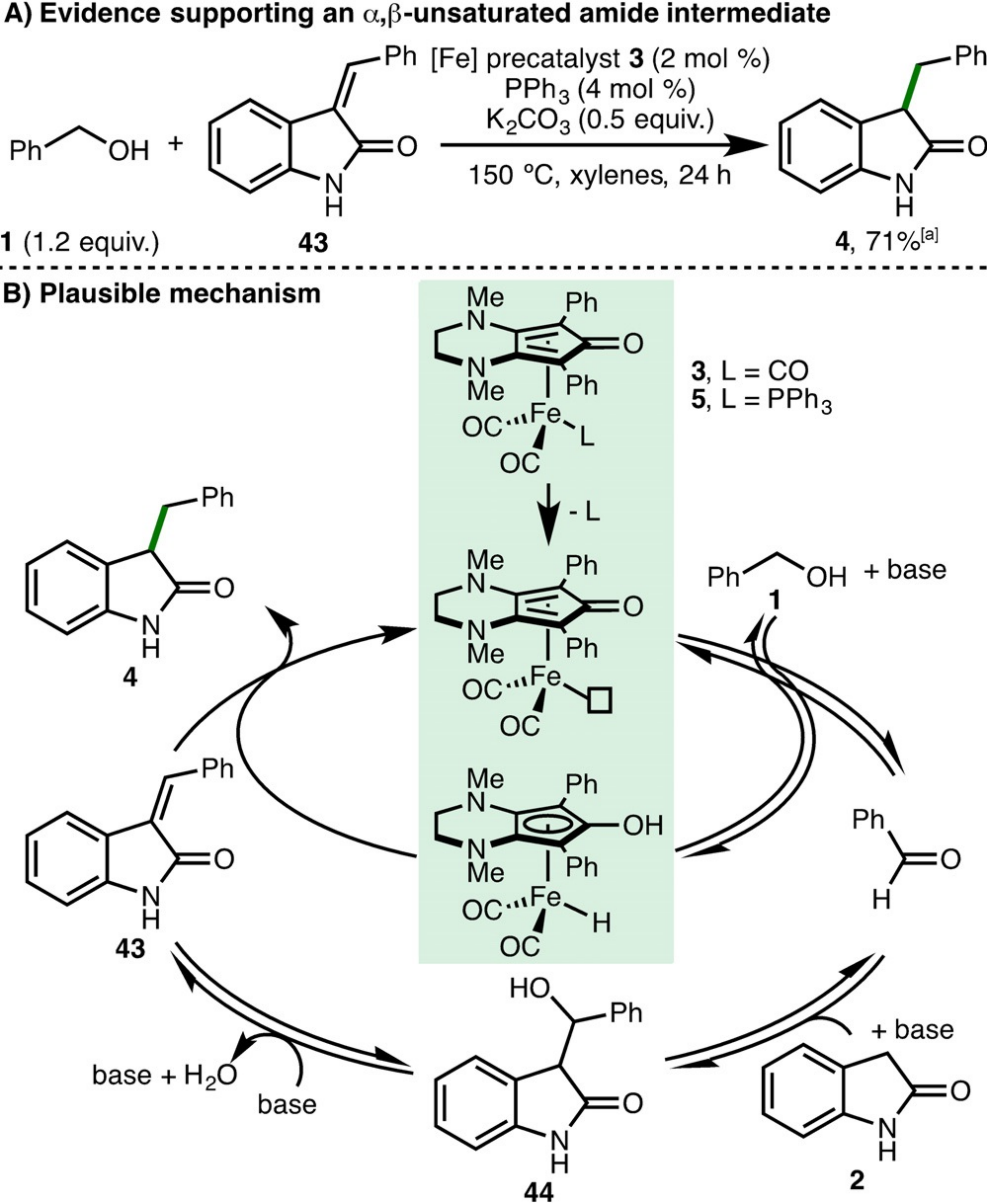
Mechanistic considerations. [a] Yield after 24 h as determined by ^1^H NMR spectroscopy of the crude reaction mixture with 1,3,5‐trimethylbenzene as the internal standard.

In conclusion, we have developed a general and efficient Fe‐catalyzed *C*‐alkylation of oxindoles with benzylic and simple primary and secondary aliphatic alcohols as alkylating agents through the borrowing hydrogen approach. A variety of oxindoles underwent selective mono‐*C*3‐alkylation in excellent isolated yields (28 examples, 50–92 % yield, 79 % average yield). Ongoing studies are focused on further applications of earth‐abundant first‐row transition metals in catalysis, and these results will be reported in due course.^20^

## Conflict of interest

*The authors declare no conflict of interest*.

## Supporting information

As a service to our authors and readers, this journal provides supporting information supplied by the authors. Such materials are peer reviewed and may be re‐organized for online delivery, but are not copy‐edited or typeset. Technical support issues arising from supporting information (other than missing files) should be addressed to the authors.

Supplementary
