# Supplementary material for: Iron‐Catalyzed Borrowing Hydrogen C‐Alkylation of Oxindoles with Alcohols
Source: ChemSusChem. 2019 May 7;12(11):2345–9. doi: 10.1002/cssc.201900799 (PMC6619250; doi:10.1002/cssc.201900799)
Supplement: Supplementary file 1 — Supplementary [file CSSC-12-2345-s001.pdf]

CHEMISTRY & SUSTAINABILITY
CHEMSUSCHEM
ENERGY & MATERIALS

Supporting Information

Iron-Catalyzed Borrowing Hydrogen C-Alkylation of Oxindoles with Alcohols

Mubarak B. Dambatta,[a] Kurt Polidano,[a] Alexander D. Northey,[a] Jonathan M. J. Williams,[b] and Louis C. Morrill*[a]

cssc_201900799_sm_miscellaneous_information.pdf

SUPPORTING INFORMATION

Iron-Catalyzed Borrowing Hydrogen *C*-Alkylation of Oxindoles Using Alcohols

Mubarak B. Dambatta,[†] Kurt Polidano,[†] Alexander D. Northey,[†] Jonathan M. J. Williams,[‡] and Louis C. Morrill*[†]

e-mail: MorrillLC@cardiff.ac.uk

[†]*School of Chemistry, Cardiff University, Main Building, Park Place, Cardiff, CF10 3AT, UK*
[‡]*Department of Chemistry, University of Bath, Claverton Down, Bath, BA2 7AY, UK*

Table of Contents

1. General information 2

2. Experimental and characterization data 3

2.1. Catalyst synthesis 3

2.2. Substrate synthesis 8

2.3. Optimization of Fe-catalyzed oxindole *C*(3)-benzylation 12

2.4. Substrate scope 13

2.4.1. General procedure 1 13

2.4.2. General procedure 2 14

2.4.3. General procedure 3 14

2.5. Evidence supporting an α,β-unsaturated amide intermediate 66

2.5.1. Synthesis of 3-benzylideneindolin-2-one 66

2.5.2. Mechanistic probe 67

3. References 69

1. General information

Unless stated otherwise, all reactions were performed using oven-dried 10 mL microwave vials sealed with an aluminium crimp caps, and were stirred with Teflon-coated magnetic stirrer bars. Dry tetrahydrofuran (THF), toluene, hexanes and diethyl ether were obtained after passing these previously degassed solvents through activated alumina columns (Mbraun, SPS-800). All other solvents and commercial reagents were used as supplied without further purification unless stated otherwise.
Room temperature (rt) refers to 20-25 °C. Ice/water and CO_2(s)/acetone baths were used to obtain temperatures of 0 °C and -78 °C respectively. All reactions involving heating were carried out using DrySyn blocks and a contact thermometer. *In vacuo* refers to reduced pressure through the use of a rotary evaporator. [Fe] precatalyst **5**[1] **6**[2] **7**,[3] **8**,[4] **9**,[4] and **10**[5] were all prepared according to respective procedures in the literature.
Analytical thin layer chromatography was carried out using aluminium plates coated with silica (Kieselgel 60 F_{254} silica) and visualization was achieved using ultraviolet light (254 nm), followed by staining with a 1% aqueous $KMnO_4$ solution. Flash chromatography used Kieselgel 60 silica in the solvent system stated.
Melting points were recorded on a Gallenkamp melting point apparatus, and corrected by linear interpolation of melting point standards benzophenone (47-49 ˚C), and benzoic acid (121-123 ˚C).
Infrared spectra were recorded on a Shimadzu IRAffinity-1 Fourier Transform ATIR spectrometer as thin films using a Pike MIRacle ATR accessory. Characteristic peaks are quoted (ν_{max} / cm^{-1}).
^{1}H, ^{13}C, ^{19}F NMR spectra were obtained on either a Bruker Avance 400 (400 MHz ^{1}H, 101 MHz ^{13}C, 376 MHz ^{19}F) or a Bruker Avance 500 (500 MHz ^{1}H, 126 MHz ^{13}C, 471 MHz ^{19}F) spectrometer at rt in the solvent stated. Chemical shifts are reported in parts per million (ppm) relative to the residual solvent signal. All coupling constants, *J*, are quoted in Hz. Multiplicities are reported with the following symbols: s = singlet, d = doublet, t = triplet, q = quartet, m = multiplet and multiples thereof. The abbreviation Ph to denote phenyl, br to denote broad.
High resolution mass spectrometry (HRMS, *m/z*) data was acquired either at Cardiff University on a Micromass LCT spectrometer or at the EPSRC UK National Mass Spectrometry Facility at Swansea University.

2. Experimental and characterization data

2.1. Catalyst synthesis

[Fe] precatalyst **3** was prepared via a 3-step synthesis as stated below:

4-hydroxy-2,5-diphenylcyclopent-4-ene-1,3-dione

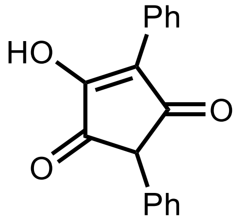

Under nitrogen, a flame dried Schlenk tube was charged with ethanol (20 mL) and metal sodium (920 mg, 40 mmol) at 0 °C. After complete dissolution, the solution was charged with 1,3-diphenylacetone (4.20 g, 20.0 mmol) and diethyl oxalate (2.70 mL, 2.92 g, 20 mmol). This was left to react at rt for 48 hours. The mixture was cooled to 0 °C and glacial acetic acid was carefully added dropwise until the colour turned yellow-orange. The reaction mixture was then poured into ice/water (100 mL) and the aqueous layer was acidified to pH 1 by careful dropwise addition of concentrated sulfuric acid (96%). The yellow solid was filtered. The precipitate was dissolved in acetone (50 mL) and transferred to a conical flask. It was dried over $MgSO_4$, filtered and concentrated *in vacuo*. Purification by recrystallization yielded a yellow solid (2.75 g, 52%), mp 168-170 °C (dec) ($CHCl_3$/hexanes), R_f = 0.33 (eluent = 100% EtOAc). **^{1}H NMR (500 MHz, $(CD_3)_2SO$)** δ_H: 4.49 (1H, s), 7.19 (2H, d, *J* 7.0), 7.28-7.46 (4H, m), 7.46-7.54 (2H, m), 7.98-8.10 (2H, m); **^{13}C NMR (126 MHz, $(CD_3)_2SO$)** δ_H: 55.9, 127.4, 128.1, 128.2, 128.7, 128.8, 128.8, 128.8, 129.5, 134.4, 166.4, 196.8, 197.5. Spectroscopic data in accordance with that stated in the literature.[6]

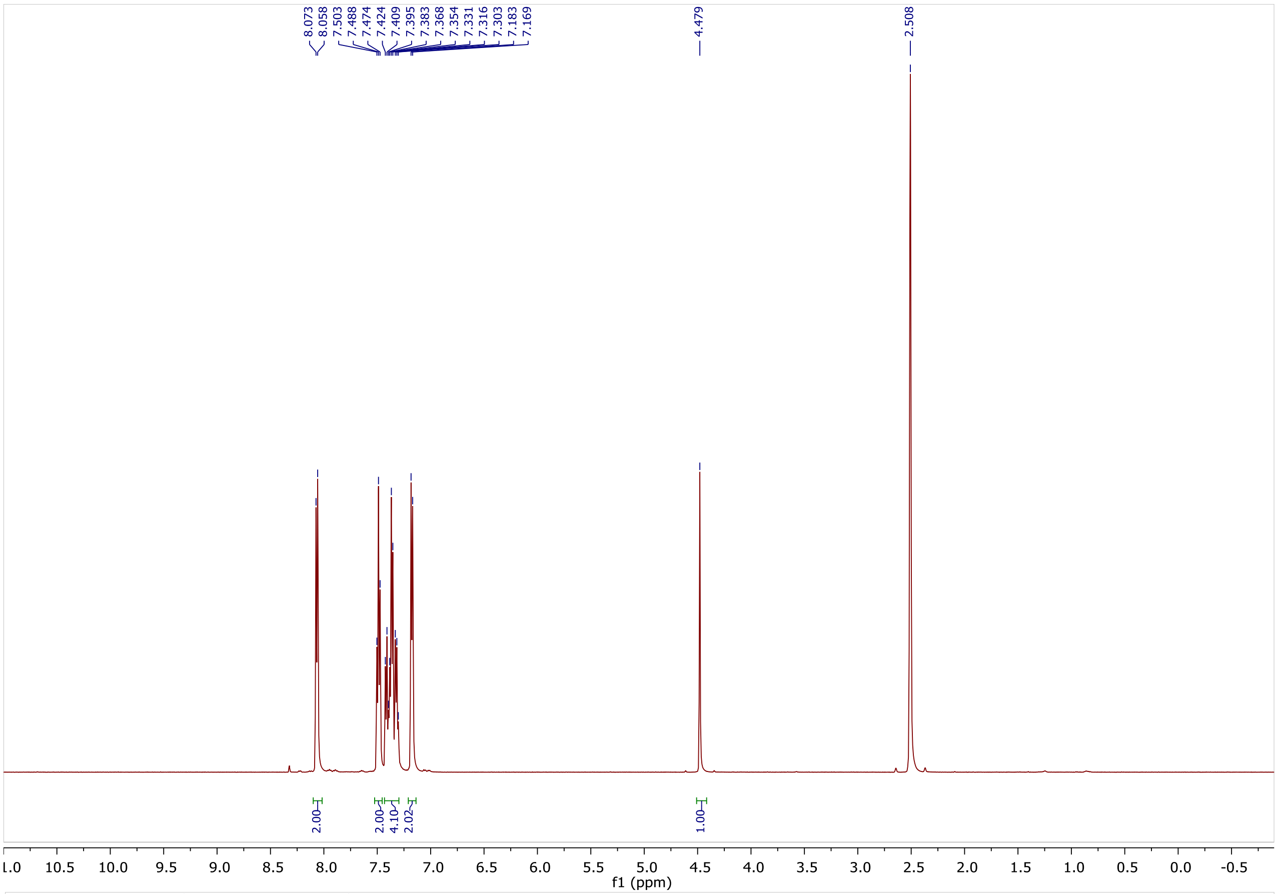
8.073
8.058
7.503
7.488
7.474
7.424
7.409
7.395
7.383
7.368
7.354
7.331
7.316
7.303
7.183
7.169
4.479
2.508
2.00
2.00
4.10
2.02
1.00
f1 (ppm)

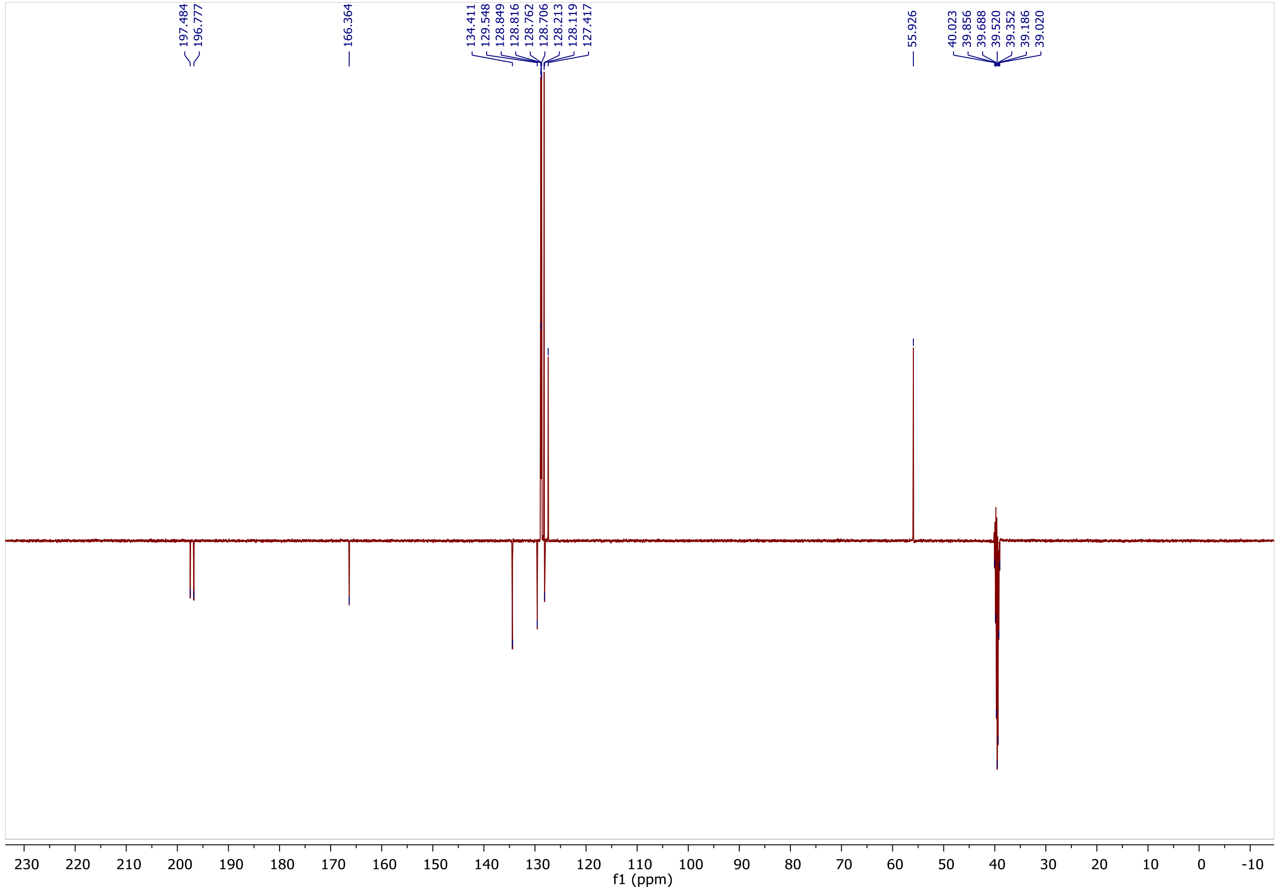
197.484
196.777
166.364
134.411
129.548
128.849
128.816
128.762
128.706
128.213
128.119
127.417
55.926
40.023
39.856
39.688
39.520
39.352
39.186
39.020
f1 (ppm)

1,4-dimethyl-5,7-diphenyl-1,2,3,4-tetrahydro-6H-cyclopenta[b]pyrazin-6-one

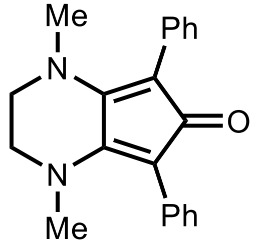

Under nitrogen, a flame dried round-bottomed flask was charged with 4-hydroxy-2,5-diphenylcyclopent-4-ene-1,3-dione (2.51 g, 9.50 mmol), methanol (15 mL) and *N*,*N'*-dimethylethylenediamine (1.23 mL, 1.00 g, 11.4 mmol). The mixture was heated under reflux for 5 h. It was then cooled and concentrated *in vacuo*, leading to the formation of the pure compound (2.86 g, 95%), mp 184-186 °C, R_f = 0.50 (eluent = 5% MeOH in CH_2Cl_2). **^{1}H NMR (500 MHz, $CDCl_3$)** δ_H: 2.84 (6H, s), 3.36 (4H, s), 7.12-7.19 (2H, m), 7.23-7.32 (8H, m); **^{13}C NMR (126 MHz, $CDCl_3$)** δ_C: 42.2, 50.1, 99.0, 125.6, 127.4, 131.2, 133.8, 151.0, 195.4. Spectroscopic data in accordance with that stated in the literature.[6]

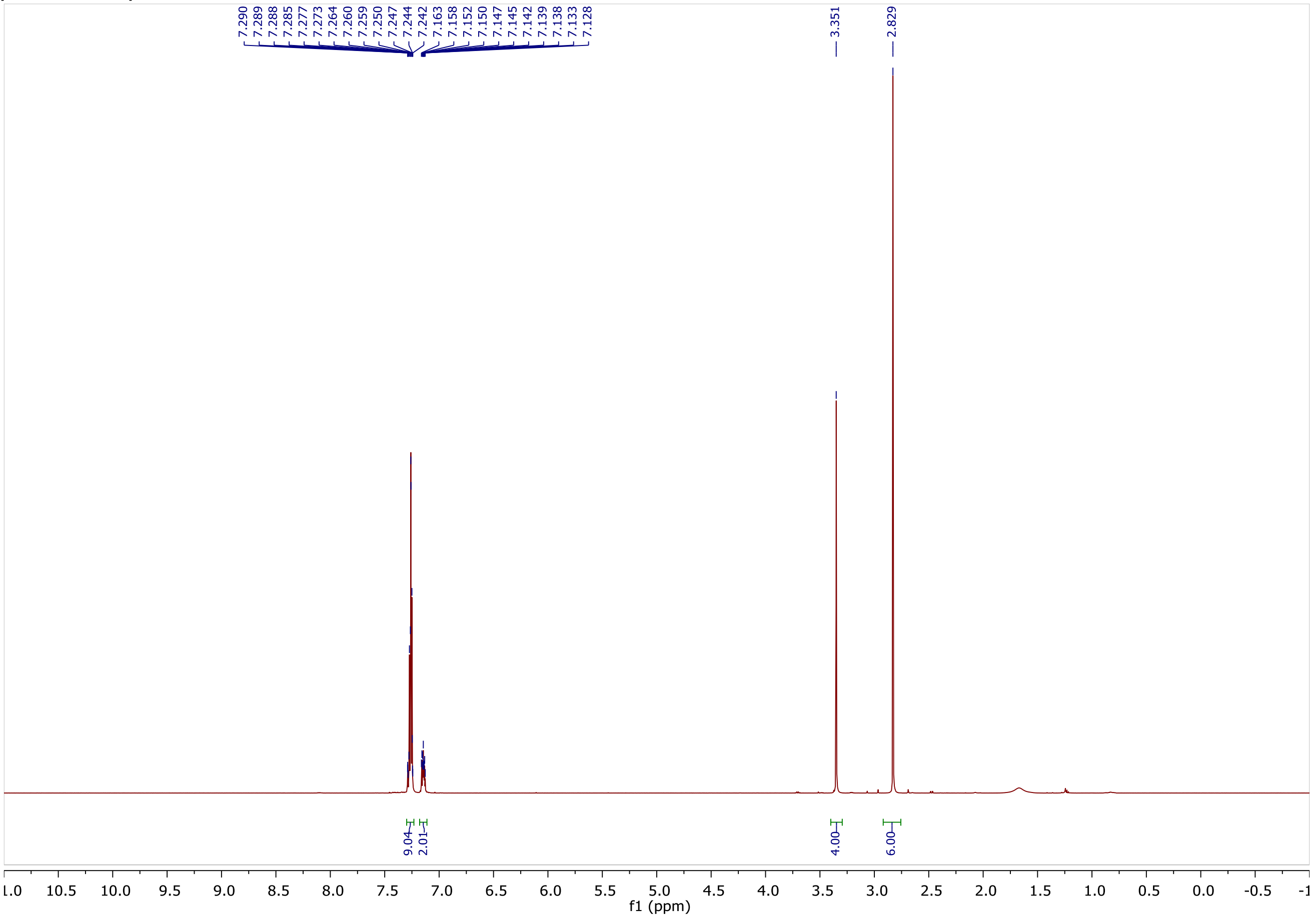

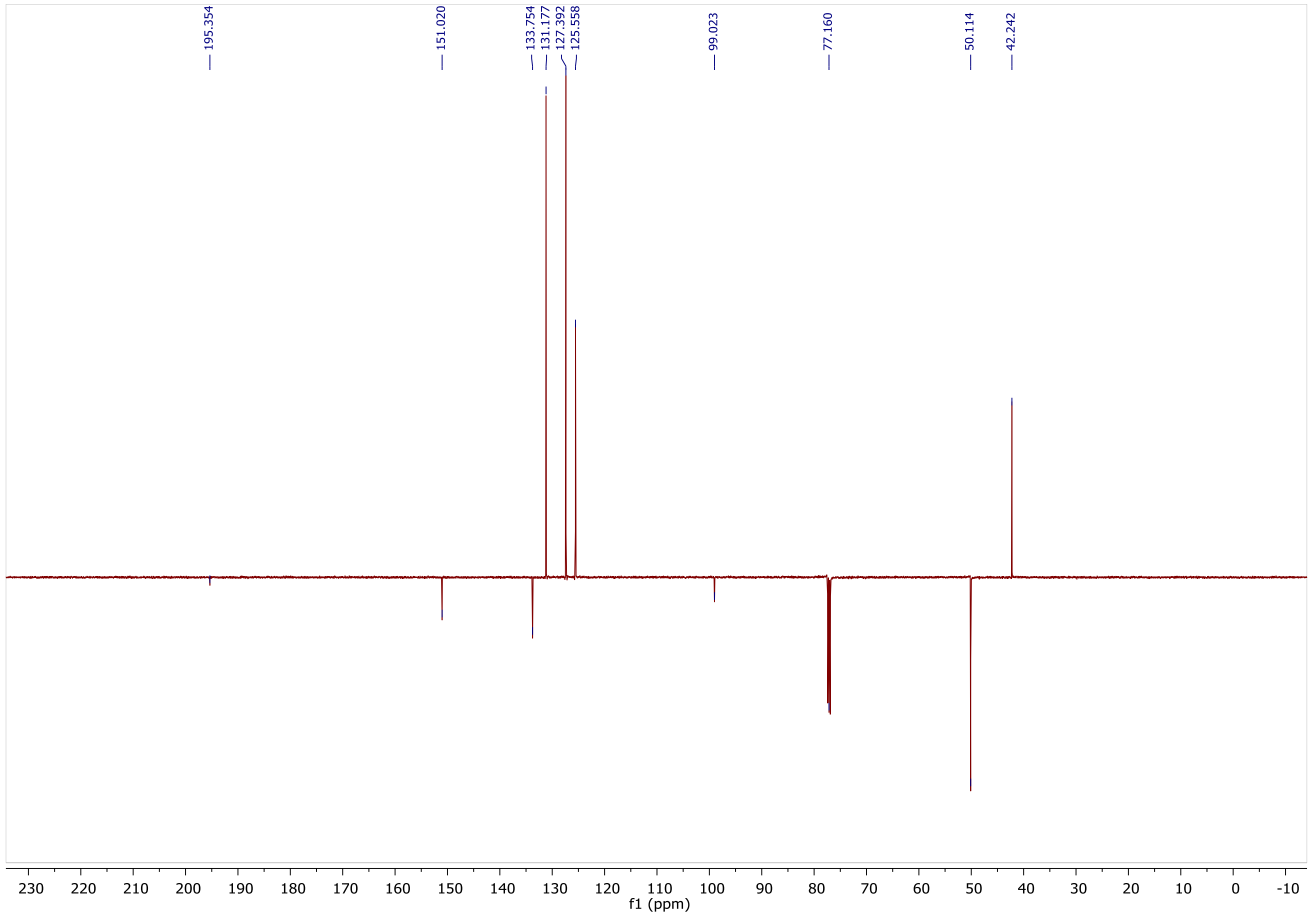

(1,4-dimethyl-5,7-diphenyl-1,2,3,4-tetrahydro-6H-cyclopenta[b]pyrazin-6-one)tricarbonyliron

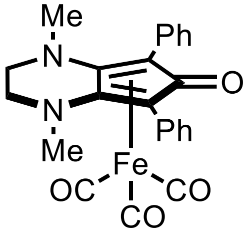

3

Under nitrogen, a flame dried Schlenk tube was charged with 1,4-dimethyl-5,7-diphenyl-1,2,3,4-tetrahydro-6H-cyclopenta[b]pyrazin-6-one (800 mg, 2.5 mmol), diiron nonacarbonyl (1.84 g, 5.0 mmol) and dry and degassed toluene (10 mL). The mixture was heated under reflux for 24 h. It was then cooled and transferred to a round-bottomed flask and washed several times with toluene (3 x 10 mL). The mixture was concentrated *in vacuo*. Purification by flash alumina chromatography surrounded by celite (0-1 % MeOH in CH_2Cl_2, 50 x 200 mm alumina) followed by precipitation (pentane/Et_2O) gave an orange-yellow solid (800 mg, 69%), mp 199-201 °C; R_f = 0.46 (eluent = 5% MeOH in CH_2Cl_2). **^{1}H NMR (500 MHz, $CDCl_3$)** δ_H: 2.38 (6H, s), 2.87-2.97 (2H, m), 3.39-3.50 (2H, m), 7.29-7.35 (2H, m), 7.36-7.42 (4H, m), 7.51-7.58 (4H, m); **^{13}C NMR (126 MHz, $CDCl_3$)** δ_C: 41.6, 50.2, 71.1, 114.6, 128.0, 128.4, 131.9, 132.4, 165.8, 210.3. Spectroscopic data in accordance with that stated in the literature.[6]

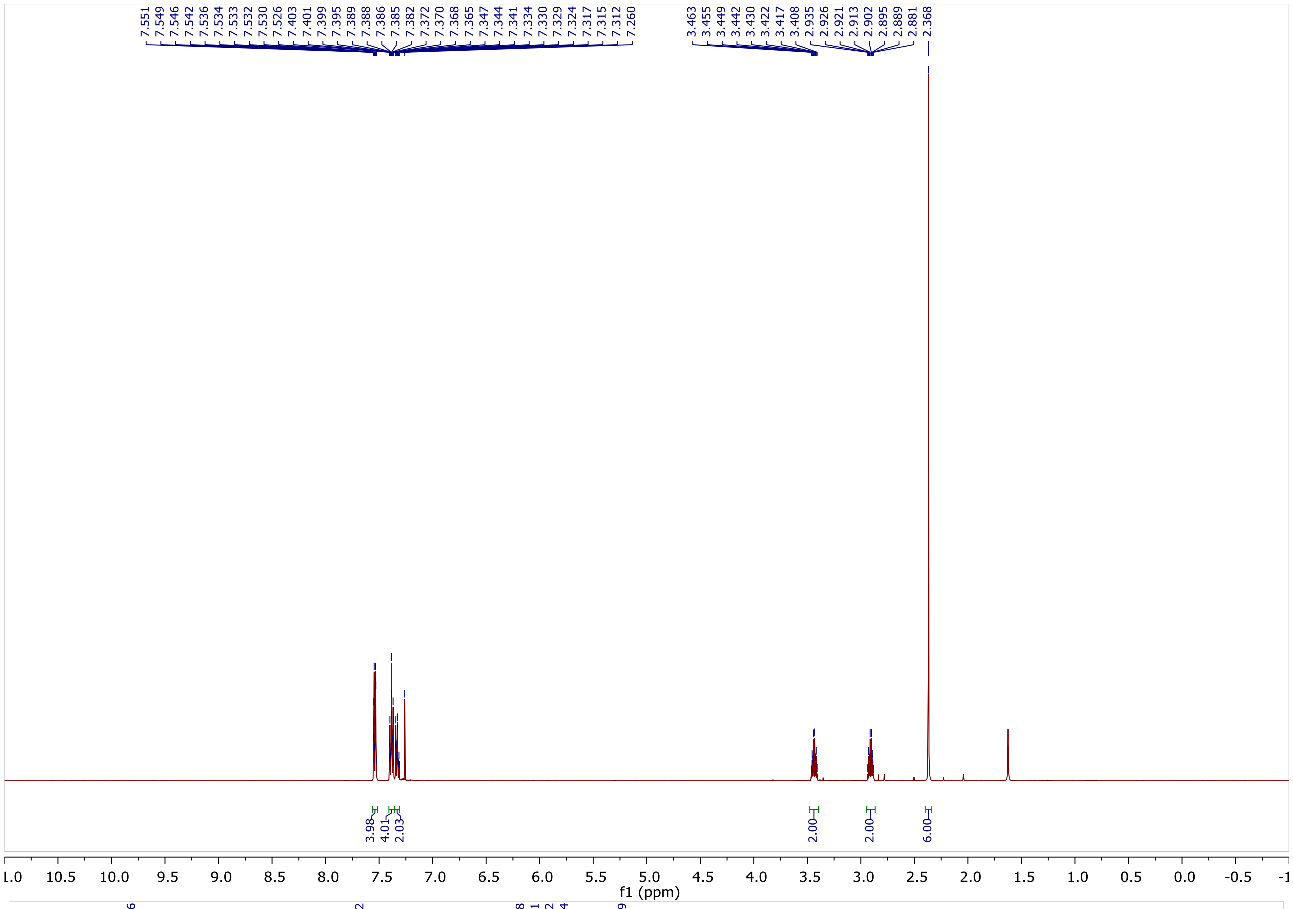

7.551
7.549
7.546
7.542
7.536
7.534
7.533
7.532
7.530
7.526
7.403
7.401
7.399
7.395
7.389
7.388
7.386
7.385
7.382
7.372
7.370
7.368
7.365
7.347
7.344
7.341
7.334
7.330
7.329
7.324
7.317
7.315
7.312
7.260
3.463
3.455
3.449
3.442
3.430
3.422
3.417
3.408
2.935
2.926
2.921
2.913
2.902
2.895
2.889
2.881
2.368
3.98
4.01
2.03
2.00
2.00
6.00
f1 (ppm)

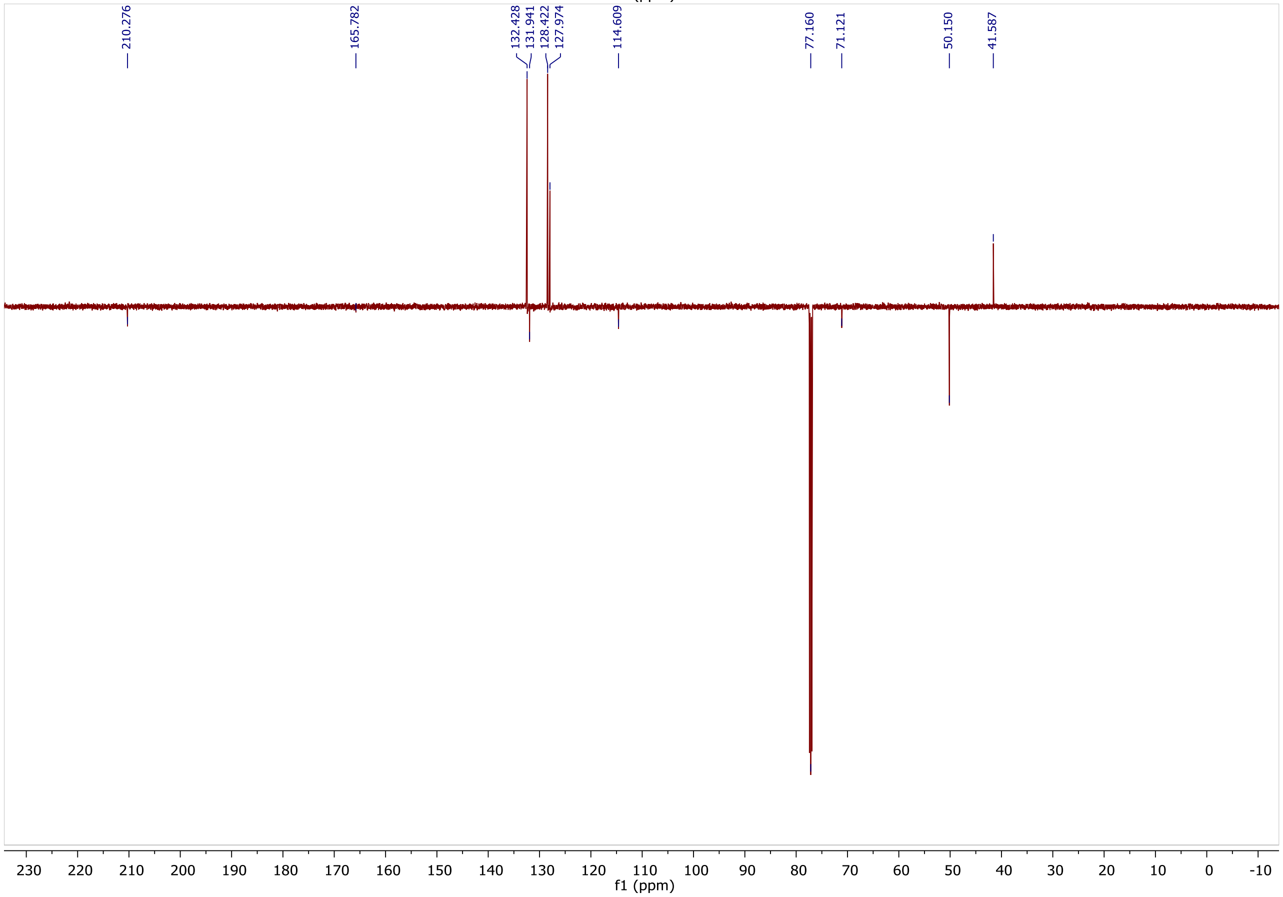

210.276
165.782
132.428
131.941
128.422
127.974
114.609
77.160
71.121
50.150
41.587
f1 (ppm)

7

2.2. Substrate synthesis

(4-vinylphenyl)methanol

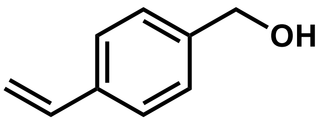

A 25 mL round-bottomed flask equipped with a magnetic stirrer bar was charged with 4-vinylbenzyl acetate (704.9 mg, 4.0 mmol), KOH (673.3 mg, 12.0 mmol), MeOH (3.6 mL) and H_2O (0.9 mL). The mixture was heated to 75 °C for 16 h. It was then cooled, diluted with EtOAc (10 mL) and transferred to a separatory funnel filled with EtOAc (15 mL). The organic phase was collected. The aqueous phase was washed with EtOAc (2 x 25 mL). The organics were combined, washed with sat. aq. NH_4Cl, dried over $MgSO_4$, filtered and concentrated *in vacuo.* Purification by flash silica chromatography (eluent = 30% Et_2O in hexanes, 30 x 150 mm silica) gave the title compound as a colourless oil (402 mg, 75%); R_f = 0.20 (eluent = 20% EtOAc in hexanes); **^{1}H NMR (500 MHz, $CDCl_3$)** δ_H: 1.72 (1H, br s), 4.68 (2H, s), 5.25 (1H, d, *J* 11.0), 5.76 (1H, d, *J* 18.0), 6.72 (1H, dd, *J* 17.5, 11.0), 7.33 (2H, d, *J* 7.0), 7.41 (2H, d, *J* 7.5); **^{13}C NMR (126 MHz, $CDCl_3$)** δ_C: 65.3, 114.0, 126.5, 127.3, 136.6, 137.2, 140.5. Spectroscopic data in accordance with the literature.[7]

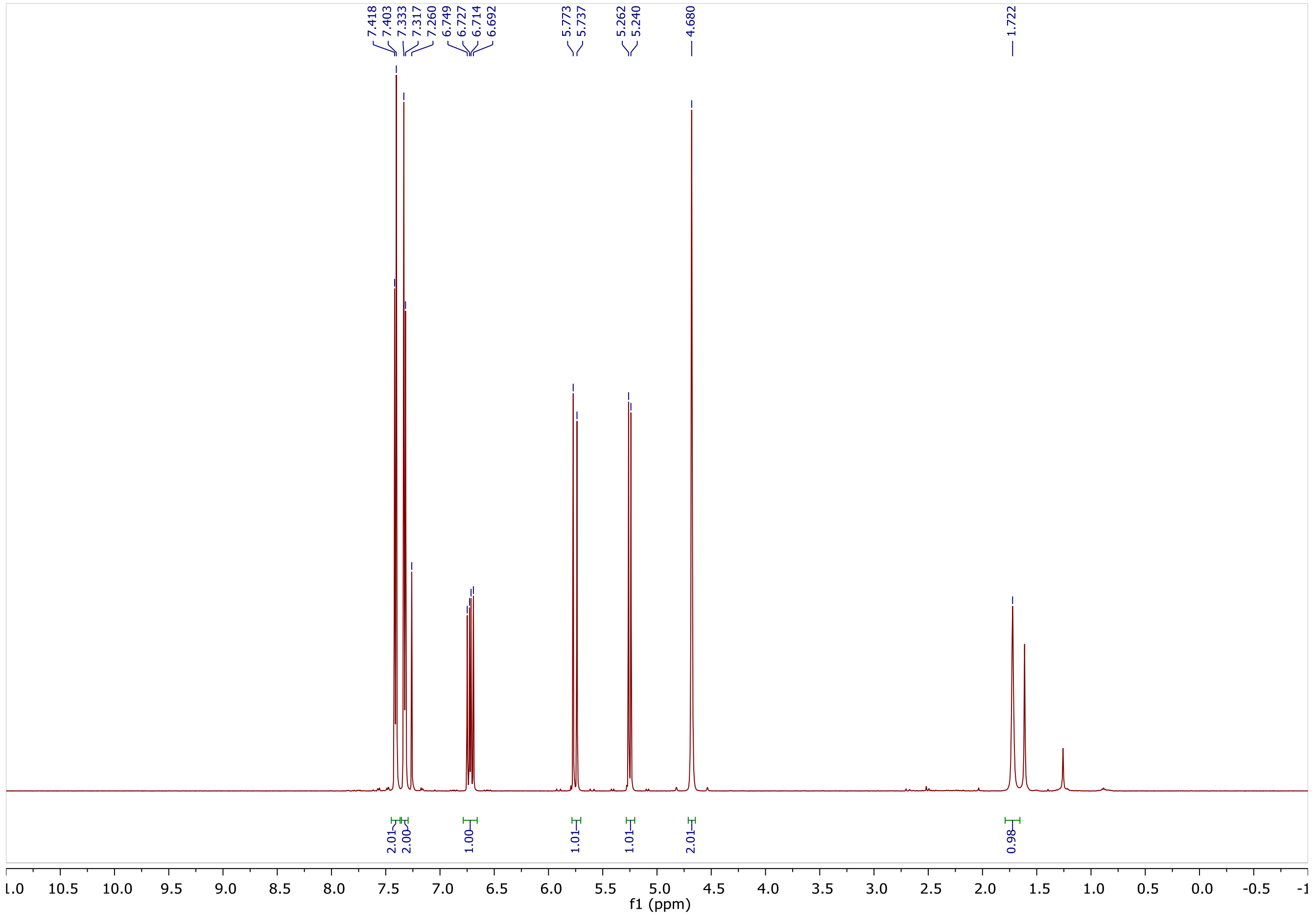

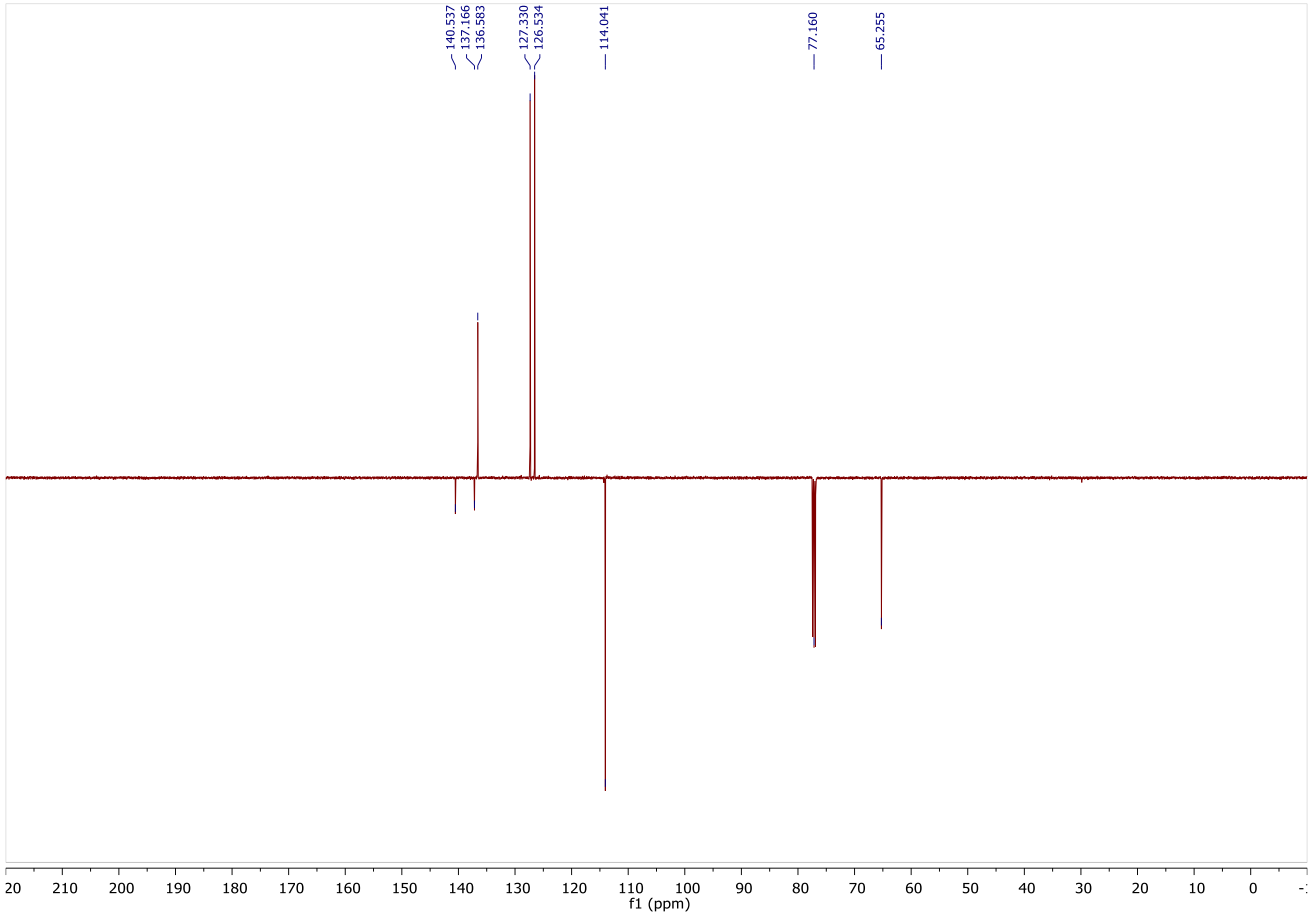

1,3-dicyclohexylpyrimidine-2,4,6(1H,3H,5H)-trione

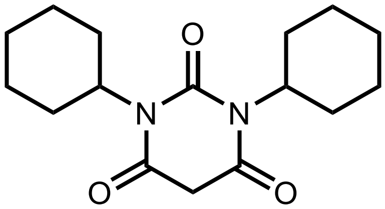

A 100 mL round-bottomed flask equipped with a magnetic stirrer bar was charged with malonic acid (2.08 g, 20 mmol) and THF (25 mL). The solution was cooled to 0 °C followed by the addition of a solution of *N,N′*-dicyclohexylcarbodiimide (8.25 g, 40 mmol) in THF (25 mL) over a period of 30 min. The mixture was left to warm up to rt and was left stirring for a total of 3 h. The urea was filtered off and the filtrate was concentrated *in vacuo*. Purification by recrystallisation gave the title compound as an off-white solid (3.18 g, 54%); mp 200-203 °C (ethanol) (Lit. 201-203 °C),[8] R_f = 0.44 (eluent = 20% EtOAc in hexanes); **^{1}H NMR (500 MHz, $CDCl_3$)** δ_H: 1.14-1.27 (2H, m), 1.34 (4H, qt, *J* 13.0, 3.5), 1.53-1.70 (6H, m), 1.77-1.88 (4H, m), 2.24 (4H, qd, *J* 12.5, 3.5), 3.59 (2H, s), 4.58 (2H, tt, *J* 12.5, 3.5); **^{13}C NMR (126 MHz, $CDCl_3$)** δ_C: 25.3, 26.5, 29.3, 41.1, 55.5, 151.4, 165.2. Spectroscopic data in accordance with the literature.[9]

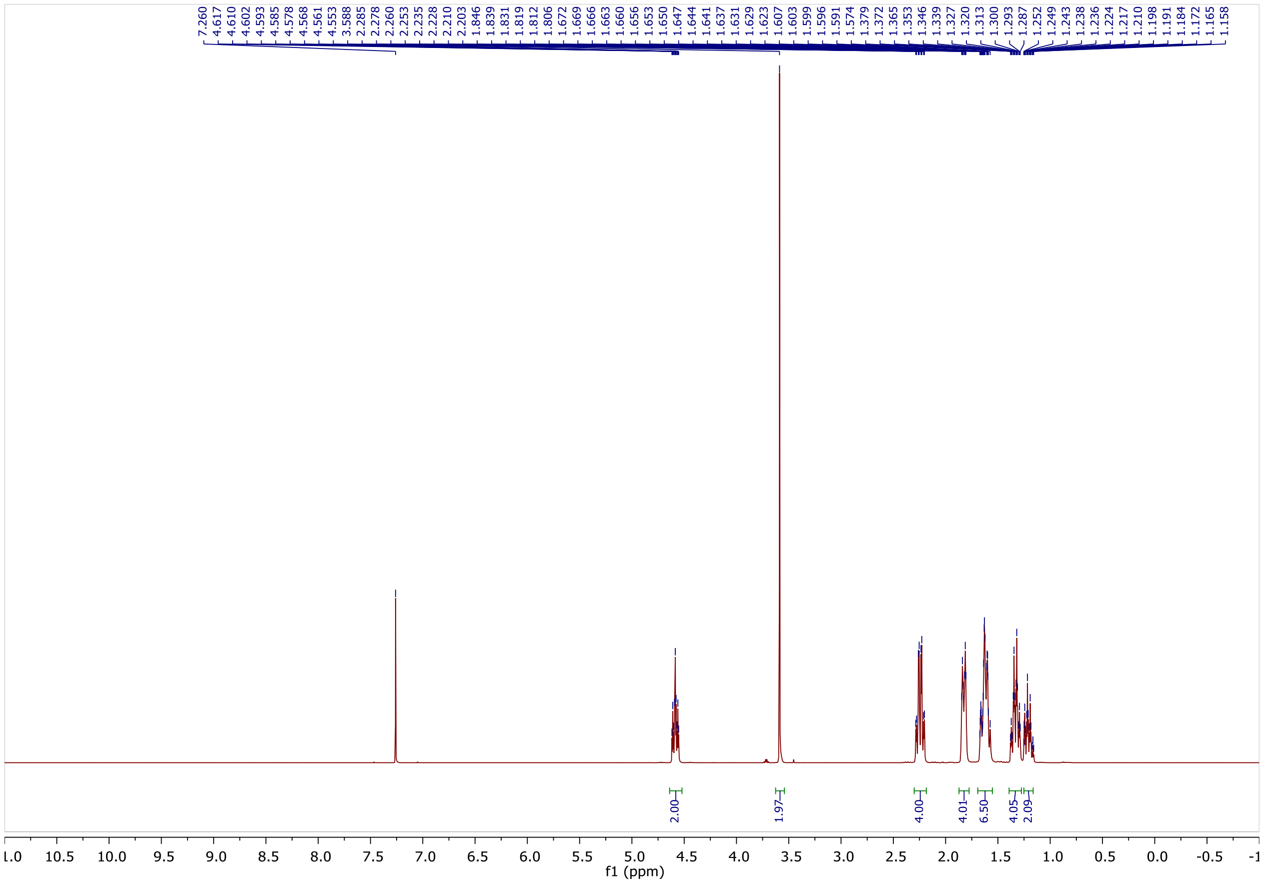

7.260
4.617 4.610 4.602 4.593 4.585 4.578 4.568 4.561 4.553 3.588 2.285 2.278 2.260 2.253 2.235 2.228 2.210 2.203 1.846 1.839 1.831 1.819 1.812 1.806 1.672 1.669 1.666 1.663 1.660 1.656 1.653 1.650 1.647 1.644 1.641 1.637 1.631 1.629 1.623 1.607 1.603 1.599 1.596 1.591 1.574 1.379 1.372 1.365 1.353 1.346 1.339 1.327 1.320 1.313 1.300 1.293 1.287 1.252 1.249 1.243 1.238 1.236 1.224 1.217 1.210 1.198 1.191 1.184 1.172 1.165 1.158
2.00
1.97
4.00
4.01
6.50
4.05
2.09
f1 (ppm)

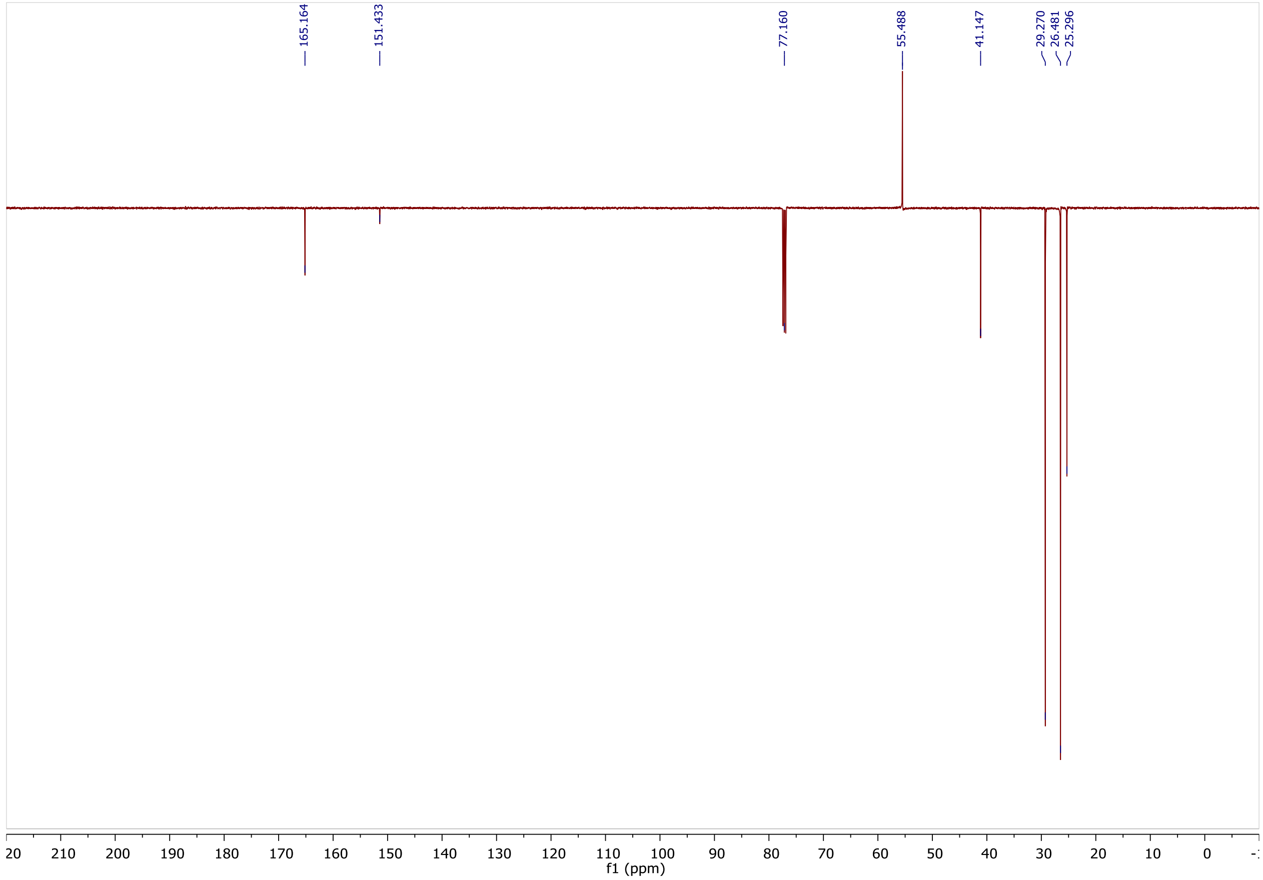

165.164
151.433
77.160
55.488
41.147
29.270
26.481
25.296
f1 (ppm)

1,3-dibenzylpyrimidine-2,4,6(1H,3H,5H)-trione

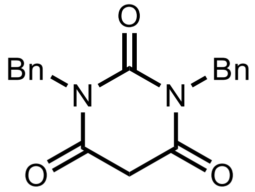

A 50 mL round-bottomed flask equipped with a magnetic stirrer bar was charged with 1,3-dibenzyl urea[10] (1.2 g, 5.0 mmol), $CHCl_3$ (15 mL) and malonyl chloride (580 µL, 846 mg, 6.0 mmol). The mixture was heated to reflux for 6 h. It was then cooled and concentrated *in vacuo*. Purification by flash silica chromatography (eluent = 20% EtOAc in hexanes, 35 x 140 mm silica) gave the title compound as a yellow solid (1.04 g, 68%), mp 142-145 °C (Lit. 146-147 °C),[11] R_f = 0.27 (eluent = 40% EtOAc in hexanes); **^{1}H NMR (500 MHz, CDCl₃)** δ_H: 3.68 (2H, s), 5.04 (4H, s), 7.27-7.35 (6H, m), 7.38-7.46 (4H, m); **^{13}C NMR (126 MHz, CDCl₃)** δ_C: 39.9, 45.3, 128.2, 128.7, 129.3, 136.1, 151.7, 164.2. Spectroscopic data in accordance with the literature.[11]

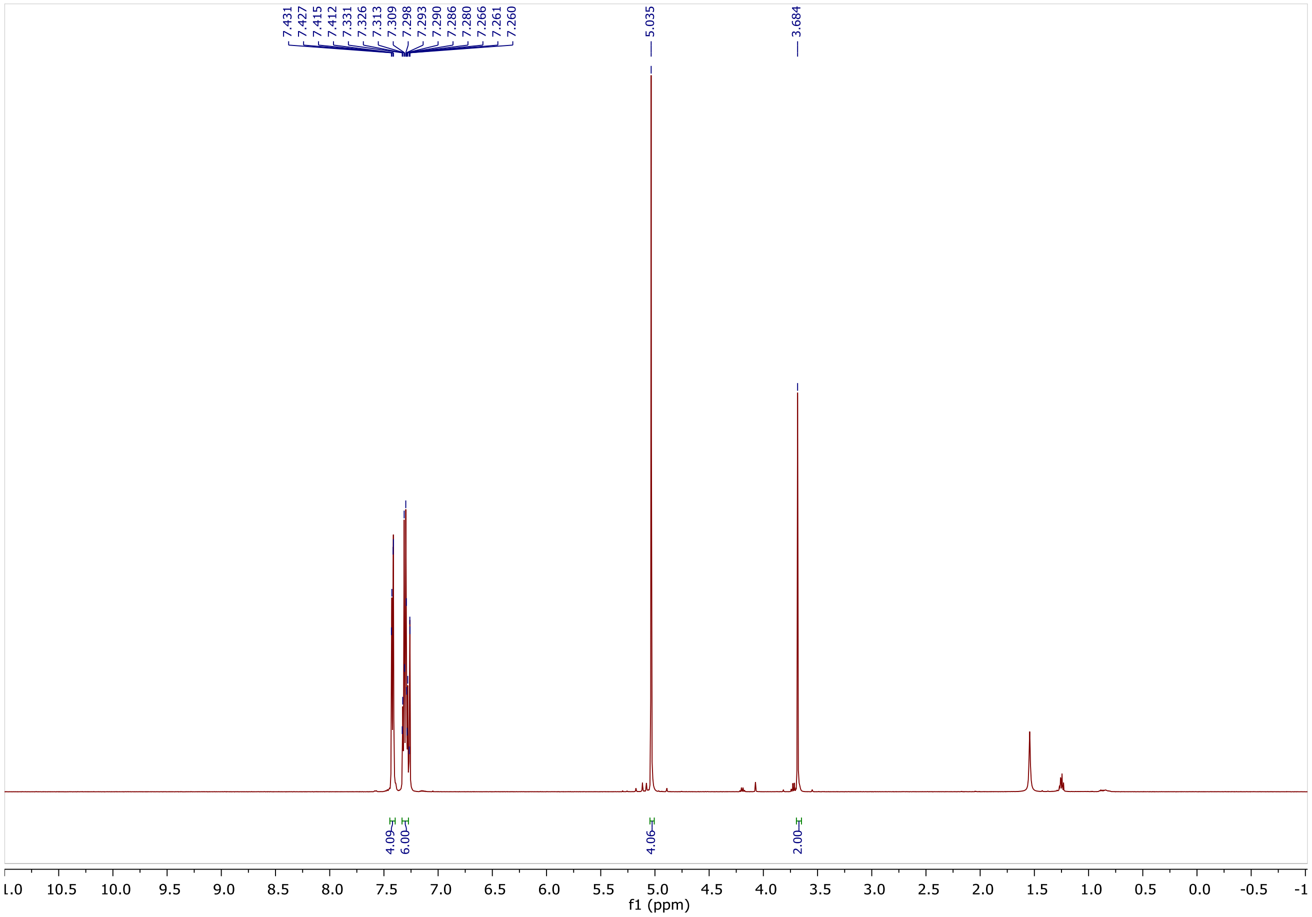

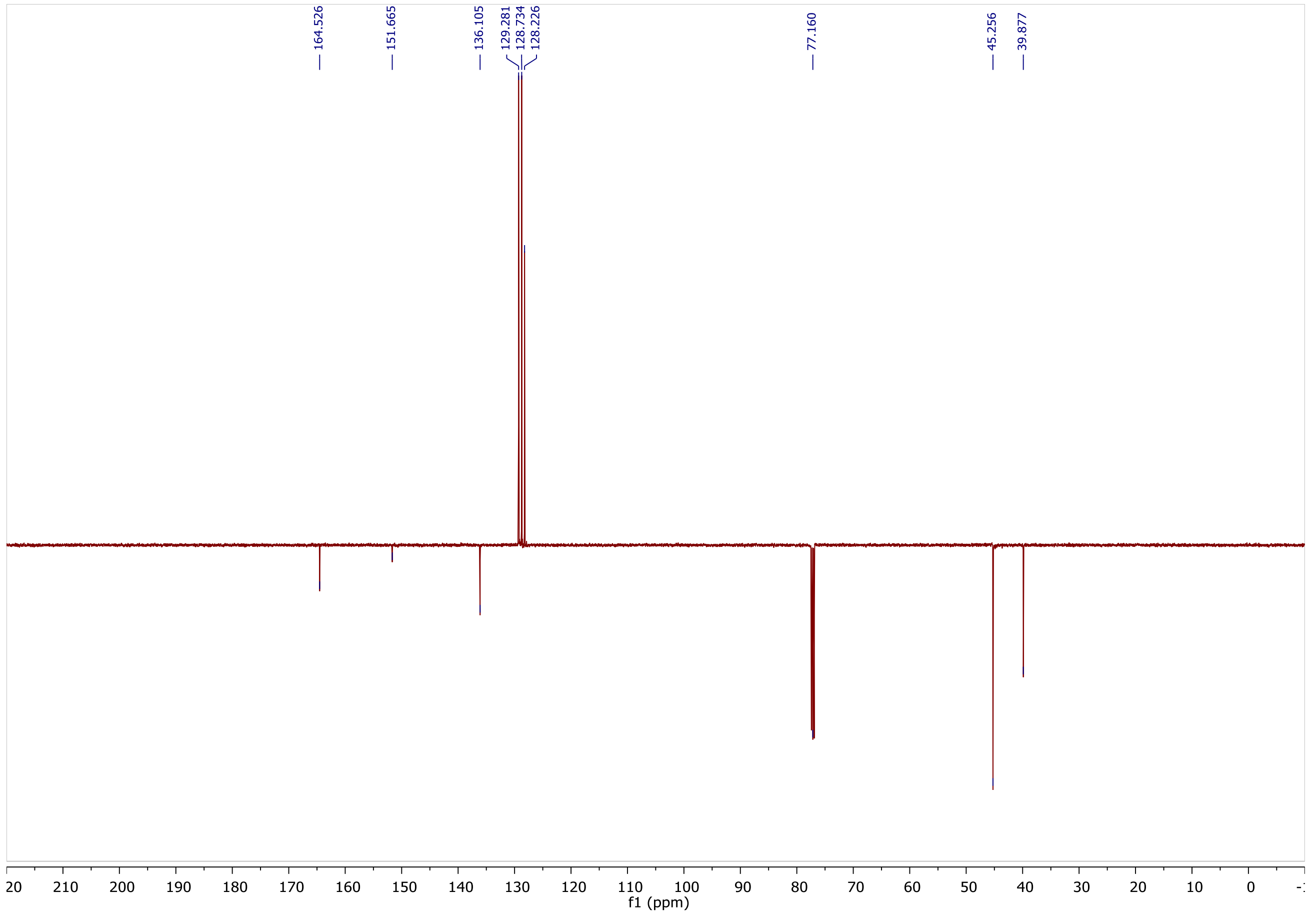

2.3. Optimization of Fe-catalyzed oxindole *C*(3)-benzylation

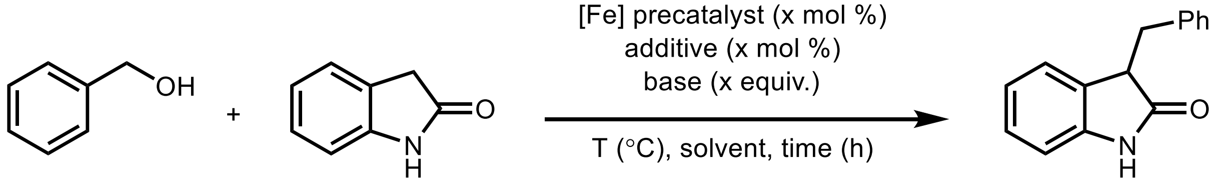

A 10 mL microwave vial equipped with a stirrer bar was charged with oxindole (133 mg, 1.0 mmol), base (x equiv.), additive (x mol %) and precatalyst (x mol %) The vial was sealed with a cap and was placed under vacuum. After 5 minutes it was flushed with nitrogen and the cycle repeated three times. Under nitrogen the vial was then charged with xylene (2 mL) and benzyl alcohol (124 µL, 130 mg, 1.2 mmol, 1.2 equiv.). The mixture was left to react at 150 °C for 24 hours. It was then cooled, followed by the addition of mesitylene (139 µL, 120 mg, 1.0 mmol), H_2O (2 mL) and EtOAc (2 mL). In some cases, brine (1 mL) was added to aid layer separation. The mixture was stirred for 5 min, left to settle for a further 5 min, cap removed and the top layer was sampled and analysed using ^1H NMR.

Entry	Cat. loading (mol %)	Additive (mol %)	Base (equiv.)	Solvent (Conc.)	T (°C)	2 (%)	4 (%)[a]
1	**[Fe 3] (2)**	PPh_3 (4)	K_2CO_3 (0.5)	xylene (0.5 M)	150	0	**97 (90)[b]**
2	**-**	PPh_3 (4)	K_2CO_3 (0.5)	xylene (0.5 M)	150	67	<2
3	[Fe 3] (2)	PPh_3 (4)	**-**	xylene (0.5 M)	150	35	26
4	**[Fe 5] (2)**	-	K_2CO_3 (0.5)	xylene (0.5 M)	150	0	95
5	**[Fe 6] (2)**	PPh_3 (4)	K_2CO_3 (0.5)	xylene (0.5 M)	150	32	18
6	**[Fe 7] (2)**	PPh_3 (4)	K_2CO_3 (0.5)	xylene (0.5 M)	150	51	5
7	**[Fe 8] (2)**	PPh_3 (4)	K_2CO_3 (0.5)	xylene (0.5 M)	150	53	5
8	**[Fe 9] (2)**	PPh_3 (4)	K_2CO_3 (0.5)	xylene (0.5 M)	150	45	5
9	**[Fe 10] (2)**	PPh_3 (4)	K_2CO_3 (0.5)	xylene (0.5 M)	150	50	5
10	[Fe 3] (2)	**-**	K_2CO_3 (0.5)	xylene (0.5 M)	150	0	90
11	[Fe 3] (2)	**PPh_3 (2)**	K_2CO_3 (0.5)	xylene (0.5 M)	150	0	95
12	[Fe 3] (2)	**Me_3NO (4)**	K_2CO_3 (0.5)	xylene (0.5 M)	150	0	92
13	[Fe 3] (2)	PPh_3 (4)	**KOH (0.5)**	xylene (0.5 M)	150	0	96
14	[Fe 3] (2)	PPh_3 (4)	**NaOH (0.5)**	xylene (0.5 M)	150	0	87
15	[Fe 3] (2)	PPh_3 (4)	**KO^tBu (0.5)**	xylene (0.5 M)	150	0	83
16	[Fe 3] (2)	PPh_3 (4)	**Cs_2CO_3(0.5)**	xylene (0.5 M)	150	0	85
17	[Fe 3] (2)	PPh_3 (4)	**LiO^tBu (1)**	xylene (0.5 M)	150	0	92
18	[Fe 3] (2)	PPh_3 (4)	**K_2CO_3 (0.1)**	xylene (0.5 M)	150	0	88
19	[Fe 3] (2)	PPh_3 (4)	K_2CO_3 (0.5)	**Xylene (0.25 M)**	150	0	83
20	[Fe 3] (2)	PPh_3 (4)	K_2CO_3 (0.5)	**Xylene (1 M))**	150	0	93
21	[Fe 3] (2)	PPh_3 (4)	K_2CO_3 (0.5)	**dioxane (0.5 M)**	150	0	48
22	[Fe 3] (2)	PPh_3 (4)	K_2CO_3 (0.5)	**THF (0.5 M)**	150	41	40
23	[Fe 3] (2)	PPh_3 (4)	K_2CO_3 (0.5)	**^{t}amyl alcohol (0.5 M)**	150	31	50
24	[Fe 3] (2)	PPh_3 (4)	K_2CO_3 (0.5)	**toluene (0.5 M)**	150	9	91
25	[Fe 3] (2)	PPh_3 (4)	K_2CO_3 (0.5)	Xylene (0.5 M)	**130**	0	86
26	[Fe 3] (2)	PPh_3 (4)	K_2CO_3 (0.5)	Xylene (0.5 M)	**110**	26	51
27	[Fe 3] (2)	PPh_3 (4)	K_2CO_3 (0.5)	Xylene (0.5 M)	**90**	74	0
28	[Fe 3] (2)	PPh_3 (2)	K_2CO_3 (0.5)	xylene (0.5 M)	150	0	92[c]
29	**[Fe 3] (1)**	PPh_3 (2)	K_2CO_3 (0.5)	Xylene (0.5 M)	150	12	73

[a]Yield after 24 h as determined by ^{1}H NMR analysis of the crude reaction mixture with 1,3,5-trimethylbenzene as the internal standard. [b]Isolated yield given in parentheses. [c]6 h reaction time.

2.4. Substrate scope

2.4.1. General procedure 1

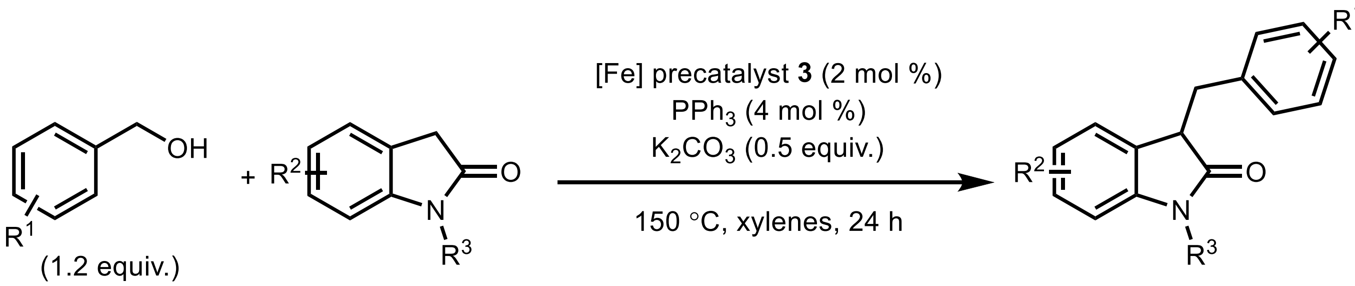

A 10 mL microwave vial equipped with a stirrer bar was charged with substituted oxindole (1.0 mmol), K_2CO_3 (69.1 mg, 0.5 mmol, 0.5 equiv.), PPh_3 (10.5 mg, 0.04 mmol, 4 mol %) and [Fe] precatalyst **3** (9.1 mg, 0.02 mmol, 2 mol %). The vial was sealed with a cap and was placed under vacuum. After 5 minutes it was flushed with nitrogen and the cycle repeated three times. Under nitrogen the vial was then charged with xylene (2 mL) and substituted benzyl alcohol (1.2 mmol, 1.2 equiv.). The mixture was left to react at 150 °C for 24 hours. It was then cooled, washed with EtOAc (25 mL) and transferred to a separatory funnel filled with brine (25 mL). The organic layer was collected and the aqueous phase washed with EtOAc (2 x 25 mL). The organics were combined, dried over $MgSO_4$, filtered and concentrated *in vacuo*.

2.4.2. General procedure 2

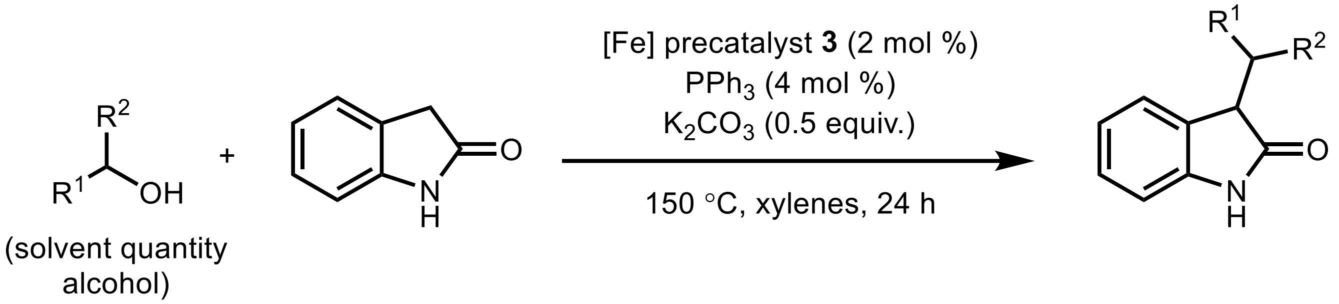

A 10 mL microwave vial equipped with a stirrer bar was charged with oxindole (133 mg, 1.0 mmol), K_2CO_3 (69.1 mg, 0.5 mmol, 0.5 equiv.), PPh_3 (10.5 mg, 0.04 mmol, 4 mol %) and [Fe] precatalyst **3** (9.1 mg, 0.02 mmol, 2 mol %). The vial was sealed with a cap and was placed under vacuum. After 5 minutes it was flushed with nitrogen and the cycle repeated three times. Under nitrogen the vial was then charged with alcohol (2 mL). The mixture was left to react at 150 °C for 24 hours. It was then cooled, washed with EtOAc (25 mL) and transferred to a separatory funnel filled with brine (25 mL). The organic layer was collected and the aqueous phase washed with EtOAc (2 x 25 mL). The organics were combined, dried over $MgSO_4$, filtered and concentrated *in vacuo*.

2.4.3. General procedure 3

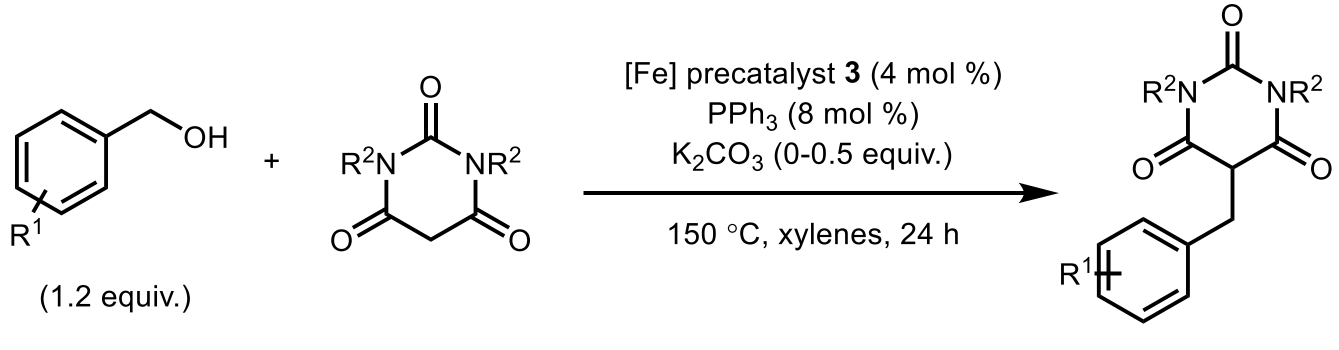

A 10 mL microwave vial equipped with a stirrer bar was charged with barbituric acid (1.0 mmol), K_2CO_3 PPh_3 (21.0 mg, 0.08 mmol, 8 mol %) and [Fe] precatalyst **3** (18.3 mg, 0.04 mmol, 4 mol %). The vial was sealed with a cap and was placed under vacuum. After 5 minutes it was flushed with nitrogen and the cycle repeated three times. Under nitrogen the vial was then charged with alcohol (1.2 mmol) and xylene (2 mL). The mixture was left to react at 150 °C for 24 hours. It was then cooled, washed with EtOAc (25 mL) and transferred to a separatory funnel filled with brine (25 mL). The organic layer was collected and

the aqueous phase washed with EtOAc (2 x 25 mL). The organics were combined, dried over $MgSO_4$, filtered and concentrated *in vacuo*.

3-benzylindolin-2-one

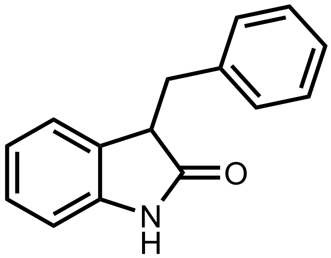

4

The title compound was prepared according to general procedure 1 using benzyl alcohol (124 µL, 130 mg, 1.2 mmol) and oxindole (133 mg, 1.0 mmol). Purification by flash silica chromatography (eluent = 25% EtOAc in hexanes, 30 x 150 mm silica) gave the title compound as a yellow solid (200 mg, 90%). mp 128-132 °C (Lit. 129-130°C);[12] R_f = 0.20 (eluent = 20% EtOAc in hexanes); **^{1}H NMR (400 MHz, $CDCl_3$)** δ_H: 2.94 (1H, dd, *J* 13.6, 9.2), 3.50 (1H, dd, *J* 13.6, 4.4), 3.75 (1H, dd, *J* 9.2, 4.8), 6.75 (1H, d, *J* 7.2), 6.83 (1H, d, *J* 7.6), 6.90 (1H, t, *J* 8.0), 7.13-7.31 (6H, m), 8.24 (1H, br s); **^{13}C NMR (101 MHz, $CDCl_3$)** δ_C: 36.8, 47.6, 109.8, 122.2, 125.0, 126.8, 125.1, 128.5, 129.1, 129.6, 138.0, 141.5, 179.5. Spectroscopic data in accordance with the literature.[12]

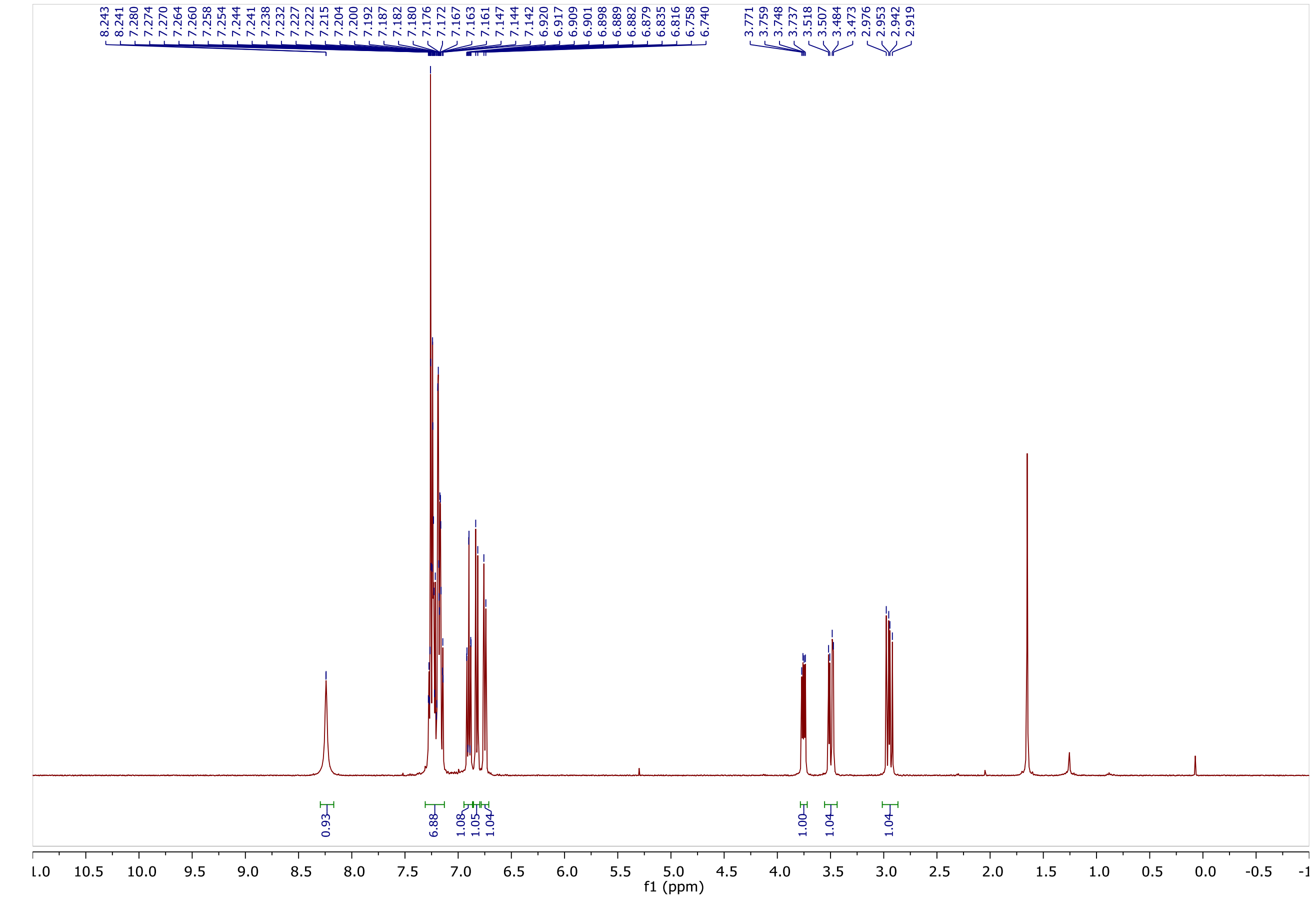

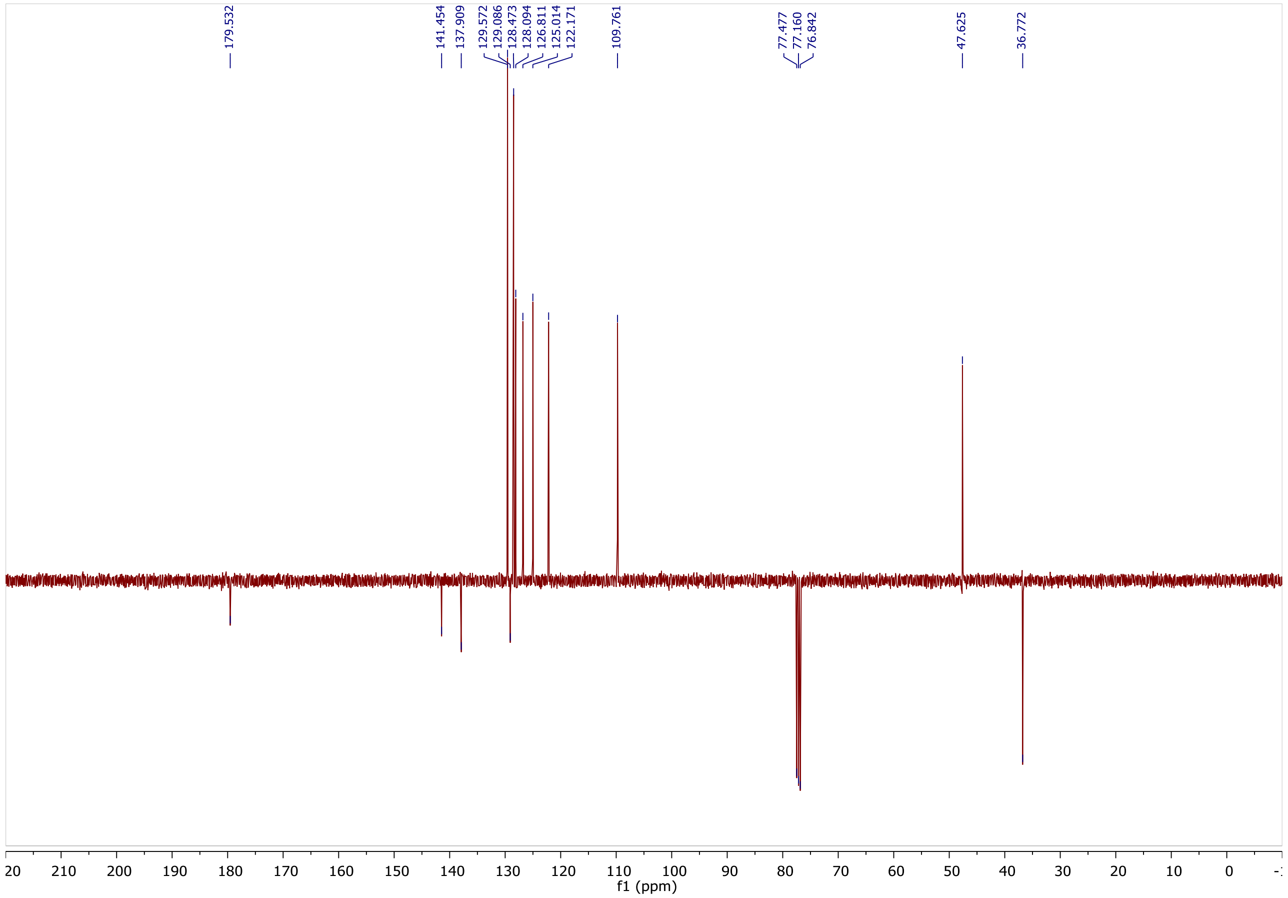

3-(4-methylbenzyl)indolin-2-one

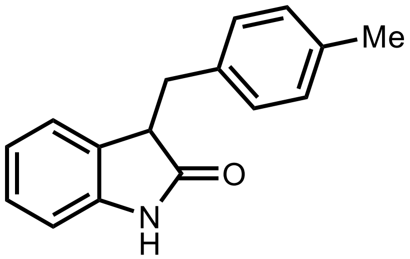

11

The title compound was prepared according to general procedure 1 using 4-methylbenzyl alcohol (147 mg, 1.2 mmol) and oxindole (133 mg, 1.0 mmol). Purification by flash silica chromatography (eluent = 15% EtOAc in hexanes, 30 x 150 mm silica) gave the title compound as a pink solid (207 mg, 87%). mp 148-150 °C, (149-150°C);[12] R_f = 0.30 (eluent = 15% EtOAc in hexanes); **^{1}H NMR (400 MHz, $CDCl_3$)** δ_H: 2.31 (3H, s), 2.91 (1H, dd, *J* 14.0, 9.2), 3.45 (1H, dd, *J* 13.6, 4.4), 3.73 (1H, dd, *J* 9.2, 4.4), 6.80 (2H, dd, *J* 17.6, 8.0), 6.91 (1H, dt, *J* 7.6), 7.06 (4H, s), 7.16 (1H, t, *J* 7.6), 8.15 (1H, br s); **^{13}C NMR (101 MHz, $CDCl_3$)** δ_C: 21.2, 36.3, 47.6, 109.7, 122.2, 125.1, 128.0, 129.1, 129.2, 129.4, 134.8, 136.3, 141.4, 179.4. Spectroscopic data in accordance with the literature.[12]

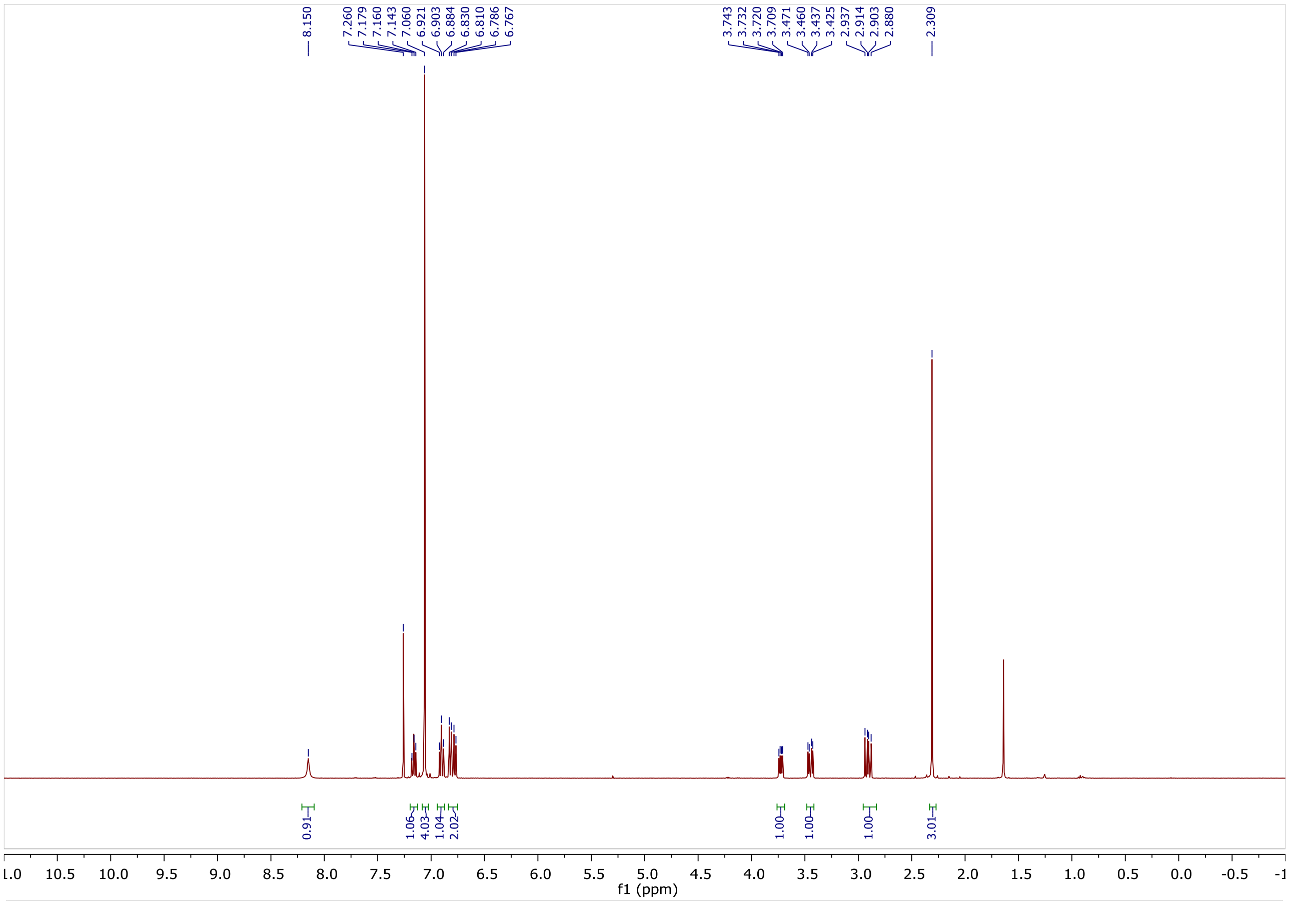
8.150
7.260
7.179
7.160
7.143
7.060
6.921
6.903
6.884
6.830
6.810
6.786
6.767
3.743
3.732
3.720
3.709
3.471
3.460
3.437
3.425
2.937
2.914
2.903
2.880
2.309
0.91
1.06
4.03
1.04
2.02
1.00
1.00
1.00
3.01
f1 (ppm)

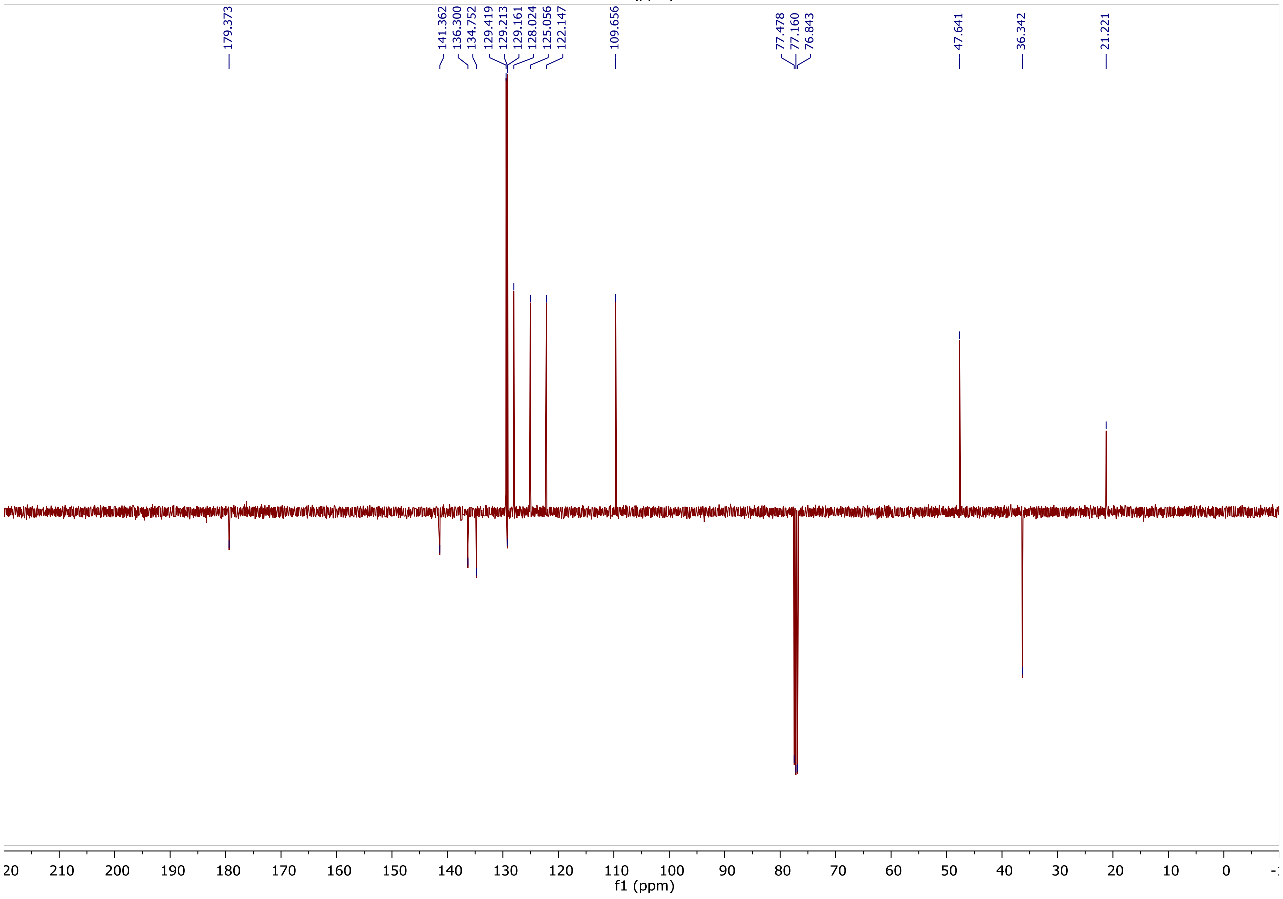
179.373
141.362
136.300
134.752
129.419
129.213
129.161
128.024
125.056
122.147
109.656
77.478
77.160
76.843
47.641
36.342
21.221
f1 (ppm)

3-(3-methylbenzyl)indolin-2-one

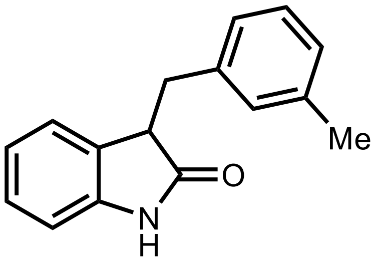

12

The title compound was prepared according to general procedure 1 using 3-methylbenzyl alcohol (145 µL, 147 mg, 1.2 mmol) and oxindole (133 mg, 1.0 mmol). Purification by flash silica chromatography (eluent = 20% EtOAc in petroleum ether, 30 x 150 mm silica) gave the title compound as an off-white solid (186 mg, 78%). mp 98-100 °C; R_f = 0.30 (eluent = 30% EtOAc in petroleum ether); ν_{max} /cm^{-1} (film): 3132, 3065, 3024, 2914, 2820, 1697, 1613, 1468, 1337, 1300, 1227, 748, 694, 667, 617, 557; **^{1}H NMR (400 MHz, $CDCl_3$)** δ_H: 2.30 (3H, s), 2.88 (1H, dd, *J* 13.6, 9.6), 3.46 (1H, dd, *J* 13.6, 4.8), 3.73 (1H, dd, *J* 9.2, 4.4), 6.74 (1H, d, *J* 7.6), 6.81 (1H, d, *J* 8.0), 6.89 (1H, dt, *J* 14.4, 7.2), 6.97 (1H, d, *J* 8.0), 7.03 (2H, d, *J* 7.6), 7.09-7.21 (2H, m), 7.87 (1H, br s); **^{13}C NMR (101 MHz, $CDCl_3$)** δ_C: 21.5, 36.8, 47.7, 109.8, 122.1, 125.0, 126.5, 127.5, 128.0, 128.3, 129.3, 130.3, 137.9, 138.1, 141.5, 179.9; HRMS (**NSI$^+$**) calculated for $[C_{16}H_{16}NO]^+$ $(M + H)^+$ m/z: 238.1226, found 238.1229 (+1.1 ppm).

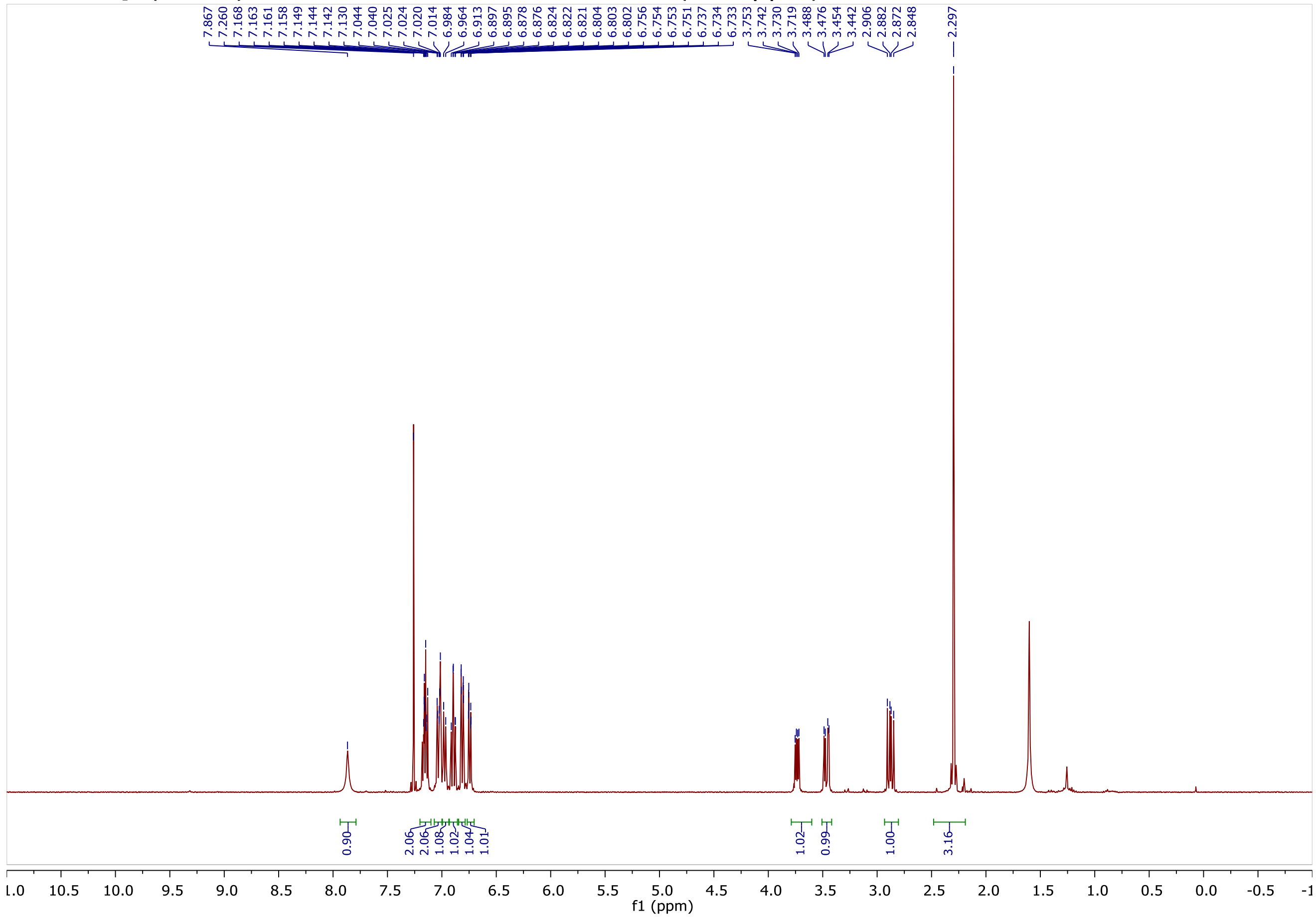

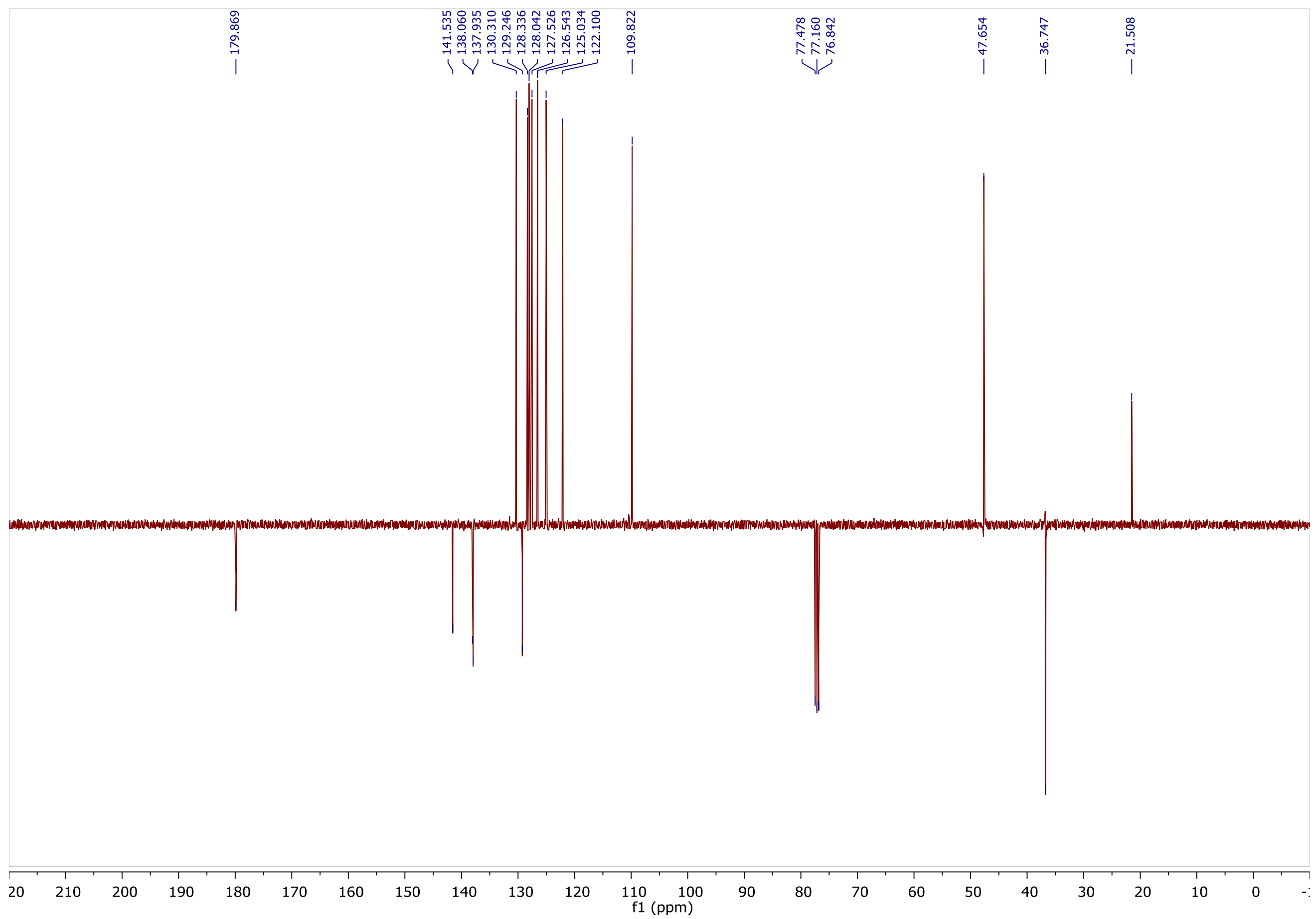

3-(2-methylbenzyl)indolin-2-one

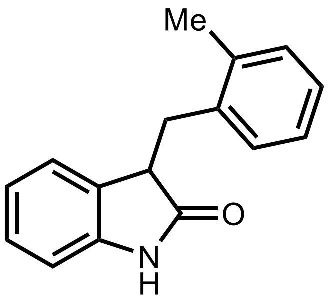

13

The title compound was prepared according to general procedure 1 using 2-methylbenzyl alcohol (145 µL, 147 mg, 1.2 mmol) and oxindole (133 mg, 1.0 mmol). Purification by flash silica chromatography (eluent = 20% EtOAc in hexanes, 30 x 150 mm silica) gave the title compound as a yellow solid (213 mg, 90%). mp 105-106 °C (Lit. 106-108 °C);[12] R_f = 0.33 (eluent = 20% EtOAc in hexanes); **^{1}H NMR (400 MHz, $CDCl_3$)** δ_H: 2.30 (3H, s) 2.81 (1H, dd, *J* 13.6, 10.8), 3.52 (1H, dd, *J* 14.0, 4.8), 3.71 (1H, dd, *J* 10.8, 4.8), 6.54 (1H, d, *J* 7.2), 6.80-6.91 (2H, m), 7.13-7.22 (5H, m), 8.63 (1H, br s); **^{13}C NMR (101 MHz, $CDCl_3$)** δ_C: 19.8, 34.4, 46.3, 109.8, 122.2, 125.2, 126.1, 127.0, 128.1, 129.3, 130.2, 130.7, 136.7, 136.9, 141.4, 179.8. Spectroscopic data in accordance with the literature.[12]

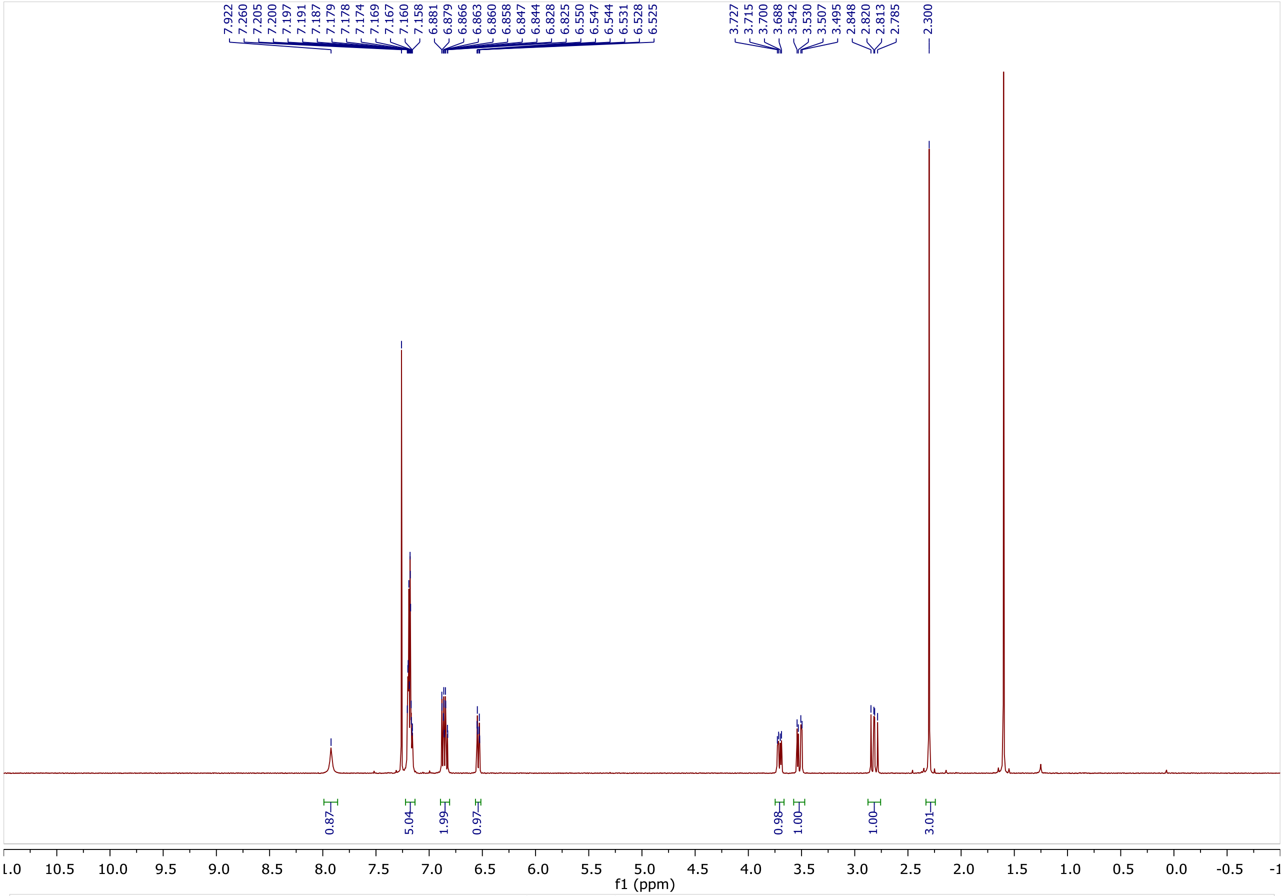
7.922
7.260
7.205
7.200
7.197
7.191
7.187
7.179
7.178
7.174
7.169
7.167
7.160
7.158
6.881
6.879
6.866
6.863
6.860
6.858
6.847
6.844
6.828
6.825
6.550
6.547
6.544
6.531
6.528
6.525
3.727
3.715
3.700
3.688
3.542
3.530
3.507
3.495
2.848
2.820
2.813
2.785
2.300
0.87
5.04
1.99
0.97
0.98
1.00
1.00
3.01
f1 (ppm)

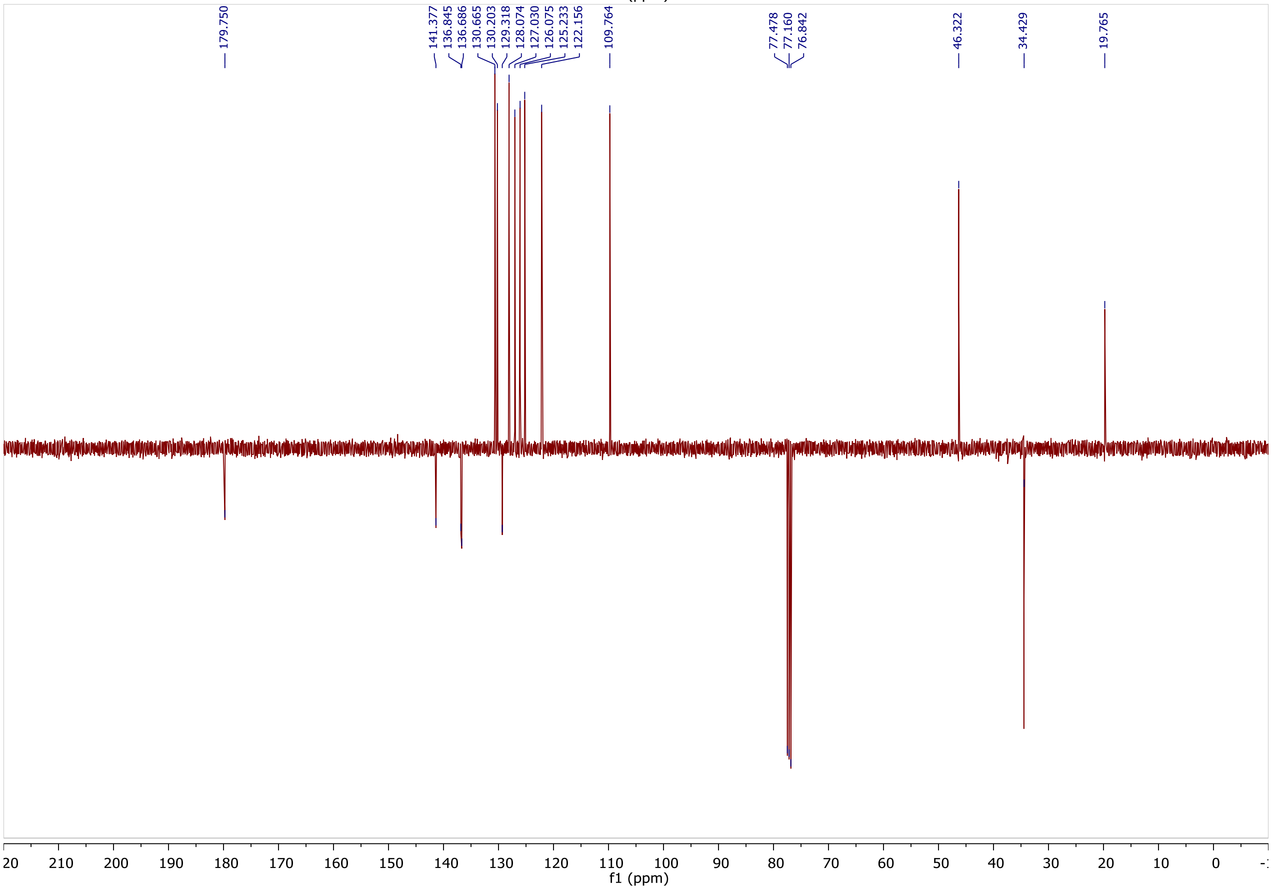
179.750
141.377
136.845
136.686
130.665
130.203
129.318
128.074
127.030
126.075
125.233
122.156
109.764
77.478
77.160
76.842
46.322
34.429
19.765
f1 (ppm)

3-(naphthalen-1-ylmethyl)indolin-2-one

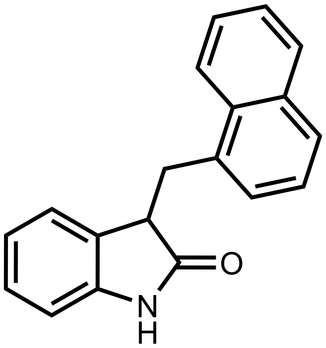

14

The title compound was prepared according to general procedure 1 using 1-naphthalenemethanol (190 mg, 1.2 mmol) and oxindole (133 mg, 1.0 mmol). Purification by flash silica chromatography (eluent = 20% EtOAc in hexanes, 30 x 150 mm silica) gave the title compound as a pale brown solid (248 mg, 91%). mp 135-138 °C; R_f = 0.33 (eluent = 20 % EtOAc in hexanes); **^{1}H NMR (400 MHz, $CDCl_3$)** δ_H: 3.06 (1H, dd, *J* 14.0, 11.2), 3.89 (1H, dd, *J* 11.2, 4.0), 4.13 (1H, dd, *J* 14.0, 4.0), 6.81 (1H, d, *J* 7.6), 6.81 (1H, t, *J* 7.2), 6.88 (1H, d, *J* 7.6), 7.16 (1H, t, *J* 8.0), 7.31 (1H, d, *J* 6.8), 7.44 (1H, t, *J* 7.2), 7.49-7.62 (2H, m), 7.84 (2H, d, *J* 7.6), 7.92 (1H, d, *J* 8.0), 8.25 (1H, d, *J* 9.6); **^{13}C NMR (101 MHz, $CDCl_3$)** δ_C: 35.1, 46.4, 109.6, 121.1, 123.8, 125.3, 125.6, 126.0, 126.5, 127.9, 128.0, 128.1, 129.1, 129.5, 131.8, 134.2, 134.4, 141.2, 179.3. Spectroscopic data in accordance with the literature.[12]

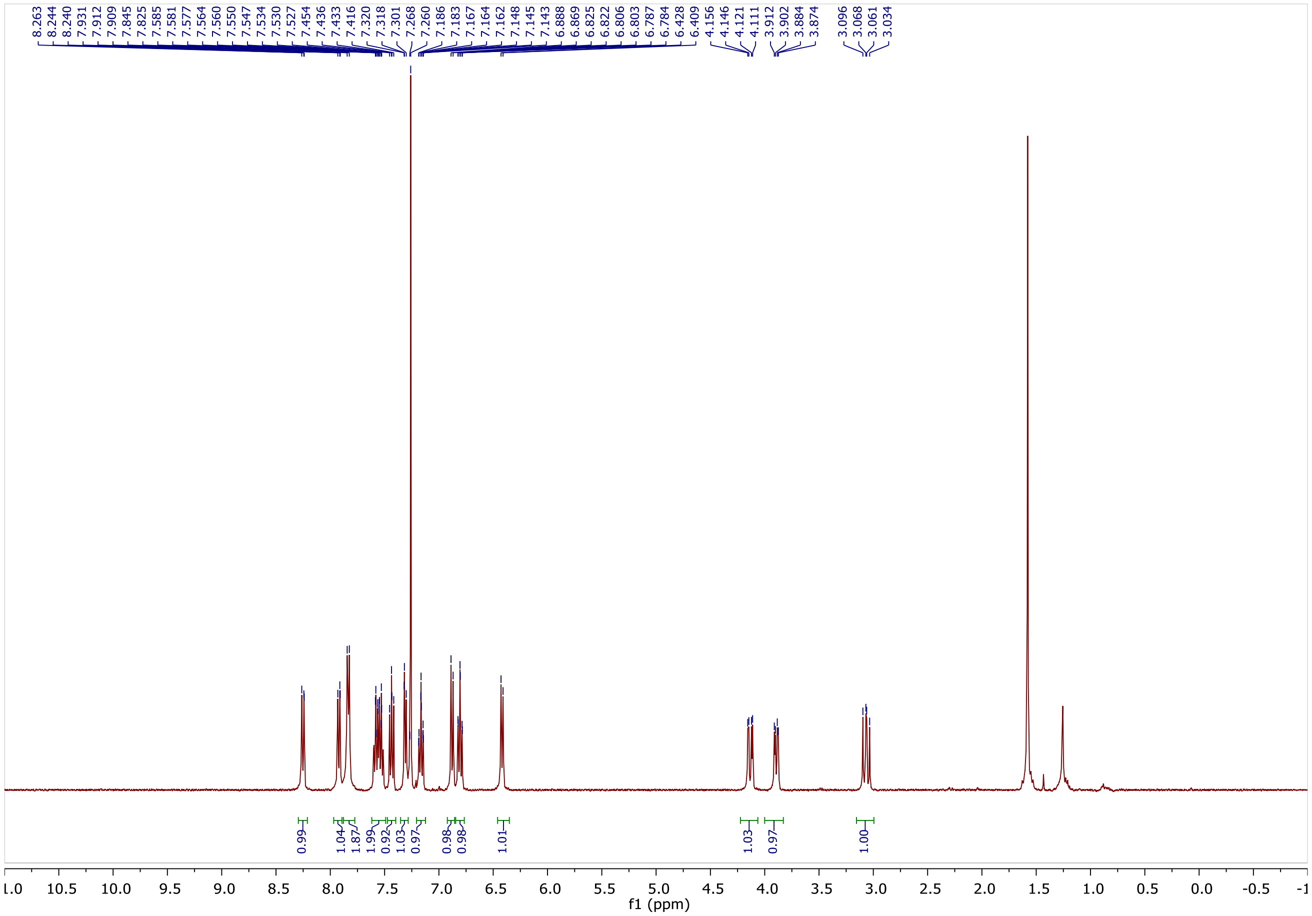

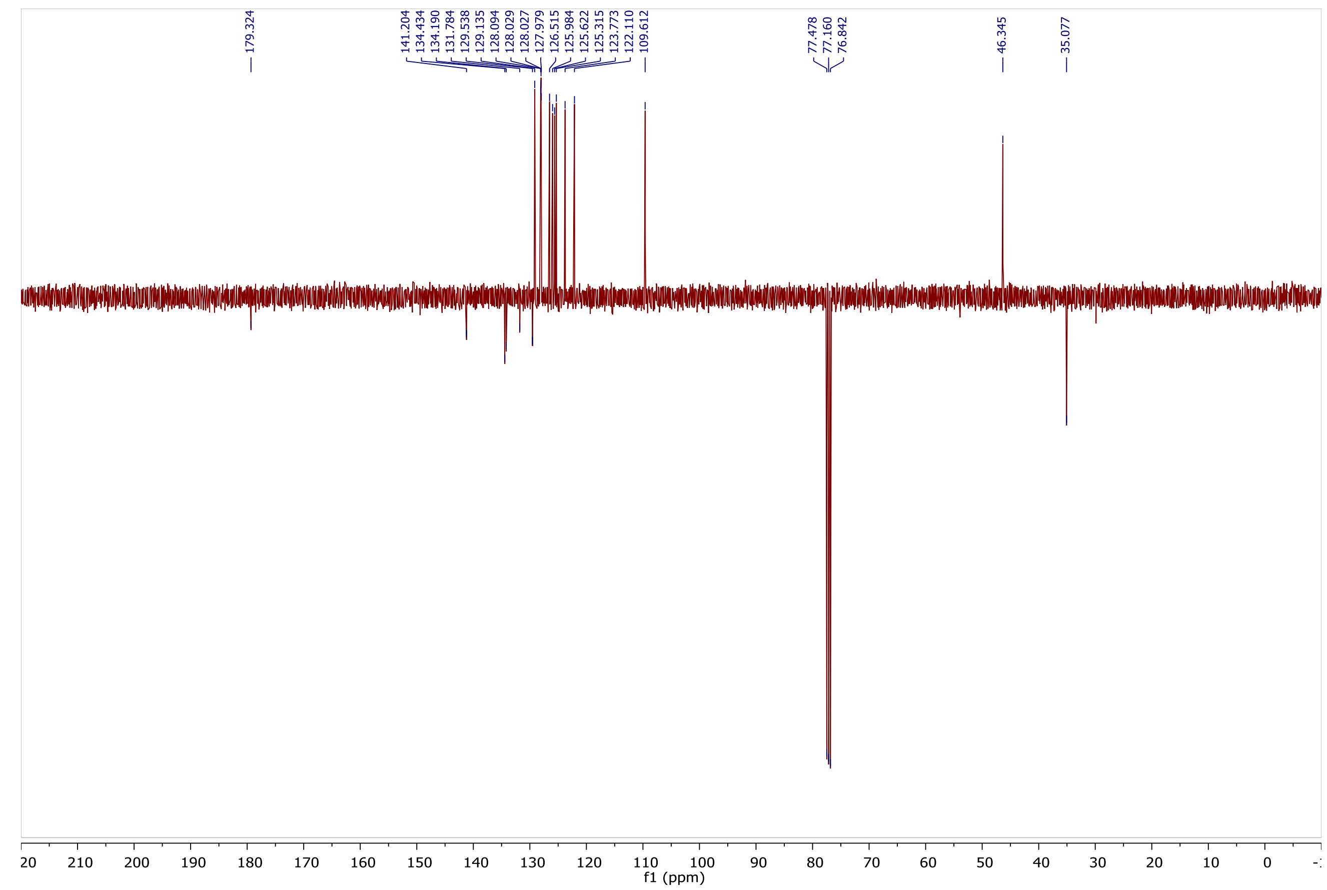

3-(naphthalen-2-ylmethyl)indolin-2-one

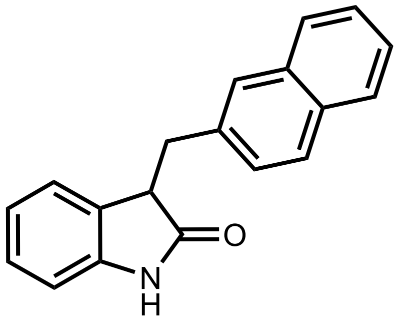

15

The title compound was prepared according to general procedure 1 using 2-naphthalenemethanol (190 mg, 1.2 mmol) and oxindole (133 mg, 1.0 mmol). Purification by flash silica chromatography (eluent = 15% EtOAc in hexanes, 30 x 150 mm silica) gave the title compound as a cream solid (249 mg, 91%). mp 151-153 °C; R_f = 0.38 (eluent = 30 % EtOAc in hexanes); ν_{max}/cm^{-1} (film): 3175, 3136, 3073, 3024, 2905, 1694, 1614, 1472, 1337, 1310, 1236, 1148, 968, 820, 754, 735, 664, 583; **^{1}H NMR (400 MHz, $CDCl_3$)** δ_H: 3.12 (1H, dd, *J* 13.6, 10.0), 3.65 (1H, dd, *J* 14.0, 4.8), 3.88 (1H, dd, *J* 10.0, 4.4), 6.78 (2H, t, *J* 8.0), 6.87 (1H, t, *J* 5.6), 7.14 (1H, t, *J* 7.6), 7.33 (1H, dd, *J* 8.4, 2.0), 7.39-7.51 (2H, m), 7.62 (1H, m), 7.69-7.82 (3H, m), 7.84 (1H, br s); **^{13}C NMR (101 MHz, $CDCl_3$)** δ_C: 36.9, 47.4, 109.7, 122.2, 125.1, 125.7, 126.1, 127.7, 127.7, 127.8, 128.1, 128.2, 128.3, 129.1, 132.5, 133.5, 135.5, 141.4, 179.2; HRMS (**NSI$^+$**) calculated for $[C_{19}H_{16}NO]^+$ $(M + H)^+$ m/z : 274.1226, found 274.1230 (+1.3 ppm).

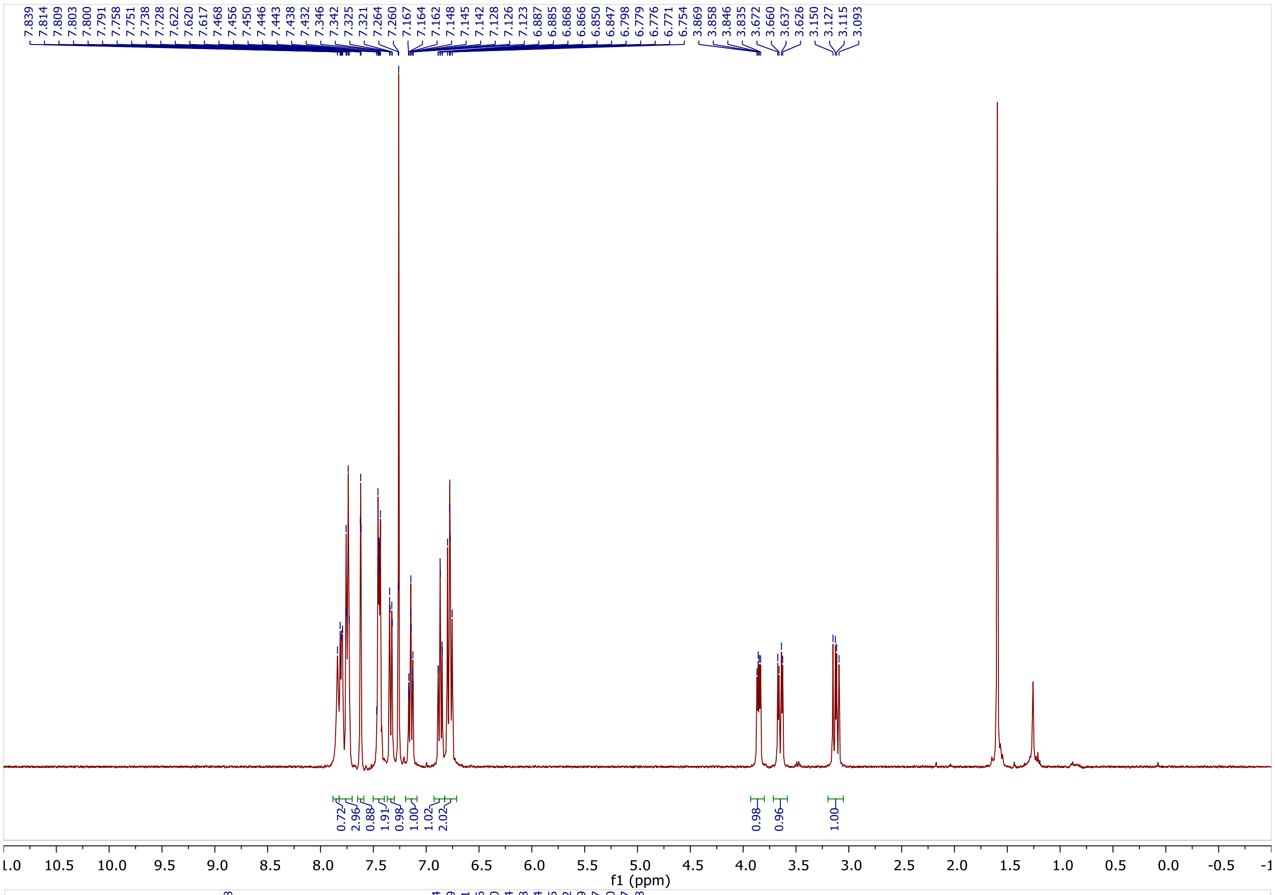
7.839
7.814
7.809
7.803
7.800
7.791
7.758
7.751
7.738
7.728
7.622
7.620
7.617
7.468
7.456
7.450
7.446
7.443
7.438
7.432
7.346
7.342
7.325
7.321
7.264
7.260
7.167
7.164
7.162
7.148
7.145
7.142
7.128
7.126
7.123
6.887
6.885
6.868
6.866
6.850
6.847
6.798
6.779
6.776
6.771
6.754
3.869
3.858
3.846
3.835
3.672
3.660
3.637
3.626
3.150
3.127
3.115
3.093
0.72
2.96
0.88
1.91
0.98
1.00
1.02
2.02
0.98
0.96
1.00
f1 (ppm)
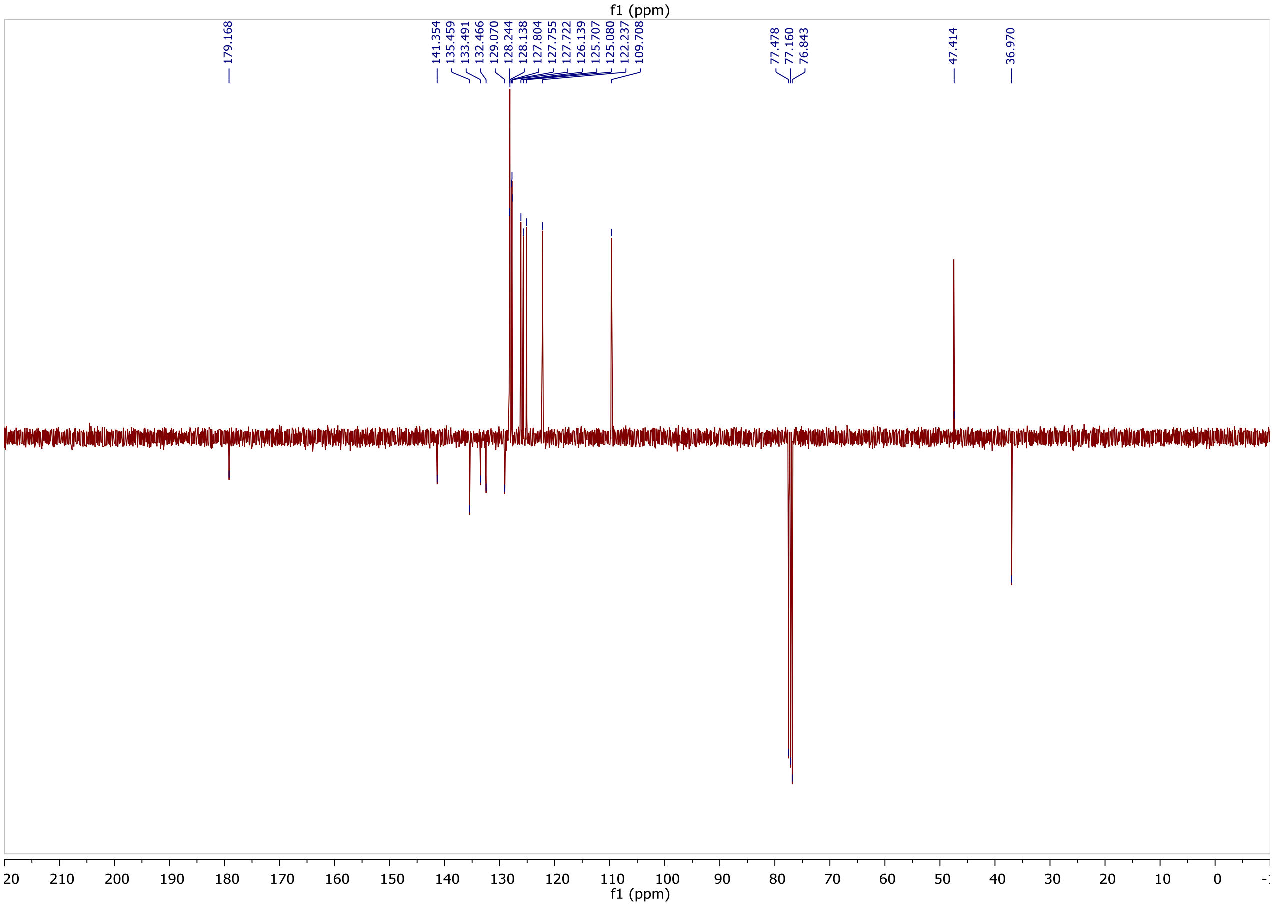
179.168
141.354
135.459
133.491
132.466
129.070
128.244
128.138
127.804
127.755
127.722
126.139
125.707
125.080
122.237
109.708
77.478
77.160
76.843
47.414
36.970
f1 (ppm)

3-(4-methoxybenzyl)indolin-2-one

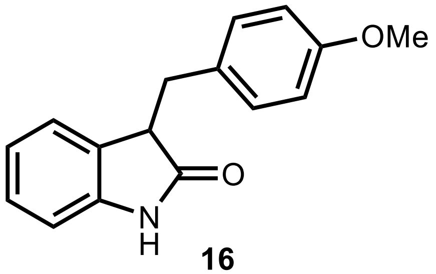

16

The title compound was prepared according to general procedure 1 using 4-methoxybenzyl alcohol (166 mg, 1.2 mmol) and oxindole (133 mg, 1.0 mmol). Purification by flash silica chromatography (eluent = 15% EtOAc in petroleum ether, 30 x 150 mm silica) gave the title compound as a pink solid (218 mg, 86%). mp 111-114 °C (Lit. 110-111 °C);[12] R_f = 0.33 (eluent = 15% EtOAc in petroleum ether); **^{1}H NMR (400 MHz, $CDCl_3$)** δ_H: 2.92 (1H, dd, *J* 13.6, 7.6), 3.41 (1H, dd, *J* 13.6, 4.4), 3.70 (1H, dd, *J* 8.8, 4.4), 3.76 (3H, s), 6.75-6.83 (4H, m), 6.91 (1H, t, *J* 6.4), 7.06 (2H, d, *J* 8.8), 7.16 (1H, t, *J* 6.8), 8.85 (1H, br s); **^{13}C NMR (101 MHz, $CDCl_3$)** δ_C: 35.9, 47.7, 55.3, 109.8, 113.9, 122.1, 150.0, 128.0, 129.2, 129.8, 130.5, 141.6, 158.4, 179.8. Spectroscopic data in accordance with the literature.[12]

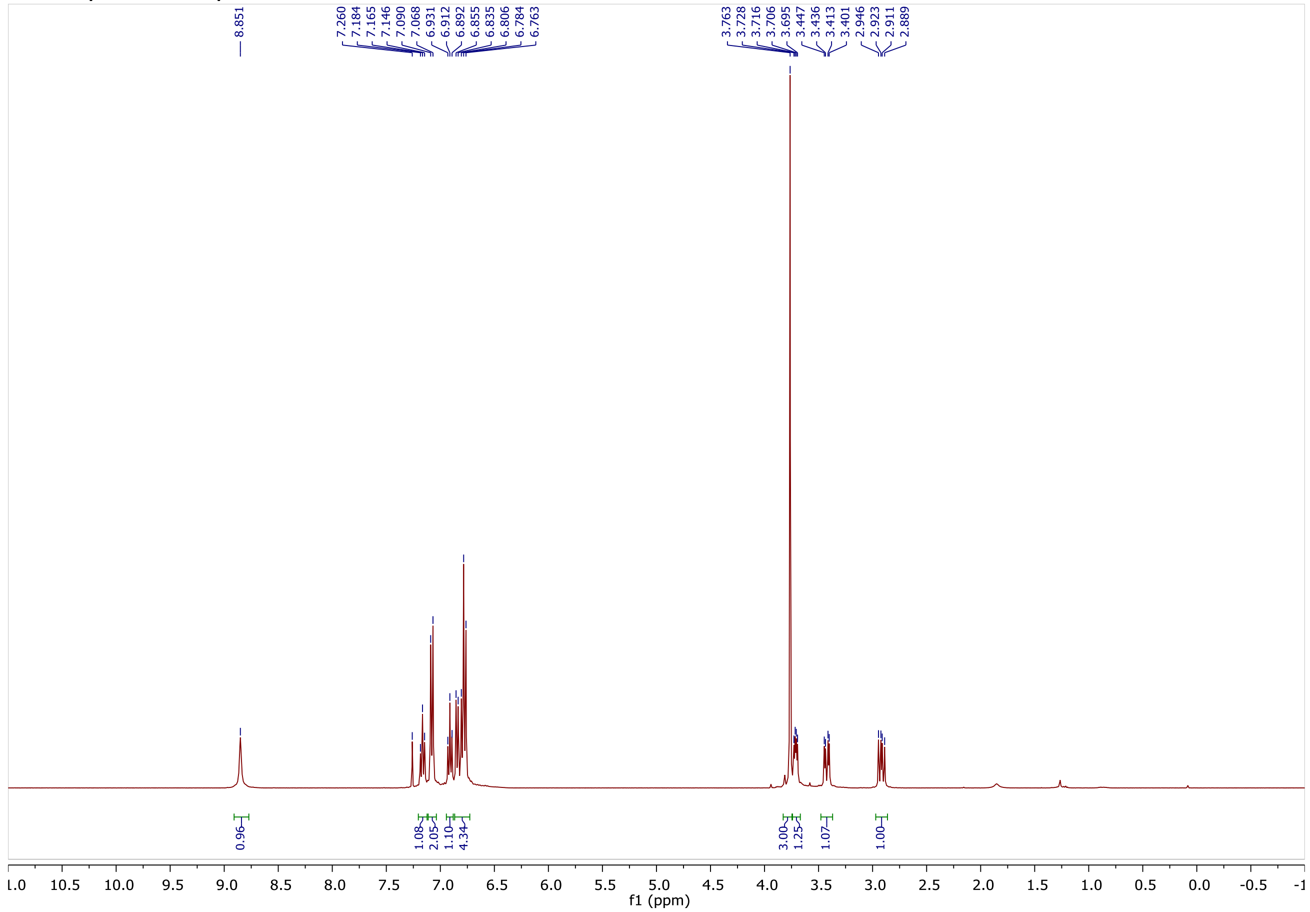

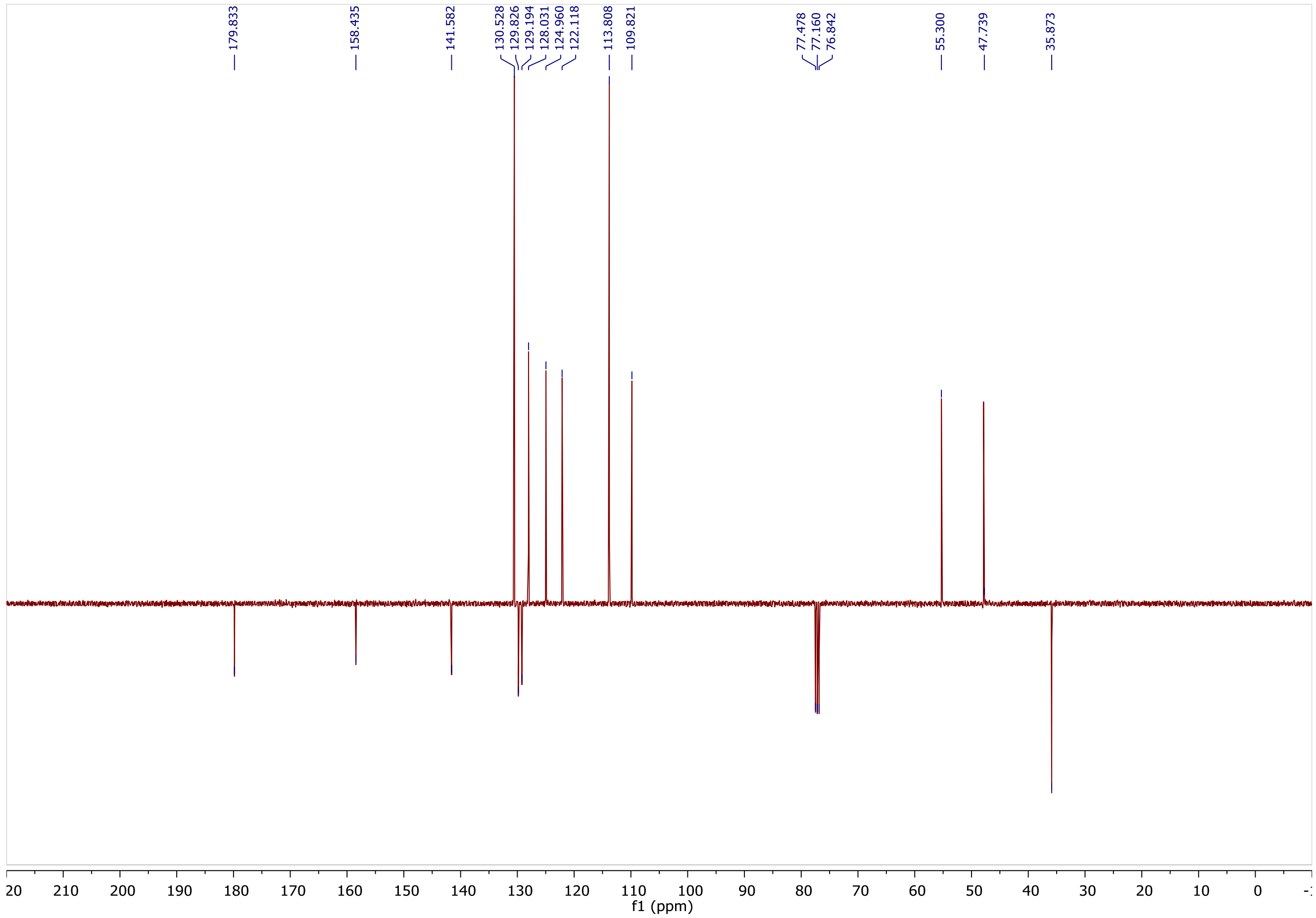

3-(4-(benzyloxy)benzyl)indolin-2-one

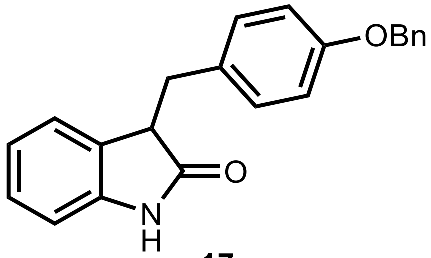

17

The title compound was prepared according to general procedure 1 using 4-benzyloxybenzyl alcohol (257 mg, 1.2 mmol) and oxindole (133 mg, 1.0 mmol). Purification by flash silica chromatography (eluent = 20-35% EtOAc in hexanes, 30 x 170 mm silica) gave the title compound as a pink solid (294 mg, 90%). mp 139-141 °C; R_f = 0.20 (eluent = 30% EtOAc in hexanes); ν_{max} / cm $^{-1}$ (film): 3184, 3130, 3084, 3034, 2895, 2847, 1697, 1618, 1512, 1468, 1234, 1175, 1013, 810, 735, 584; **^{1}H NMR (500 MHz, $CDCl_3$)** δ_H: 2.93 (1H, dd, *J* 14.0, 9.0), 3.42 (1H, dd, *J* 13.5, 4.5), 3.71 (1H, dd, *J* 8.5, 4.5), 5.02 (2H, s), 6.80 (2H, d, *J* 7.5), 6.86 (2H, d, *J* 8.5), 6.91 (1H, t, *J* 7.5), 7.07 (2H, d, *J* 8.0), 7.16 (1H, t, *J* 7.5), 7.32 (1H, t, *J* 7.0), 7.35-7.45 (4H, m), 7.88 (1H, br s); **^{13}C NMR (500 MHz, $CDCl_3$)** δ_C: 35.9, 47.8, 70.1, 109.8, 114.8, 122.1, 125.0, 127.6, 128.0, 128.1, 128.7, 129.2, 130.1, 130.6, 137.1, 141.5, 157.7, 179.6; HRMS (**ES$^+$**) calculated for $[C_{22}H_{20}NO_2]^+$ $(M+H)^+$ m/z : 330.1494, found 330.1493 (-0.3 ppm).

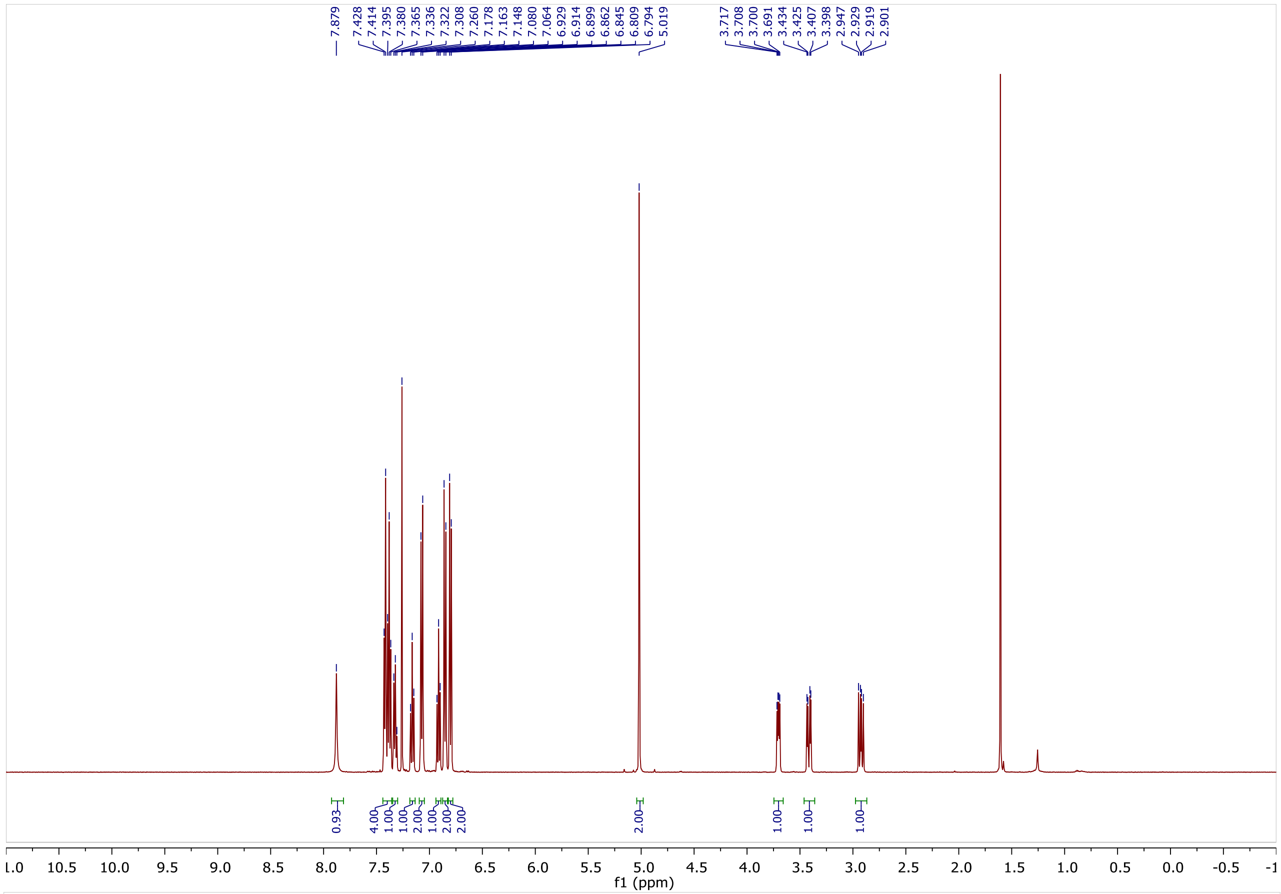
7.879
7.428
7.414
7.395
7.380
7.365
7.336
7.322
7.308
7.260
7.178
7.163
7.148
7.080
7.064
6.929
6.914
6.899
6.862
6.845
6.809
6.794
5.019
3.717
3.708
3.700
3.691
3.434
3.425
3.407
3.398
2.947
2.929
2.919
2.901
0.93
4.00
1.00
1.00
2.00
1.00
2.00
2.00
2.00
1.00
1.00
1.00
f1 (ppm)

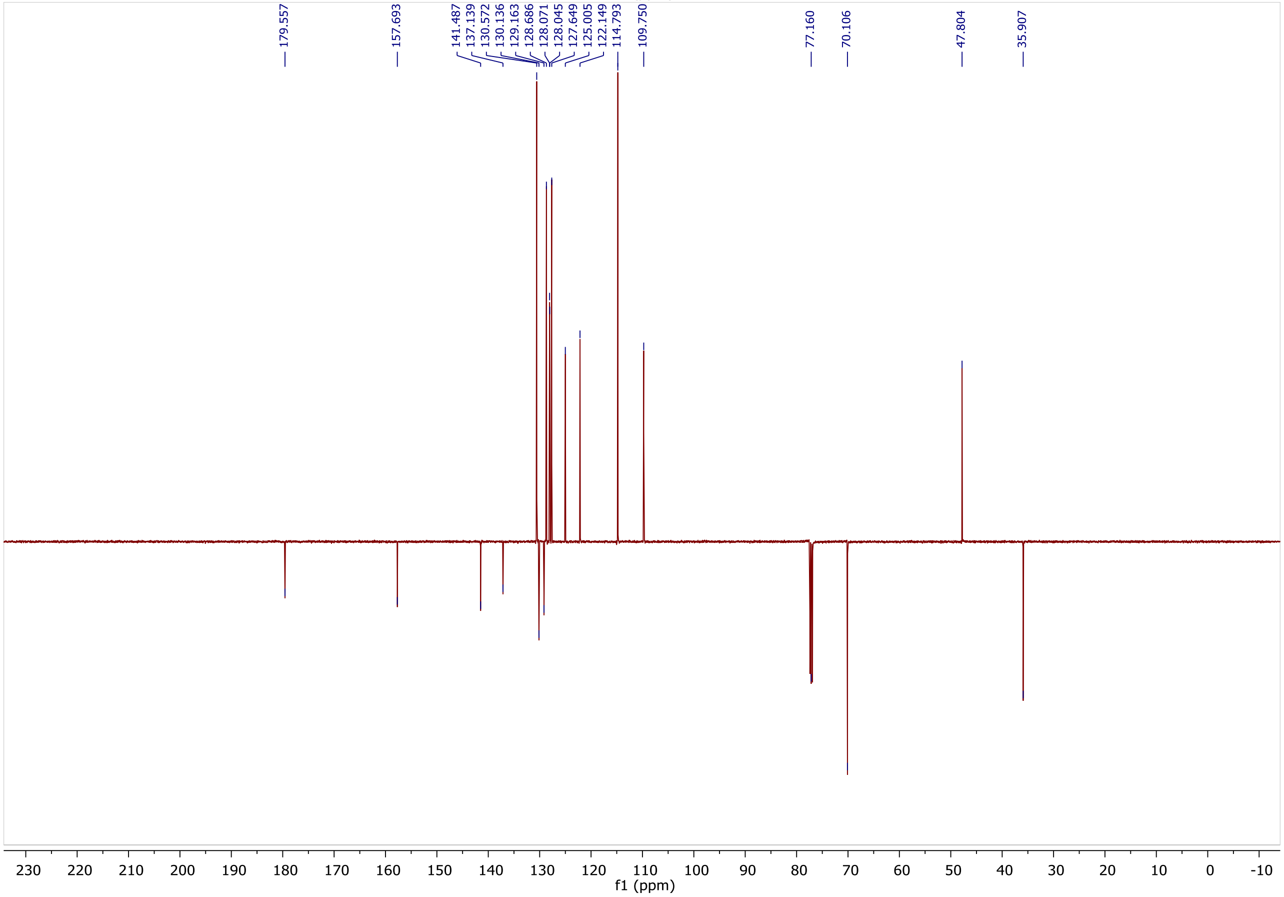
179.557
157.693
141.487
137.139
130.572
130.136
129.163
128.686
128.071
128.045
127.649
125.005
122.149
114.793
109.750
77.160
70.106
47.804
35.907
f1 (ppm)

3-(4-(trifluoromethyl)benzyl)indolin-2-one

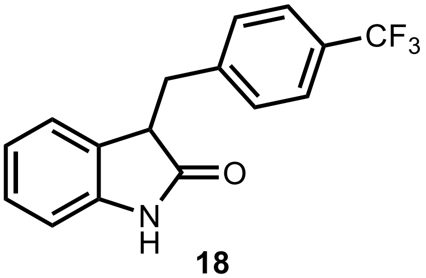

18

The title compound was prepared according to general procedure 1 using 3-(4-trifluoromethyl) benzyl alcohol (164 µL, 211 mg, 1.2 mmol) and oxindole (133 mg, 1.0 mmol). Purification by flash silica chromatography (eluent = 20% EtOAc in hexanes, 30 x 150 mm silica) gave the title compound as a cream solid (259 mg, 89%). mp 128-130 °C; (Lit.112-113 °C);[12] R_f = 0.30 (eluent = 30% EtOAc in hexanes); **^{1}H NMR (400 MHz, $CDCl_3$)** δ_H: 3.09 (1H, dd, *J* 14.0, 8.4), 3.46 (1H, dd, *J* 14.0, 4.8), 3.67 (1H, dd, *J* 8.0, 4.4), 6.82 (2H, dd, *J* 16.0, 7.6), 6.93 (1H, t, *J* 7.6), 7.17 (1H, t, *J* 8.0), 7.21-7.32 (2H, m), 7.41-7.53 (2H, m), 8.06 (1H, br, s); **^{19}F NMR (376 MHz, $CDCl_3$)** δ_F: -62.4; **^{13}C NMR (101 MHz, $CDCl_3$)** δ_C: 36.3, 47.3, 110.1, 122.4, 124.3 (q, *J* 272.2), 124.7, 125.4 (q, *J* 3.5), 128.4, 128.5, 129.2 (q, *J* 32.1), 129.9, 141.5, 141.9, 179.3. Spectroscopic data in accordance with the literature.[12]

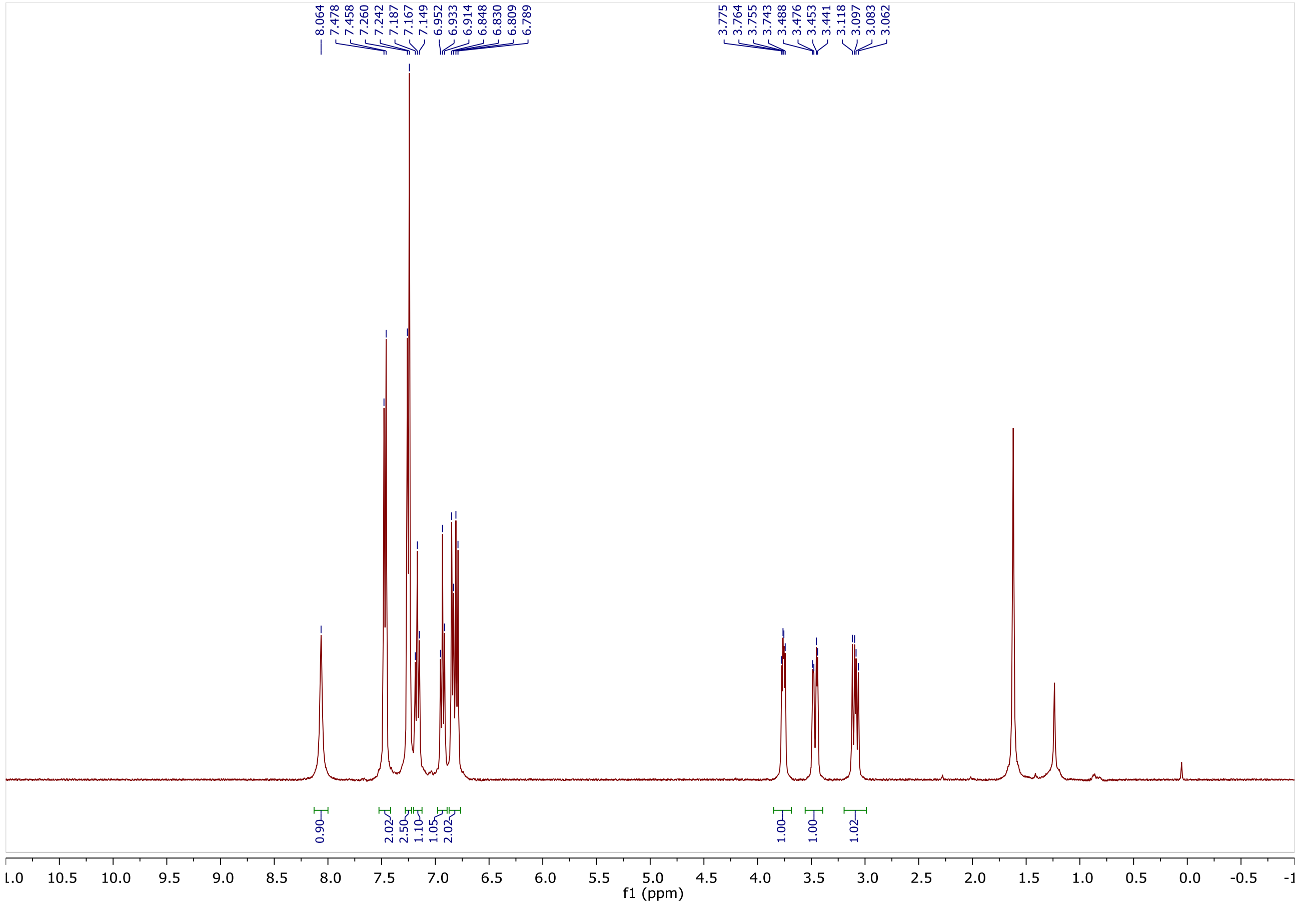

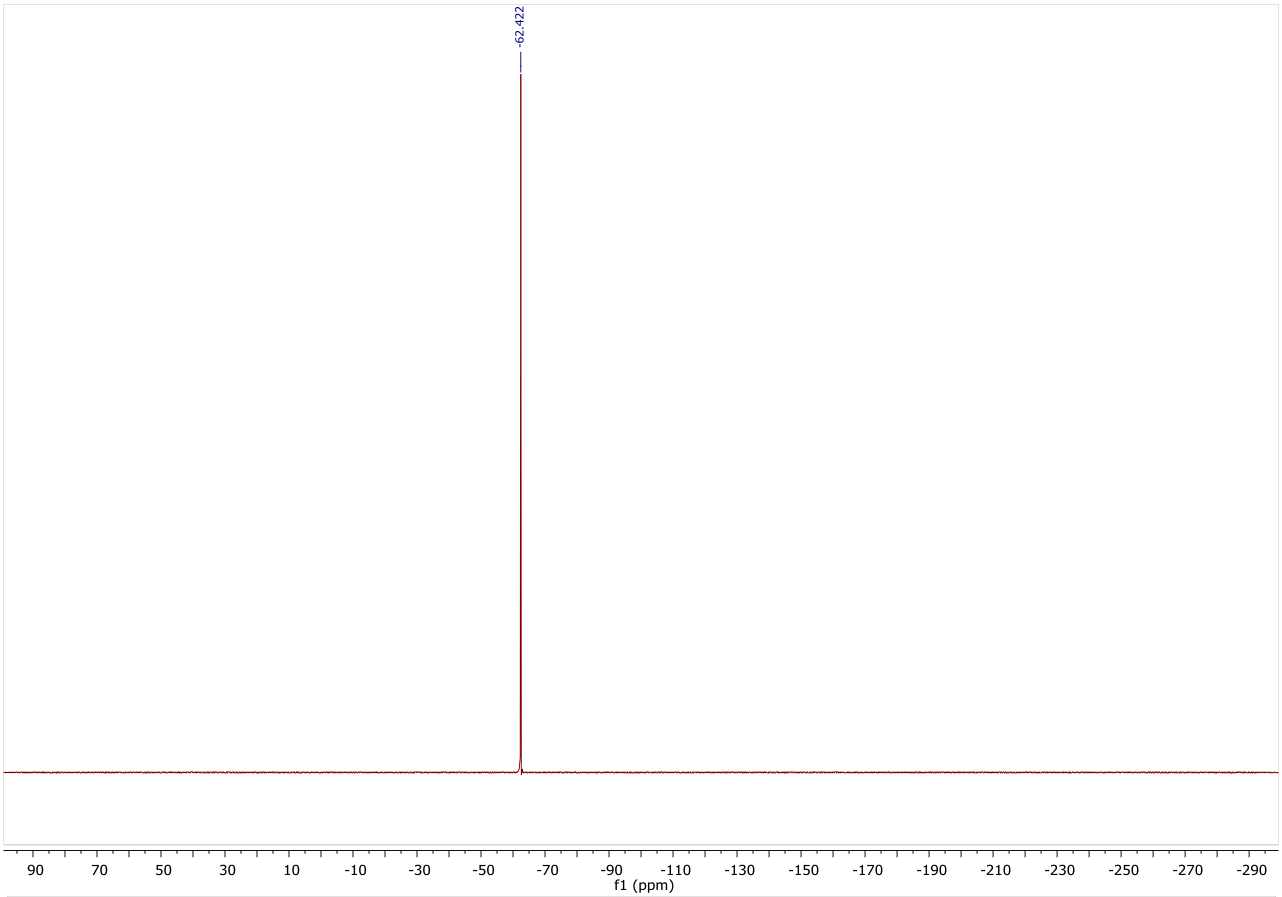

-62.422
90
70
50
30
10
-10
-30
-50
-70
-90
-110
-130
-150
-170
-190
-210
-230
-250
-270
-290
f1 (ppm)

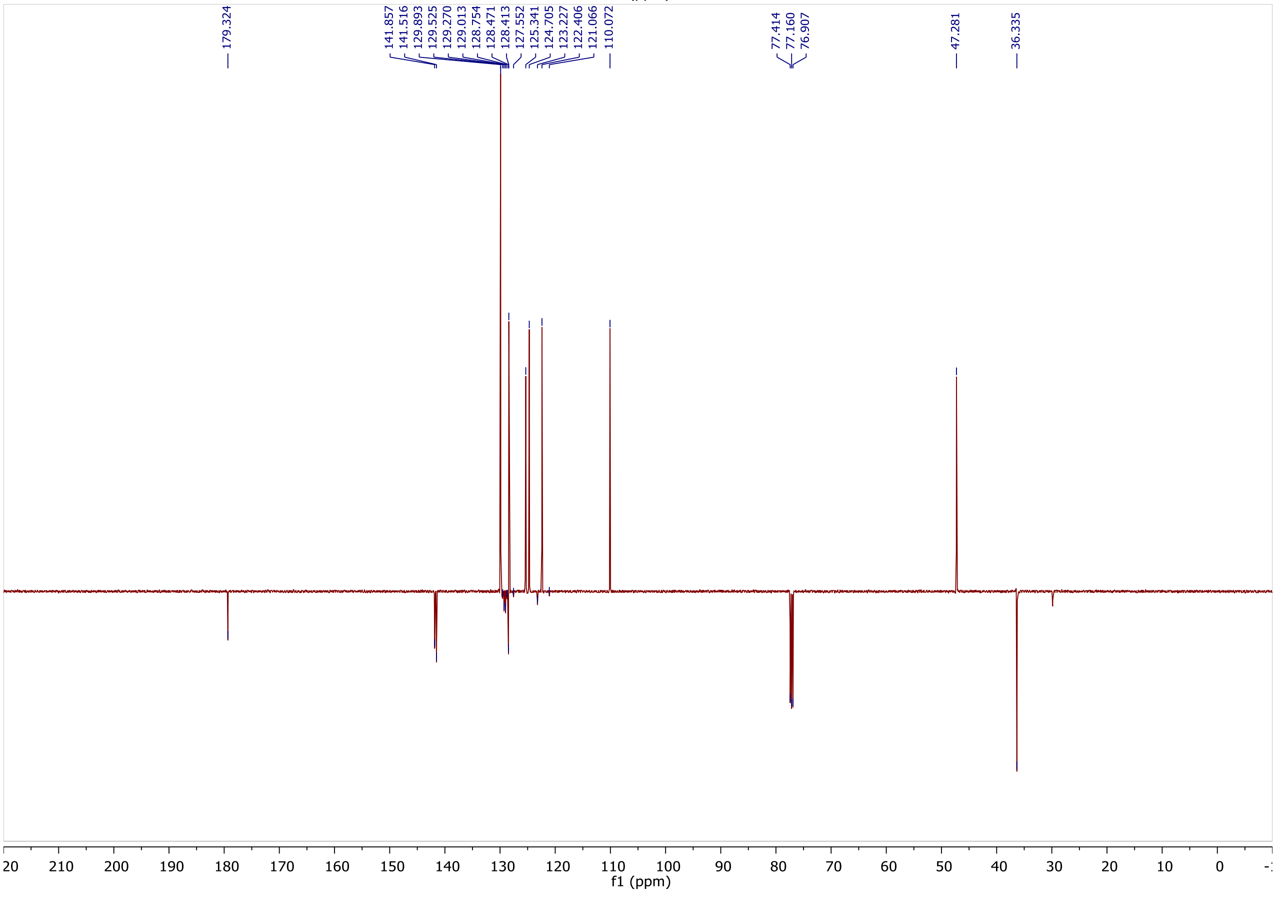

179.324
141.857
141.516
129.893
129.525
129.270
129.013
128.754
128.471
128.413
127.552
125.341
124.705
123.227
122.406
121.066
110.072
77.414
77.160
76.907
47.281
36.335
20
210
200
190
180
170
160
150
140
130
120
110
100
90
80
70
60
50
40
30
20
10
0
f1 (ppm)

4-((2-oxoindolin-3-yl)methyl)benzonitrile

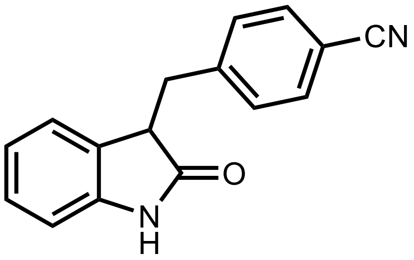

19

The title compound was prepared according to general procedure 1 using 4-(hydroxymethyl)benzonitrile (160 mg, 1.2 mmol) and oxindole (133 mg, 1.0 mmol). Purification by flash silica chromatography (eluent = 20-35% EtOAc in hexanes, 30 x 180 mm silica) gave the title compound as an off-white solid (208 mg, 83%). mp 132-134 °C; R_f = 0.10 (eluent = 30% EtOAc in hexanes); ν_{max}/cm^{-1} (film): 3184, 3132, 3086, 3034, 2895, 2843, 2226, 1703, 1616, 1472, 1412, 1337, 1234, 1175, 1103, 835, 756, 652, 583; **^{1}H NMR (500 MHz, $CDCl_3$)** δ_H: 3.17 (1H, dd, *J* 13.5, 7.5), 3.45 (1H, dd, *J* 13.5, 5.0), 3.78 (1H, dd, *J* 8.0, 5.0), 6.80 (1H, d, *J* 7.5), 6.89 (1H, d, *J* 7.5), 6.97 (1H, t, *J* 7.5), 7.19 (1H, t, *J* 7.5), 7.25 (2H, d, *J* 8.0), 7.50 (2H, d, *J* 8.5), 7.98 (1H, br s); **^{13}C NMR (126 MHz, $CDCl_3$)** δ_C: 36.5, 47.0, 110.0, 110.8, 118.9, 122.5, 124.7, 128.1, 128.6, 130.4, 132.2, 141.4, 143.2, 178.6; HRMS (**ES$^+$**) calculated for $[C_{16}H_{13}N_2O]^+$ $(M+H)^+$ m/z : 249.1028, found 249.1030 (0.8 ppm).

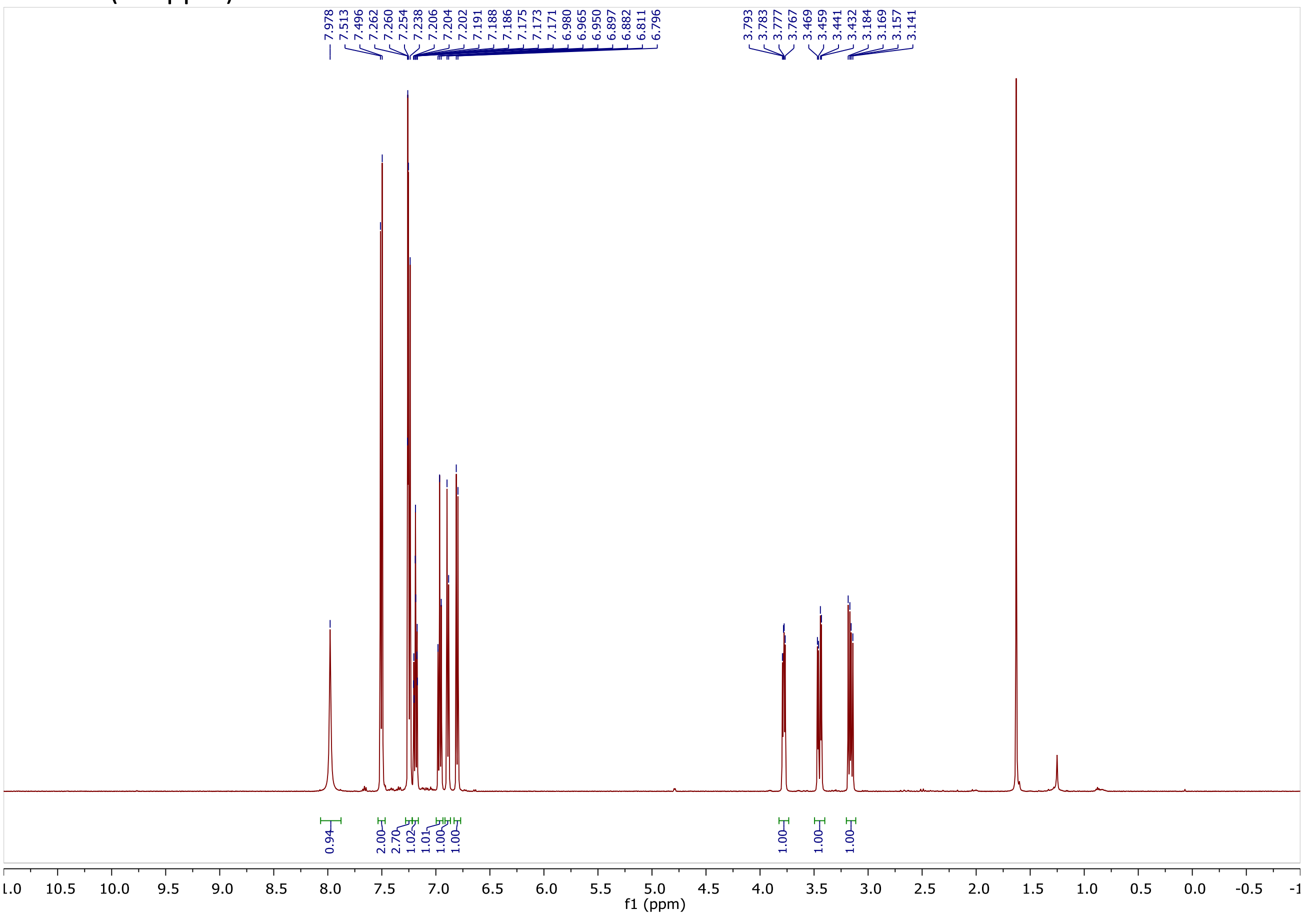

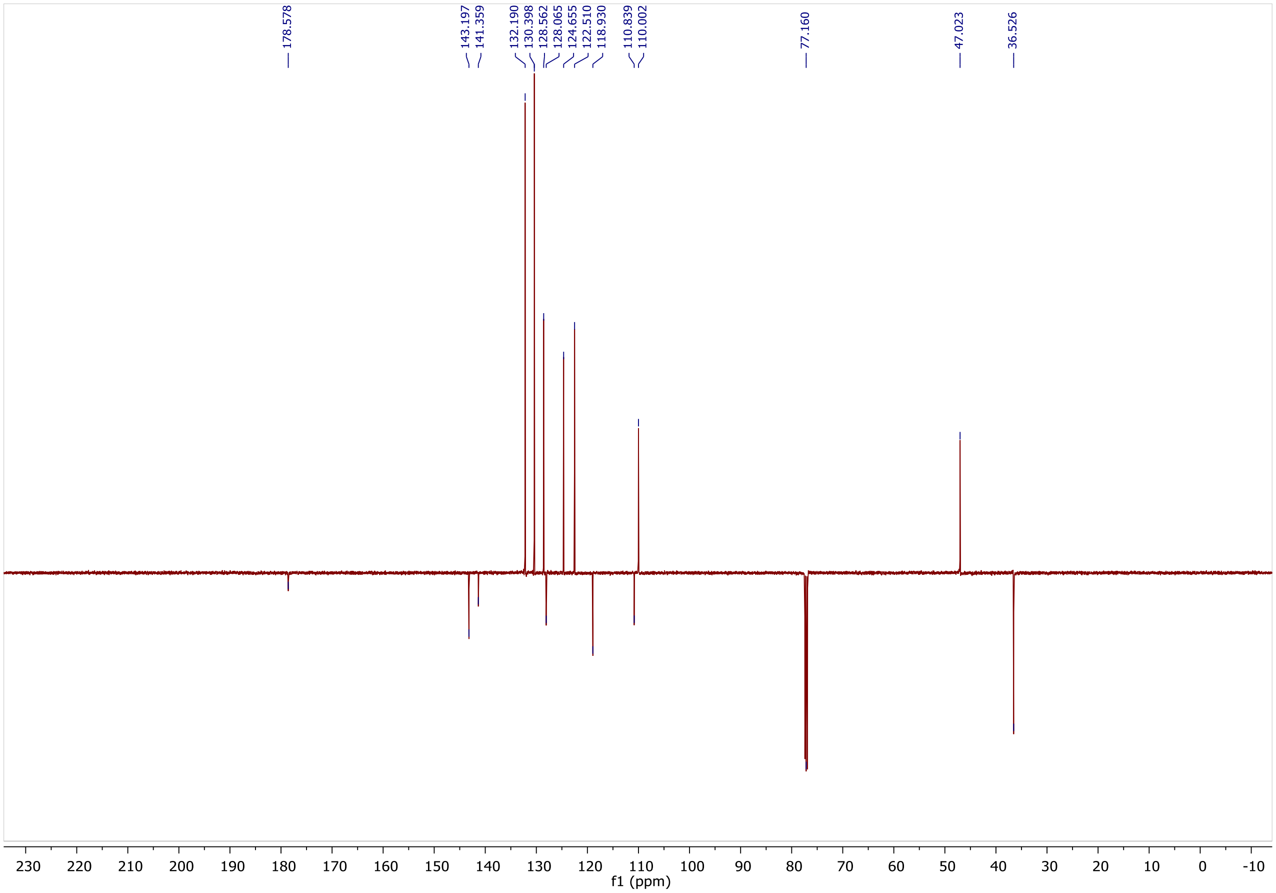

3-(4-vinylbenzyl)indolin-2-one

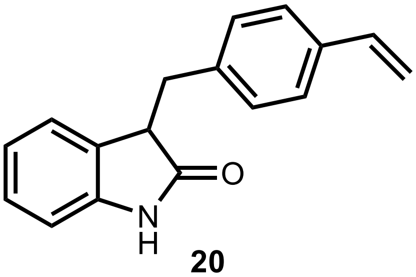

20

The title compound was prepared according to general procedure 1 using (4-vinylphenyl)methanol (161 mg, 1.2 mmol) and oxindole (133 mg, 1.0 mmol). Purification by flash silica chromatography (eluent = 20% EtOAc in hexanes, 30 x 160 mm silica) gave the title compound as an off-white solid (130 mg, 52%). mp 104-106 °C; R_f = 0.33 (eluent = 40% EtOAc in hexanes); ν_{max}/cm $^{-1}$ (film): 3169, 3132, 3073, 3019, 2889, 2832, 1701, 1616, 1510, 1466, 1339, 1236, 988, 907, 839, 764, 748, 662, 584, 490; **^{1}H NMR (500 MHz, $CDCl_3$)** δ_H: 2.96 (1H, dd, *J* 13.5, 9.0), 3.47 (1H, dd, *J* 13.5, 4.5), 3.75 (1H, dd, *J* 9.5, 5.0), 5.21 (1H, d, *J* 11.0), 5.71 (1H, d, *J* 17.5), 6.68 (1H, dd, *J* 17.5, 11.0), 6.81 (2H, t, *J* 8.0), 6.91 (1H, t, *J* 7.5), 7.07-7.21 (3H, m), 7.29 (2H, d, *J* 8.0), 8.25 (1H, br s); **^{13}C NMR (126 MHz, $CDCl_3$)** δ_C: 36.5, 47.5, 109.7, 113.6, 122.2, 125.0, 126.3, 128.1, 129.0, 129.8, 136.1, 136.7, 137.5, 141.3, 179.0; HRMS (**ES$^+$**) calculated for $[C_{17}H_{16}NO]^+$ $(M+H)^+$ m/z : 250.1232, found 250.1227 (-2.0 ppm).

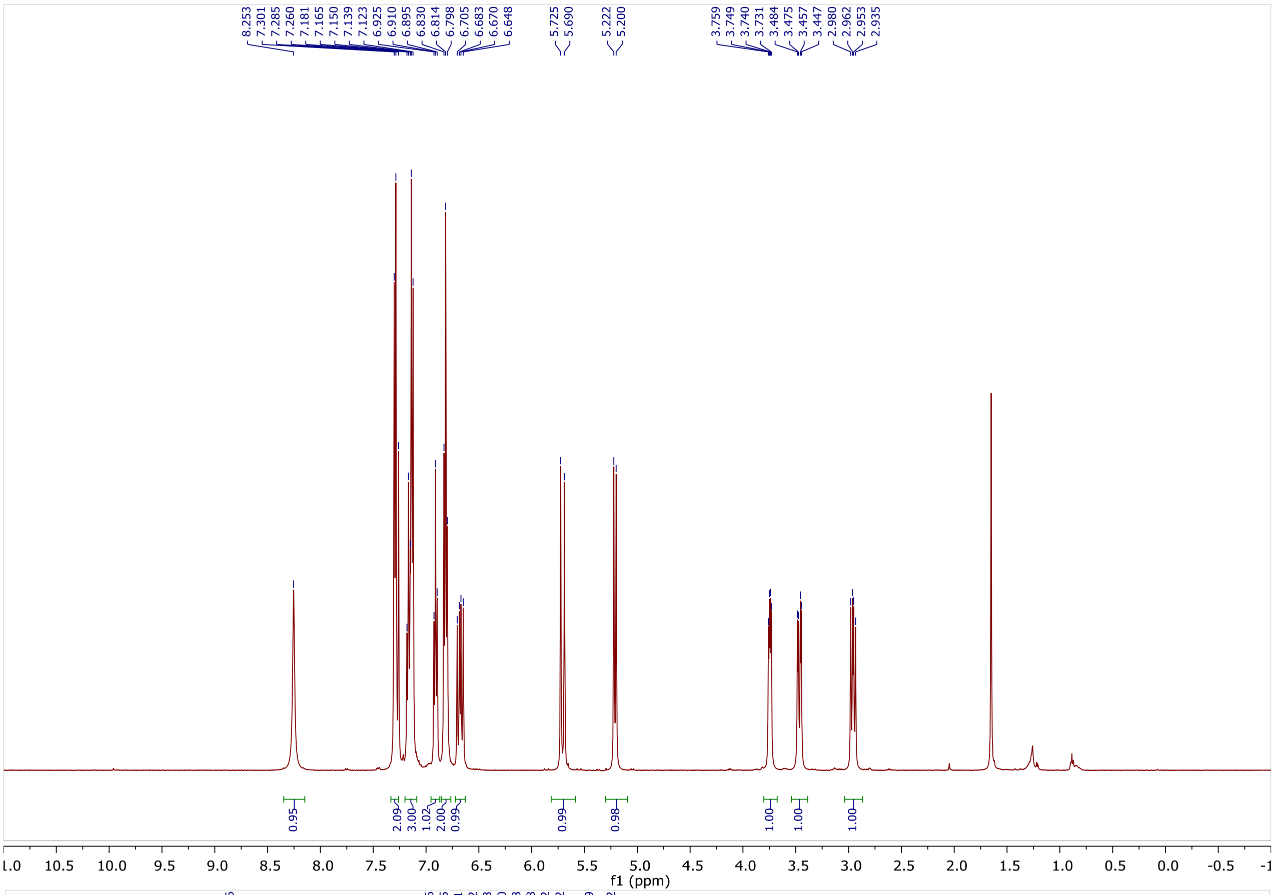
8.253
7.301
7.285
7.260
7.181
7.165
7.150
7.139
7.123
6.925
6.910
6.895
6.830
6.814
6.798
6.705
6.683
6.670
6.648
5.725
5.690
5.222
5.200
3.759
3.749
3.740
3.731
3.484
3.475
3.457
3.447
2.980
2.962
2.953
2.935
0.95
2.09
3.00
1.02
2.00
0.99
0.99
0.98
1.00
1.00
1.00
f1 (ppm)

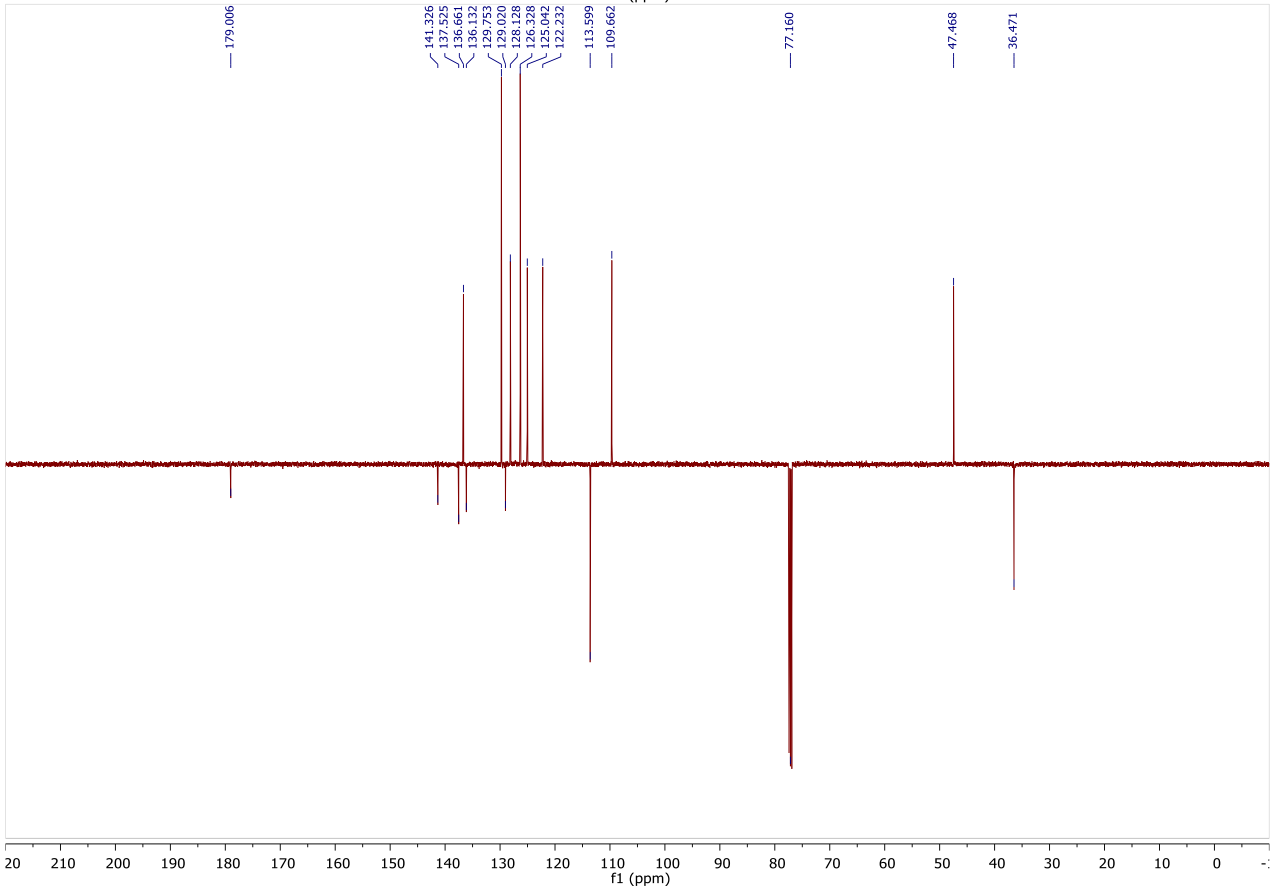
179.006
141.326
137.525
136.661
136.132
129.753
129.020
128.128
126.328
125.042
122.232
113.599
109.662
77.160
47.468
36.471
f1 (ppm)

3-(4-Iodobenzyl)indolin-2-one

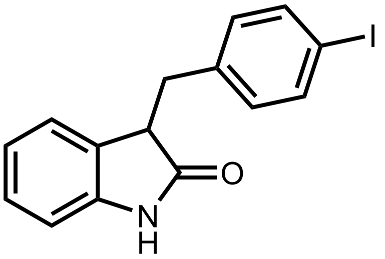

21

The title compound was prepared according to general procedure 1 using 4-iodobenzyl alcohol (281 mg, 1.2 mmol) and oxindole (133 mg, 1.0 mmol). Purification by flash silica chromatography (eluent = 15% EtOAc in petroleum ether, 30 x 150 mm silica) gave the title compound as a cream solid (213 mg, 91%). mp 153-155 °C; R_f = 0.35 (eluent = 20% EtOAc in petroleum ether); ν_{max} /cm $^{-1}$ (film): 3129, 3061, 3024, 2918, 2832, 1703, 1609, 1483, 1466, 1437, 1398, 1337, 1310, 1234, 1177, 1098, 1059, 1003, 775, 752, 658, 619; **^{1}H NMR (400 MHz, CDCl$_3$)** δ_H: 2.98 (1H, dd, *J* 14.0, 8.5), 3.37 (1H, dd, *J* 13.5, 4.5), 3.72 (1H, dd, *J* 8.0, 4.5), 6.79 (1H, dd, *J* 8.0), 6.84-6.92 (3H, m), 6.94 (1H, t, *J* 7.5), 7.18 (1H, t, *J* 8.0), 7.55 (2H, d, *J* 8.5), 7.72 (1H, br s); **^{13}C NMR (126 MHz, CDCl$_3$)** δ_C: 36.1, 47.3, 92.3, 109.9, 122.3, 124.9, 128.3, 128.6, 131.6, 137.4, 137.5, 141.4, 178.9; HRMS (**NSI$^+$**) calculated for $[C_{15}H_{13}NOI]^+$ $(M+H)^+$ m/z: 350.0036, found 350.0040 (+1.0 ppm).

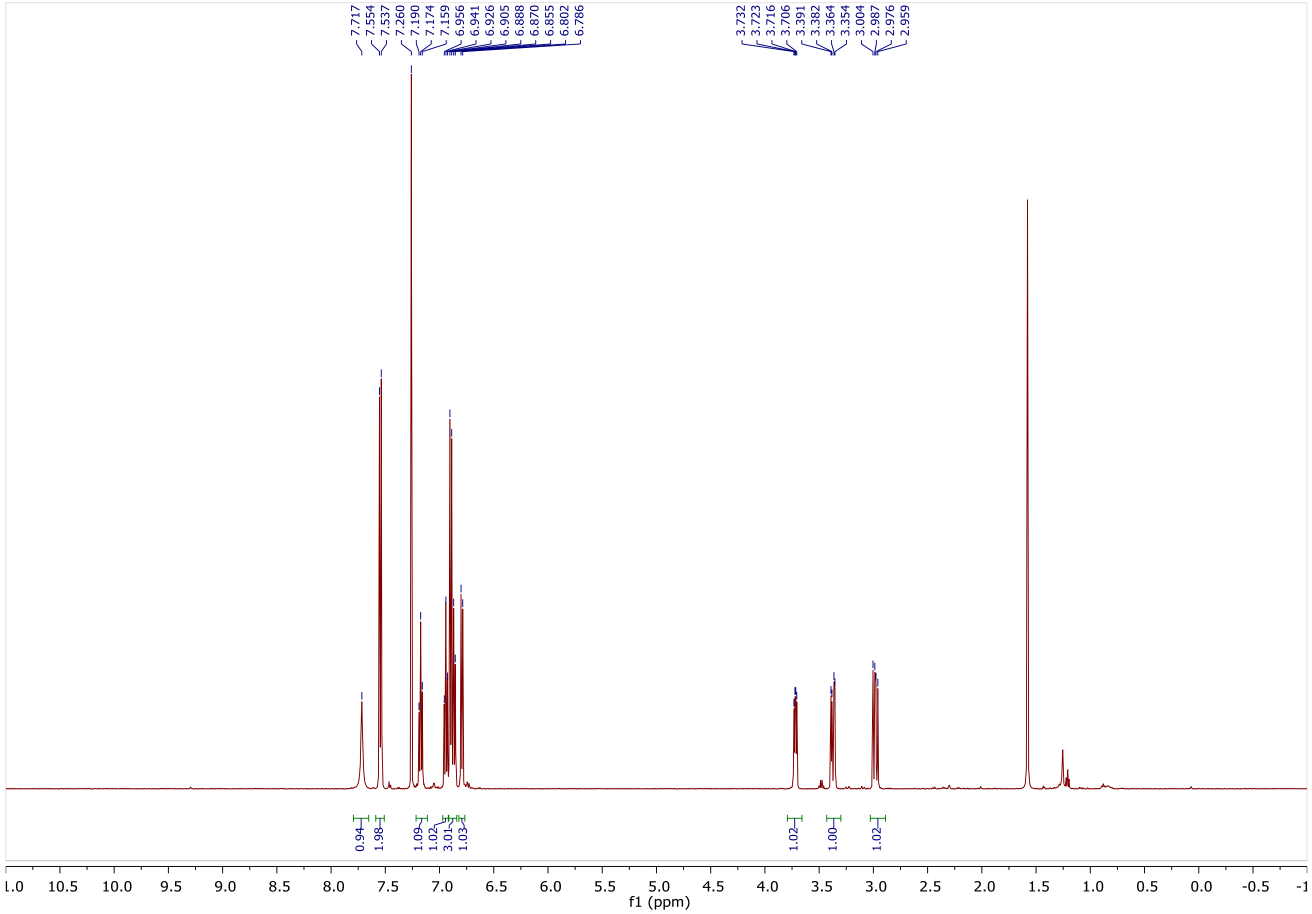

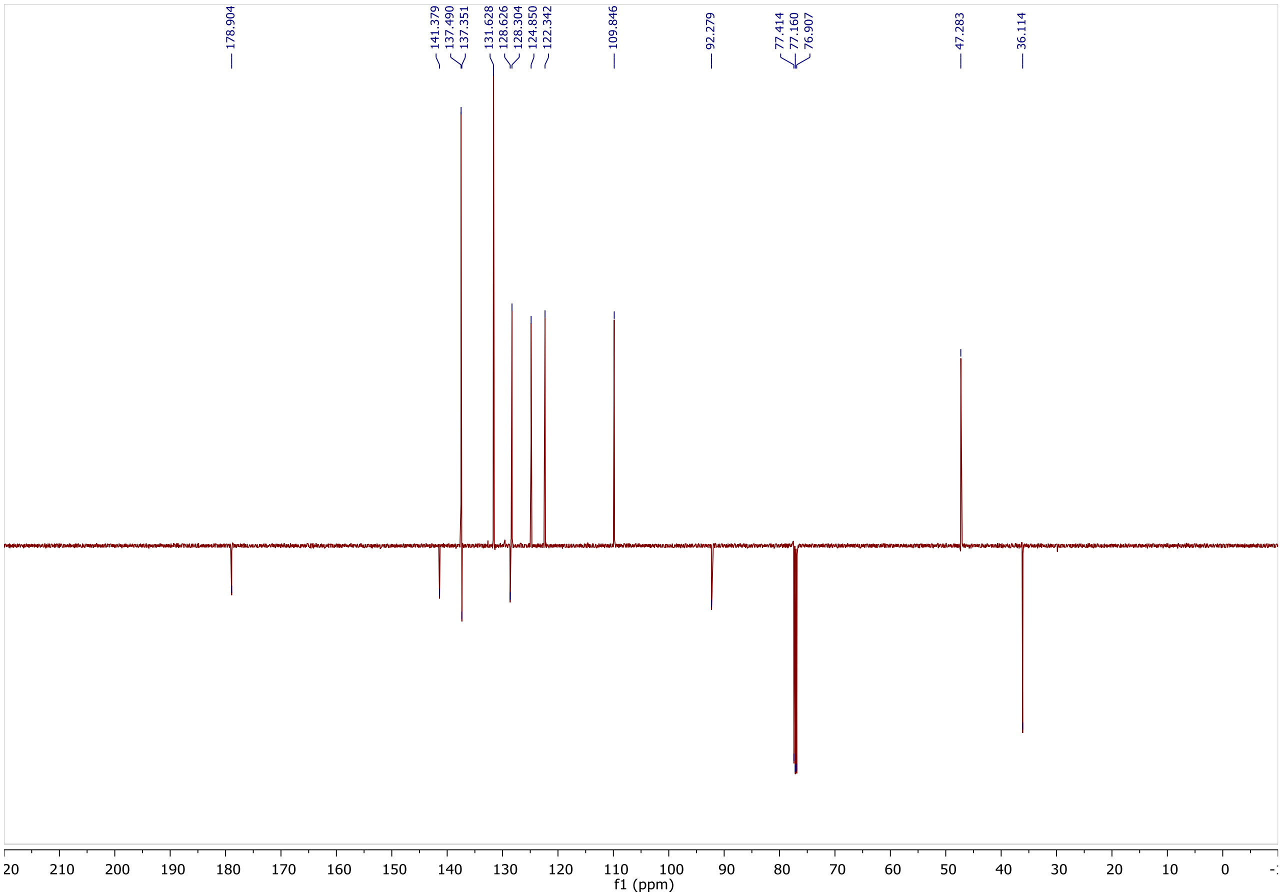

3-(4-fluorobenzyl)indolin-2-one

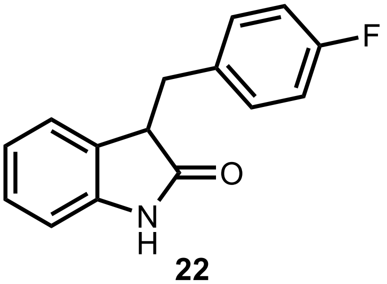

22

The title compound was prepared according to general procedure 1 using 4-fluorobenzyl alcohol (131 µL, 151 mg 1.2 mmol) and oxindole (133 mg, 1.0 mmol). Purification by flash silica chromatography (eluent = 15% EtOAc in petroleum ether, 30 x 150 mm silica) gave the title compound as pale a yellow solid (213 mg, 88%). mp 128-130 °C (Lit. 151-152 °C);[12] R_f = 0.20 (eluent = 20% EtOAc in petroleum ether); **^{1}H NMR (500 MHz, $CDCl_3$)** δ_H: 3.02 (1H, dd, *J* 14, 7.5), 3.41 (1H, dd, *J* 14.0, 5.0), 3.72 (1H, dd, *J* 7.5.0, 5.0), 6.79 (1H, d, *J* 8.0), 6.85 (1H, d, *J* 7.5), 6.88-6.97 (3H, m), 7.04-7.13 (2H, m), 7.17 (1H, t, *J* 15.0), 7.71 (1H, br, s); **^{19}F NMR (376 MHz, $CDCl_3$)** δ_F: -116.2; **^{13}C NMR (126 MHz, $CDCl_3$)** δ_C: 35.8, 47.7, 109.9, 115.3 (d, *J* 21.2), 122.3, 124.8, 128.2, 128.8, 131.0 (d, *J* 16.2), 133.3 (d, *J* 3.2), 141.5, 161.9 (d, *J* 245), 179.5. Spectroscopic data in accordance with the literature.[12]

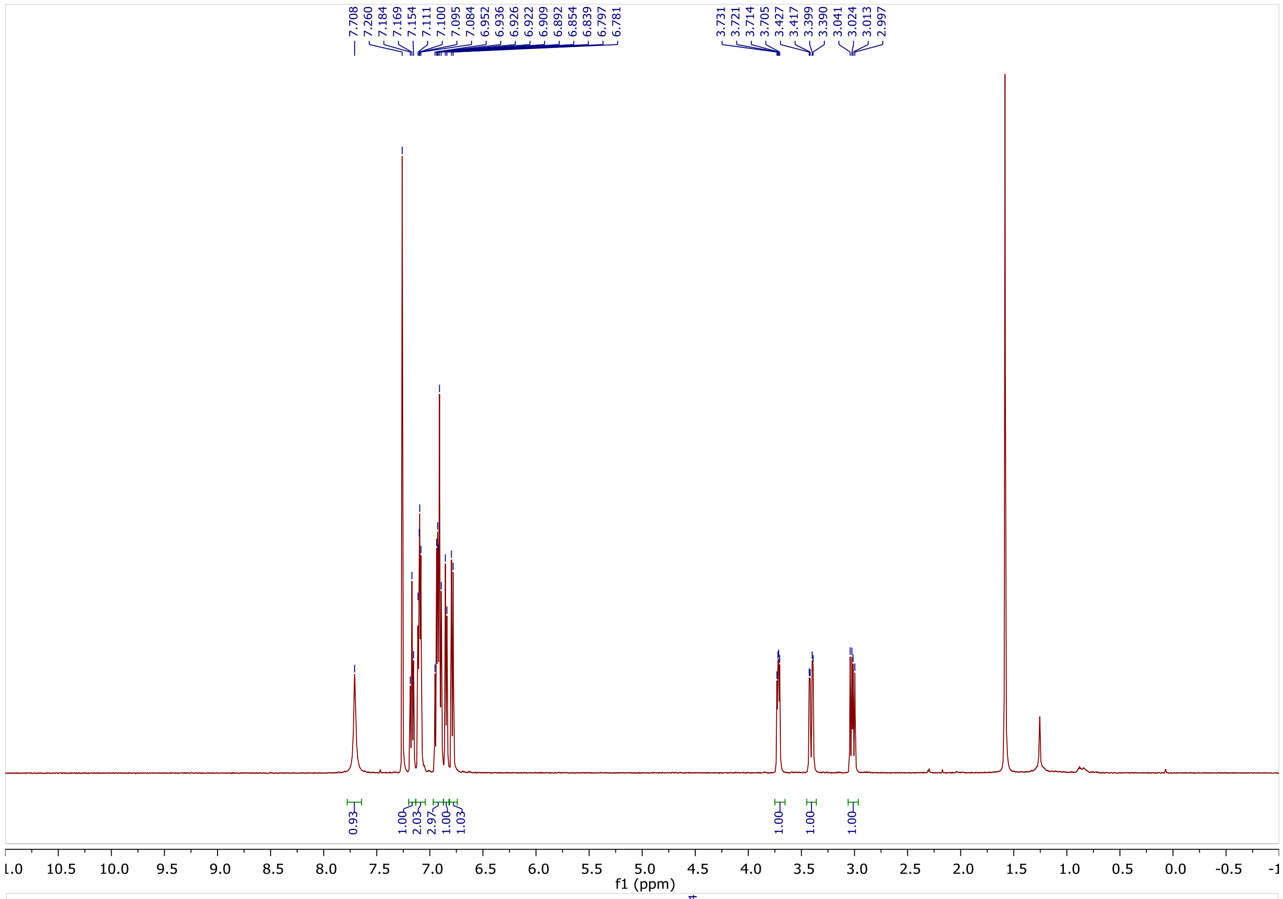

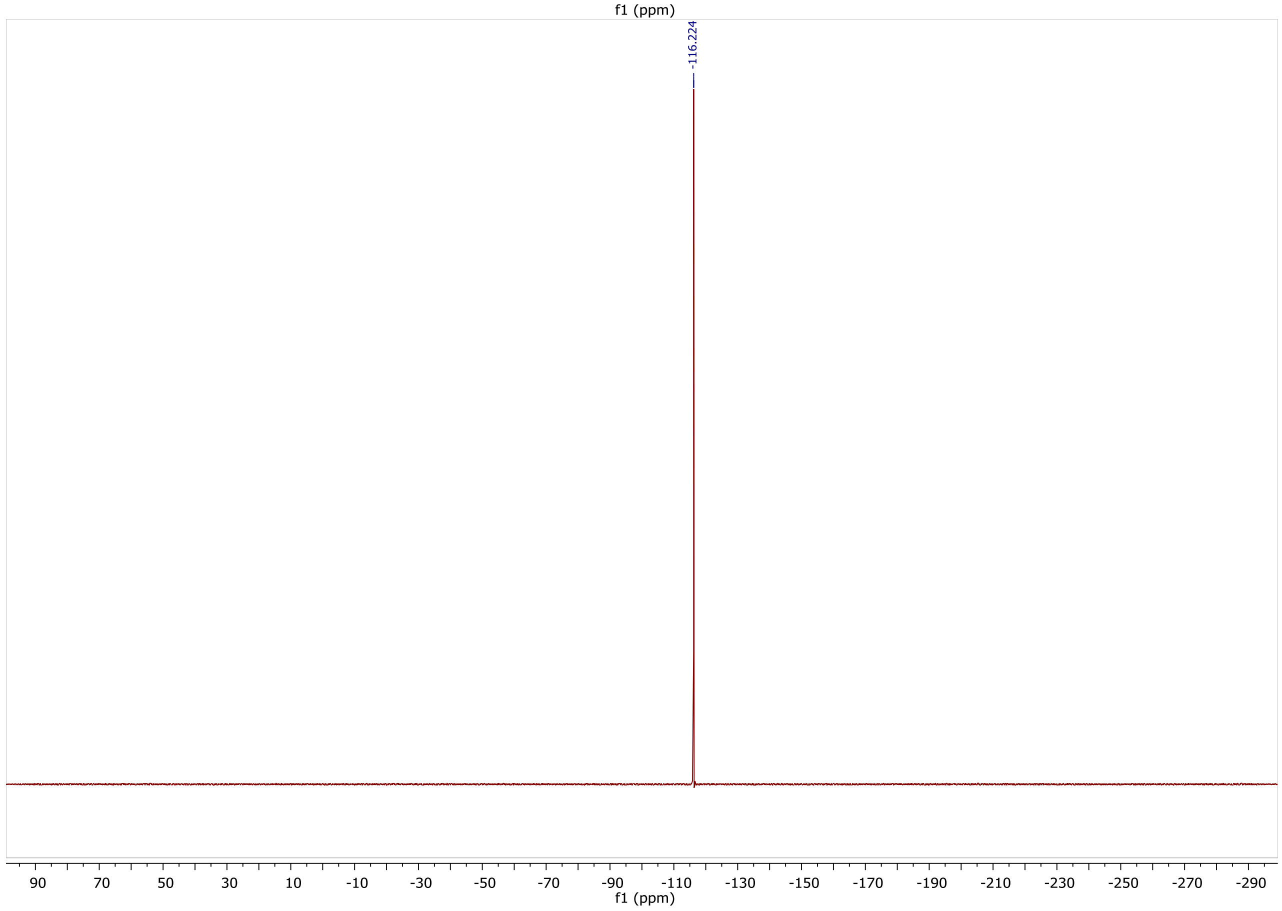

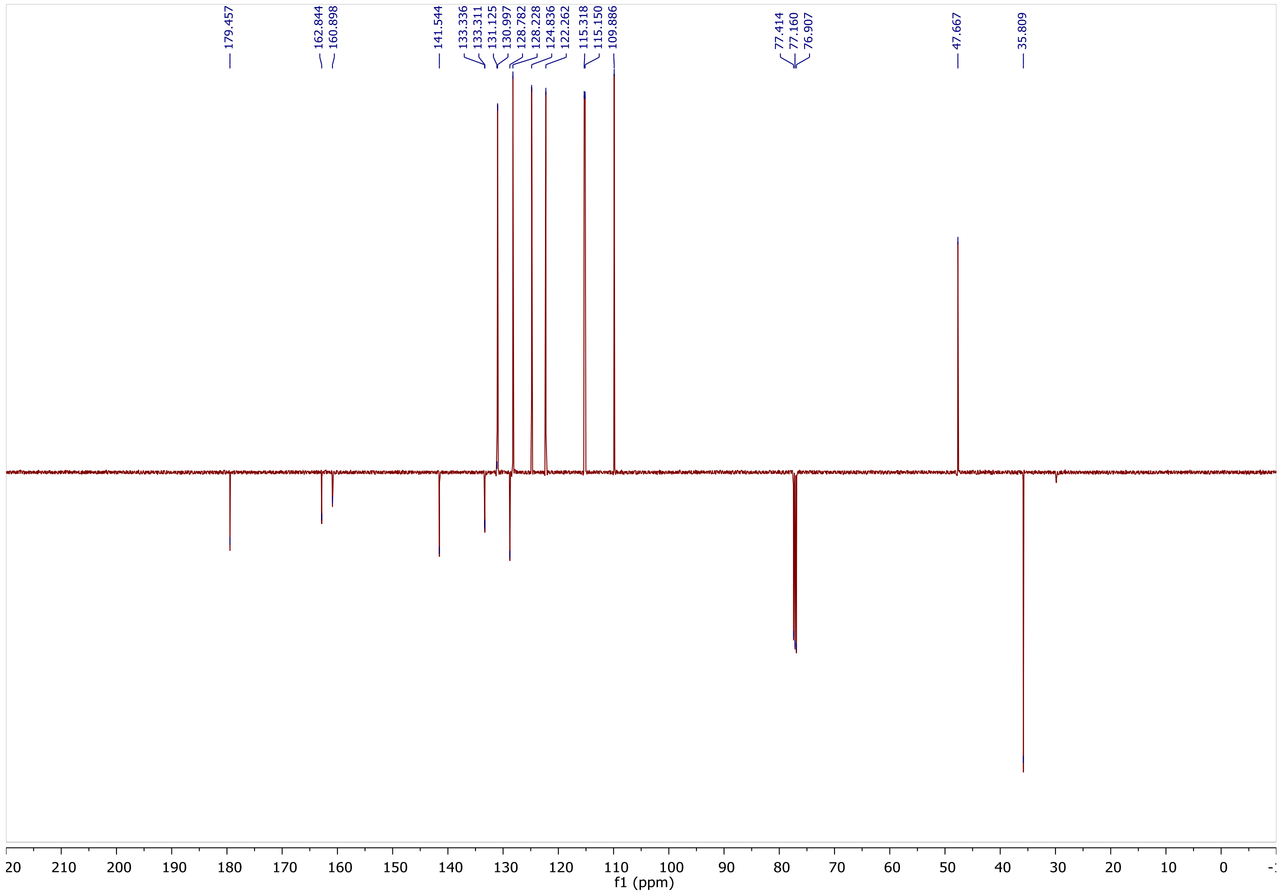

3-(furan-2-ylmethyl)indolin-2-one

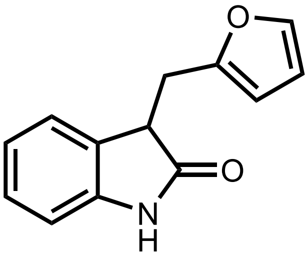

23

The title compound was prepared according to general procedure 1 using 2-hydroxymethyl furan (104 µL, 118 mg, 1.2 mmol) and oxindole (133 mg, 1.0 mmol). Purification by flash silica chromatography (eluent = 20% EtOAc in petroleum ether, 30 x 150 mm silica) gave the title compound as a pale brown solid (164 mg, 77%). mp 142-147 °C (Lit. 146-147 °C);[12] R_f = 0.52 (eluent = 30% EtOAc in petroleum ether); **^{1}H NMR (500 MHz, $CDCl_3$)** δ_H: 2.99 (1H, dd, *J* 14.5, 9.5), 3.48 (1H, dd, *J* 15.0, 4.0), 3.81 (1H, dd, *J* 9.0, 4.0), 6.03 (1H, s), 6.29 (1H, s), 6.79 (1H, d, *J* 7.0), 6.86 (1H, d, *J* 7.5), 6.94 (1H, t, *J* 8.5), 7.19 (1H, t, *J* 7.5), 7.34 (1H, s), 8.12 (1H, br s); **^{13}C NMR (126 MHz, $CDCl_3$)** δ_C: 29.2, 45.2, 107.5, 109.8, 110.5, 122.4, 124.9, 128.2, 128.9, 141.4, 141.7, 152.0, 179.2. Spectroscopic data in accordance with the literature.[12]

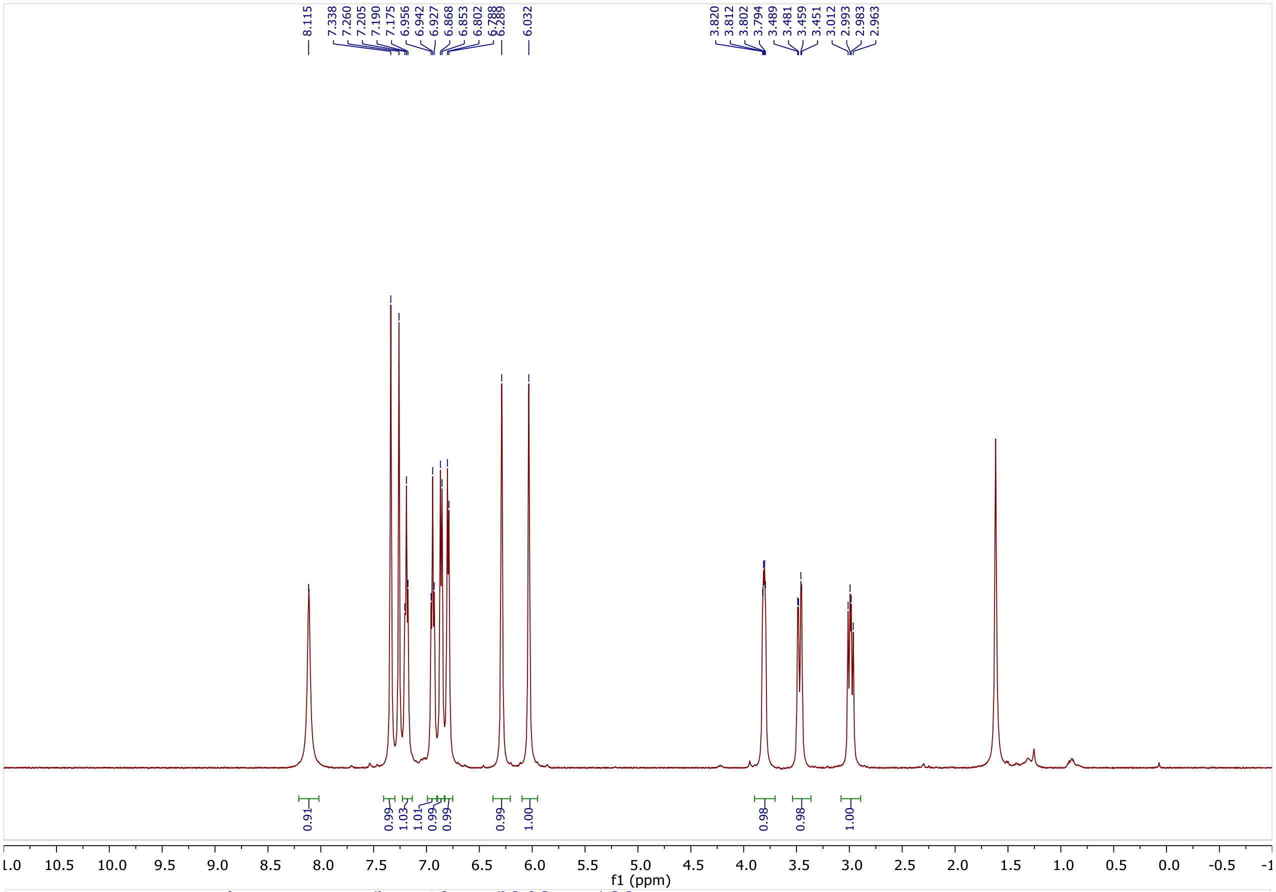
8.115
7.338
7.260
7.205
7.190
7.175
6.956
6.942
6.927
6.868
6.853
6.802
6.788
6.289
6.032
3.820
3.812
3.802
3.794
3.489
3.481
3.459
3.451
3.012
2.993
2.983
2.963
0.91
0.99
1.03
1.01
0.99
0.99
0.99
1.00
0.98
0.98
1.00
f1 (ppm)

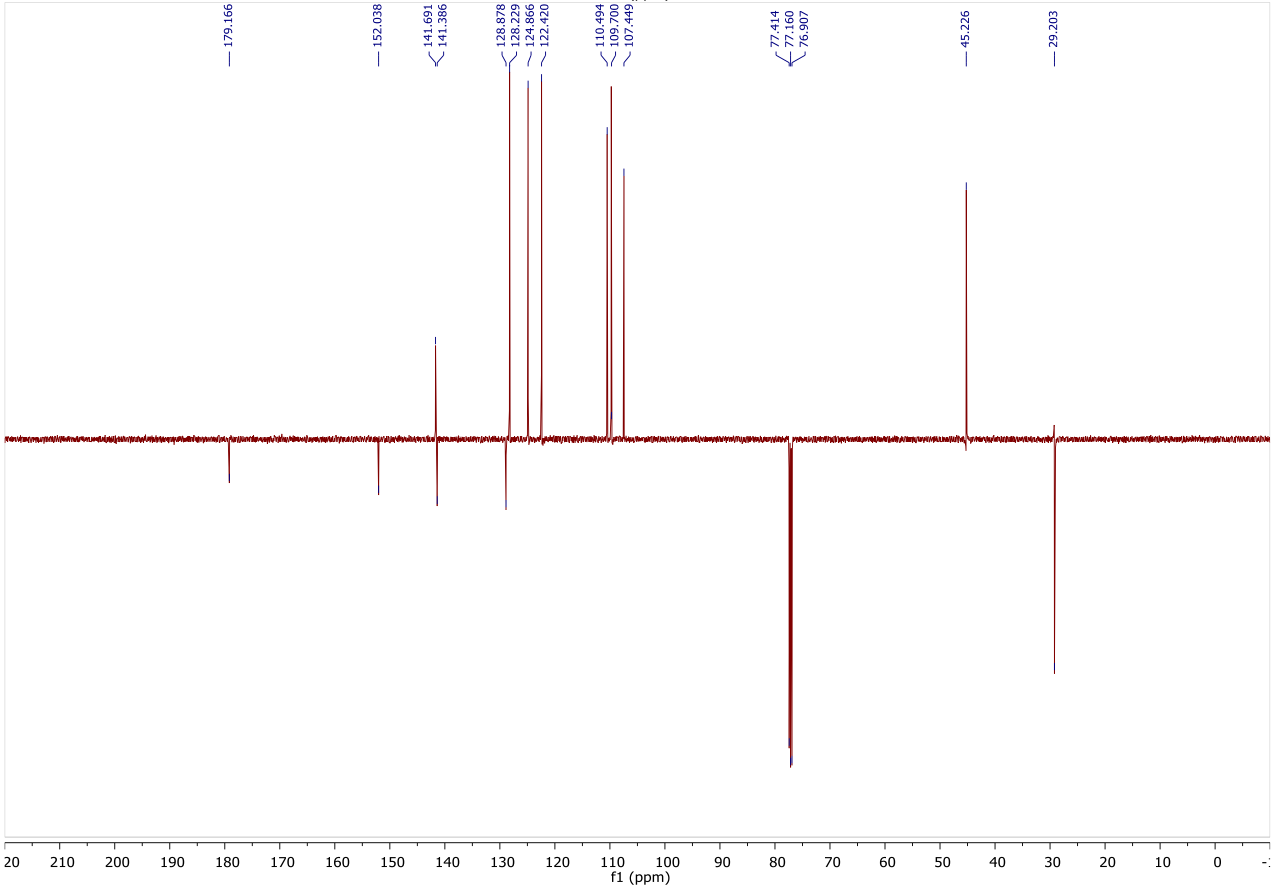
179.166
152.038
141.691
141.386
128.878
128.229
124.866
122.420
110.494
109.700
107.449
77.414
77.160
76.907
45.226
29.203
f1 (ppm)

3-(thiophen-2-ylmethyl)indolin-2-one

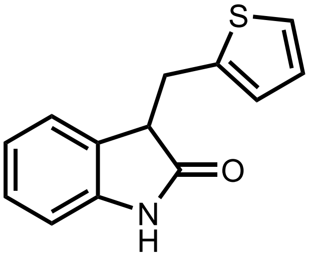

24

The title compound was prepared according to general procedure 1 using 2-hydroxymethyl thiophene (114 µL, 137 mg, 1.2 mmol) and oxindole (133 mg, 1.0 mmol). Purification by flash silica chromatography (eluent = 15% EtOAc in petroleum ether, 30 x 150 mm silica) gave the title compound as a brown solid (192 mg, 84%). mp 152-153 °C (Lit. 154-155 °C);[12] R_f = 0.50 (eluent = 20% EtOAc in petroleum ether); **^{1}H NMR (500 MHz, CDCl$_3$)** δ_H: 3.35 (1H, dd, *J* 15.0, 8.0), 3.60 (1H, dd, *J* 15.0, 4.5), 3.75 (1H, dd, *J* 7.5, 4.5), 6.75-6.83 (2H, m), 6.87 (1H, t, *J* 4.0), 6.94-7.01 (2H, m), 7.09 (1H, d, *J* 4.0), 7.19 (1H, t, *J* 5.5), 7.57 (1H, br, s); **^{13}C NMR (126 MHz, CDCl$_3$)** δ_C: 30.9, 47.6, 109.7, 122.4, 124.4, 124.9, 126.6, 126.8, 128.4, 128.7, 139.7, 141.5, 178.4. Spectroscopic data in accordance with the literature.[12]

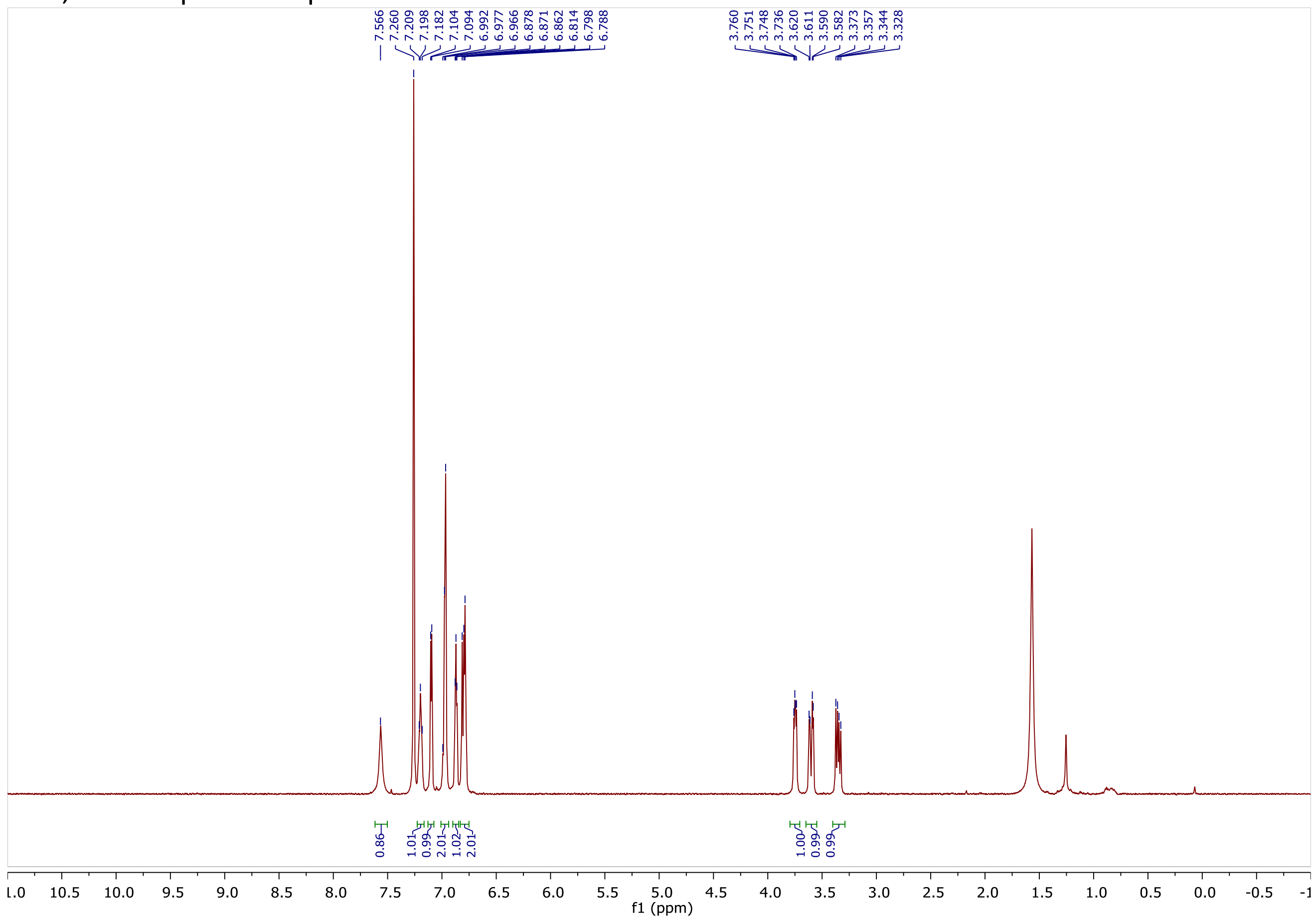

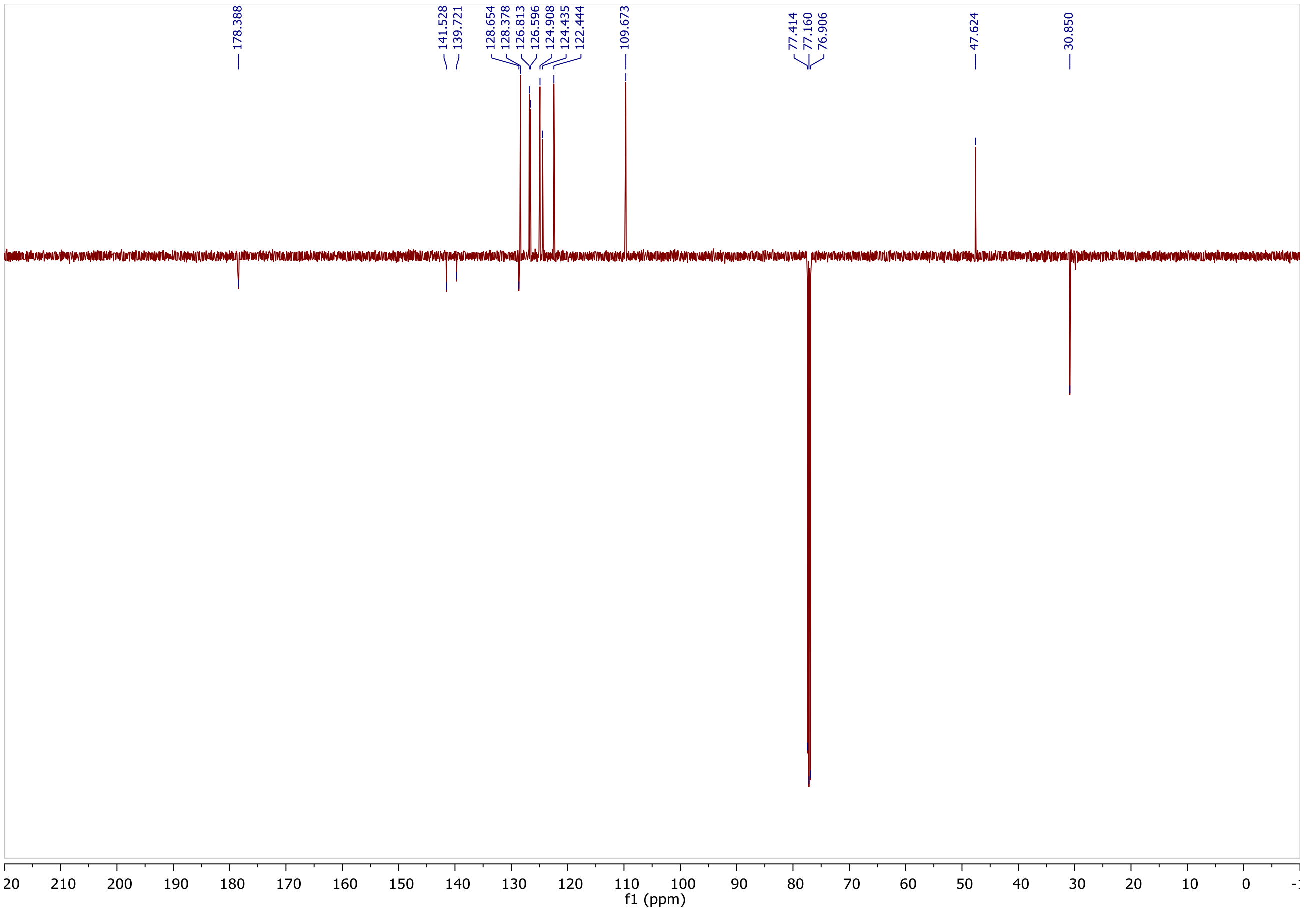

3-decylindolin-2-one

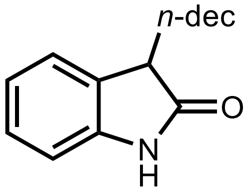

25

The title compound was prepared according to general procedure 2 using 1-decanol (2 mL) and oxindole (133 mg, 1.0 mmol). Purification by flash silica chromatography (eluent = 15% EtOAc in petroleum ether, 30 x 150 mm silica) gave the title compound as pale yellow solid (112 mg, 59%). mp 70-71°C; R_f = 0.29 (eluent = 20% EtOAc in petroleum ether); ν_{max}/cm^{-1} (film): 3177, 3065, 2911, 2851, 1697, 1616, 1470, 1344, 1319, 1223, 1173, 1152, 747, 718, 665, 584; **^{1}H NMR (500 MHz, CDCl$_3$)** δ_H: 0.87 (3H, t, *J* 7.0), 1.17-1.47 (16H, m), 1.85-2.06 (2H, m), 3.46 (1H, t, *J* 5.5), 6.88 (1H, d, *J* 7.5), 7.03 (1H, t, *J* 9.0), 7.17-7.25 (2H, m), 8.25 (1H, br s); **^{13}C NMR (126 MHz, CDCl$_3$)** δ_C: 14.3, 22.8, 25.9, 29.4, 29.5, 29.7, 29.7, 29.8, 30.7, 32.0, 46.3, 109.8, 122.3, 124.3, 127.9, 130.1, 141.7, 180.8; HRMS (**NSI$^+$**) calculated for $[C_{18}H_{28}NO]^+$ $(M + H)^+$ m/z : 274.2165, found 274.2167 (+0.6 ppm).

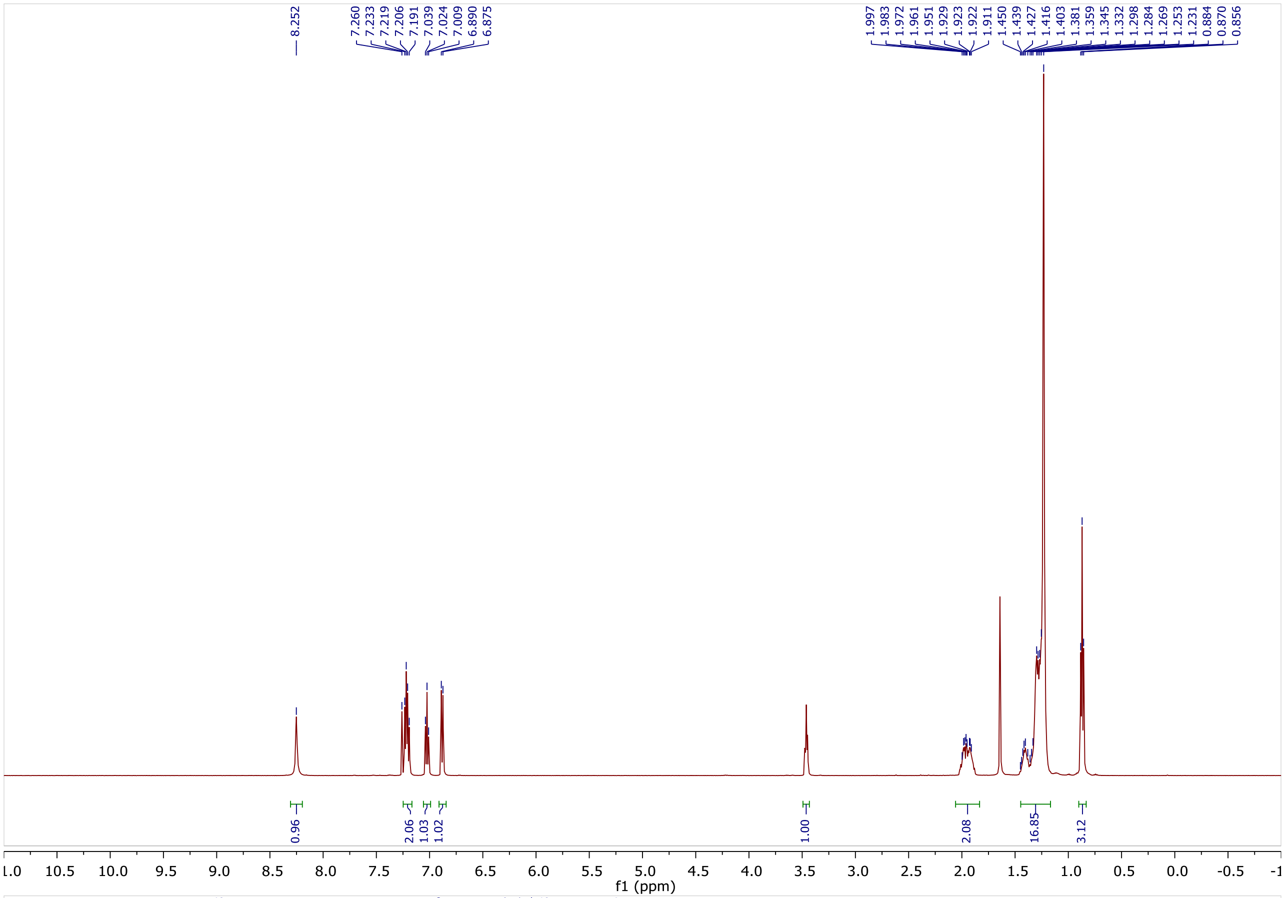

8.252
7.260
7.233
7.219
7.206
7.191
7.039
7.024
7.009
6.890
6.875
1.997
1.983
1.972
1.961
1.951
1.929
1.923
1.922
1.911
1.450
1.439
1.427
1.416
1.403
1.381
1.359
1.345
1.332
1.298
1.284
1.269
1.253
1.231
0.884
0.870
0.856
0.96
2.06
1.03
1.02
1.00
2.08
16.85
3.12
f1 (ppm)

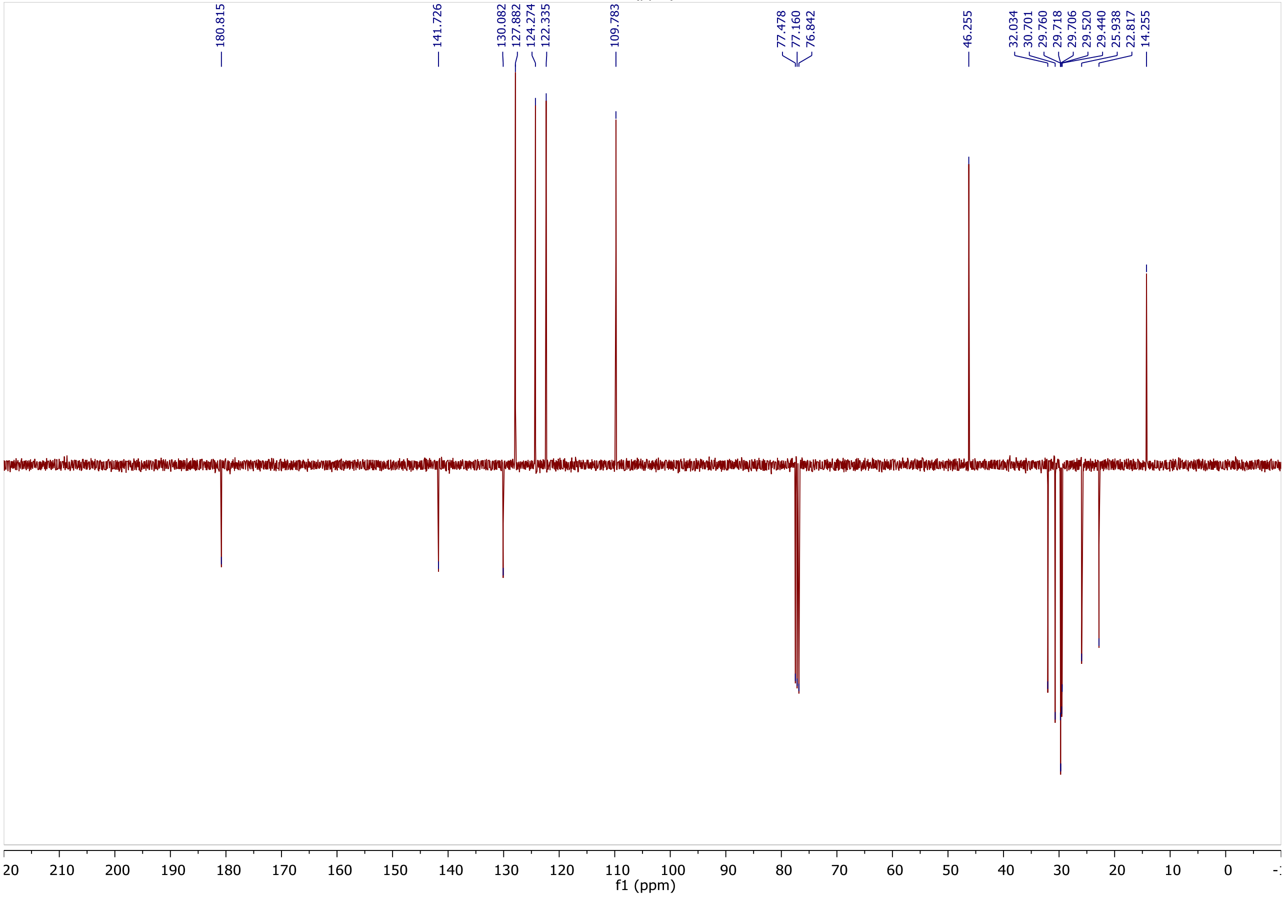

180.815
141.726
130.082
127.882
124.274
122.335
109.783
77.478
77.160
76.842
46.255
32.034
30.701
29.760
29.718
29.706
29.520
29.440
25.938
22.817
14.255
f1 (ppm)

3-butylindolin-2-one

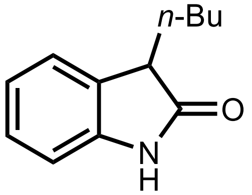

26

The title compound was prepared according to general procedure 2 using 1-butanol (2 mL) and oxindole (133 mg, 1.0 mmol). Purification by flash silica chromatography (eluent = 15% EtOAc in petroleum ether, 30 x 150 mm silica) gave the title compound as a yellow solid (159 mg, 71%). mp 59-62°C; R_f = 0.62 (eluent = 20% EtOAc in petroleum ether); ν_{max}/cm^{-1} (film): 3183, 3078, 2953, 2928, 2862, 1697, 1613, 1468, 1333, 1314, 1223, 1171, 1153, 1099, 795, 747, 702, 665, 583; **^{1}H NMR (500 MHz, CDCl$_3$)** δ_H: 0.88 (3H, t, *J* 7.0), 1.23-1.45 (4H, m), 1.87-2.05 (2H, m), 3.47 (1H, t, *J* 6.0), 6.88 (1H, d, *J* 8.0), 7.03 (1H, t, *J* 7.0), 7.17-7.25 (2H, m), 8.18 (1H, br s); **^{13}C NMR (126 MHz, CDCl$_3$)** δ_C: 14.0, 22.9, 28.0, 30.4, 46.2, 109.8, 122.4, 124.3, 127.9, 130.1, 141.7, 180.8; HRMS (**NSI$^+$**) calculated for $[C_{12}H_{16}NO]^+$ $(M + H)^+$ m/z: 190.1226, found 190.1225 (-0.7 ppm).

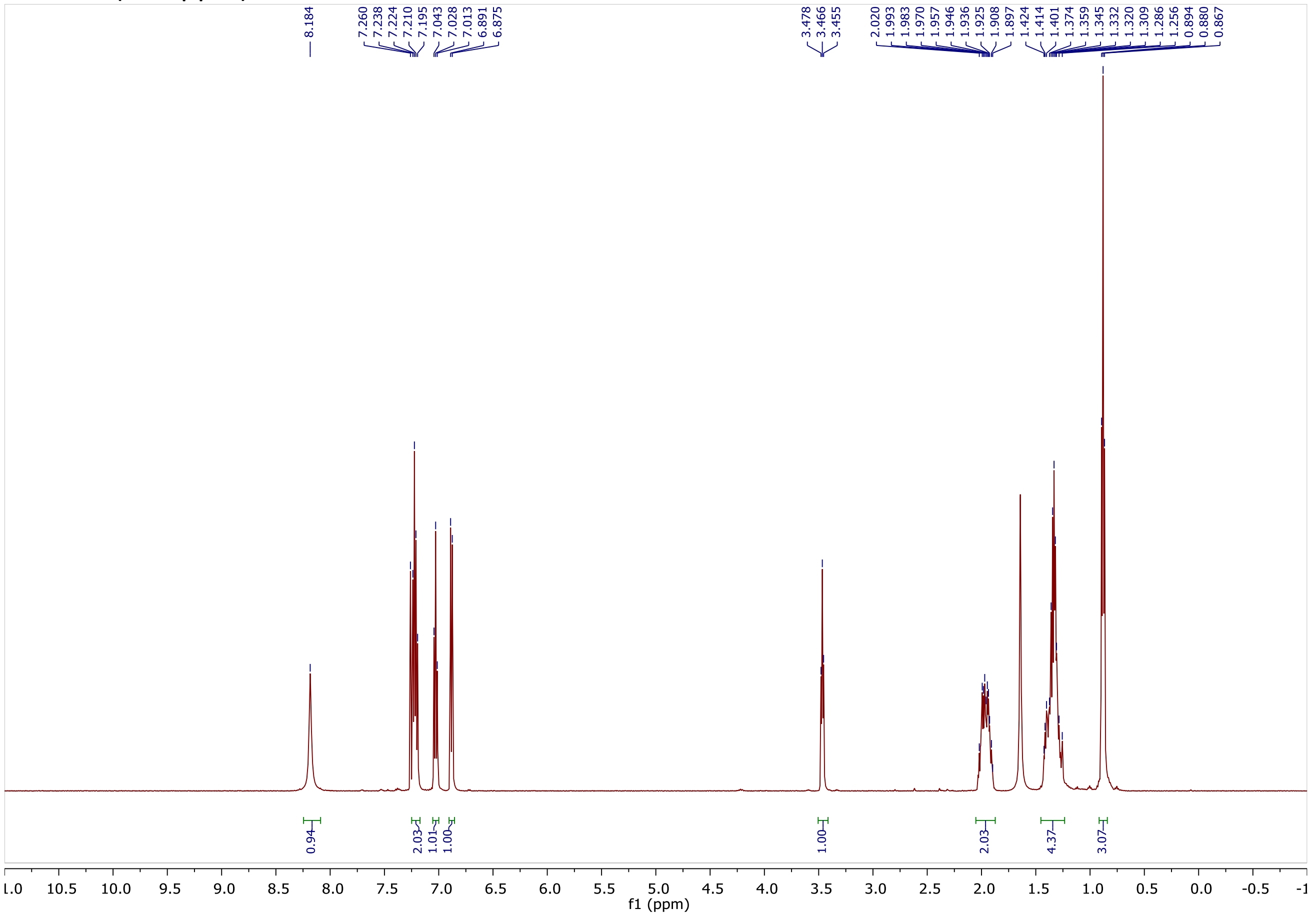

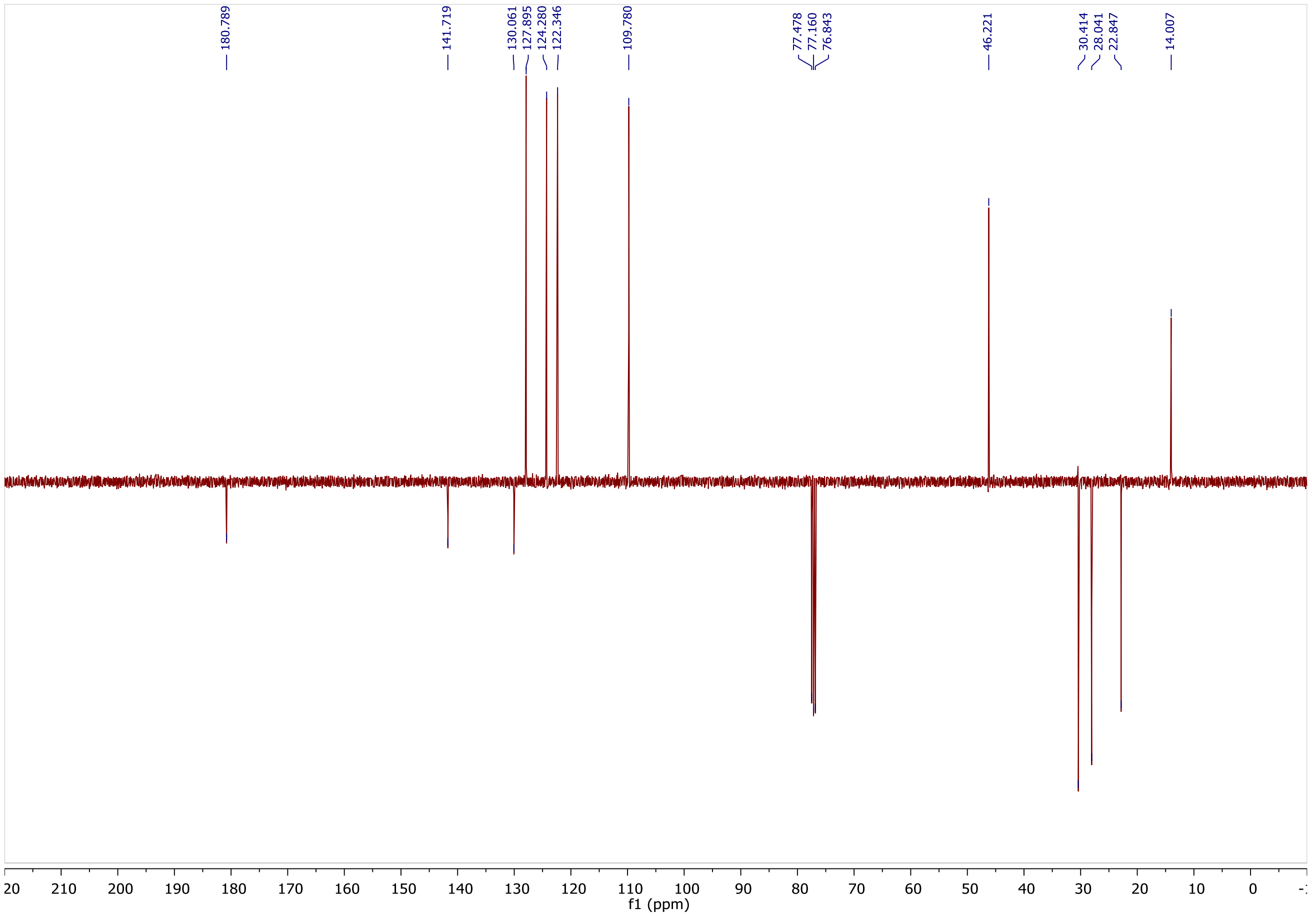

3-ethylindolin-2-one

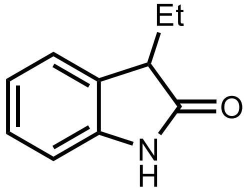

27

The title compound was prepared according to general procedure 2 using ethanol (2 mL) and oxindole (133 mg, 1.0 mmol). Purification by flash silica chromatography (eluent = 20% EtOAc in petroleum ether, 30 x 150 mm silica) gave the title compound as a cream solid (126 mg, 79%). mp 98-100 °C (Lit. 99-101°C);[13] R_f = 0.23 (eluent = 20% EtOAc in petroleum ether); **^{1}H NMR (400 MHz, $CDCl_3$)** δ_H: 0.93 (3H, t, *J* 7.2), 1.94-2.16 (2H, m), 3.46 (1H, t, *J* 5.6), 6.91 (1H, d, *J* 6.8), 7.03 (1H, t, *J* 8.0), 7.17-7.25 (2H, m), 9.01 (1H, br s); **^{13}C NMR (101 MHz, $CDCl_3$)** δ_C: 10.1, 23.7, 47.4, 109.9, 122.3, 124.2, 127.9, 129.7, 142.0, 181.1. Spectroscopic data in accordance with the literature.[13]

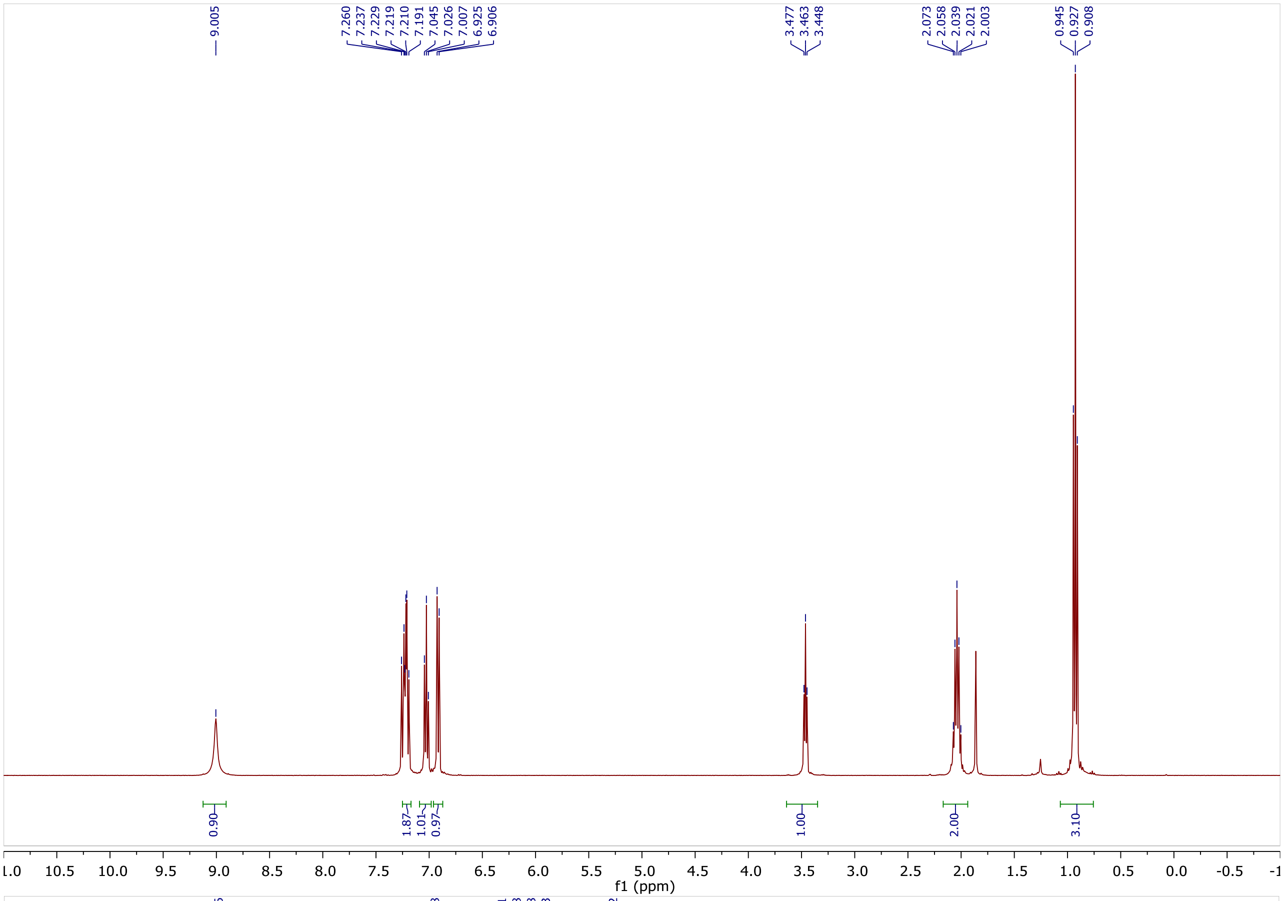
9.005
7.260
7.237
7.229
7.219
7.210
7.191
7.045
7.026
7.007
6.925
6.906
3.477
3.463
3.448
2.073
2.058
2.039
2.021
2.003
0.945
0.927
0.908
0.90
1.87
1.01
0.97
1.00
2.00
3.10
f1 (ppm)

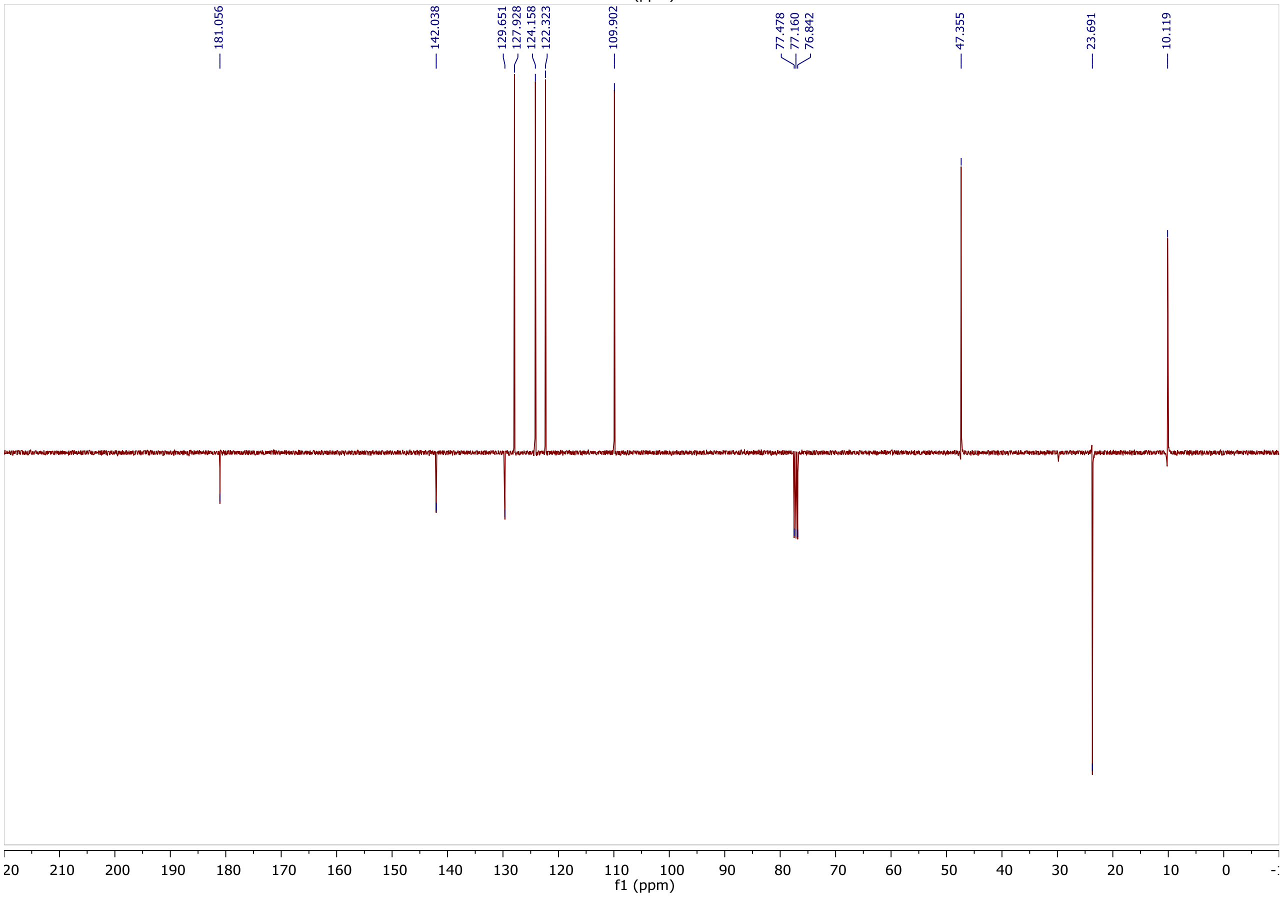
181.056
142.038
129.651
127.928
124.158
122.323
109.902
77.478
77.160
76.842
47.355
23.691
10.119
f1 (ppm)

3-methylindolin-2-one

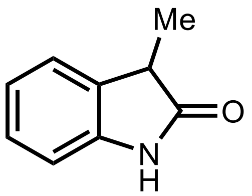

28

The title compound was prepared according to general procedure 2 using methanol (2 mL) and oxindole (133 mg, 1.0 mmol). Purification by flash silica chromatography (eluent = 20% EtOAc in petroleum ether, 30 x 150 mm silica) gave the title compound as a pale brown solid (90 mg, 61%). mp 117-119 °C, (Lit. 119-120°C);[14] R_f = 0.24 (eluent = 20% EtOAc in hexanes); **^{1}H NMR (400 MHz, $CDCl_3$)** δ_H: 1.51 (3H, d, *J* 7.6), 3.47 (1H, q, *J* 7.6), 6.89 (1H, d, *J* 7.6), 7.03 (1H, dt, *J* 8.3, 1.0), 7.16-7.26 (2H, m), 8.46 (1H, br s); **^{13}C NMR (101 MHz, $CDCl_3$)** δ_C: 15.4, 41.3, 109.9, 122.5, 123.9, 128.0, 131.4, 141.4, 181.8. Spectroscopic data in accordance with the literature.[14]

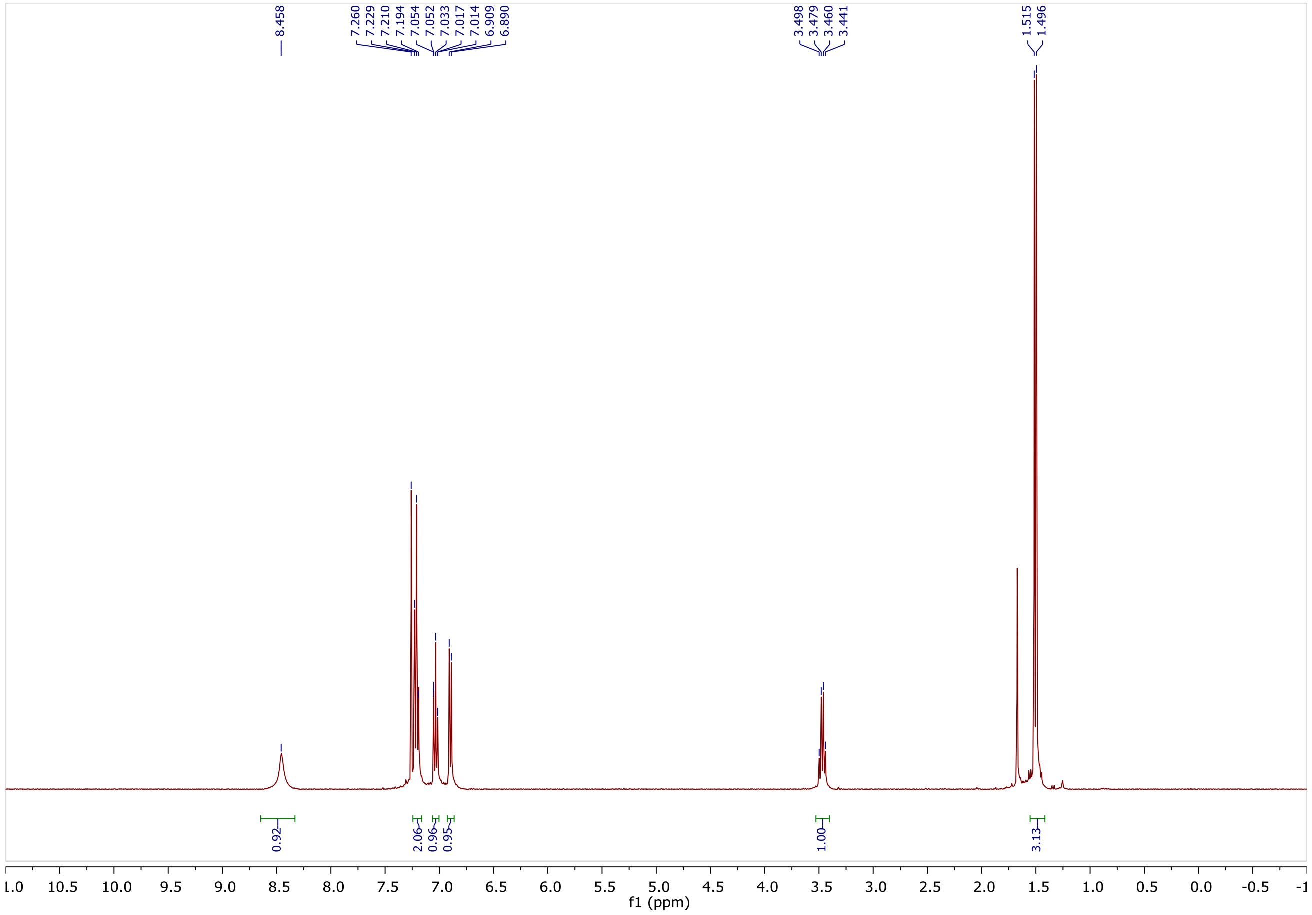

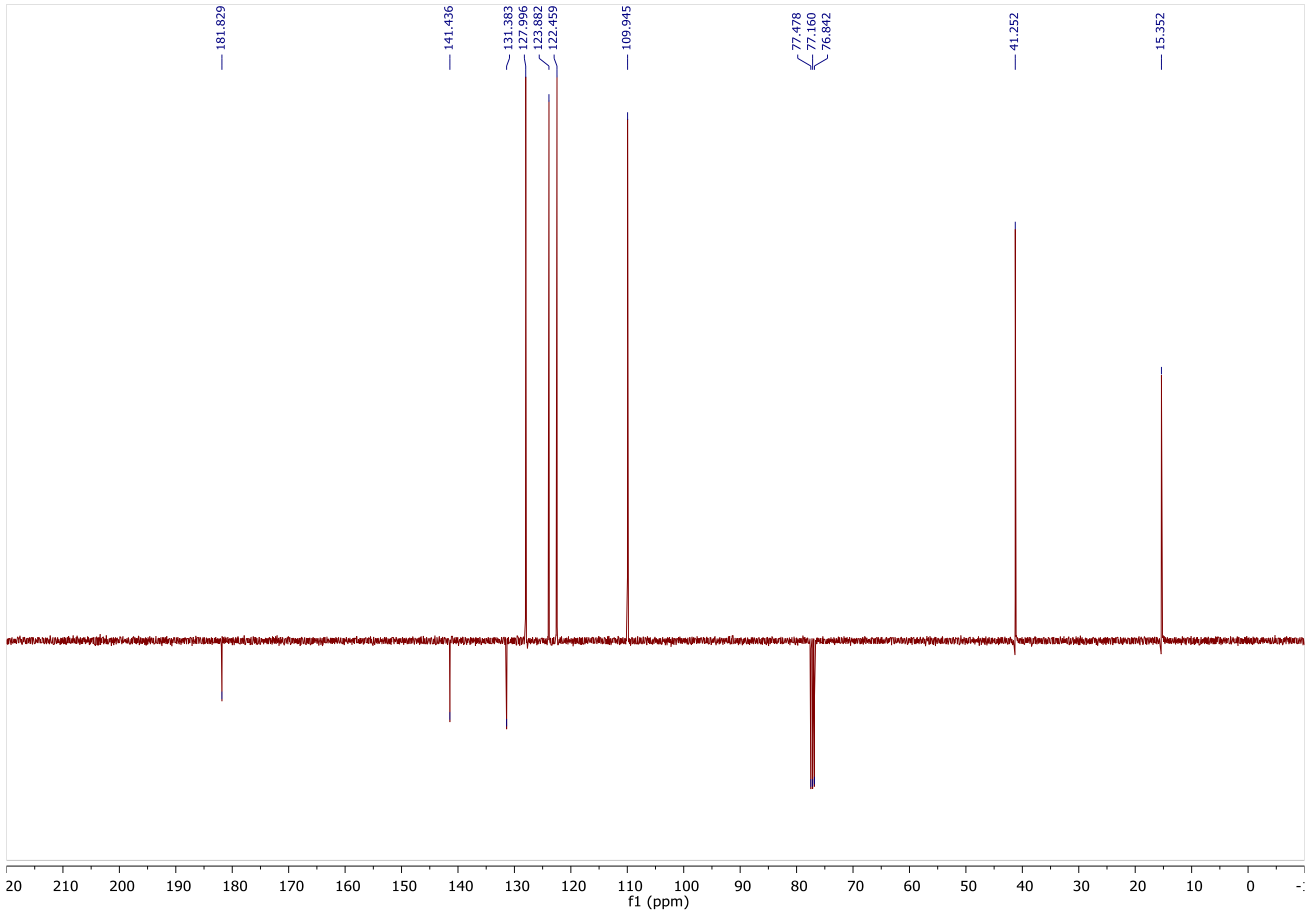

3-(4-hydroxybutyl)indolin-2-one

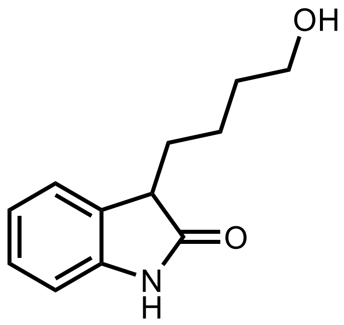

29

The title compound was prepared according to general procedure 2 using 1,4-butanediol (2 mL) and oxindole (133 mg, 1.0 mmol). Purification by flash silica chromatography (eluent = 60% EtOAc in hexanes, 30 x 150 mm silica) gave the title compound as a viscous oil (133 mg, 53%). R_f = 0.42 (eluent = 50% EtOAc in hexanes); ν_{max}/cm^{-1} (film): 3262, 2938, 2864, 1701, 1620, 1487, 1472, 1339, 1227, 1173, 1104, 1018, 752 ; **^{1}H NMR (400 MHz, CDCl$_3$)** δ_H: 1.34-1.53 (2H, m), 1.58 (2H, q, *J* 7.0), 1.74 (1H, br s), 1.93-2.08 (2H, m), 3.49 (1H, t, *J* 5.6), 3.61 (2H, t, *J* 6.4), 6.89 (1H, d, *J* 7.6), 7.02 (1H, dt, *J* 7.6, 0.8), 7.16-7.25 (2H, m), 8.61 (1H, br s); **^{13}C NMR (101 MHz, CDCl$_3$)** δ_C: 22.1, 30.3, 32.7, 46.1, 62.6, 109.8, 122.5, 124.3, 128.0, 129.7, 141.7, 180.6; HRMS (**ES$^+$**) calculated for $[C_{12}H_{16}NO_2]^+$ $(M+H)^+$ m/z: 206.1181, found 206.1179 (-1.0 ppm).

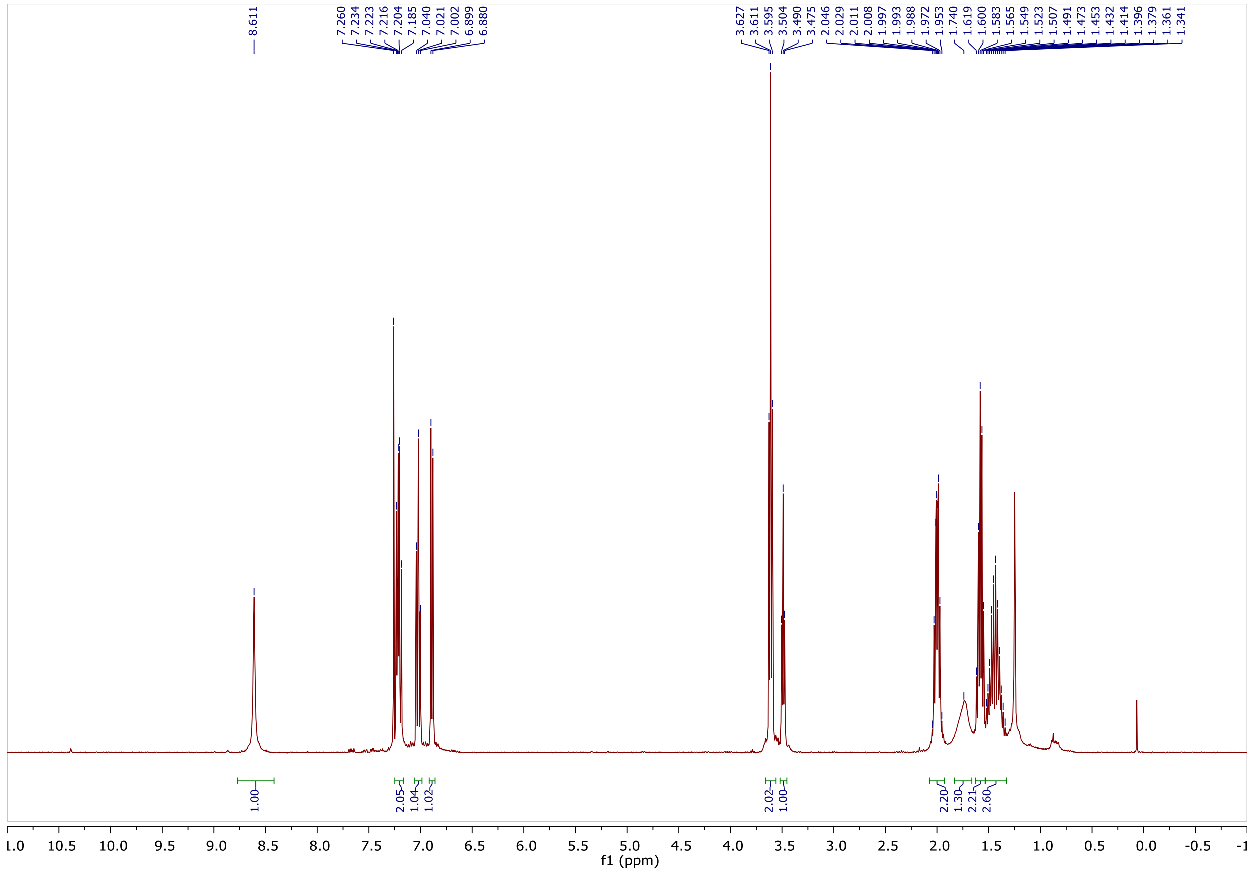

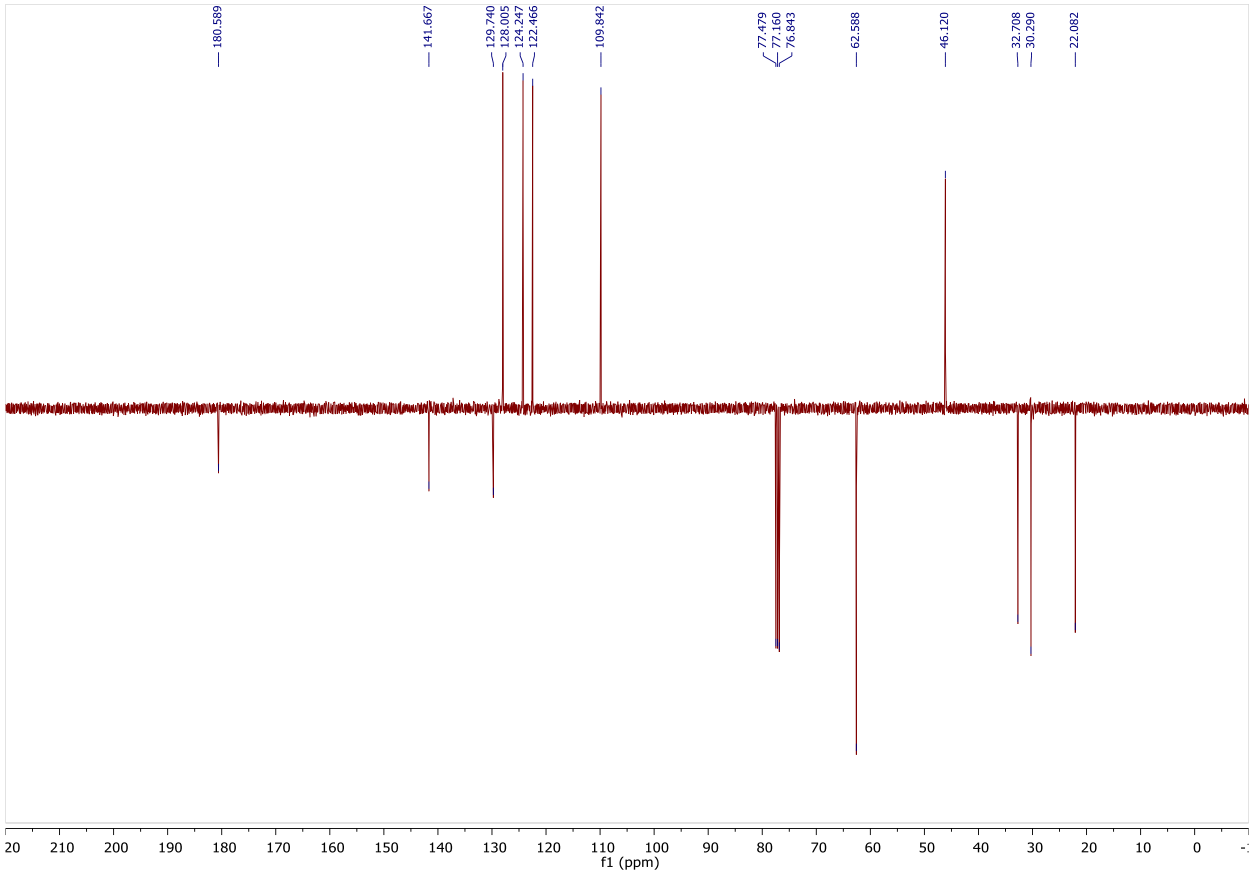

3-isopropylindolin-2-one

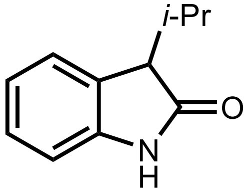

30

The title compound was prepared according to general procedure 2 using 2-propanol (2 mL) and oxindole (133 mg, 1.0 mmol). Purification by flash silica chromatography (eluent = 15% EtOAc in petroleum ether, 30 x 150 mm silica) gave the title compound as an orange solid (140 mg, 80%). mp 102-106 °C (Lit. 102-106 °C);[12] R_f = 0.36 (eluent = 20% EtOAc in petroleum ether); **^{1}H NMR (400 MHz, CDCl$_3$)** δ_H: 0.93 (3H, d, *J* 6.8), 1.21 (3H, d, *J* 7.2), 2.43-2.57 (1H, m), 3.40 (1H, d, *J* 3.6), 6.88 (1H, d, *J* 8.0), 7.01 (1H, dt, *J* 7.6, 1.2), 7.17-7.25 (2H, m), 8.32 (1H, br s); **^{13}C NMR (101 MHz, CDCl$_3$)** δ_C: 18.1, 20.0, 30.9, 52.3, 109.7, 122.2, 124.8, 128.0, 128.5, 142.1, 180.1. Spectroscopic data in accordance with the literature.[12]

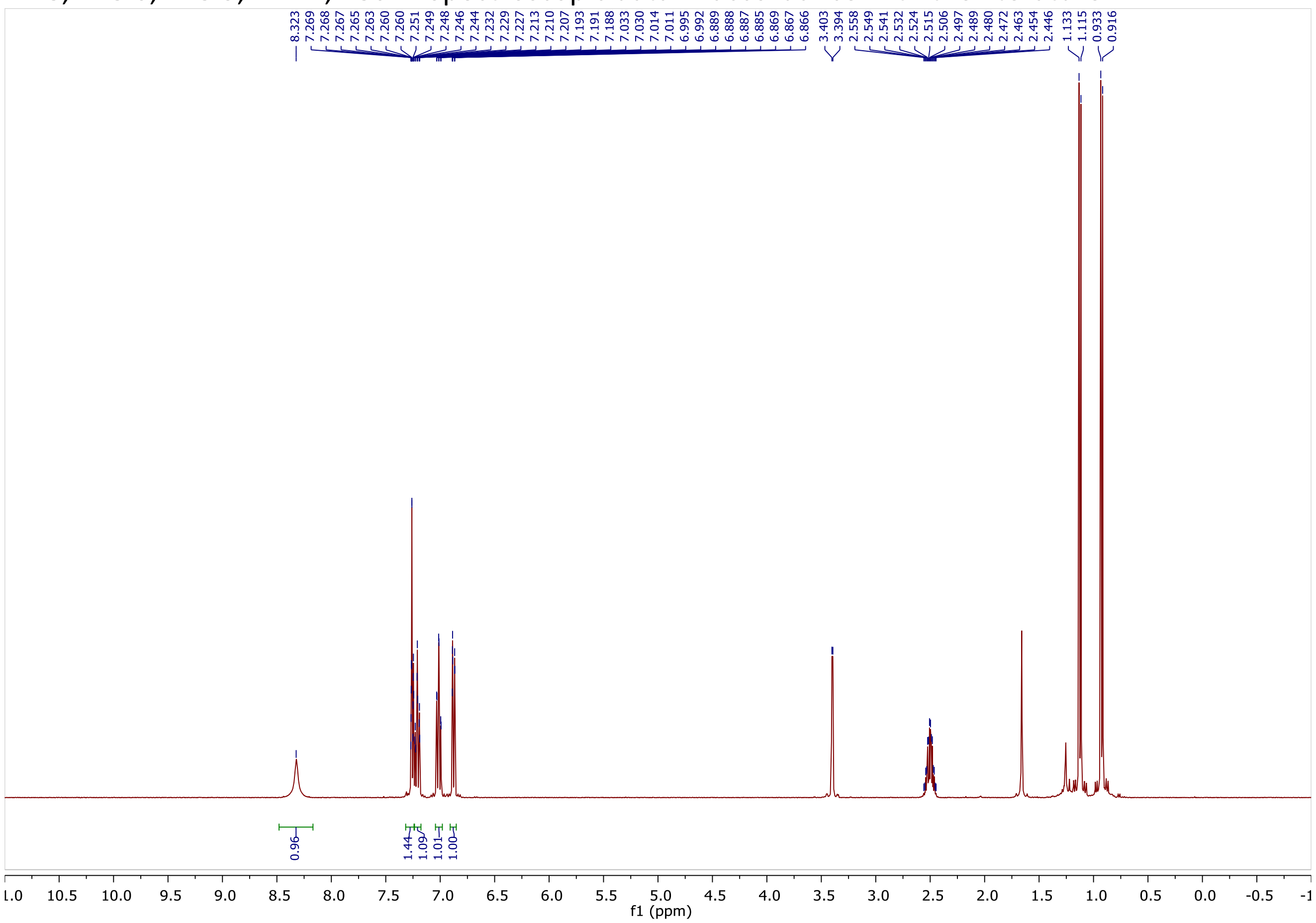

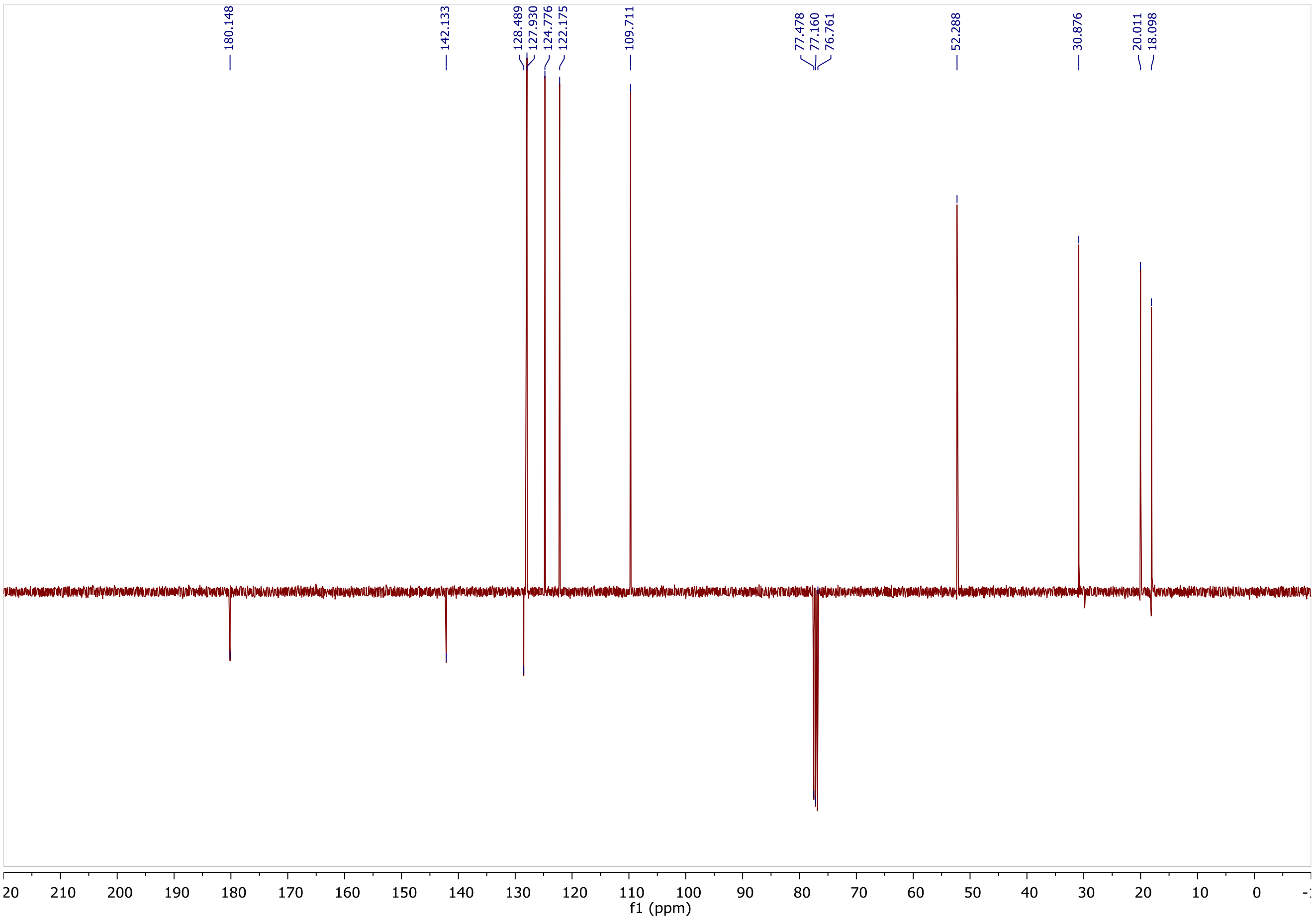

3-(sec-butyl)indoline

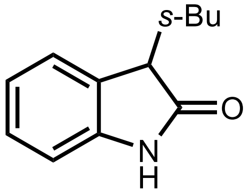

31

The title compound was prepared according to general procedure 2 using [Fe] precatalyst **3** (18.2 mg, 0.04 mmol, 4 mol %), PPh_3 (21 mg, 0.08 mmol, 8 mol %), 2-butanol (2 mL) and oxindole (133 mg, 1.0 mmol); giving the crude product after work up (56:44 *dr*). Purification by flash silica chromatography (eluent = 15% EtOAc in petroleum ether, 30 x 150 mm silica) gave the title compound as an inseparable mixture of diastereomers, as a white solid (159 mg, 84%, 3:1 *dr*); mp 114-118 °C; R_f = 0.30 (eluent = 20% EtOAc in petroleum ether); ν_{max}/cm^{-1} (film): 3158, 2957, 2928, 2870, 1703, 1663, 1620, 1487, 1466, 1333, 1227, 1018, 943, 743, 665, 625, 579; Data for mixture of diastereomers: **^{1}H NMR (500 MHz, $CDCl_3$)** δ_H: 0.78 (2H, d, *J* 6.5), 0.93 (1H, t, *J* 7.5), 0.98-1.08 (3H, m), 1.48-1.68 (2H, m), 2.15-2.32 (1H, m), 3.44-3.55 (1H, m), 6.86-6.92 (1H, m), 6.97-7.04 (1H, m), 7.16-7.25 (2H, m), 8.57-8.71 (1H, m); **^{13}C NMR (126 MHz, $CDCl_3$)** δ_C: 12.3, 12.4, 15.1, 16.6, 25.8, 27.5, 37.5, 37.8, 50.7, 51.2, 109.6, 109.7, 122.1, 122.2, 124.4, 125.0, 127.8, 127.9, 128.1, 129.2, 142.0, 142.2, 180.0, 180.6; HRMS (**NSI$^+$**) calculated for $[C_{12}H_{16}NO]^+$ $(M + H)^+$ m/z: 190.1226, found 190.1225 (-0.7 ppm).

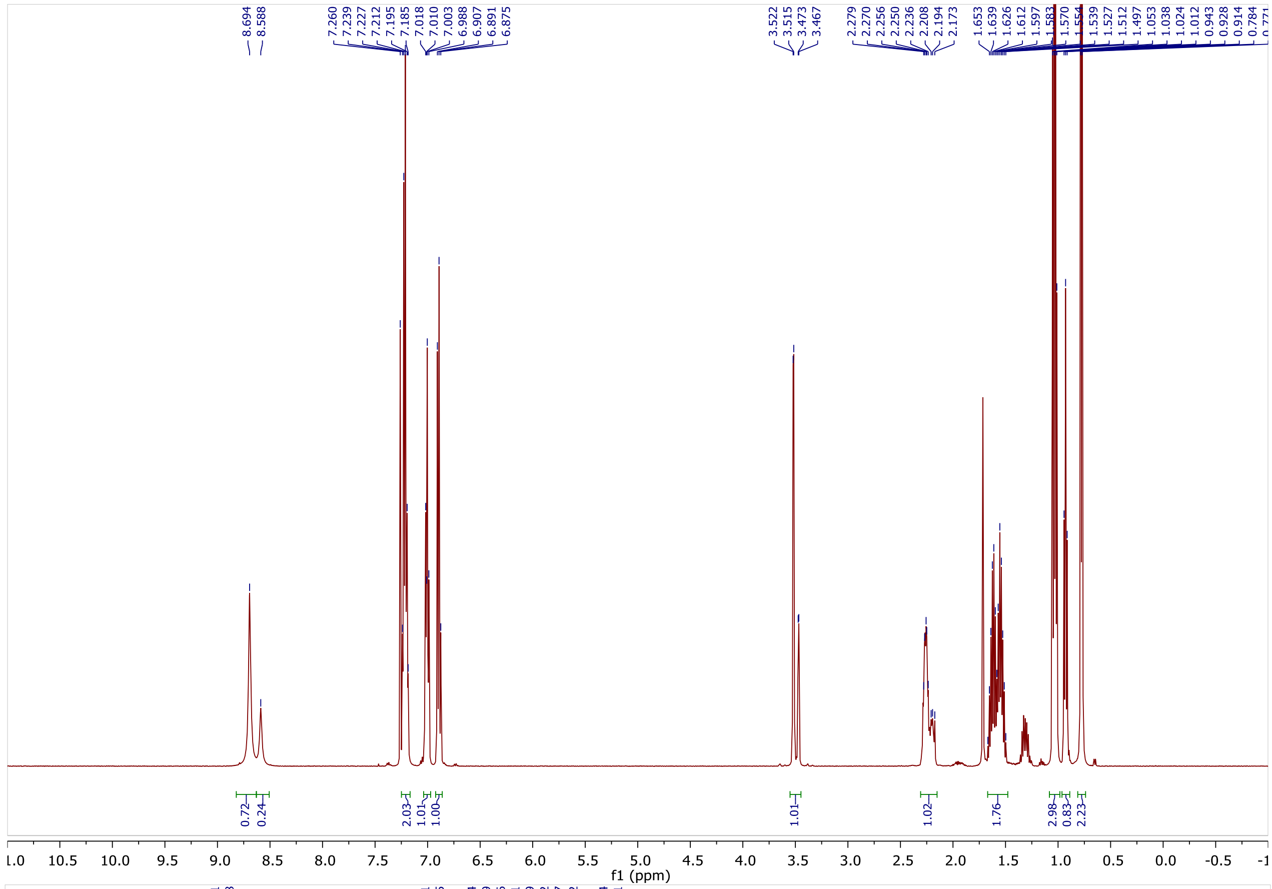

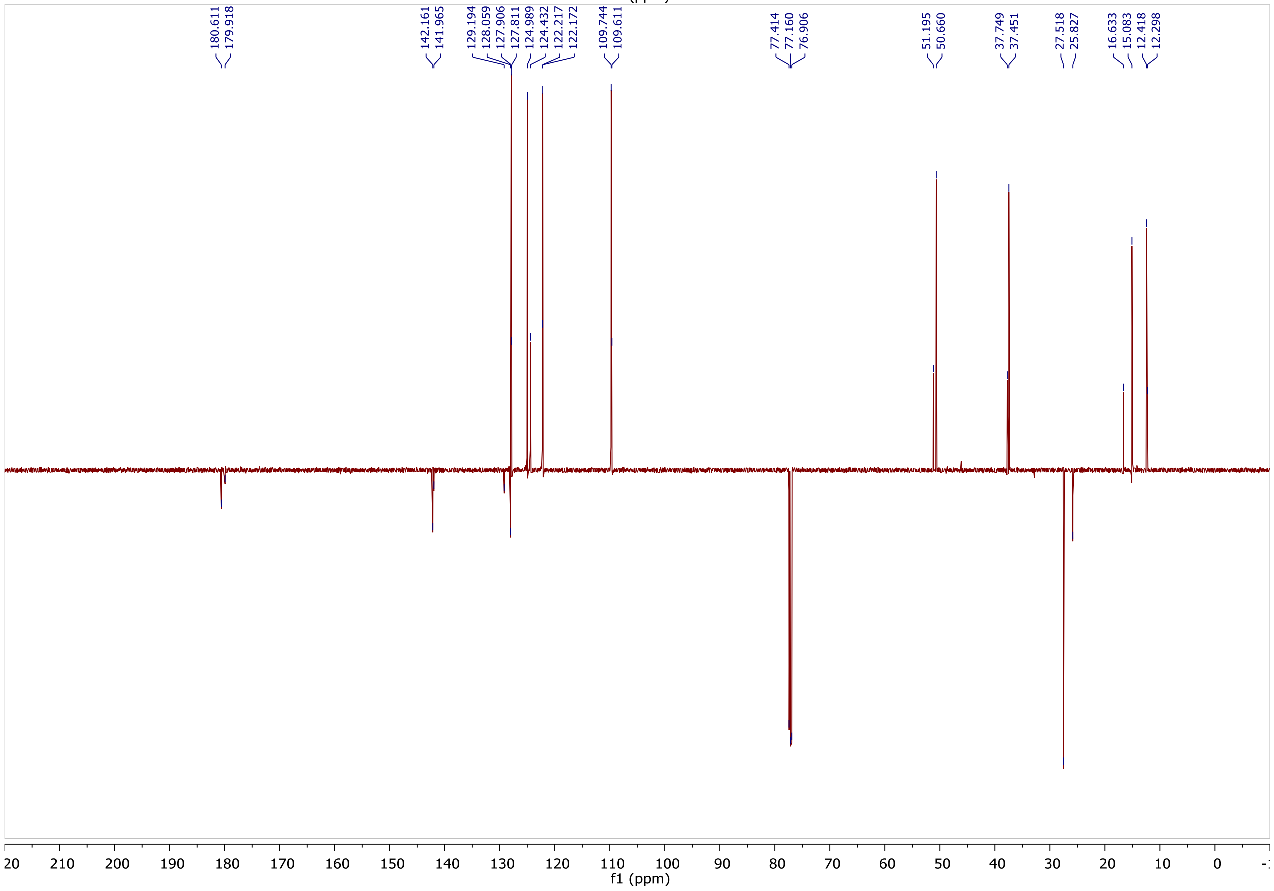

3-benzyl-5-bromoindolin-2-one

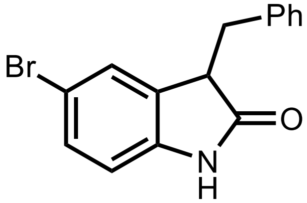

32

The title compound was prepared according to general procedure 1 using benzyl alcohol (124 µL, 130 mg, 1.2 mmol) and 5-bromo-2-oxindole (212 mg, 1.0 mmol). Purification by flash silica chromatography (eluent = 20% EtOAc in hexanes, 30 x 150 mm silica) gave the title compound as a yellow-brown solid (246 mg, 80%); mp 133-134 °C (Lit. 138-140 °C);[13] R_f = 0.20 (eluent = 20% EtOAc in hexanes); **^{1}H NMR (500 MHz, $CDCl_3$)** δ_H: 2.90 (1H, dd, *J* 14.0, 9.5), 3.41 (1H, dd, *J* 13.5, 4.5), 3.70 (1H, dd, *J* 8.5, 4.5), 6.70 (1H, d, *J* 8.0), 6.82 (1H, s), 7.12 (2H, d, *J* 7.0), 7.16-7.30 (4H, m), 9.24 (1H, br s); **^{13}C NMR (126 MHz, $CDCl_3$)** δ_C: 36.6, 47.8, 111.3, 114.8, 127.1, 128.1, 128.6, 129.5, 131.0, 131.1, 137.2, 140.6, 179.4. Spectroscopic data in accordance with the literature.[13]

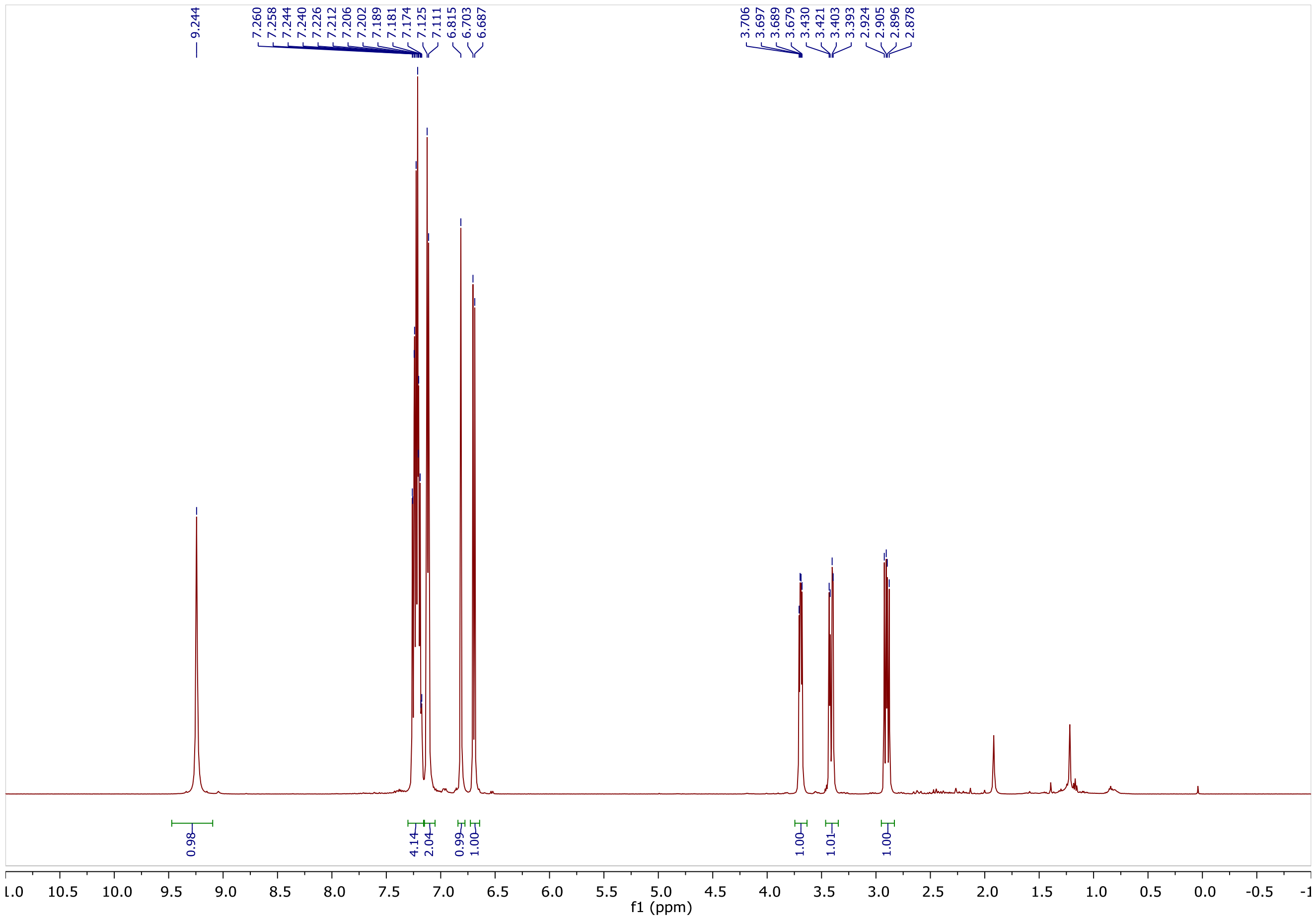

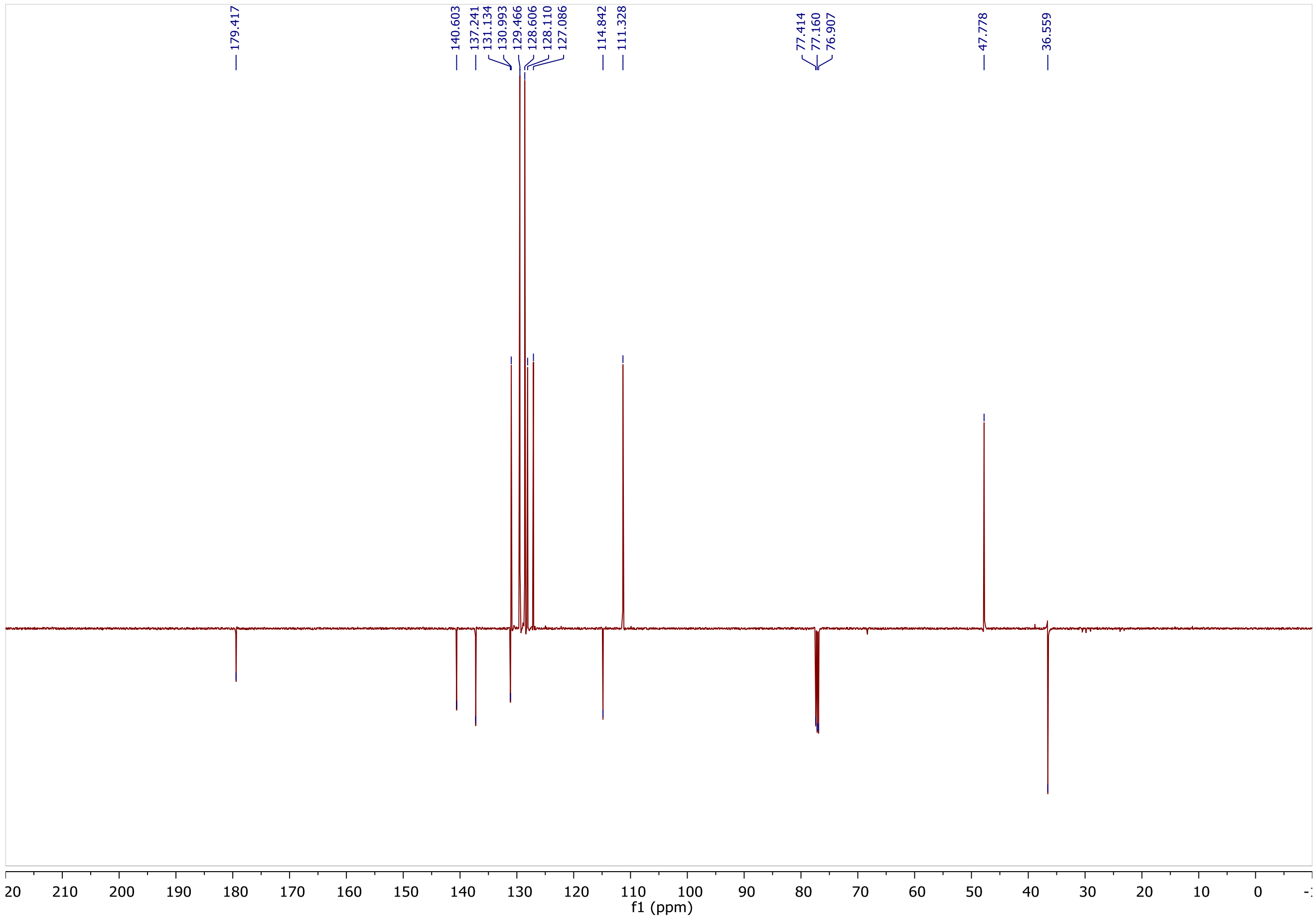

3-benzyl-5-chloroindolin-2-one

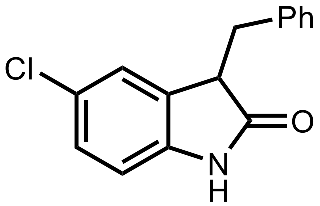

33

The title compound was prepared according to general procedure 1 using benzyl alcohol (124 µL, 130 mg, 1.2 mmol) and 5-chloro-2-oxindole (168 mg, 1.0 mmol). Purification by flash silica chromatography (eluent = 20% EtOAc in hexanes, 30 x 150 mm silica) gave the title compound as a yellow solid (236 mg, 92%); mp 106-109 °C (Lit. 115-116 °C);[13] R_f = 0.26 (eluent = 20% EtOAc in hexanes); **^{1}H NMR (500 MHz, $CDCl_3$)** δ_H: 2.94 (1H, dd, *J* 13.5, 9.0), 3.43 (1H, dd, *J* 13.5, 4.5), 3.71 (1H, dd, *J* 8.5, 4.5), 6.72 (2H, d, *J* 8.5), 7.08-7.18 (3H, m), 7.18-7.26 (3H, m), 8.39 (1H, br s); **^{13}C NMR (126 MHz, $CDCl_3$)** δ_C: 36.6, 47.9, 110.8, 125.3, 127.1, 127.5, 128.1, 128.6, 129.5, 130.8, 137.3, 140.2, 179.6. Spectroscopic data in accordance with the literature.[13]

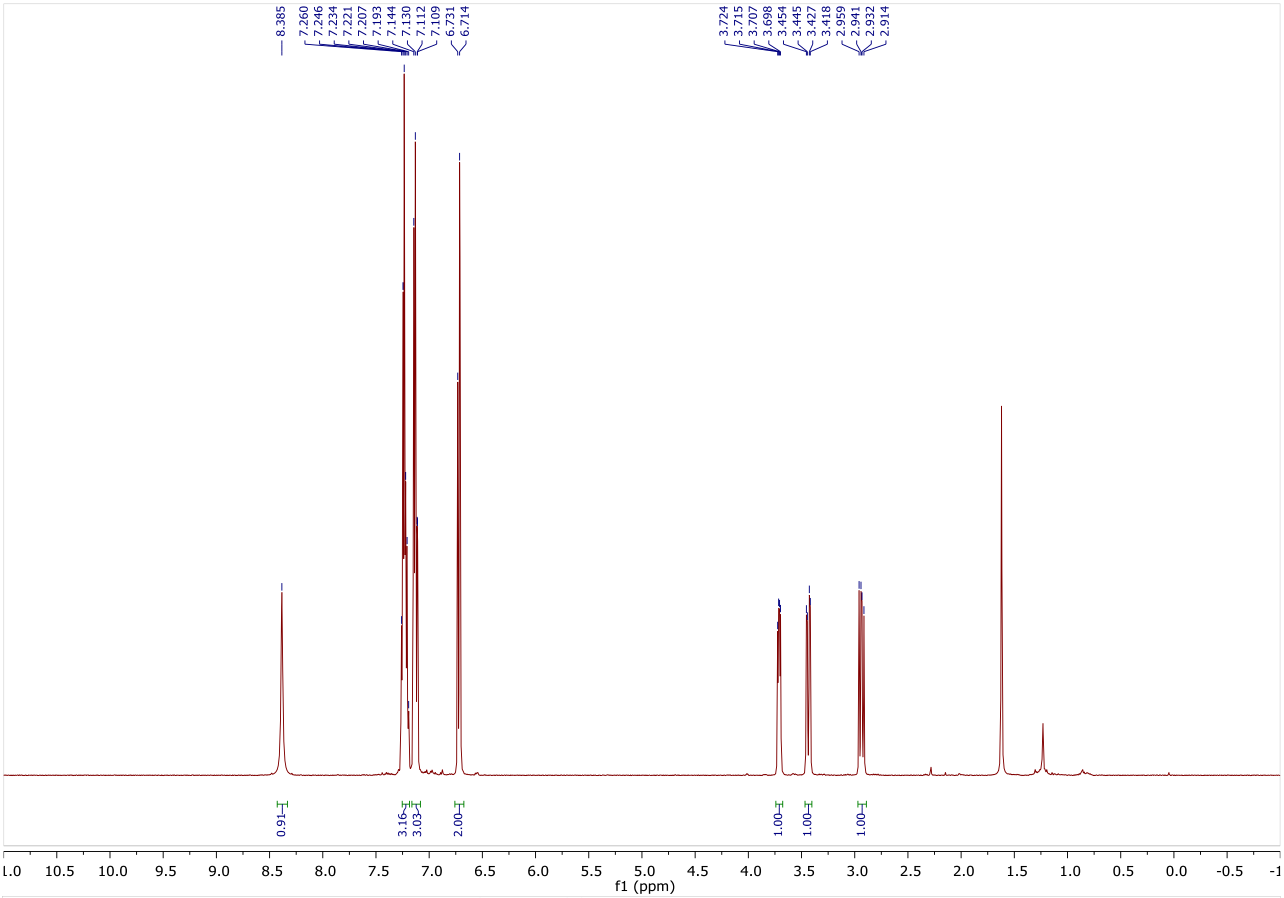
8.385
7.260
7.246
7.234
7.221
7.207
7.193
7.144
7.130
7.112
7.109
6.731
6.714
3.724
3.715
3.707
3.698
3.454
3.445
3.427
3.418
2.959
2.941
2.932
2.914
0.91
3.16
3.03
2.00
1.00
1.00
1.00
f1 (ppm)

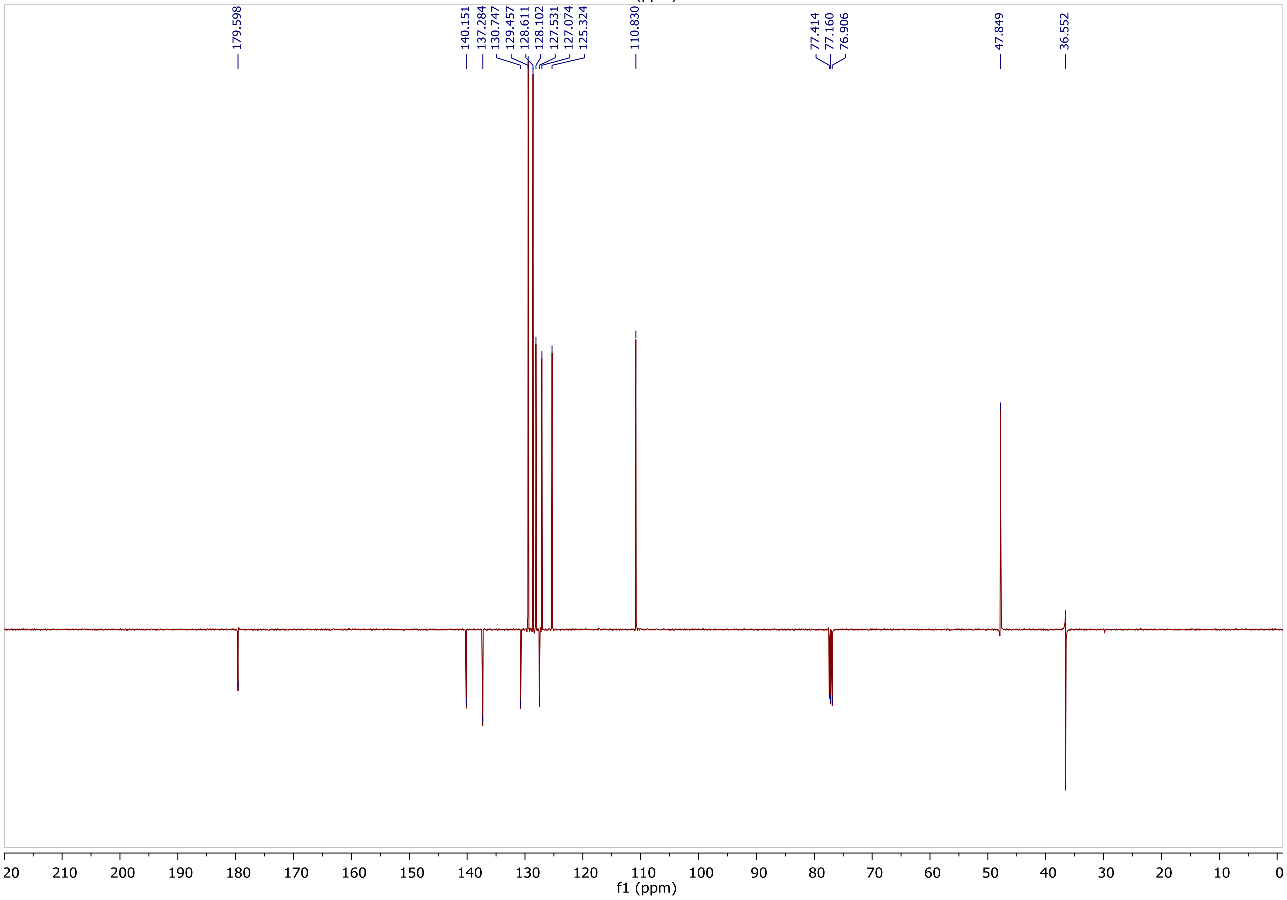
179.598
140.151
137.284
130.747
129.457
128.611
128.102
127.531
127.074
125.324
110.830
77.414
77.160
76.906
47.849
36.552
f1 (ppm)

3-benzyl-5-fluoroindolin-2-one

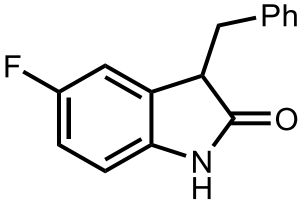

34

The title compound was prepared according to general procedure 1 using benzyl alcohol (124 µL, 130 mg, 1.2 mmol) and 5-fluoro-2-oxindole (151 mg, 1.0 mmol). Purification by flash silica chromatography (eluent = 20% EtOAc in hexanes, 30 x 150 mm silica) gave the title compound as a pale brown solid (120 mg, 50%). mp 137-139 °C; R_f = 0.26 (eluent = 20% EtOAc in hexanes); ν_{max}/cm^{-1} (film): 3080, 3026, 2918, 2872, 1715, 1672, 1628, 1479, 1252, 1206, 1159, 889, 866, 818, 777, 741, 692, 652; **^{1}H NMR (500 MHz, $CDCl_3$)** δ_H: 2.96 (1H, dd, *J* 14.0, 9.5), 3.50 (1H, dd, *J* 14.0, 4.5), 3.76 (1H, dd, *J* 9.0, 4.5), 6.50 (1H, d, *J* 7.5), 6.75 (1H, dd, *J* 8.5, 4.5), 6.88 (1H, dt, *J* 9.0, 2.0), 7.19 (2H, d, *J* 7.0*)*, 7.22-7.33 (3H, m), 8.03 (1H, br s); **^{19}F NMR (376 MHz, $CDCl_3$)** δ_F: 120.8; **^{13}C NMR (126 MHz, $CDCl_3$)** δ_C: 36.6, 48.1, 110.2 (d, *J* 8.2), 113.0 (d, *J* 25.0), 114.5 (d, *J* 23.7), 127.1, 128.6, 129.5, 130.7 (d, *J* 8.4), 137.4 (d, *J* 2.0), 137.4, 158.8 (d, *J* 240), 179.3; HRMS (**NSI$^+$**) calculated for $[C_{15}H_{13}FNO]^+$ $(M + H)^+$ m/z: 242.0976, found 242.0979 (+1.4 ppm).

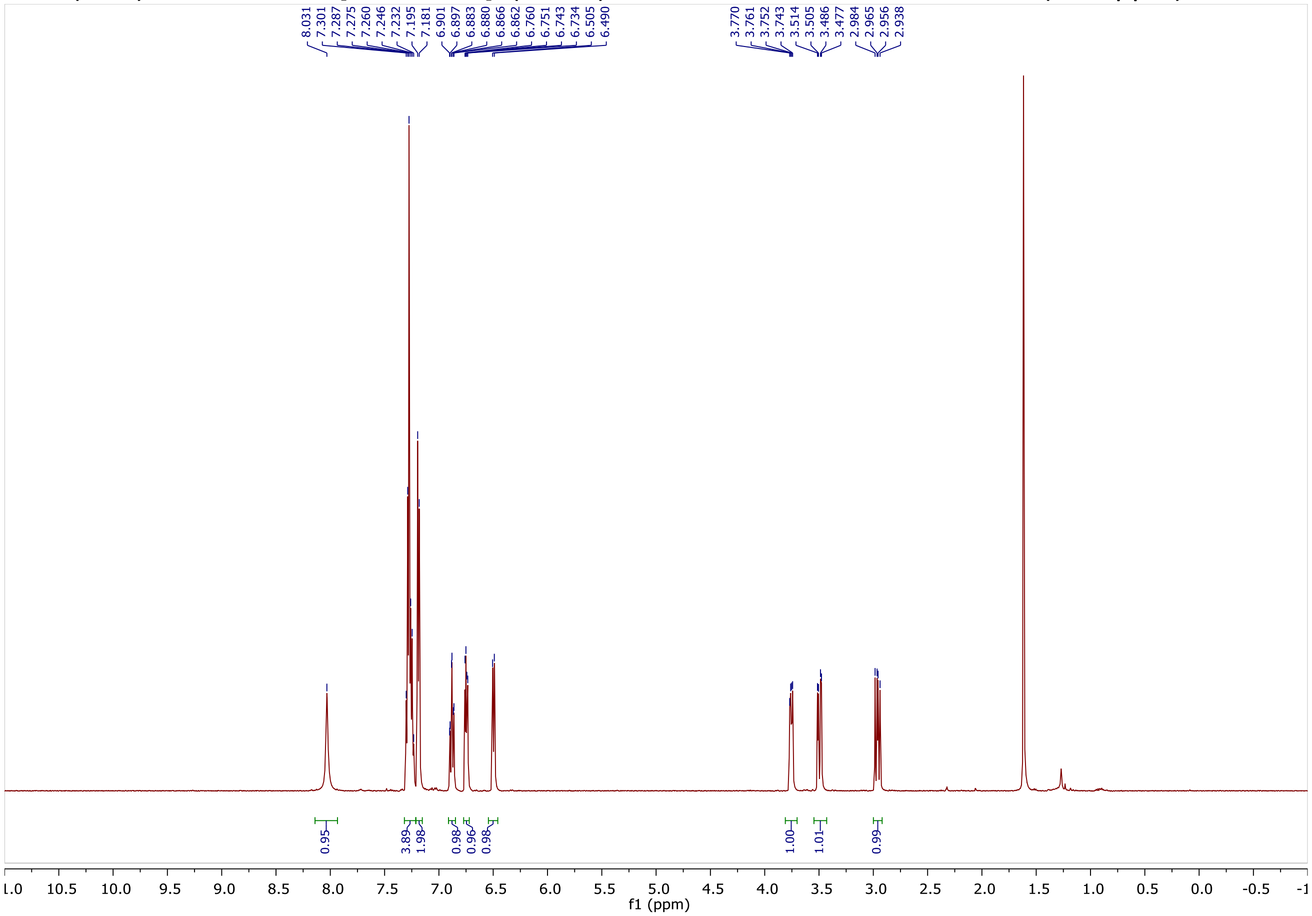

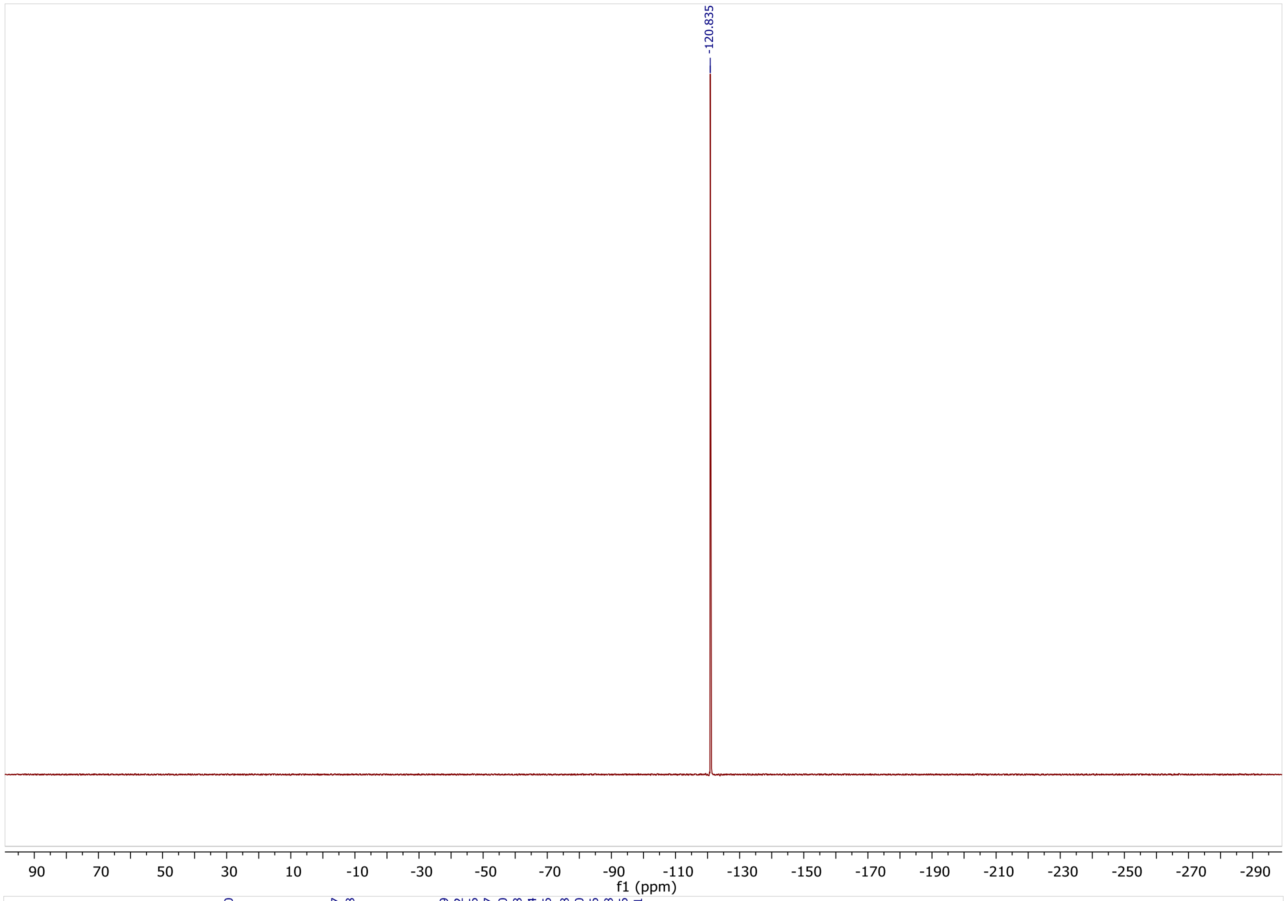

-120.835
90
70
50
30
10
-10
-30
-50
-70
-90
-110
-130
-150
-170
-190
-210
-230
-250
-270
-290
f1 (ppm)

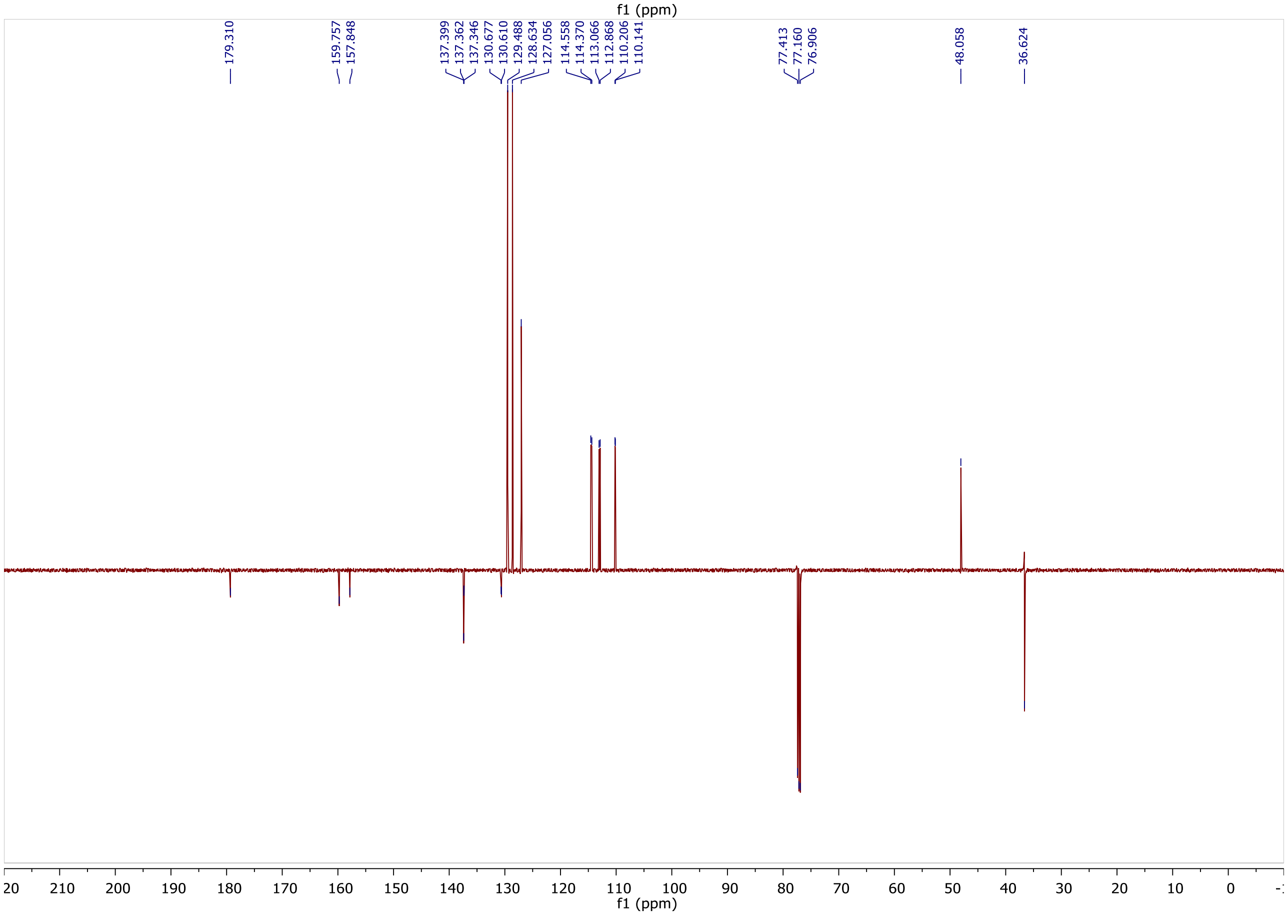

179.310
159.757
157.848
137.399
137.362
137.346
130.677
130.610
129.488
128.634
127.056
114.558
114.370
113.066
112.868
110.206
110.141
77.413
77.160
76.906
48.058
36.624
210
200
190
180
170
160
150
140
130
120
110
100
90
80
70
60
50
40
30
20
10
0
f1 (ppm)

3-benzyl-1-methylindolin-2-one

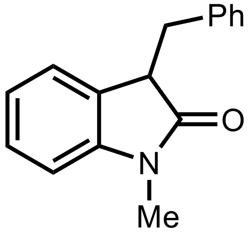

35

The title compound was prepared according to general procedure 1 using benzyl alcohol (124 µL, 130 mg, 1.2 mmol) and *N*-methyloxindole (147 mg, 1.0 mmol). Purification by flash silica chromatography (eluent = 15% EtOAc in hexanes, 30 x 150 mm silica) gave the title compound as a yellow solid (177 mg, 75%); mp 61-63 °C; R_f = 0.32 (eluent = 20% EtOAc in hexanes); ν_{max}/cm^{-1} (film): 2922, 2851, 1697, 1609, 1495, 1466, 1452, 1370, 1354, 1339, 1262, 1157, 1121, 1084, 1018, 991, 748, 702, 623, 586, 540; **^{1}H NMR (500 MHz, $CDCl_3$)** δ_H: 2.90 (1H, dd, *J* 14.0, 9.5), 3.18 (3H, s) 3.52 (1H, dd, *J* 13.5, 4.5), 3.74 (1H, dd, *J* 9.5, 4.5), 6.77 (2H, t, *J* 7.0), 6.94 (1H, t, *J* 6.5), 7.19 (2H, d, *J* 7.0), 7.21-7.32 (4H, m); **^{13}C NMR (126 MHz, $CDCl_3$)** δ_C: 26.3, 37.0, 47.2, 108.0, 122.2, 124.7, 126.7, 128.1, 128.4, 128.5, 129.5, 138.1, 144.3, 177.2; HRMS (**NSI$^+$**) calculated for $[C_{16}H_{16}NO]^+$ $(M + H)^+$ m/z: 238.1226, found 238.1228 (+0.7 ppm).

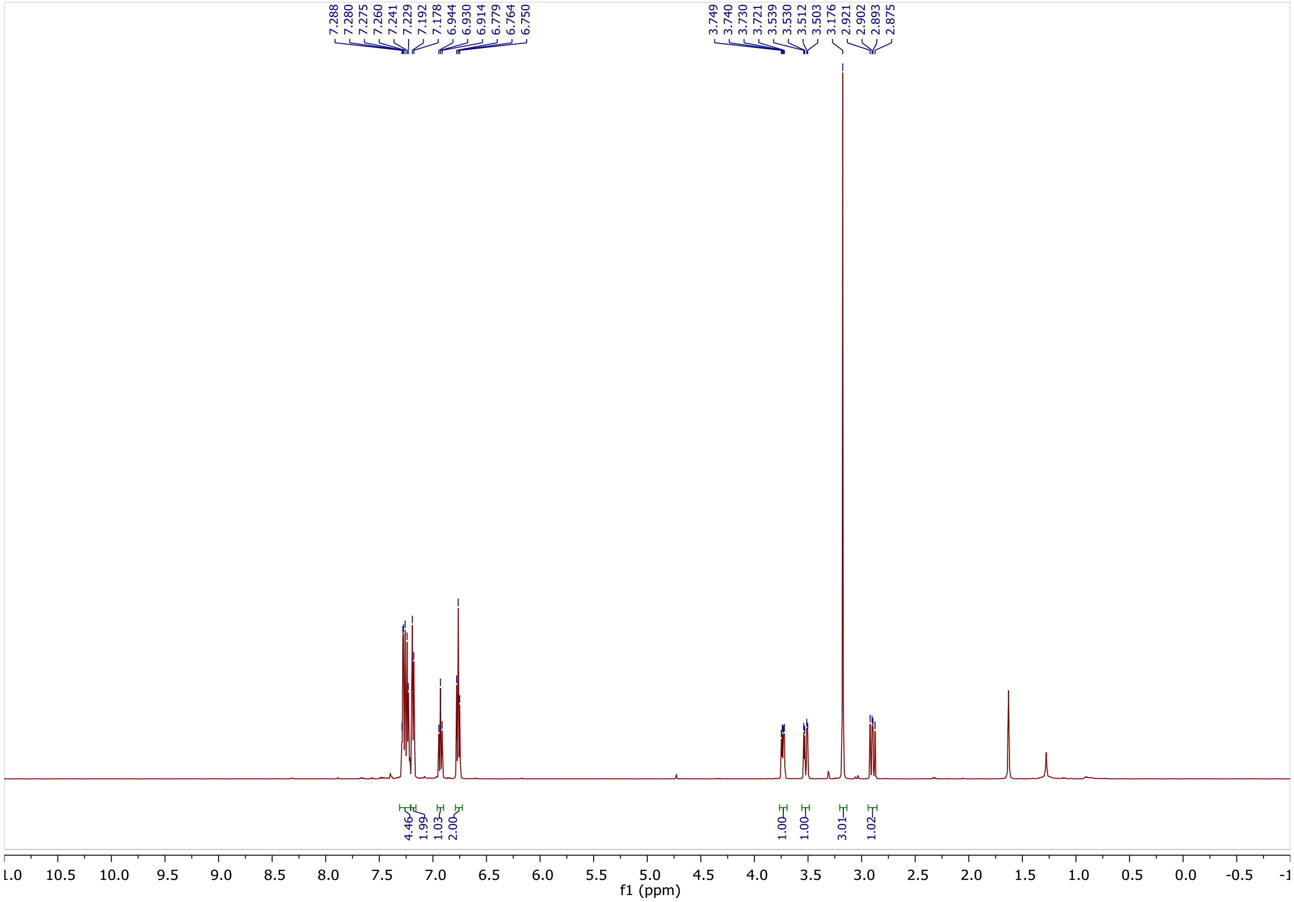

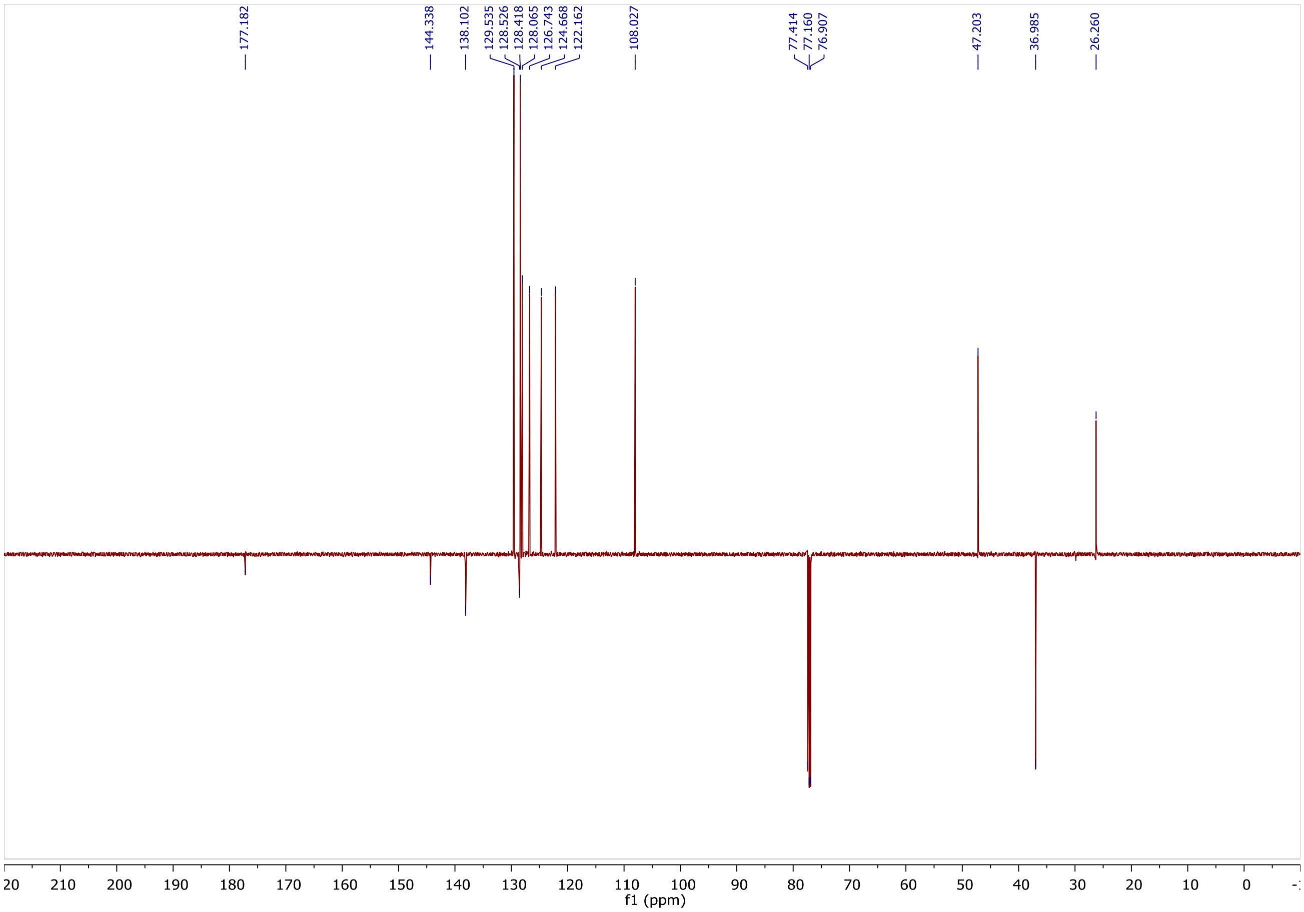

1,3-dibenzylindolin-2-one

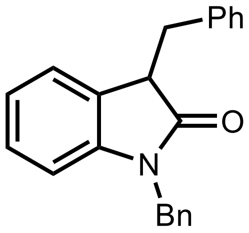

36

The title compound was prepared according to general procedure 1 using benzyl alcohol (124 µL, 130 mg, 1.2 mmol) and *N*-benzyloxindole (223 mg, 1.2 mmol). Purification by flash silica chromatography (eluent = 10% EtOAc in hexanes, 30 x 150 mm silica) gave the title compound as a white solid (253 mg, 80%); mp 95-97 °C (Lit. 97-98 °C);[12,] R_f = 0.36 (eluent = 20% EtOAc in hexanes); **^{1}H NMR (400 MHz, $CDCl_3$)** δ_H: 3.13 (1H, dd, *J* 13.6, 8.0), 3.51 (1H, dd, *J* 13.6, 4.4), 3.85 (1H, dd, *J* 8.4, 4.4), 4.58 (1H, d, *J* 15.6), 5.03 (1H, d, *J* 15.6), *6*.55 (1H, d, *J* 7.6), 6.94-6.99 (4H, m), 7.19-7.15 (3H, m), 7.17-7.25 (6H, m); **^{13}C NMR (101 MHz, $CDCl_3$)** δ_C: 36.6, 43.6, 47.2, 109.2, 122.2, 124.6, 126.8, 127.0, 127.4, 128.0, 128.3, 128.4, 128.8, 129.8, 135.7, 137.5, 143.5, 177.0. Spectroscopic data in accordance with the literature.[12]

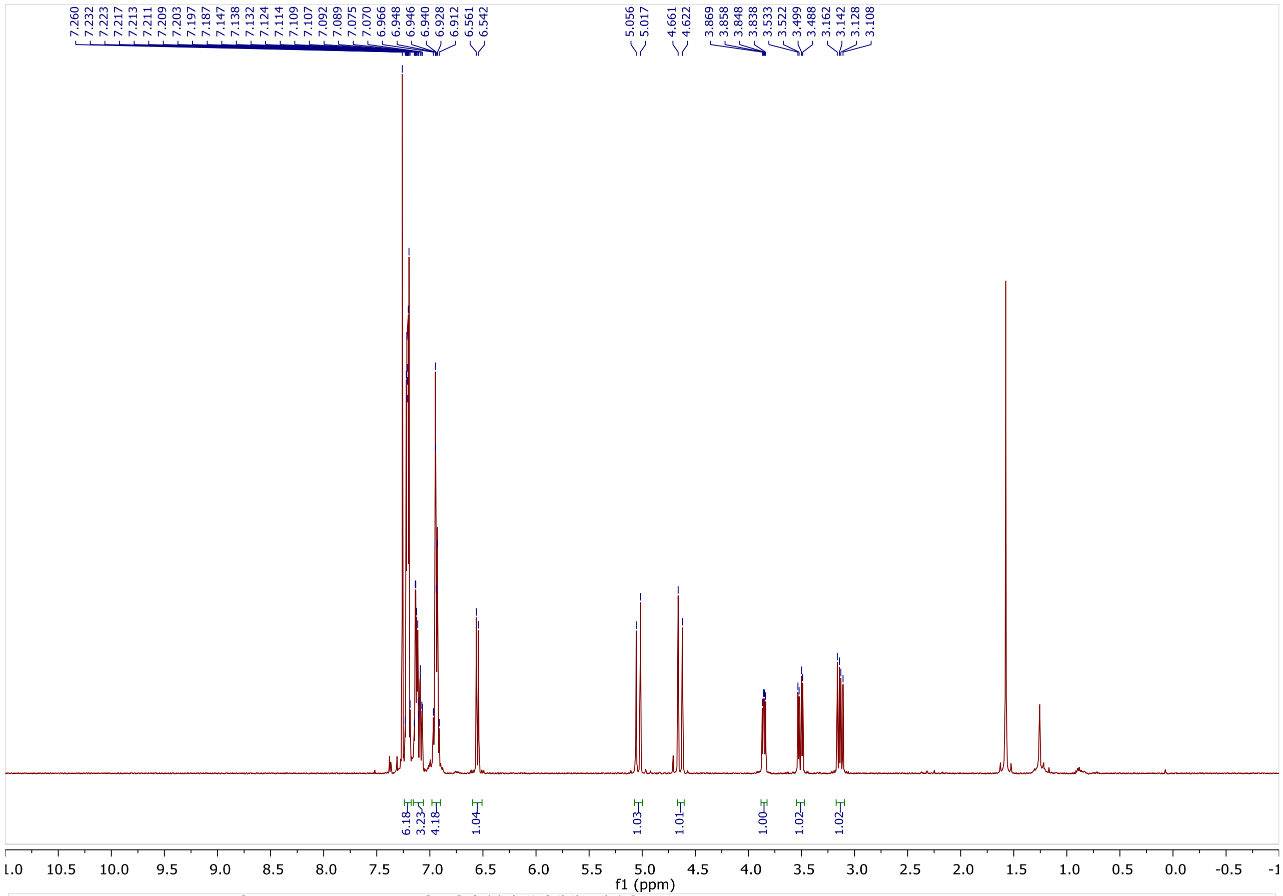

7.260
7.232
7.223
7.217
7.213
7.211
7.209
7.203
7.197
7.187
7.147
7.138
7.132
7.124
7.114
7.109
7.107
7.092
7.089
7.075
7.070
6.966
6.948
6.946
6.940
6.928
6.912
6.561
6.542
5.056
5.017
4.661
4.622
3.869
3.858
3.848
3.838
3.533
3.522
3.499
3.488
3.162
3.142
3.128
3.108
6.18
3.23
4.18
1.04
1.03
1.01
1.00
1.02
1.02
f1 (ppm)

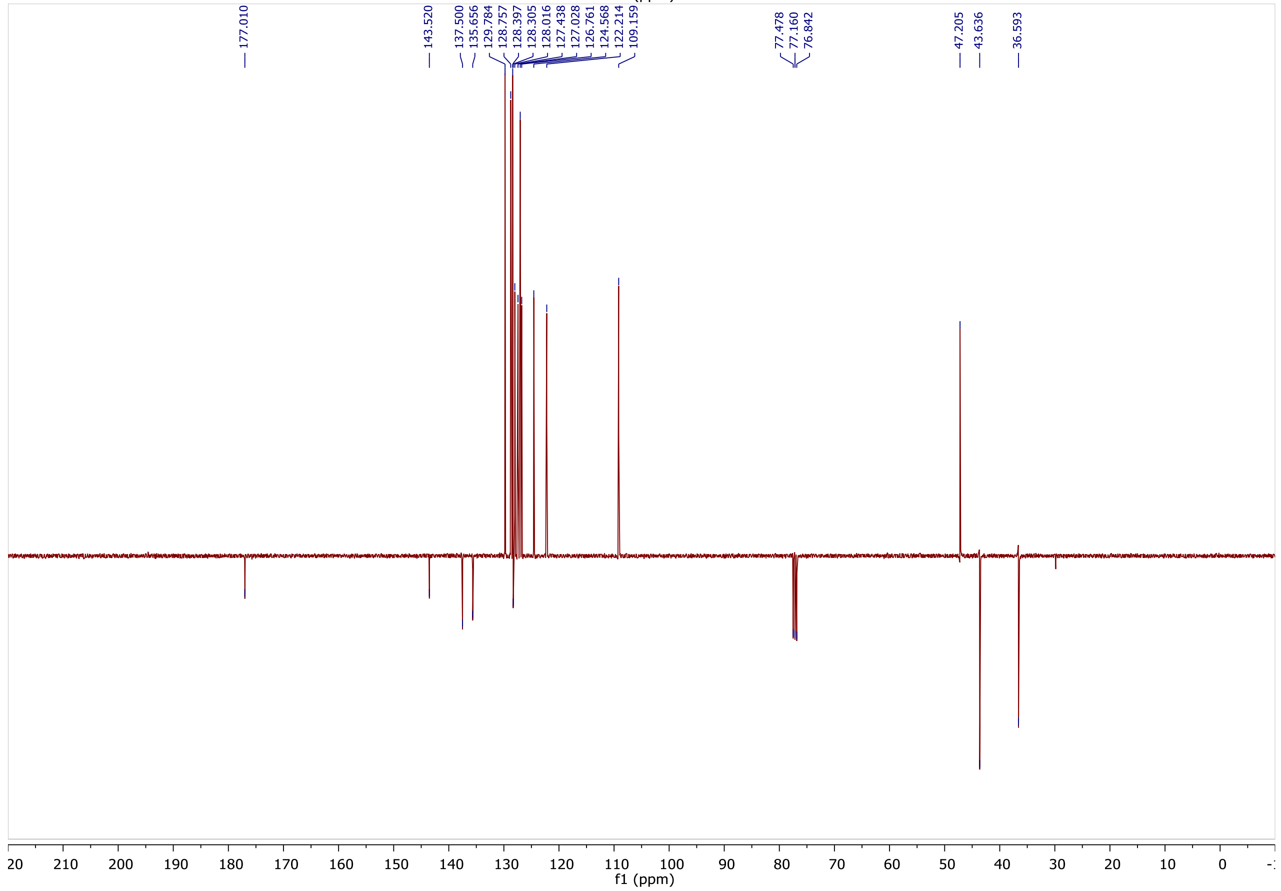

177.010
143.520
137.500
135.656
129.784
128.757
128.397
128.305
128.016
127.438
127.028
126.761
124.568
122.214
109.159
77.478
77.160
76.842
47.205
43.636
36.593
f1 (ppm)

3-benzyl-1-phenylindolin-2-one

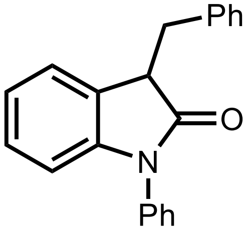

37

The title compound was prepared according to general procedure 1 using benzyl alcohol (124 µL, 130 mg, 1.2 mmol) and 1-phenyloxindole (209 mg, 1.2 mmol). Purification by flash silica chromatography (eluent = 15% EtOAc in hexanes, 30 x 150 mm silica) gave the title compound as an off-white solid (223 mg, 75%); mp 87-89 °C; R_f = 0.26 (eluent = 20% EtOAc in hexanes); ν_{max}/cm^{-1} (film): 3057, 3034, 1717, 1609, 1589, 1497, 1476, 1451, 1371, 1327, 1275, 1223, 1171, 1099, 1076, 1022, 754, 696; **^{1}H NMR (400 MHz, $CDCl_3$)** δ_H: 3.17 (1H, dd, *J* 14.0, 8.4), 3.52 (1H, dd, *J* 13.2, 4.4), 3.92 (1H, dd, *J* 8.4, 4.4), 6.64 (1H, d, *J* 7.6), 6.93-7.02 (2H, m), 7.11-7.17 (3H, m), 7.17-7.25 (5H, m), 7.38 (1H, t, *J* 6.8), 7.48 (2H, t, *J* 7.2); **^{13}C NMR (101 MHz, $CDCl_3$)** δ_C: 37.2, 47.3, 109.2, 122.6, 124.8, 126.7, 126.8, 128.0, 128.1, 128.2, 128.3, 129.6, 129.7, 134.5, 137.3, 144.4, 176.5; HRMS (**NSI$^+$**) calculated for $[C_{21}H_{18}NO]^+(M + H)^+$ m/z: 300.1383, found 300.1384 (+0.4 ppm).

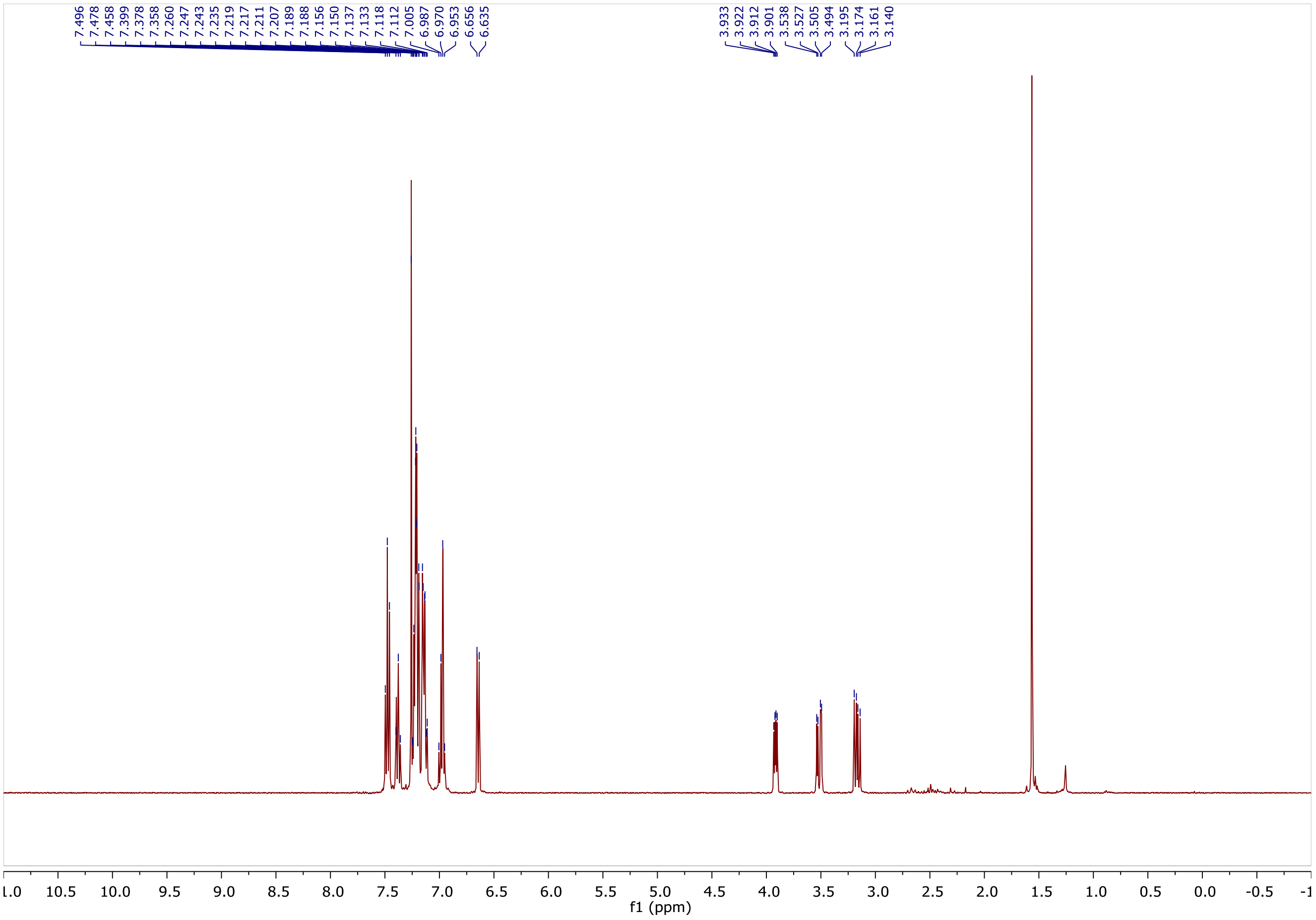

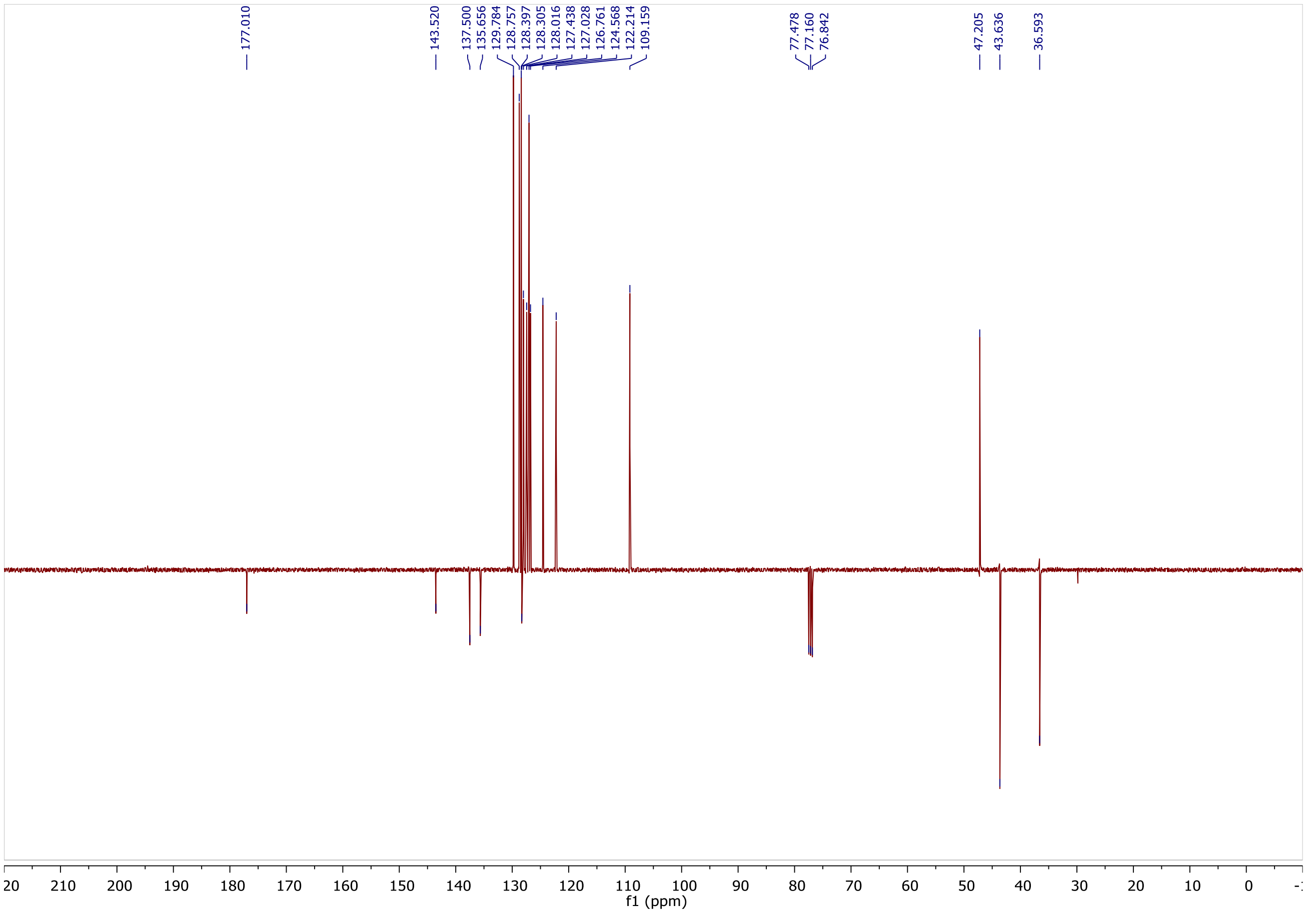

5-benzyl-1,3-dimethylpyrimidine-2,4,6(1H,3H,5H)-trione

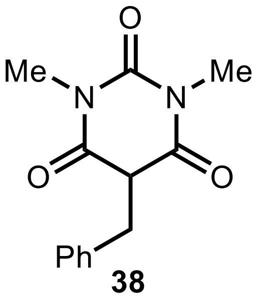

38

The title compound was prepared according to general procedure 3 using *N*,*N*-dimethyl barbituric acid (156 mg, 1.0 mmol) and benzyl alcohol (124 µL, 130 mg, 1.2 mmol), in the **absence of base**. Purification by flash silica chromatography (eluent = 20% EtOAc in hexanes, 30 x 150 mm silica) gave the title compound as an orange solid (147 mg, 60%). mp 110-112 °C; R_f = 0.23 (eluent = 20% EtOAc in hexanes); ν_{max}/cm^{-1} (film): 2928, 2851, 1665, 1441, 1418, 1377, 1310, 1279, 1202, 1161, 1101, 1024, 995, 930, 849, 750, 706, 561; **^{1}H NMR (400 MHz, $CDCl_3$)** δ_H: 3.12 (6H, s), 3.46 (2H, d, *J* 4.8), 3.77 (1H, t, *J* 4.8), 7.03 (2H, dd, *J* 7.2, 3.6), 7.21-7.25 (3H, m); **^{13}C NMR (101 MHz, $CDCl_3$)** δ_C: 28.3, 38.0, 50.9, 128.0, 128.7, 129.0, 135.3, 151.1, 168.4; HRMS (**NSI$^+$**) calculated for $[C_{13}H_{15}N_2O_3]^+(M + H)^+$ m/z: 247.1077, found 247.1080 (+1.1 ppm).

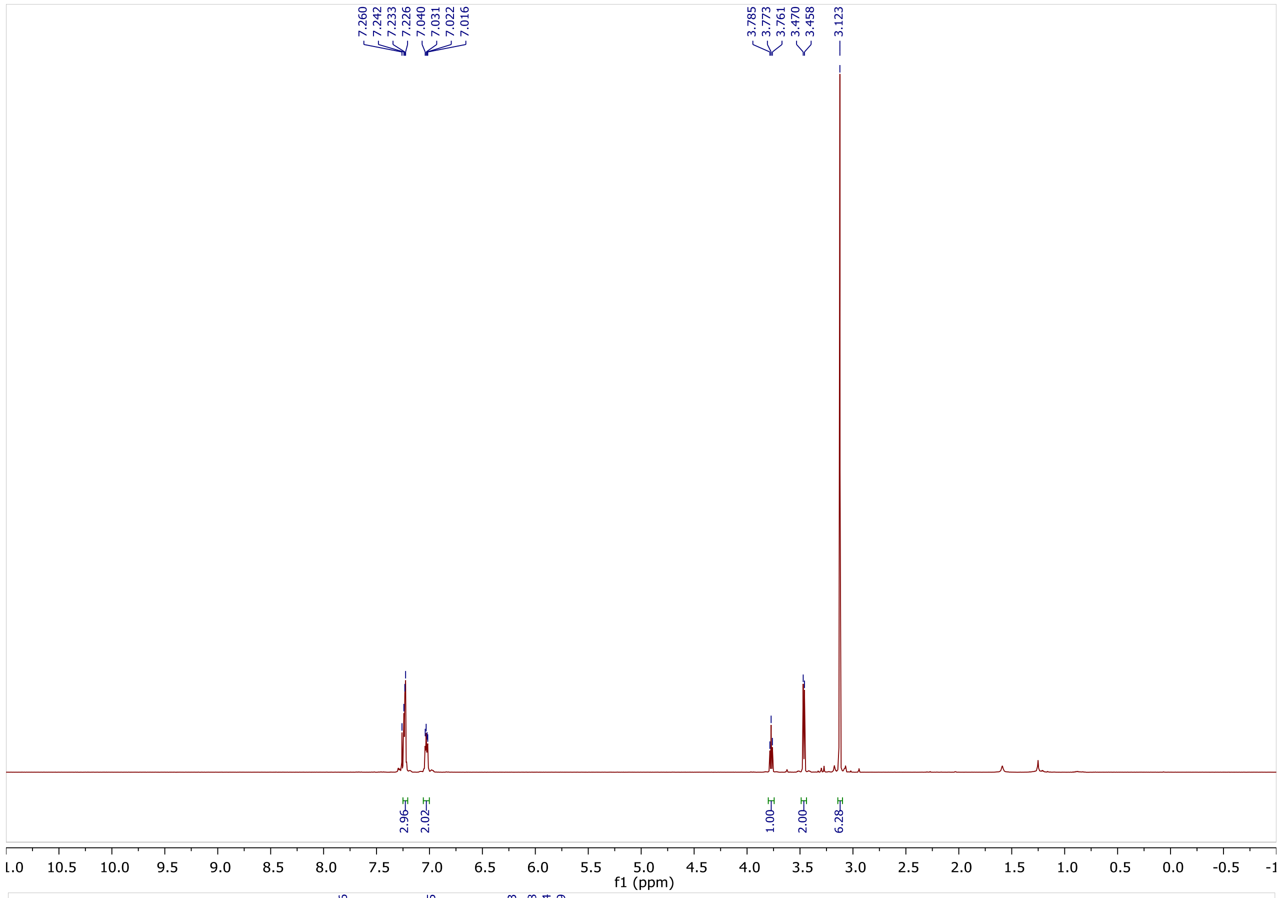
7.260
7.242
7.233
7.226
7.040
7.031
7.022
7.016
3.785
3.773
3.761
3.470
3.458
3.123
2.96
2.02
1.00
2.00
6.28
f1 (ppm)

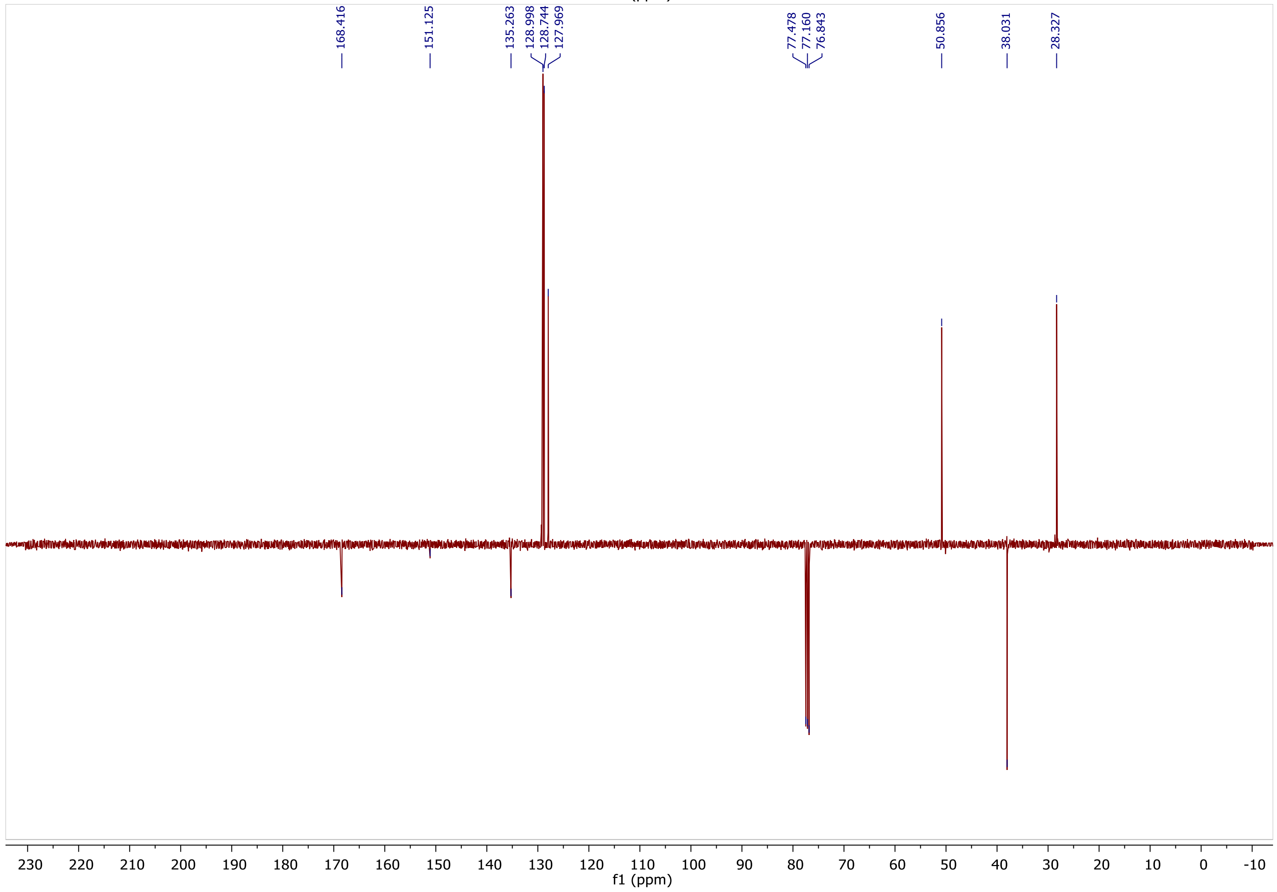
168.416
151.125
135.263
128.998
128.744
127.969
77.478
77.160
76.843
50.856
38.031
28.327
f1 (ppm)

5-benzyl-1,3-dicyclohexylpyrimidine-2,4,6(1H,3H,5H)-trione

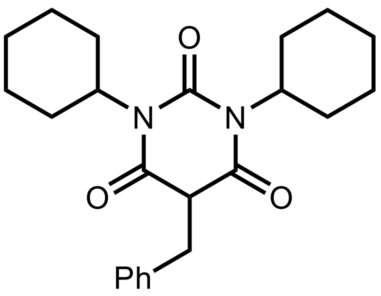

39

The title compound was prepared according to general procedure 3 using *N*,*N*-dicyclohexyl barbituric acid (292 mg, 1.0 mmol) and benzyl alcohol (124 µL, 130 mg, 1.2 mmol) with 0 equiv of base. Purification by flash silica chromatography (eluent = 5% EtOAc in hexanes, 30 x 150 mm silica) gave the title compound as a off-white solid (285 mg, 75%). mp 115-118 °C; R_f = 0.52 (eluent = 10% EtOAc in hexanes); ν_{max}/cm^{-1} (film): 2930, 2859, 2359, 1690, 1672, 1456, 1439, 1418, 1379, 1360, 1333, 1314, 1267, 1216, 1177, 897, 760, 741, 702, 575, 527, 488, 461; **^{1}H NMR (400 MHz, $CDCl_3$)** δ_H: 1.07-1.50 (10H, m), 1.56-1.67 (2H, m), 1.72-1.88 (4H, m), 2.02-2.23 (4H, m), 3.44 (2H, d, *J* 4.8), 3.65 (1H, t, *J* 5.2), 4.47 (2H, tt, *J* 12.0, 4.0), 7.02-7.10 (2H, m), 7.19-7.25 (3H, m); **^{13}C NMR (101 MHz, $CDCl_3$)** δ_C: 25.3, 26.4, 26.5, 28.9, 29.2, 51.0, 55.4, 127.7, 128.7, 129.5, 135.3, 150.5, 168.7. HRMS (**ES$^+$**) calculated for $[C_{23}H_{31}N_2O_3]^+$ (M)$^+$ m/z : 383.2335, found 383.2329 (-1.6 ppm).

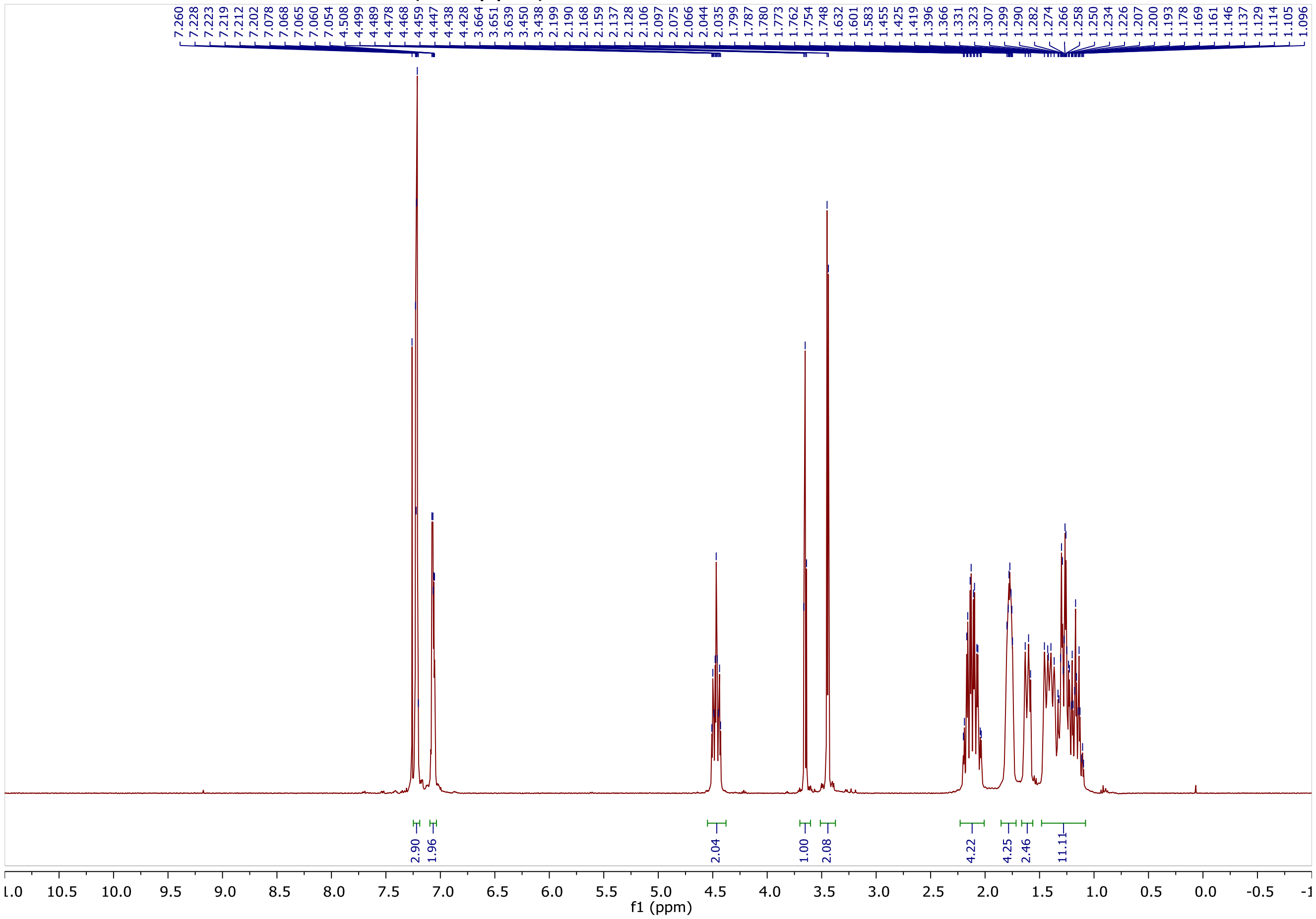

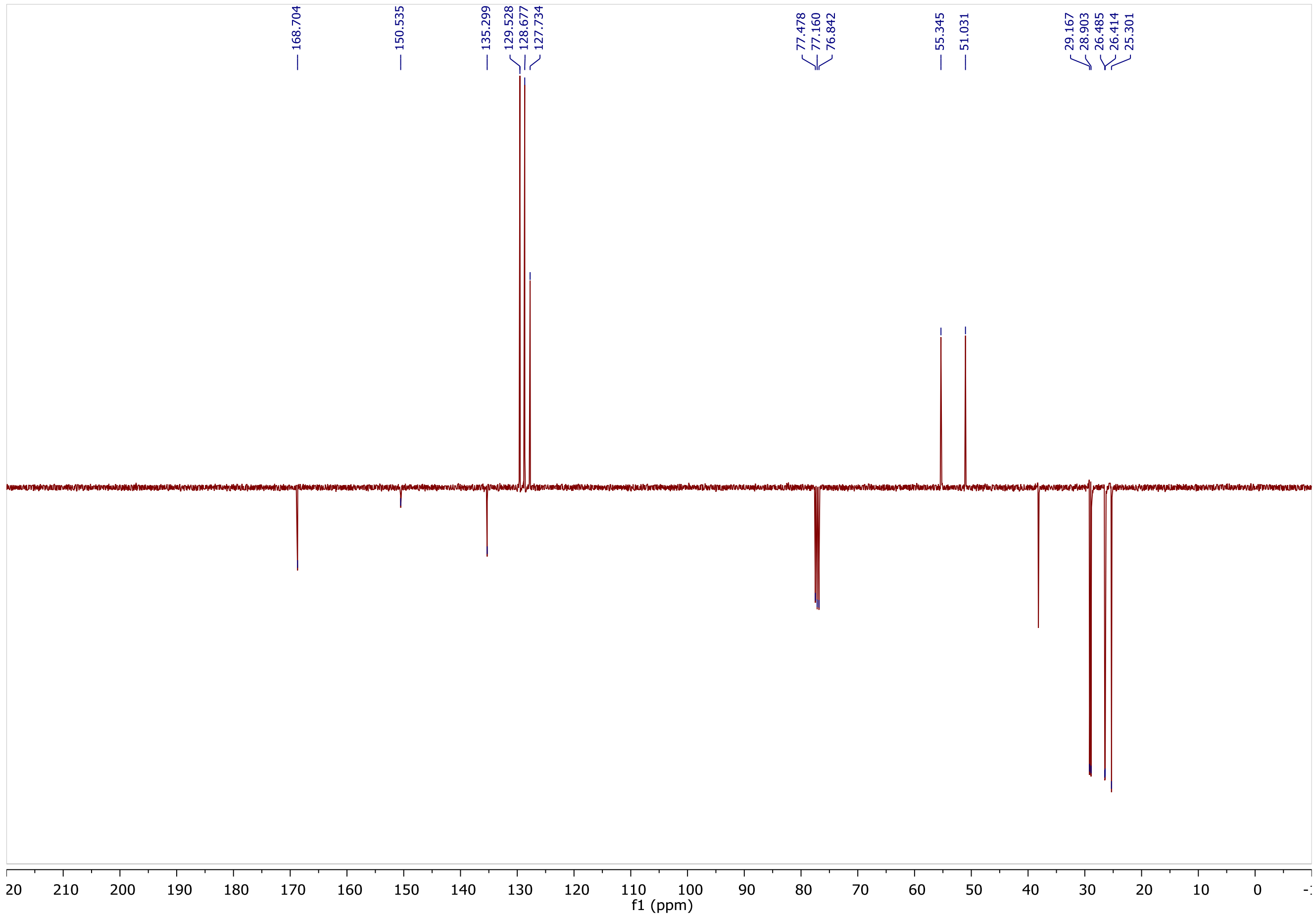

1,3,5-tribenzylpyrimidine-2,4,6(1H,3H,5H)-trione

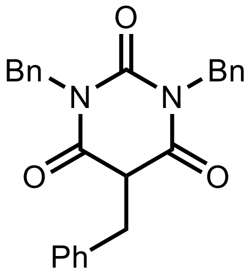

40

The title compound was prepared according to general procedure 3 using 1,3-dibenzylpyrimidine-2,4,6(1H,3H,5H)-trione (308 mg, 1.0 mmol) and benzyl alcohol (124 µL, 130 mg, 1.2 mmol) and K_2CO_3 (69 mg, 0.5 mmol). Purification by flash silica chromatography (eluent = 10% EtOAc in hexanes, 30 x 180 mm silica) gave the title compound as a white solid (227 mg, 57%); mp 105-108 °C (Lit. 110-112 °C),[15] R_f = 0.48 (eluent = 30% EtOAc in hexanes); ν_{max}/cm^{-1} (film): 3061, 3032, 2978, 1678, 1584, 1493, 1433, 1400, 1337, 1273, 1204, 1155, 1084, 1063, 1028, 745, 691, 604, 573, 500; **^{1}H NMR (500 MHz, $CDCl_3$)** δ_H: 3.47 (2H, d, *J* 5.0), 3.79 (1H, t, *J* 5.0), 4.90 (4H, s), 6.82 (2H, d, *J* 7.5), 6.95 (2H, t, *J* 7.5), 7.12 (1H, t, *J* 7.5), 7.18-7.32 (10H, m); **^{13}C NMR (126 MHz, $CDCl_3$)** δ_C: 37.2, 45.2, 50.5, 127.5, 128.0, 128.6, 128.7, 129.0, 129.3, 135.0, 136.0, 151.0, 168.0; HRMS (**ES$^+$**) calculated for $[C_{25}H_{23}N_2O_3]^+$ (M+H)$^+$ m/z : 399.1709, found 399.1707 (-0.5 ppm).

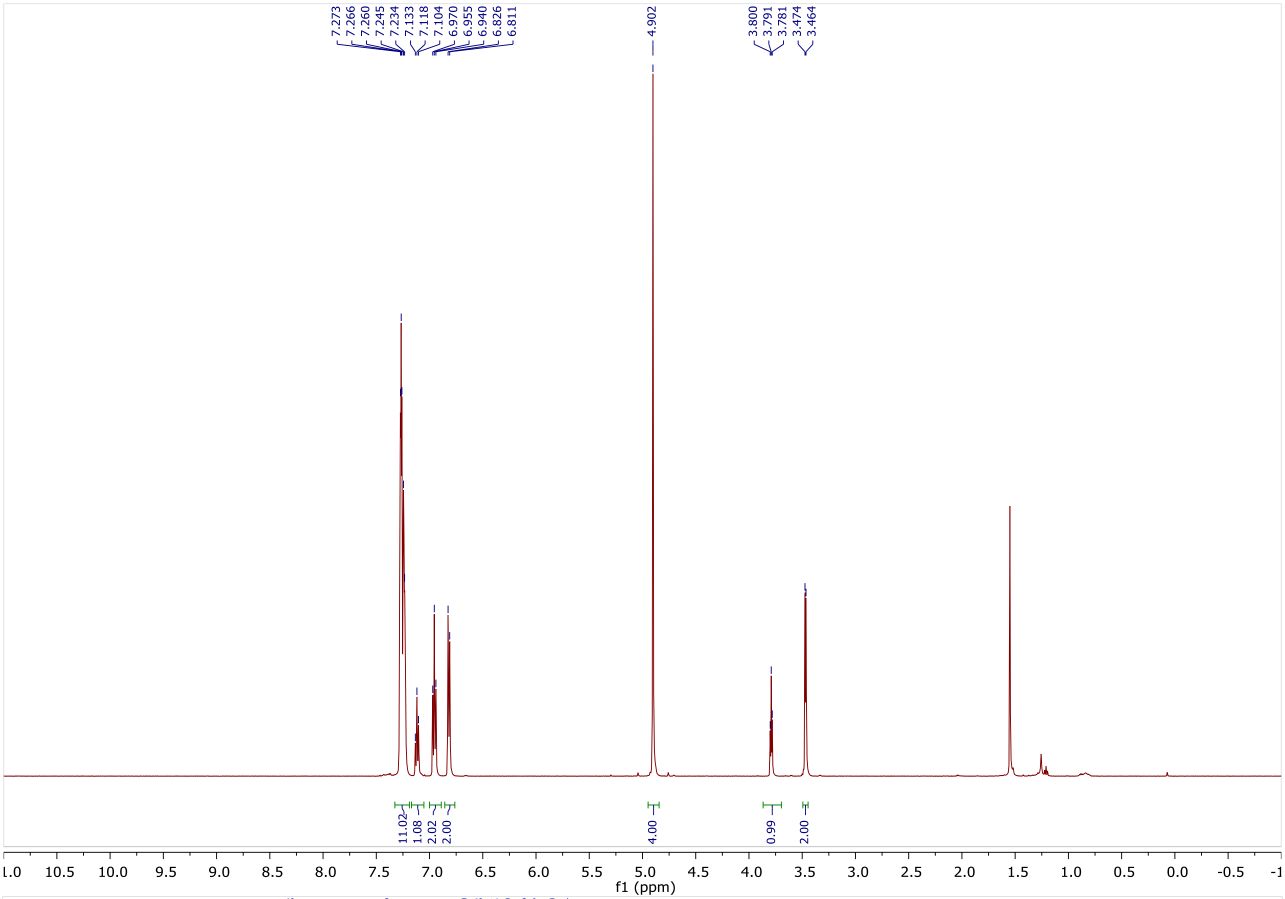
7.273
7.266
7.260
7.245
7.234
7.133
7.118
7.104
6.970
6.955
6.940
6.826
6.811
4.902
3.800
3.791
3.781
3.474
3.464
11.02
1.08
2.02
2.00
4.00
0.99
2.00
f1 (ppm)

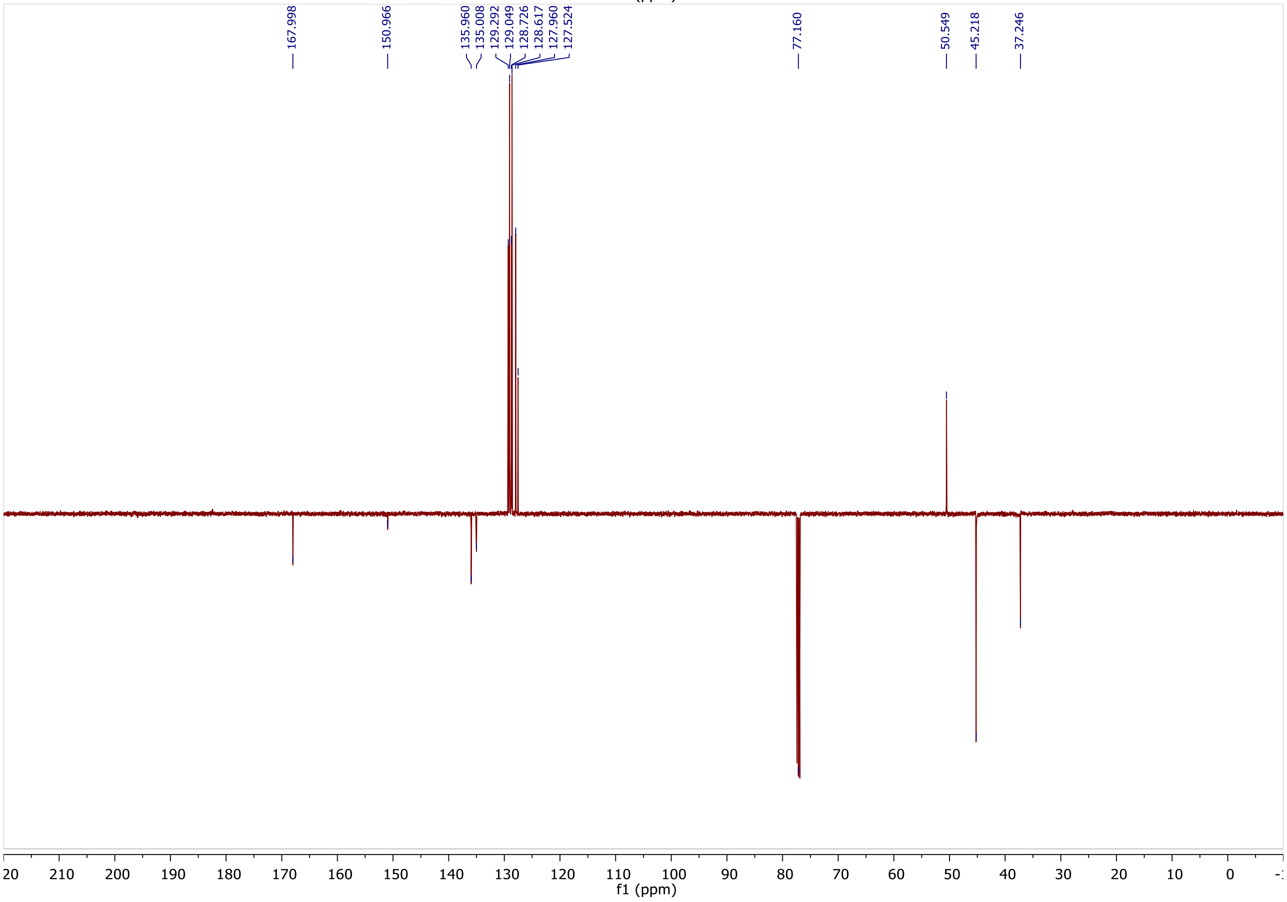
167.998
150.966
135.960
135.008
129.292
129.049
128.726
128.617
127.960
127.524
77.160
50.549
45.218
37.246
f1 (ppm)

5-(4-methoxybenzyl)-1,3-dimethylpyrimidine-2,4,6(1H,3H,5H)-trione

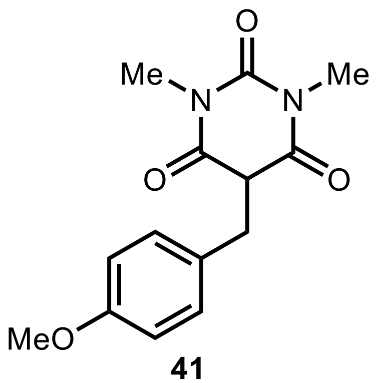

41

The title compound was prepared according to general procedure 3 using *N*,*N*-dimethyl barbituric acid (156 mg, 1.0 mmol), 4-methoxybenzyl alcohol (138 mg, 1.2 mmol), and K_2CO_3 (69 mg, 0.5 mmol). Purification by flash silica chromatography (eluent = 20% EtOAc in hexanes, 30 x 150 mm silica) gave the title compound as an off-white solid (138 mg, 50%); mp 108-111 °C (Lit. 113 °C),[16] R_f = 0.16 (eluent = 30% EtOAc in hexanes); **^{1}H NMR (500 MHz, $CDCl_3$)** δ_H: 3.14 (6H, s), 3.42 (2H, d, *J* 4.5), 3.74 (1H, t, *J* 4.5), 3.76 (3H, s), 6.75 (2H, d, *J* 7.5), 6.95 (2H, d, *J* 8.0); **^{13}C NMR (126 MHz, $CDCl_3$)** δ_C: 28.4, 37.3, 51.0, 55.3, 114.1, 127.1, 130.1, 151.2, 159.3, 168.5. Spectroscopic data in accordance with the literature.[16]

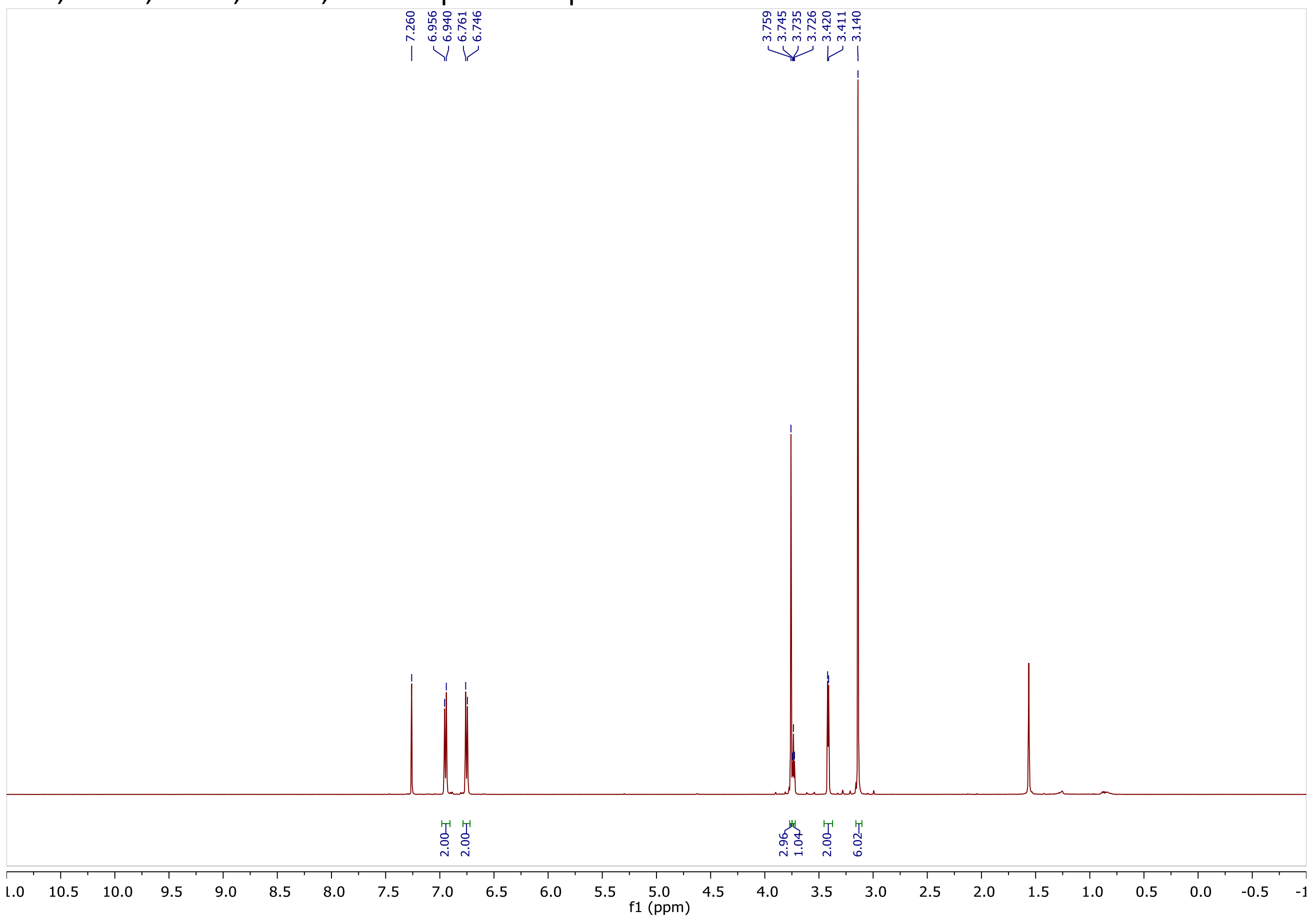

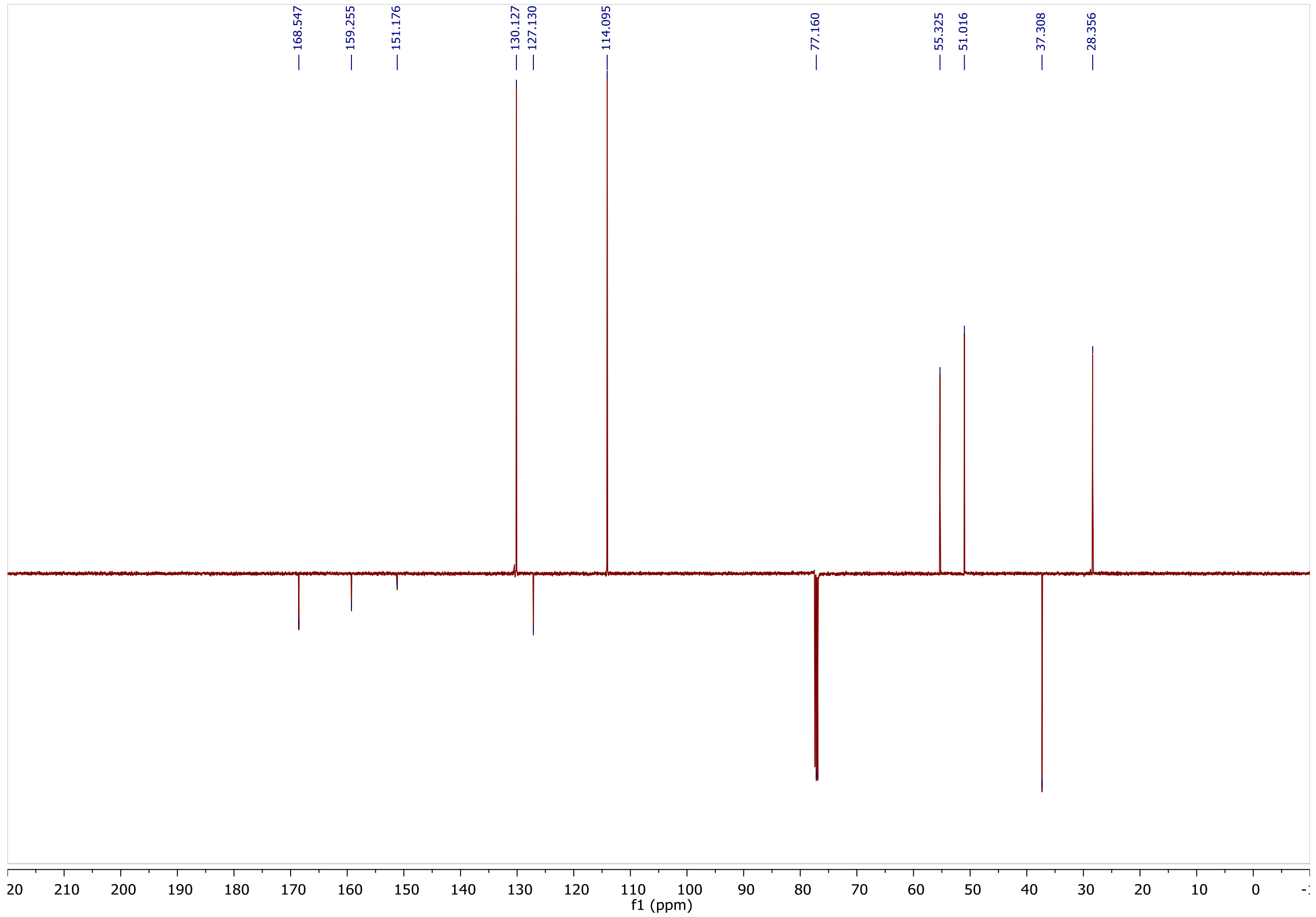

1,3-dimethyl-5-(4-methylbenzyl)pyrimidine-2,4,6(1H,3H,5H)-trione

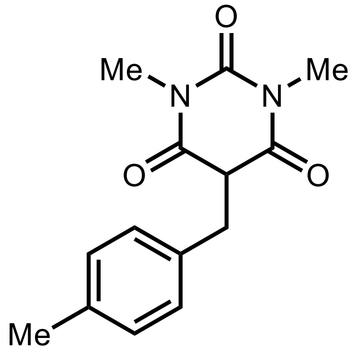

42

The title compound was prepared according to general procedure 3 using *N*,*N*-dimethyl barbituric acid (156 mg, 1.0 mmol) and 4-methylbenzyl alcohol (147 mg, 1.2 mmol). Purification by flash silica chromatography (eluent = 5-15% EtOAc in cyclohexane, 30 x 160 mm silica) gave the title compound as a yellow (189 mg, 73%); mp 94-97 °C; R_f = 0.24 (eluent = 30% EtOAc in hexanes); ν_{max}/cm^{-1} (film): 3036, 2986, 2936, 1680, 1653, 1468, 1435, 1383, 1323, 1308, 1287, 1119, 1003, 866, 804, 764, 604, 548, 474; **^{1}H NMR (500 MHz, CDCl$_3$)** δ_H: 2.28 (3H, s), 3.13 (6H, s), 3.42 (2H, d, *J* 4.5), 3.75 (1H, t, *J* 4.5), 6.91 (2H, d, *J* 7.5), 7.03 (2H, d, *J* 7.5); **^{13}C NMR (500 MHz, CDCl$_3$)** δ_C: 21.2, 28.3, 37.7, 50.9, 128.9, 129.4, 132.1, 137.6, 151.2, 168.5. HRMS (**EI$^+$**) calculated for $[C_{14}H_{16}N_2O_3]^+$ (M)$^+$ m/z : 260.1161, found 260.1170 (+3.5 ppm).

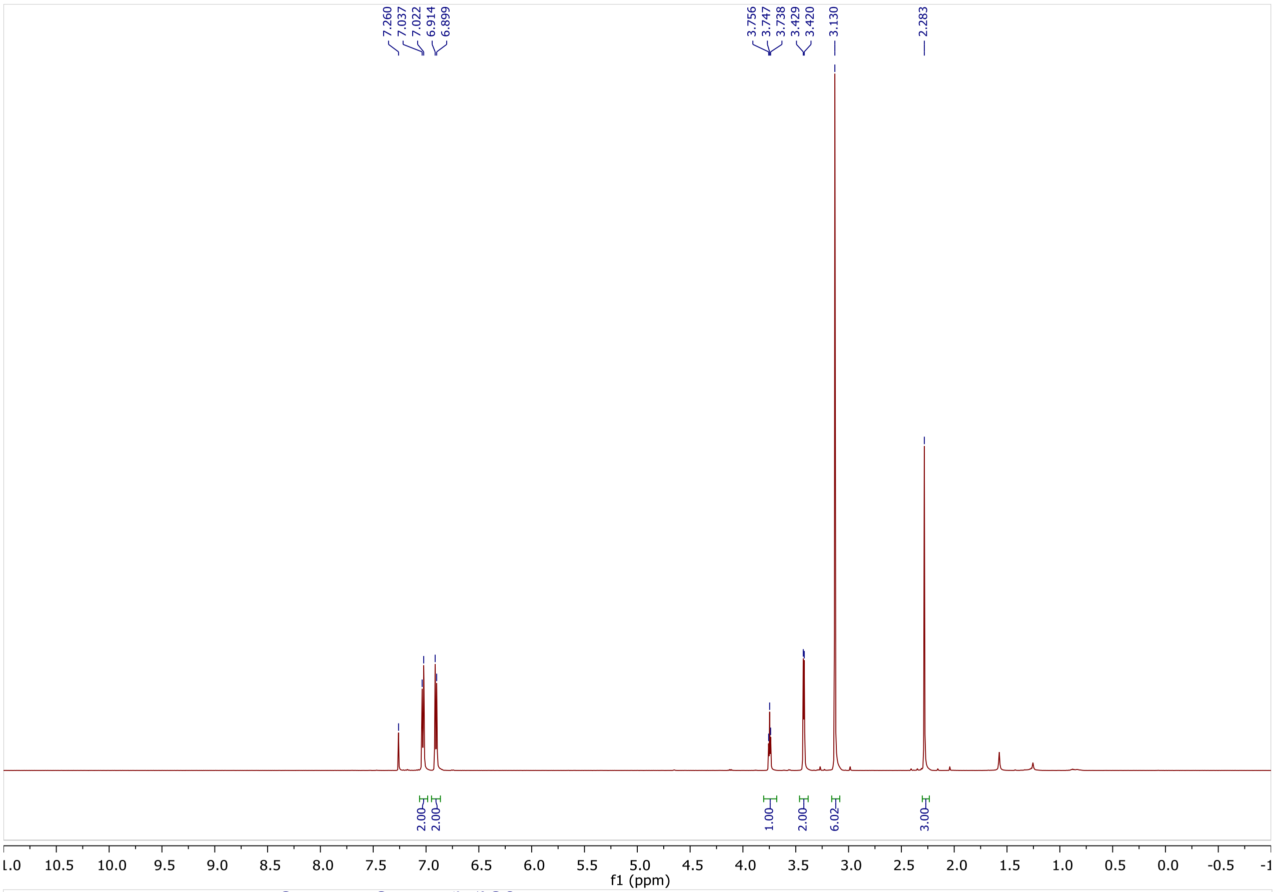
7.260
7.037
7.022
6.914
6.899
3.756
3.747
3.738
3.429
3.420
3.130
2.283
2.00
2.00
1.00
2.00
6.02
3.00
f1 (ppm)

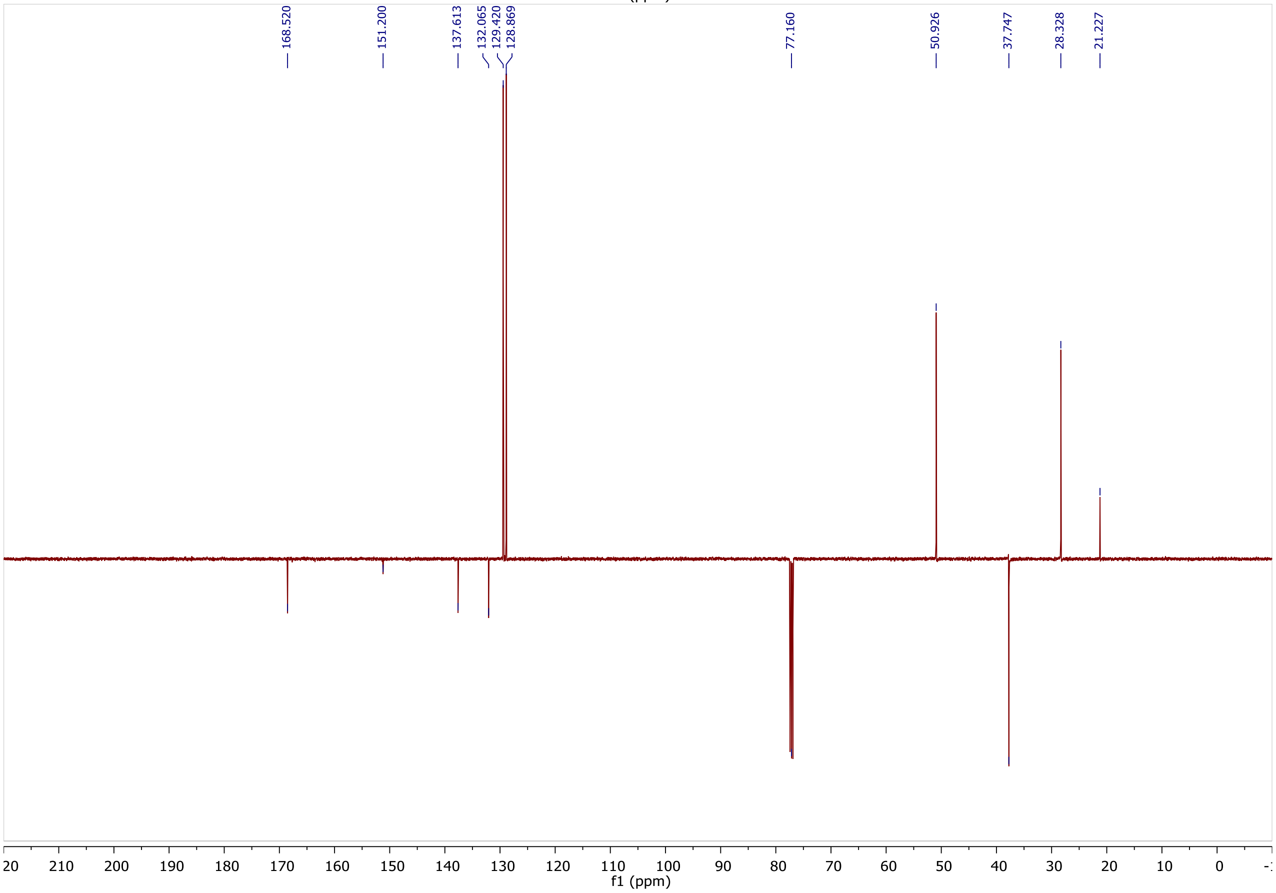
168.520
151.200
137.613
132.065
129.420
128.869
77.160
50.926
37.747
28.328
21.227
f1 (ppm)

2.5. Evidence supporting an α,β-unsaturated amide intermediate

2.5.1. Synthesis of 3-benzylideneindolin-2-one

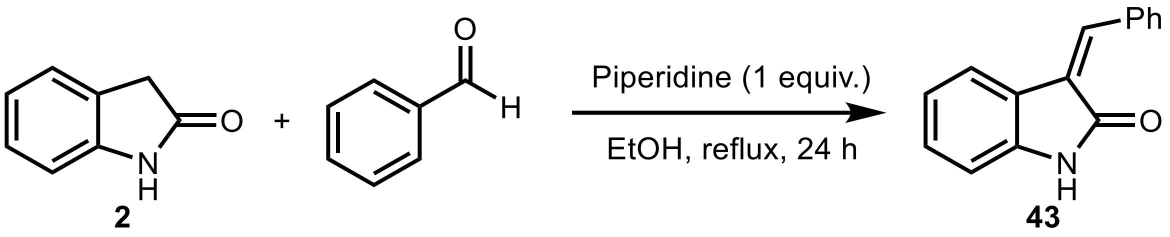

A 50 mL round-bottomed flask was charged with oxindole (666 mg, 5.0 mmol), EtOH (20 mL), piperidine (494 µL, 426 mg, 5.0 mmol) and benzaldehyde (610 µL, 637 mg, 6.0 mmol). The mixture was heated to reflux for 24 h. It was then cooled and concentrated *in vacuo*. Purification by flash silica chromatography (eluent = 10-25% EtOAc in hexanes, 40 x 160 mm silica) gave the title compound as a yellow solid (758 mg, 68%). mp 158-160 °C (Lit. 164-166 °C);[17] R_f = 0.30 (eluent = 30% EtOAc in hexanes); ν_{max} / cm^{-1} (film): 3186, 3150, 3078, 3021, 2832, 2357, 1705, 1607, 1460, 1327, 1231, 1202, 781, 689, 650, 550; **^{1}H NMR (500 MHz, $CDCl_3$)** δ_H: 6.87 (1H, t, *J* 7.5), 6.94 (1H, d, *J* 8.0), 7.40-7.52 (3H, m), 7.61-7.71 (3H, m), 7.86 (1H, s), 9.03 (1H, br s); **^{13}C NMR (500 MHz, $CDCl_3$)** δ_C: 110.3, 121.8, 122.0, 123.2, 127.6, 128.8, 129.5, 129.8, 130.0, 135.0, 137.7, 141.6, 170.3; HRMS (**EI^+**) calculated for $[C_{15}H_{11}NO]^+$ $(M)^+$ m/z : 221.0841, found 221.0845 (1.8 ppm).

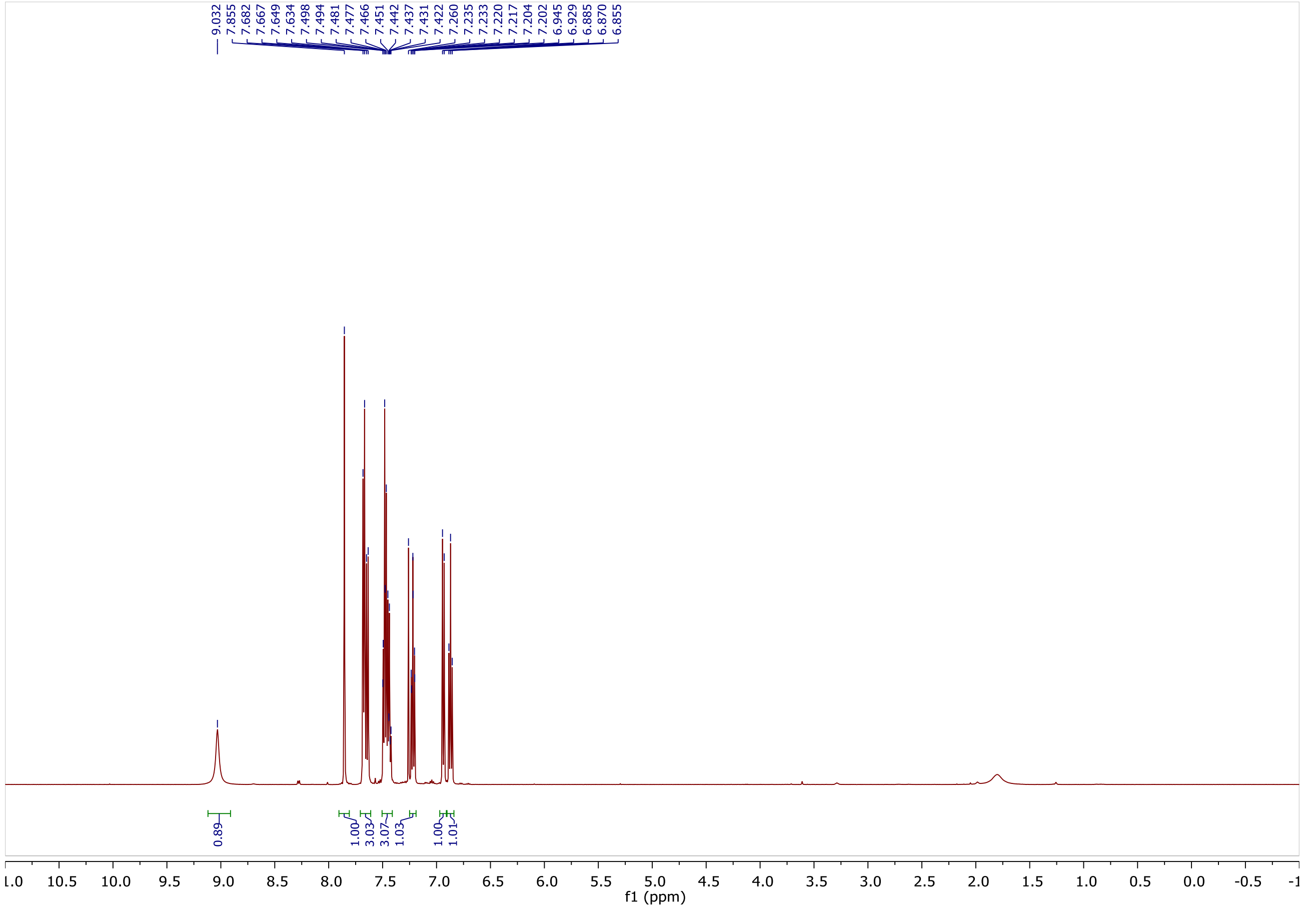

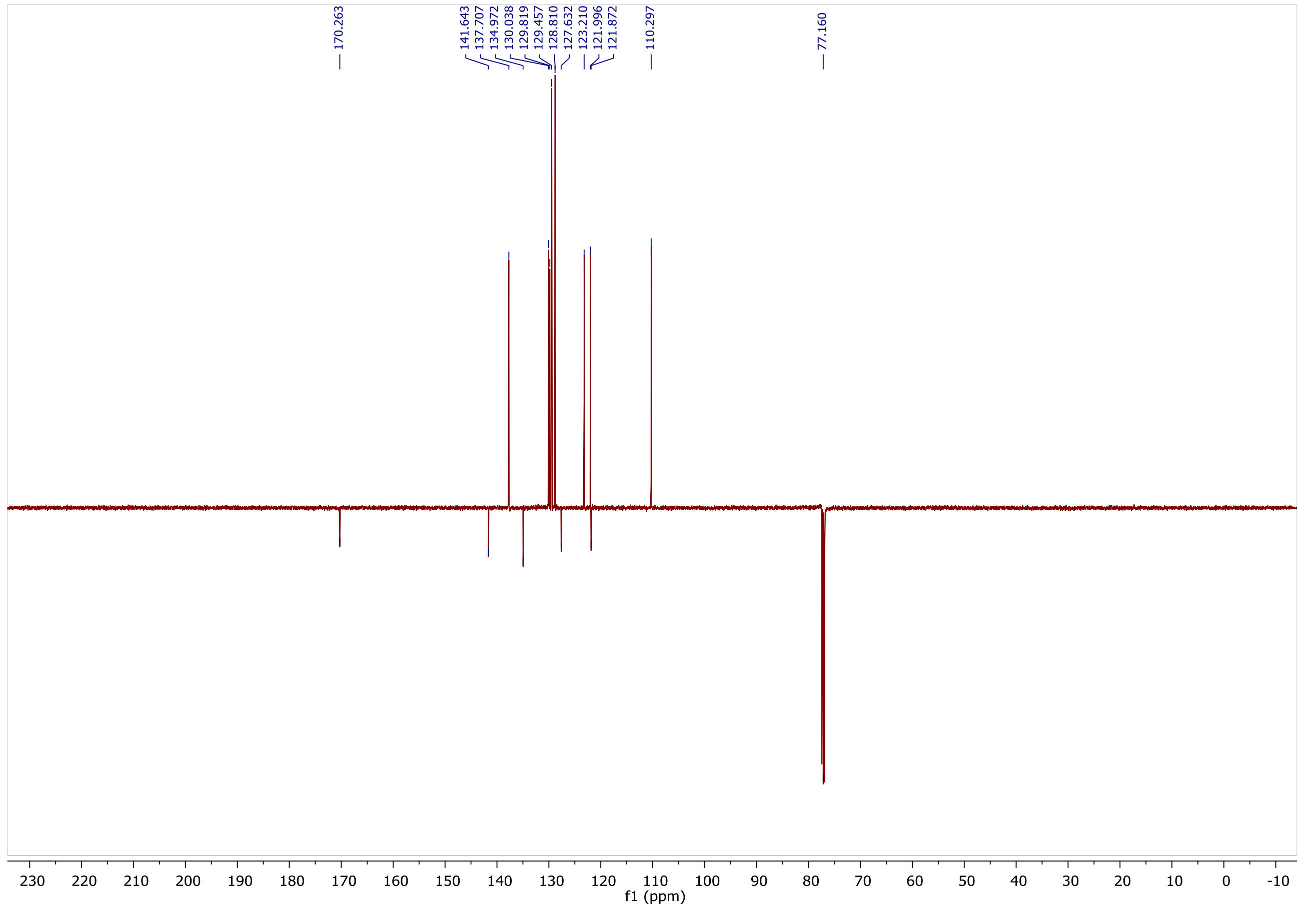

2.5.2. Mechanistic probe

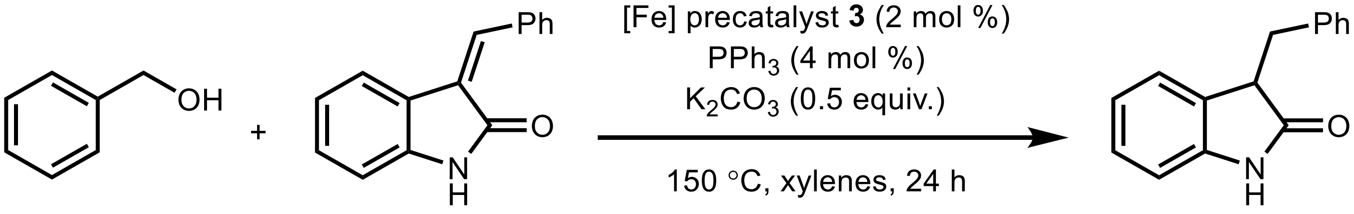

A 10 mL microwave vial equipped with a stirrer bar was charged with 3-benzylideneindolin-2-one (221 mg, 1.0 mmol), K_2CO_3 (69.1 mg, 0.5 mmol, 0.5 equiv.), PPh_3 (10.5 mg, 0.04 mmol, 4 mol %) and [Fe] precatalyst **3** (9.1 mg, 0.02 mmol, 2 mol %). The vial was sealed with a cap and was placed under vacuum. After 5 minutes it was flushed with nitrogen and the cycle repeated three times. Under nitrogen the vial was then charged with xylene (2 mL) and benzyl alcohol (124 µL, 130 mg, 1.2 mmol, 1.2 equiv.). The mixture was left to react at 150 °C for 24 hours. It was then cooled, followed by the addition of mesitylene (139 µL, 120 mg, 1.0 mmol), H_2O (2 mL) and EtOAc (2 mL). Brine (1 mL) was added to aid layer separation. The mixture was stirred for 5 min, left to settle for a further 5 min, cap removed and the top layer was sampled and analysed using ^1H NMR. The result gave a **71%** NMR yield of 3-benzylindolin-2-one.

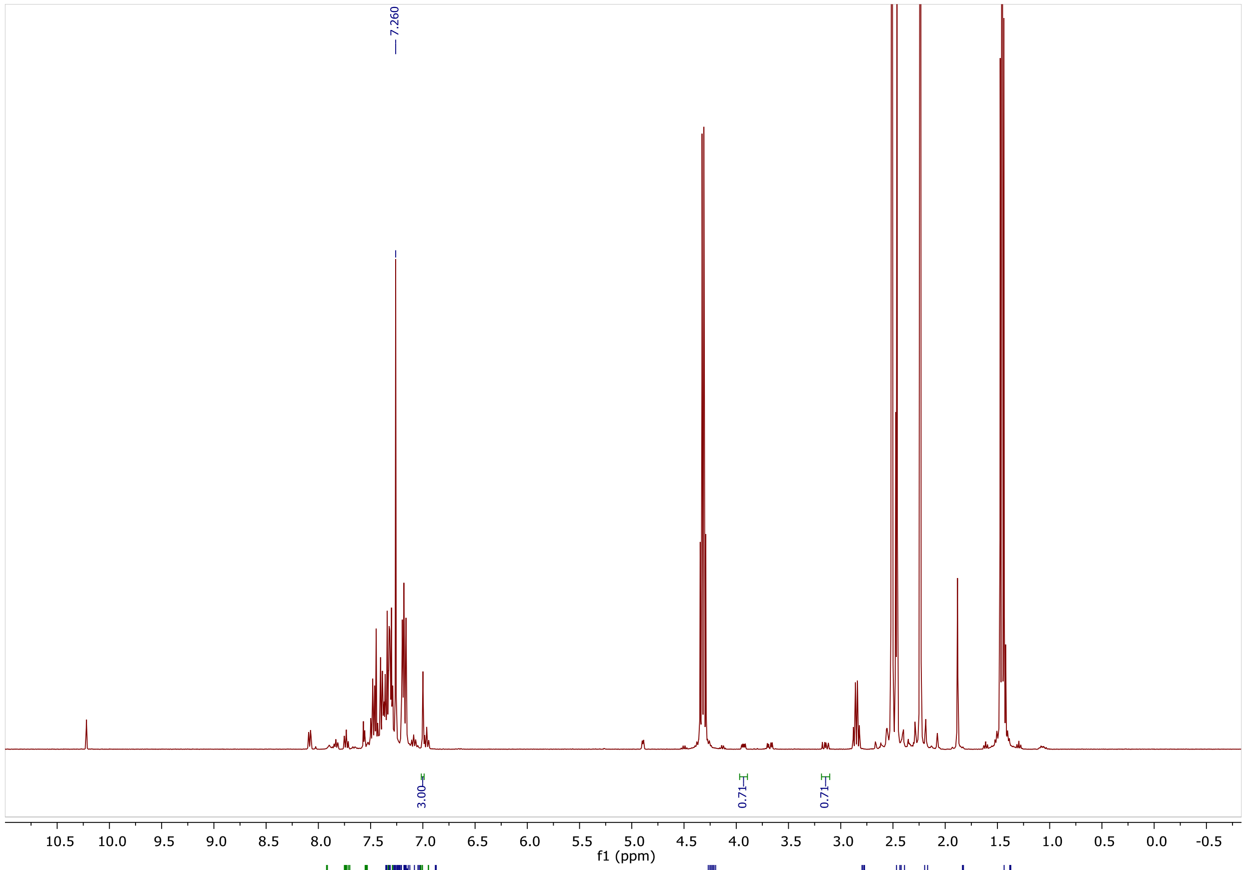
7.260
3.00
0.71
0.71
10.5 10.0 9.5 9.0 8.5 8.0 7.5 7.0 6.5 6.0 5.5 5.0 4.5 4.0 3.5 3.0 2.5 2.0 1.5 1.0 0.5 0.0 -0.5
f1 (ppm)

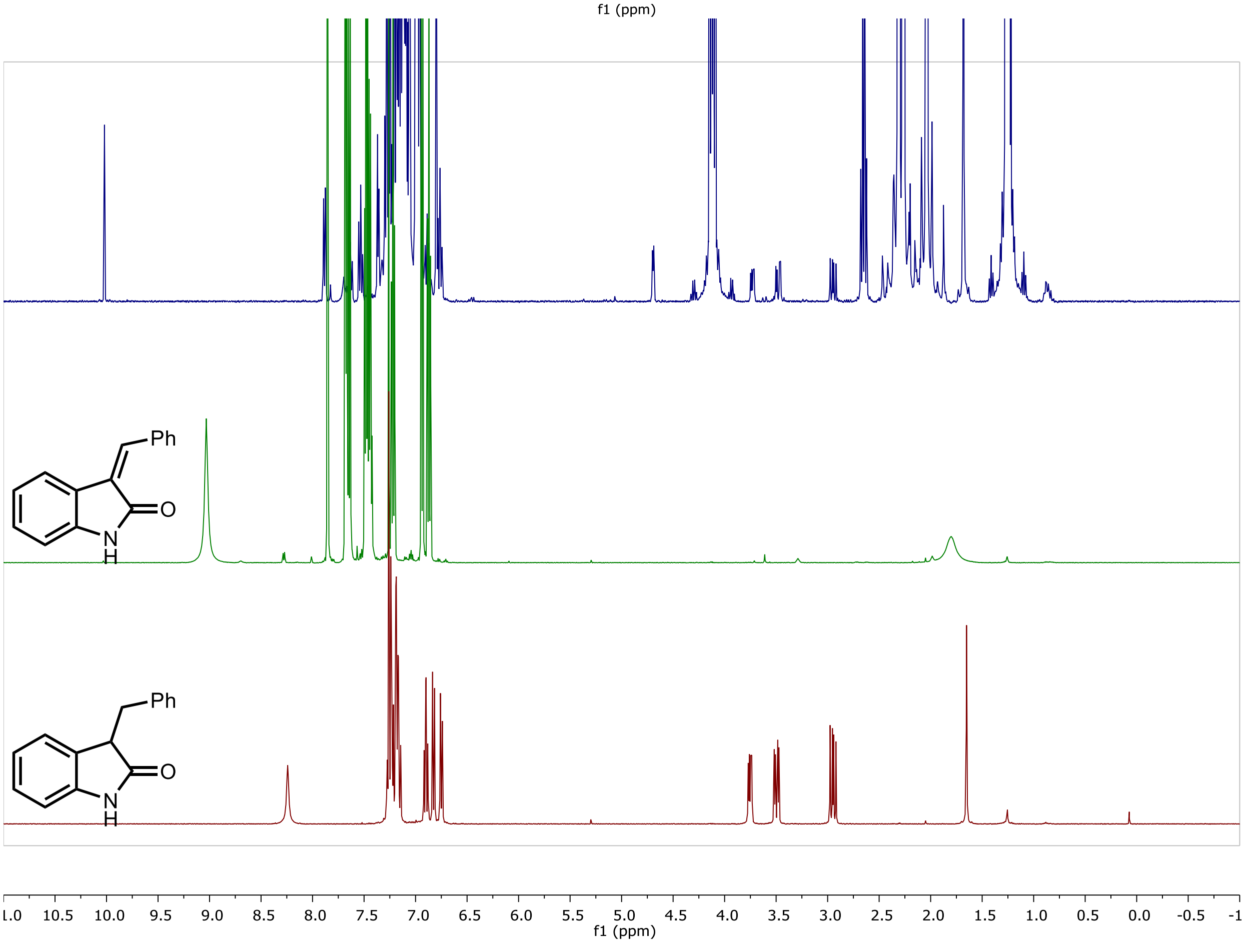
Ph
O
N
H
Ph
O
N
H
10.5 10.0 9.5 9.0 8.5 8.0 7.5 7.0 6.5 6.0 5.5 5.0 4.5 4.0 3.5 3.0 2.5 2.0 1.5 1.0 0.5 0.0 -0.5
f1 (ppm)

3. References

1. Seck, C.; Mbaye, M. D.; Coufourier, S.; Lator, A.; Lohier, J. F.; Poater, A.; Ward, T. R.; Gaillard, S. *ChemCatChem* **2017**, *9*, 4410–4416.
2. Plank, T. N.; Drake, J. L.; Kim, D. K.; Funk, T. W. *Adv. Synth. Catal.* **2012**, *354*, 597–601.
3. Funk, T. W.; Mahoney, A. R.; Sponenburg, R. A.; Zimmerman, K. P.; Kim, D. K.; Harrison, E. E. *Organometallics* **2018**, *37*, 1133–1140.
4. Moulin, S.; Dentel, H.; Pagnoux-Ozherelyeva, A.; Gaillard, S.; Poater, A.; Cavallo, L.; Lohier, J. F.; Renaud, J. L. *Chem. Eur. J.* **2013**, *19*, 17881–17890.
5. Facchini, S. V.; Cettolin, M.; Bai, X.; Casamassima, G.; Pignataro, L.; Gennari, C.; Piarulli, U. *Adv. Synth. Catal.* **2018**, *360,* 1054–1059.
6. Thai, T. T.; Mérel, D. S.; Poater, A.; Gaillard, S.; Renaud, J. L. *Chem. Eur. J.* **2015**, *21*, 7066–7070.
7. Denmark, S. E.; Butler, C. R. *Org. Lett.* **2006**, *8*, 63−66.
8. ; Bevz, O. V; Gorokhova, O. V. *Chem. Heterocyc. Comp*. **2011**, *47*, 833–837.
9. Schofield, C. J.; Yeh, T.; Thinnes, C. C.; Loenarz, C.; Nowak, R. P.; Oppermann, U.; Attwood, M.; Tumber, A.; Abboud, M. I.; Lohans, C. T. *Chem. Eur. J.* **2018**, *25*, 2019–2024.
10. Wang, M.; Han, J.; Si, X.; Hu, Y.; Zhu, J.; Sun, X. *Tetrahedron Lett.* **2018**, *59*, 1614–1618.
11. Manick, A. D.; Berhal, F.; Prestat, G. Development of a One-Pot Four C-C Bond-Forming Sequence Based on Palladium/Ruthenium Tandem Catalysis. *Org. Lett.* **2018**, *20*, 194–197.
12. Jensen, T., Madsen, R. *J. Org. Chem.* **2009**, *74*, 3990-3992.
13. Grigg, R.; Whitney, S.; Sridharan, V.; Keep, A.; Derrick, A. *Tetrahedron* **2009**, *65*, 7468–7473.
14. Xinpeng J., Cong Z., Lijun L., Kai L., Chuanming. Y. *Eur. J. Org. Chem.* **2018**, 1437–1442.
15. Milite, C.; Feoli, A.; Sasaki, K.; La Pietra, V.; Balzano, A. L.; Marinelli, L.; Mai, A.; Novellino, E.; Castellano, S.; Tosco, A.; Sbardella, G. *J. Med. Chem.* **2015**, *58*, 2779–2798.
16. Guyon, C.; Duclos, M. C.; Sutter, M.; Métay, E.; Lemaire, M. *Org. Biomol. Chem.* **2015**, *13*, 7067–7075.
17. Konyar, D.; Andac, C. A.; Buyukbingol, E. *Lett. Drug Des. Discov.* **2018**, *15*, 37-45.
